# Reaction of Nitrogen‐Radicals with Organometallics Under Ni‐Catalysis: N‐Arylations and Amino‐Functionalization Cascades

**DOI:** 10.1002/anie.201900510

**Published:** 2019-03-12

**Authors:** Lucrezia Angelini, Jacob Davies, Marco Simonetti, Laia Malet Sanz, Nadeem S. Sheikh, Daniele Leonori

**Affiliations:** ^1^ School of Chemistry University of Manchester Oxford Road Manchester M13 9PL UK; ^2^ Eli Lilly and Company Limited Erl Wood Manor, Windelesham Surrey GU20 6PH UK; ^3^ Department of Chemistry College of Science King Faisal University P.O. Box 380 Al-Ahsa 31982 Saudi Arabia

**Keywords:** alkaloid, arylation, nitrogen radicals, radical cyclization, single electron transfer

## Abstract

Herein, we report a strategy for the generation of nitrogen‐radicals by ground‐state single electron transfer with organyl–Ni^I^ species. Depending on the philicity of the N‐radical, two types of processes have been developed. In the case of nucleophilic aminyl radicals direct N‐arylation with aryl organozinc, organoboron, and organosilicon reagents was achieved. In the case of electrophilic amidyl radicals, cascade processes involving intramolecular cyclization, followed by reaction with both aryl and alkyl organometallics have been developed. The N‐cyclization–alkylation cascade introduces a novel retrosynthetic disconnection for the assembly of substituted lactams and pyrrolidines with its potential demonstrated in the short total synthesis of four venom alkaloids.

Nitrogen‐radicals are versatile synthetic intermediates that can engage in a broad range of chemical reactions.^1^ In general, they are used in intramolecular cyclizations,^1^ intramolecular 1,5‐H‐atom abstractions (HATs),^2^ and intermolecular additions to π‐systems^3^ (Scheme 1 A). A considerably underdeveloped reactivity stream is their engagement in transition metal‐based processes, an interplay heavily exploited with C‐radicals (Scheme 1 B).^4^ Indeed, the aptitude of N‐radicals to undergo SET transmetalation with organometallic complexes has been scarcely adopted,^5^ and its implementation has the capability to unlock unique reactivity patterns. Given the relevance of nitrogenated molecules as therapeutic agents and agrochemicals, methods facilitating their construction are of high importance in both academia and industry.^6^

**Scheme 1:**
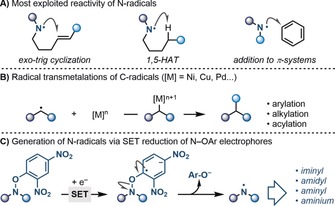
A) Reactivity of N‐radicals. B) SET transmetalation of C‐radicals. C) Generation of N‐radicals by SET reduction of *N*‐OAr hydroxylamines.

We have started a research program aimed at downstreaming N‐radicals using highly electron poor *O*‐aryl hydroxylamine derivatives (Scheme 1 C).^7^ These substrates can, under photoredox conditions,^8^ provide access to iminyl,7d amidyl,7c and aminium7b radicals for cyclization and aromatic C−H amination. Crucial to the success of these processes is the high reduction potential of these precursors (*E* ^red^≈−0.8 V vs. SCE) as this enables facile SET (single‐electron transfer) reduction by the excited state of many photoredox catalysts.

As part of an effort to expand the synthetic utility of these N‐radical precursors, we envisaged that their SET reduction could be achieved using ground‐state transition metal complexes. We were particularly interested by reports from the Vicic^9^ and Doyle^10^ groups demonstrating how organyl–Ni^I^ species can act as competent reductant (*E* ^ox^≈−1.1 V vs. SCE), thus making a ground‐state SET with our derivatives potentially exergonic (Δ*G*_SET_≈−4 kcal mol^−1^). Specifically, we questioned if these precursors could be used in Ni‐catalyzed umpolung aminations and amidations of aryl organometallic reagents.^11^ The preparation of tertiary anilines by umpolung strategies has received considerable attention in the last few years.^12^ However, methods based on Ni‐catalysis^13^ have only been demonstrated on aryl organozincs, thus a general strategy able to engage easy to handle organometallics of boron and silicon has yet to be reported.

Our proposed catalytic cycle would start with the transmetalation of a Ni^I^ species **A** with an aryl organometallic to give an aryl‐Ni^I^ complex **B** (Scheme 2 A). At this point, an energetically favorable SET with the N‐radical precursors **C** would deliver the aryl‐Ni^II^ intermediate **D** and the N‐radical **E**. Our mechanistic picture hinged on the ability of **E** to intercept **D** and provide a N‐bound aryl‐Ni^III^ complex **F**. As related alkyl‐Ni^III^‐amides have been shown by Hillhouse^14^ to undergo facile reductive elimination, we predicted the latter to generate the desired product **G** and a Ni^I^ species **A** thus closing the catalytic cycle.

**Scheme 2:**
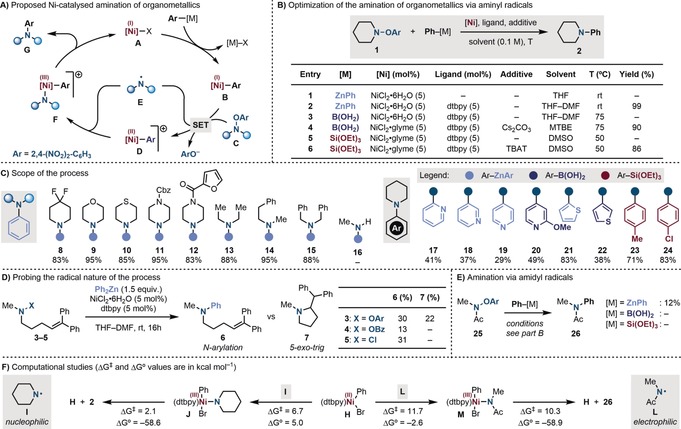
A) Proposed catalytic cycle for a Ni‐catalyzed amination of organometallics by N‐radicals. B) Reaction optimization using piperidine **1**. C) Scope of the process. D) Radical clock experiments. E) Amination of organometallics by amidyl radicals. F) DFT studies [UB3LYP/6‐31G(d)‐LANL2DZ].^21^

In light of the wealth of organometallics utilized in Ni catalysis,^15^ we assessed the stability of our precursor (**1**) in the presence of several potential coupling partners. We discovered **1** to be stable in the presence of diarylzincs, aryl boronic acids, and aryl silanes up to high temperature (*T*≈100 °C) but to decompose by addition of aryl Grignards. We thus considered the development Ni‐catalyzed umpolung aminations of *N*,*N*‐*O*‐aryl‐hydroxylamine derivatives as the redox active coupling partners with aryl zincs, boronic acids and silanes.

We started the optimization of the N‐arylations reaction using piperidine **1** (prepared on multi‐gram scale in 1 step) (Scheme 2 B).^16^ By using Ph_2_Zn as the coupling partner, efficient amination (**1**→**2**) could be achieved employing 5 mol % of NiCl_2_⋅6 H_2_O and dtbbpy ligand in THF–DMF at room temperature. Both the ligand and the DMF had a critical role in the success of the reaction (entries 1–2). Replacing the catalytic system with the preformed dttbpy⋅NiCl_2_ catalyst gave **2** in almost identical yield. As organozinc reagents can be obtained by transmetalation of Grignards with ZnCl_2_ we also evaluated the use of PhZnCl, which gave **2** in high yield.^16^

Next, the amination was investigated with PhB(OH)_2_.^17^ A similar Ni‐ligand system could be used, however the addition of Cs_2_CO_3_ was critical, likely by easing the transmetalation of the boronic acid at the Ni^I^ center (entries 3,4). Also in this case, the solvent played an important role and optimum yields were obtained in MTBE (entry 6).^16^ The implementation of aryl silanes proved more difficult and a stoichiometric fluoride source was required to promote reactivity.^18^ Specifically, when PhSi(OEt)_3_ was used in combination with CsF and TBAF, a complex mixture of products was observed. Conversely, the use of TBAT provided **2** in high yield (entry 5,6). Other trialkyl‐phenyl silanes were evaluated, but this reactivity could not be extended to other derivatives.^16^

With the optimized conditions, we evaluated the scope of this umpolung aromatic amination strategy (Scheme 2 C). Using Ph_2_Zn we screened several aminyl precursors and found the amination to be compatible with a functionalized piperidine (**8**), morpholine (**9**), thiomorpholine (**10**), piperazines (**11**, **12**), as well as dialkyl amines (**13**–**15**). At the moment this reactivity is limited to the installment of secondary amines as efforts directed towards the use of primary amines proved unsuccessful (**16**). Pleasingly, we could access C‐2 (**17**), C‐3 (**18**), and C‐4 (**19**) aminated pyridines using both aryl organozincs and a boronic acid (**20**) in good to moderate yields. C‐2 (**21**) and C‐3 (**22**) amination of thiophene was also possible in high and moderate yields, respectively. Two commercially available silanes were also engaged in the reaction with **1** (**23**, **24**). The successful formation of product **18**–**21** is noteworthy as, to the best of our knowledge, their preparation by umpolung or Chan–Lam strategies on zincated/borylated pyridines and zincated 5‐membered ring heterocycles has not been reported before.^19^

We believe this umpolung amination strategy to be mechanistically distinct to known protocols based on N−Cl and N−OBz reagents, as pioneered by Johnson and Jarvo.^13^ Indeed, the low redox potentials of N−Cl and N−OBz piperidines (*E* ^red^≈−1.9 V and −1.8 vs. SCE respectively) would result in endergonic SET reductions. This suggests that a two‐electron process [Ni^0^/Ni^II^ cycle] is operating in processes based on those reagents while a distinct radical reactivity characterizes our strategy with N−OAr derivatives. To validate this mechanistic hypothesis, we tested N‐reagents **3**, **4** and **5**, which feature N−OAr, N−OBz, and N−Cl bonds, and can be used as radical‐clock probes.^20^ As shown in Scheme 2 D, although the N‐arylation product **6** was obtained in all cases, product **7**, which would result from a radical 5‐*exo*‐*trig* cyclisation, was exclusively obtained when using **3**, regardless of the nature of the organometallic partner (entries 1–6), which supports our mechanistic framework.^22^

Having demonstrated the ability of aminyl radicals to participate in the amination process, we evaluated the use of amidyl radicals. However, despite attempting re‐optimization, the use of oxyamide **25** was not possible and the desired *N*‐Ph‐acetamide **26** was obtained in low yield only in combination with Ph_2_Zn (Scheme 2 E). We propose the different behavior of N‐radical precursors **1** and **25** to be related to the different philicity of their respective N‐radicals. In fact, while aminyls have a nucleophilic character (calculated electrophilicity indexes,^23^ *ω* ^+^_rc_=+0.72 eV), amidyls are electrophilic (*ω* ^+^_rc_=+1.2 eV).^16^ This might prevent their SET transmetalation with aryl‐Ni^II^ species. In order to support this mechanistic proposal, we performed DFT studies evaluating the feasibility of N‐radical transmetalation and subsequent reductive elimination using (dtbpy)Ni^II^(Ph)Br **H** as the model substrate. As shown in Scheme 2 F, the reaction profile associated with aminyl **I** revealed a low activation barrier for the radical transmetalation (**H** + **I** → **J**), which is slightly endothermic, and an almost barrier‐less and highly exergonic reductive elimination (**J** → **H + 2**). Conversely, both radical transmetalation (**H** + **L** → **M**) and reductive elimination (**M** → **H + 26**) using amidyl **L**, have significantly higher barriers, which might explain the low reactivity of **25**.

Given the unsuccessful development of umpolung aminations by amidyl radicals, we wondered if this lack of reactivity could be harnessed to develop radical cascade processes. Specifically, we targeted the development of a multicomponent reaction starting with the amidyl radical **O** generated by reductive SET of the aryloxy amides **N** from **B** (Scheme 3 A). At this point, as the N‐radical transmetalation is disfavored, a fast intramolecular 5‐*exo‐trig* cyclization (*k*≈10^9^ m ^−1^)^24^ would convert the electrophilic amidyl **O** into the nucleophilic C‐radical **P**. This species should now possess the matched philicity to enter the catalytic cycle by reacting with **D**. Reductive elimination from di‐organyl–Ni^III^ species **Q** would generate the amido‐arylated product **R**. It is worth pointing out that, while Pd^0^‐catalyzed processes achieving related transformations using amides, carbamates and their derivatives in conjunction with aryl halides have been developed,^25^ a radical umpolung counterpart has, to the best of our knowledge, not been reported yet.^26^

**Scheme 3:**
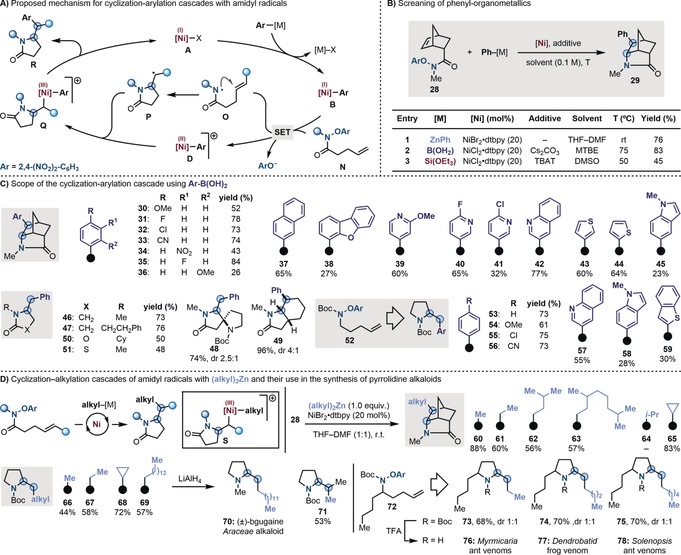
A) Proposed catalytic cycle for a Ni‐catalyzed cyclization–arylation by N‐radicals. B) Optimization of the process. C) Scope of the process. D) Cascade cyclization–alkylation of amidyl radicals and applications in the synthesis of pyrrolidine alkaloids.

Having this mechanistic framework in mind, we prepared and tested the norbornene precursor **28** (Scheme 3 B). Pleasingly, by using conditions similar to the ones developed for the N‐arylations, we successfully accessed arylated lactam **29** (*exo* isomer) with all three organometallic reagents. In this case, as the use of PhB(OH)_2_ proved optimum, we evaluated the aromatic scope by taking advantage of the vast range of commercially available boronic acids. As shown in Scheme 3 C, both electron‐rich and electron‐poor (**30**–**33**) partners were successfully engaged, as well as substrates containing handles for further functionalization at either *para* and *meta* positions (**34**–**35**). *ortho*‐Substituents were tolerated, albeit with decreased efficiency (**36**). This reaction has also enabled the installation of other (hetero)aromatics (**37**–**38**), including substituted pyridines (**39**–**41**), quinoline (**42**), C2‐ and C3‐thiophenes (**43**–**44**), and *N*‐Me‐indole (**45**). The scope regarding the amidyl radical substitution pattern was run in conjunction with PhB(OH)_2_. The process allowed the construction of simple 5‐membered ring lactams (**46**–**47**), proving that cascades centered on primary radicals are feasible.^27^ The formation of spirocyclic and bicyclic derivatives (**48**–**49**) was possible as well as the extension of this methodology to strongly electrophilic (thio)carbamoyl radicals (**50**–**51**). Next, we prepared radical precursor **52** with the aim of accessing α‐benzyl‐pyrrolidines, a class of biologically active heterocycles. Gratifyingly, this approach enabled the fast assembly of a small library in good to moderate yields, spanning electron neutral (**53**), electron rich (**54**), electron poor (**55** and **56**), and heteroaromatic derivatives (**57**–**59**).

Finally, we questioned if the presented cyclization–functionalization strategy could be extended to alkyl organometallic reagents (Scheme 3 D).^28^ Mechanistically, we were intrigued by the possibility of accessing dialkyl‐Ni^III^ intermediate **S**, which would ultimately lead to a N‐cyclization–alkylation process. This strategy would deliver the concomitant formation of vicinal sp^3^ C−N and sp^3^–sp^3^ C−C bonds, a transformation that, to the best of our knowledge, has not been possible by either ionic, transition metal‐mediated, or radical pathways.

After a screening of alkyl organometallics and reaction conditions using **28**, we found that this cascade could be unlocked using dialkylzinc reagents, and the preformed catalyst NiBr_2_⋅dtbbpy.^16^ The scope of the process was evaluated and we were able to achieve lactamisation–methylation, ethylation, and *i*‐butylation (**60**–**62**) cascades in good to high yields, as well as introducing a 3,7‐dimethyloctyl alkyl chain (**63**). Currently, the use of more sterically hindered secondary organozincs (such as *i*‐Pr_2_Zn, **64**) is a limitation with the exception of the sp^2^‐like cyclopropyl unit, which was introduced in high yield (**65**).

Finally, we extended this reactivity to **52** with the aim to build 2‐alkyl‐pyrrolidines, a motif found in many highly biologically active natural products.^29^ Pleasingly, we successfully achieved cyclization–alkylation cascades introducing methyl, ethyl, and cyclopropyl groups (**66**–**68**). By using ditridecyl zinc, we accessed **69** that upon *N*‐Boc reduction gave the *Aracae* alkaloid (±)‐bgugaine^30^ **70** in just 2 steps. Substrates containing di‐substituted olefins are also amenable to this reactivity as demonstrated by the successful formation of **71** where two contiguous tertiary centers have been assembled by the radical cascade.

As the N‐radical precursors are easily assembled by Mitsunobu reaction between the corresponding alcohol and the Boc‐NH(OAr) reagent,^16^ we prepared **72** on gram scale. This derivative was diversified by reaction with diethyl, di‐*n*‐butyl, and di‐*n*‐hexyl zinc reagents to give pyrrolidines **73**–**75** in good yields but 1:1 *syn*:*anti* diasteroselectivity. This strategy allowed, following *N*‐Boc removal, the synthesis of the *Myrmicaria melanogaster* ant venoms **76**,^31^ the *Dendrobatib* frog venoms **77**,^32^ and the *Solenopsis fugax* ant venoms **78**.

In conclusion, we have successfully developed cascade reactions were N‐radicals are generated by ground‐state SET with aryl‐ and alkyl‐Ni^I^ species. Based on the philicity of the N‐radical generated, direct transmetalation to organyl‐Ni^II^ species or cyclization and then transmetalation could be achieved. Overall this reactivity has enabled N‐arylation, amino‐arylation and for the first time, amino‐alkylation cascades using readily available aryl and alkyl organometallic reagents.

## Conflict of interest

The authors declare no conflict of interest.

## Supporting information

As a service to our authors and readers, this journal provides supporting information supplied by the authors. Such materials are peer reviewed and may be re‐organized for online delivery, but are not copy‐edited or typeset. Technical support issues arising from supporting information (other than missing files) should be addressed to the authors.

Supplementary
