# Supplementary material for: Reaction of Nitrogen‐Radicals with Organometallics Under Ni‐Catalysis: N‐Arylations and Amino‐Functionalization Cascades
Source: Angew Chem Int Ed Engl. 2019 Mar 12;58(15):5003–7. doi: 10.1002/anie.201900510 (PMC6519068; doi:10.1002/anie.201900510)
Supplement: Supplementary file 1 — Supplementary [file ANIE-58-5003-s001.pdf]

Angewandte Chemie
Eine Zeitschrift der Gesellschaft Deutscher Chemiker

Supporting Information

Reaction of Nitrogen-Radicals with Organometallics Under Ni-Catalysis: N-Arylations and Amino-Functionalization Cascades

*Lucrezia Angelini[+], Jacob Davies[+], Marco Simonetti, Laia Malet Sanz, Nadeem S. Sheikh, and Daniele Leonori**

anie_201900510_sm_miscellaneous_information.pdf

1 Table of Contents

2 General Experimental Details 2

3 Starting Material Synthesis 3

3.1 Synthesis of Known Compounds 3

3.2 Preparation of Organozinc Reagents 11

4 Umpolung *N*-Arylations and Cyclization–Cascades 12

4.1 Reactions Optimization 12

4.2 GP3 – General Procedure for *N*-arylation and cyclization-arylation with organozincs 19

4.3 GP4 – General Procedure for *N*-arylation and cyclization-arylation with aryl boronic acids 19

4.4 GP5 – General Procedure for *N*-arylation and cyclization-arylation with organo silanes 20

5 Analysis of Chan-Lam Couplings with Heteroaromatic Organometallics 21

6 Mechanistic Considerations 22

7 Products Characterization 24

8 Computational Studies 51

8.1 Computational Methods 51

8.2 Activation Energy (DG‡) and Reaction Energy (DG) for Radical Additions and Reductive Elimination Reactions 52

9 NMR Spectra 65

10 References 122

2 General Experimental Details

All required fine chemicals were used directly without purification unless stated otherwise. All air and moisture sensitive reactions were carried out under nitrogen atmosphere using standard Schlenk manifold technique. THF was distilled from sodium/benzophenone, CH_2Cl_2 and was distilled from CaH_2, CH_3CN was distilled from activated 4Å molecular sieves, EtN(*i*-Pr)$_2$ was distilled over KOH. ^{1}H and ^{13}C Nuclear Magnetic Resonance (NMR) spectra were acquired at various field strengths as indicated and were referenced to $CHCl_3$ (7.26 and 77.0 ppm for ^{1}H and ^{13}C respectively). ^{1}H NMR coupling constants are reported in Hertz and refer to apparent multiplicities and not true coupling constants. Data are reported as follows: chemical shift, integration, multiplicity (s = singlet, br s = broad singlet, d = doublet, t = triplet, q = quartet, qi = quintet, sx = sextet, sp = septet, m = multiplet, dd = doublet of doublets, etc.), proton assignment (determined by 2D NMR experiments: COSY, HSQC and HMBC) where possible. High-resolution mass spectra were obtained using a JEOL JMS-700 spectrometer or a Fissions VG Trio 2000 quadrupole mass spectrometer. Spectra were obtained using electron impact ionization (EI) and chemical ionization (CI) techniques, or positive electrospray (ES). Infra-red spectra were recorded using a JASCO FT/IR 410 spectrometer or using an ATI Mattson Genesis Seris FTIR spectrometer as evaporated films or liquid films. Analytical TLC: aluminum backed plates pre-coated (0.25 mm) with Merck Silica Gel 60 F254. Compounds were visualized by exposure to UV-light or by dipping the plates in permanganate ($KMnO_4$) stain followed by heating. Flash column chromatography was performed using Merck Silica Gel 60 (40–63 μm). All mixed solvent eluents are reported as v/v solutions.

3 Starting Material Synthesis

3.1 Synthesis of Known Compounds

Compounds **19** and **S1–9** have been prepared according to previously reported procedures. [1–3]

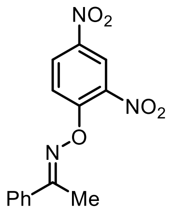

24

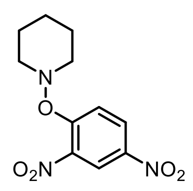

S1

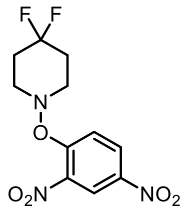

S2

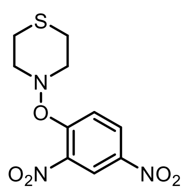

S3

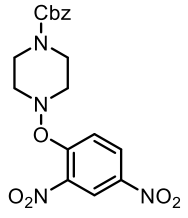

S4

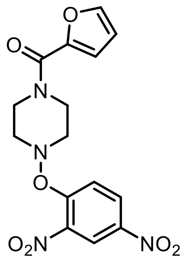

S5

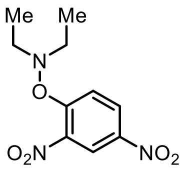

S6

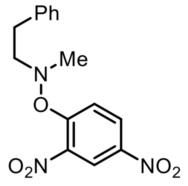

S7

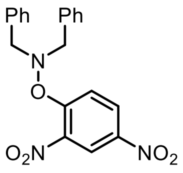

S8

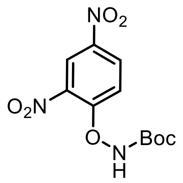

S9

Catalysts [nickel(II)(4,4'-di-*tert*-butyl-2,2'-bipyridine)(chloride)$_2$] **(C1)** and [nickel(II) (4,4'-di-*tert*-butyl-2,2'-bipyridine)(bromide)$_2$] **(C2)** have been prepared according to literature procedures. [4-5]

(4-(2,4-Dinitrophenoxy)piperazin-1-yl)(furan-2-yl)methanone (S9)

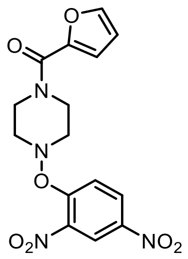

Following the reported procedure,[2] furan-2-yl(piperazin-1-yl)methanone (1.0 g, 5.5 mmol), gave **S9** (557 mg, 28%) as an orange solid. FT-IR ν_{max} (film)/cm^{-1} 2974, 2876, 2360, 1695, 1607, 1553, 1480, 1447, 1393, 1318, 1241, 1217, 1187, 1154, 1046; ^{1}H NMR (400 MHz, $CDCl_3$) δ 8.83 (1H, d, *J* = 2.1 Hz), 8.43 (1H, dd, *J* = 9.3, 2.1 Hz), 7.91 (1H, d, *J* = 9.3 Hz), 7.51 (1H, s), 7.10 (1H, d, *J* = 3.4 Hz), 6.58–6.46 (1H, m), 4.47 (2H, d, *J* = 13.4 Hz), 3.65 (2H, br s), 3.42 (2H, d, *J* = 11.1 Hz), 3.19 (2H, t, *J* = 9.9 Hz); ^{13}C NMR (101 MHz, $CDCl_3$) δ 159.1, 157.2, 147.6, 144.2, 140.9, 136.8, 129.6, 122.3, 117.6, 116.8, 111.8, 55.7; HRMS (ESI) Found MNa^+ 446.1898 $C_{20}H_{29}N_3O_7Na$ requires 446.1890.

4-(2,4-Dinitrophenoxy)morpholine (S10)

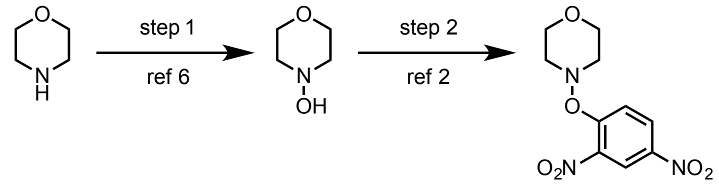

Step1. Following the reported procedure,[6] morpholine (1.01 g, 10 mmol, 1.0 equiv.) gave morpholin-4-ol (685 mg, 85%) as an oil. ^{1}H NMR (500 MHz, $CDCl_3$, OH missing) δ 4.06 (2 H, dt, J = 13.1, 3.4 Hz), 3.75 (2H, ddd, J = 12.6, 10.2, 2.4 Hz), 3.29 (2H, dt, J = 12.5, 2.9 Hz), 3.04 (2H, ddd, J = 11.8, 10.2, 3.4 Hz); ^{13}C NMR ($CDCl_3$, 126 MHz) δ 22.3, 22.4, 44.4. Data in accordance with the literature[6].

Step 2. Following the reported procedure,[2] morpholin-4-ol (685 mg, 6.6 mmol), gave **S10** (710 mg, 40 %) as an orange solid. ^{1}H NMR (400 MHz, $CDCl_3$) δ 8.80 (1H, d, J = 2.7 Hz), 8.40 (1H, dd, J = 9.4, 2.7 Hz), 7.89 (1H, d, J = 9.4 Hz), 4.06 (2H, br d, J = 12.2 Hz), 3.75 (2H, ddd, J = 12.2, 10.3, 2.3 Hz), 3.48–3.29 (2H, m), 3.15 (2H, td, J = 10.6, 3.3 Hz); ^{13}C NMR (101 MHz, $CDCl_3$) δ 157.2, 140.6, 137.6, 129.3, 122.1, 116.7, 65.7, 56.5. Data in accordance with the literature.[2]

***N*-Methyl-5,5-diphenylpent-4-en-1-amine (S11)**

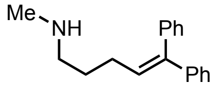

S11 has been prepared according to the reported procedure.[7]

***O*-Benzoyl-*N*-(5,5-diphenylpent-4-en-1-yl)-*N*-methylhydroxylamine (4)**

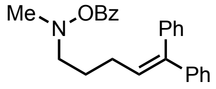

To a solution of *N*-methyl-5,5-diphenylpent-4-en-1-amine **S11** (5.03 g, 20 mmol, 1.0 equiv.) in DMF (100 mL, 0.2 M), K_2HPO_4 (6.97 g, 40 mmol, 2.0 equiv.) and BPO (wet with 25% of water, 6.66 g, 1.1 equiv.) were loaded and the reaction was vigorously stirred at room temperature for 10 h. After this time, ice (100 g) was added to the mixture and the crude was diluted with EtOAc (350 mL) and $NaHCO_3$ (100 mL). The organic phase was washed with water (2×), brine, dried over magnesium sulphate and concentrate under reduced pressure to afford a crude oil. Purification by column chromatography on silica gel eluting with [petrol:EtOAc (90:10)] gave **4** (58%) as a white solid. FT-IR ν_{max} (film)/cm^{-1} 3058, 3023, 2935, 2845, 1739, 1599, 1495, 1450, 1442, 1259, 1175, 1081, 1061, 1024; ^{1}H NMR (500

MHz, $CDCl_3$) δ 8.00 (2H, dd, *J* = 8.3, 1.4 Hz), 7.62–7.53 (1H, m), 7.49–7.41 (2H, m), 7.38–7.32 (2H, m), 7.31–7.28 (1H, m), 7.28–7.22 (2H, m), 7.22–7.18 (3H, m), 7.16 – 7.11 (2H, m), 6.07 (1H, t, *J* = 7.4 Hz), 2.99–2.92 (2H, m), 2.87 (3H, s), 2.20 (2H, q, *J* = 7.5 Hz), 1.77 (2H, p, *J* = 7.6 Hz); ^{13}C NMR (126 MHz, $CDCl_3$) δ 165.3, 142.7, 142.4, 140.1, 133.2, 129.9, 129.6, 129.4, 129.0, 128.5, 128.3, 128.2, 127.3, 127.1, 127.0, 60.9, 47.2, 31.1, 27.6.

N-(5,5-Diphenylpent-4-en-1-yl)-*N*-methylhydroxylamine (S12)

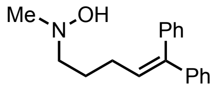

K_2CO_3 (2.76 g, 20 mmol, 2.0 equiv.) was loaded to a solution of *O*-benzoyl-*N*-(5,5-diphenylpent-4-en-1-yl)-*N*-methylhydroxylamine **4** (3.72 g, 10 mmol) in MeOH (100 mL, 0.1 M) and the reaction was stirred at room temperature for 2 h. After this time, ice (50 g) and NH_4Cl (100 mL) were added to the miixture and the organic solvent was removed under reduced pressure. The remaining aqueous phase was extracted with EtOAc (3×) and the organic phase was washed brine, dried over magnesium sulphate and concentrate under reduced pressure to provide a crude oil. Purification by column chromatography on silica gel eluting with [petrol:EtOAc (70:30)] gave **S12** (85%) as a white solid. FT-IR ν_{max} (film)/cm^{-1} 3205, 3052, 3020, 2949, 2844, 2358, 2342, 1597, 1495, 1442, 1073, 1029; ^{1}H NMR (500 MHz, $CDCl_3$) δ 7.48–7.02 (10H, m), 6.09 (1H, t, *J* = 7.5 Hz), 2.60 (3H, s), 2.64–2.58 (2H, m), 2.16 (2H, q, *J* = 7.5 Hz), 1.71 (2H, p, *J* = 7.5 Hz); ^{13}C NMR (126 MHz, $CDCl_3$) δ 142.7, 142.2, 140.3, 130.0, 129.4, 128.3, 128.2, 127.3, 127.1, 127.0, 61.9, 48.8, 27.8, 27.6.

O-(2,4-dinitrophenyl)-*N*-(5,5-diphenylpent-4-en-1-yl)-*N*-methylhydroxylamine (3)

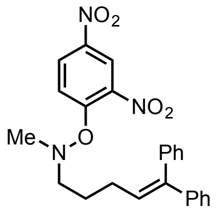

To a solution of *N*-(5,5-diphenylpent-4-en-1-yl)-*N*-methylhydroxylamine **S12** (1.34 g, 5 mmol) and 1-fluoro-2,4-dinitrobenzene (1.86 g, 10 mmol, 2.0 equiv.) in degassed and anhydrous DCM (25 mL, 0.2 M) was added anhydrous NEt_3 (1.74 mL, 12.5 mmol, 2.5 equiv.) and the reaction was stirred under nitrogen at room temperature for 16 h. After this time, the solvent was evaporated, then the crude was diluted with EtOAc (150 mL), the

organic phase was washed with $NaHCO_3$ (2×), brine, dried over magnesium sulphate and concentrate under reduced pressure to provide a crude oil. Purification by column chromatography on silica gel eluting with [petrol:EtOAc (85:15)] gave **3** (35%) as an off-white solid. FT-IR ν_{max} (film)/cm^{-1} 1603, 1524, 1459, 1470, 1442, 1340, 1315, 1271, 1139, 1065; ^{1}H NMR (500 MHz, $CDCl_3$) δ 8.75 (1H, d, *J* = 2.7 Hz), 8.22 (1H, dd, *J* = 9.3, 2.7 Hz), 7.55 (1H, d, *J* = 9.3 Hz), 7.43–7.31 (3H, m), 7.28–7.21 (3H, m), 7.18 (2H, d, *J* = 6.7 Hz), 7.12 (2H, d, *J* = 6.5 Hz), 6.00 (1H, t, *J* = 7.5 Hz), 3.16 – 2.85 (2H, m), 2.90 (1H, br s), 2.80 (3H, s), 2.18 (2H, q, *J* = 7.4 Hz), 1.68 (2H, br s); ^{13}C NMR (126 MHz, $CDCl_3$) δ 158.3, 142.8, 142.4, 140.2, 140.1, 136.2, 130.0, 129.5, 128.5, 128.3, 128.3, 127.3, 127.2, 127.1, 122.1, 116.8, 60.5, 46.6, 31.1, 27.4, 27.1.

_N_-Chloro-_N_-methyl-5,5-diphenylpent-4-en-1-amine (5)

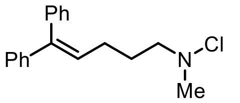

To a solution of *N*-methyl-5,5-diphenylpent-4-en-1-amine **S11** (377 mg, 1.5 mmol, 1.5 equiv.) in CH_2Cl_2 (10 mL, 0.1 M), NCS (133.5 mg, 1.0 mmol, 1.0 equiv.) was added and the reaction was stirred at room temperature for 15 min. The solvent was evaporated and the crude was filtered on a short plug of Al_2O_3 eluting with CH_2Cl_2. The organic solvent was evaporated, pentane (2 mL) was added and the crude was filtered on a short plug of Celite. The filtrate was evaporated to give **5** (75%) as an oil. ^{1}H NMR (500 MHz, $CDCl_3$) δ 7.37 (2H, t, *J* = 7.8 Hz), 7.33–7.27 (2H, m), 7.25–7.19 (4H, m, 2H), 7.18–7.15 (2H, m), 6.08 (1H, t, *J* = 7.5 Hz), 2.90 (3H, s), 2.88–2.82 (2H, m), 2.17 (2H, q, *J* = 7.5 Hz), 1.78 (2H, p, *J* = 8.1, 7.6 Hz).

Compounds **S13–18** have been prepared according to previously reported procedures.[8]

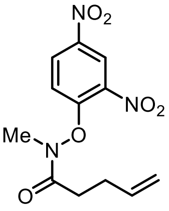

S13

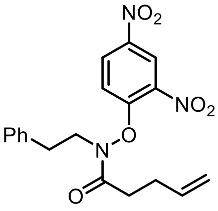

S14

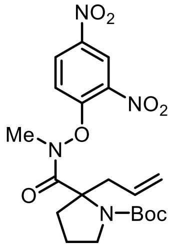

S15

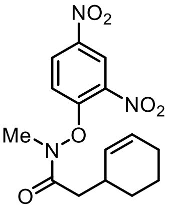

S16

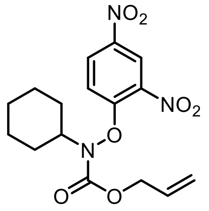

S17

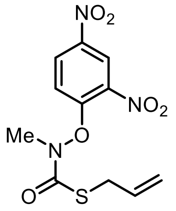

S18

Synthesis of 28 and 60. General Procedure 1 – GP1

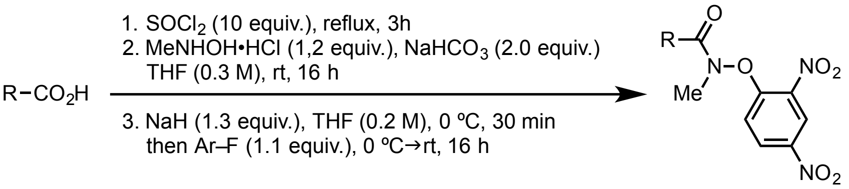

The appropriate carboxylic acid (20 mmol) was loaded in a 150 mL flask equipped with a condenser and a stirring bar, then $SOCl_2$ (14.5 mL, 200 mmol, 10 equiv.) was slowly added in one portion and the reaction mixture was refluxed for 3 h. After cooling to room temperature, the mixture was treated with ice (100 g) under vigorous stirring, the organic fraction was extracted with pentane (100 mL, 3×), then the combined organic layers were washed with brine, dried over magnesium sulphate and concentrate under reduced pressure to provide a crude oil. To a solution of the former oil in THF (67 mL, 0.3 M) *N*-methylhydroxylamine hydrochloride (2.00 g, 24 mmol, 1.2 equiv.) and $NaHCO_3$ (3.36 g, 40 mmol, 2.0 equiv.) were loaded and the reaction was stirred at room temperature for 16 h. After this time, the solvent was evaporated, then the crude was diluted with EtOAc (250 mL), the organic phase was washed with $NaHCO_3$ (5×), brine, dried over magnesium sulphate and concentrate under reduced pressure to provide a crude oil. To a solution of the former crude oil in THF (100 mL), NaH (60%, 1.01 g, 30 mmol, 1.5 equiv.) was added portionwise at 0 °C. After the addition, the reaction was continued stirring for additional 30 min at 0 °C, then 1-fluoro-2,4-dinitrobenzene was slowly added in one portion and the reaction mixture

was stirred for 16 h at room temperature. Upon completion, the mixture was diluted with H_2O (50 mL) and the organic solvent was removed under reduced pressure. Then, the crude was diluted with EtOAc (250 mL), the organic phase was washed with Na_2CO_3 (5×), brine, dried over magnesium sulphate and concentrate under reduced pressure to provide a crude oil, which was dissolved in the minim amount of CH_2Cl_2 and treated with MeOH/H_2O (90:10, 150 mL) affording the pure desired compound as an off-white solid in a 9:1 ratio of *endo*/*exo* isomers.

***N*-(2,4-dinitrophenoxy)-*N*-methylbicyclo[2.2.1]hept-5-ene-2-carboxamide (28)**

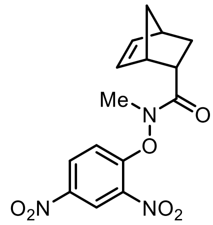

Following **GP1** with 5-norbornene-2-carboxylic acid (2.76 g, 20 mmol, mixture of *endo* and *exo*, predominantly *endo*), **28** was obtained in 65% yield. The pure *endo* isomer was obtained by purification by column chromatography on silica gel eluting with [petrol:CH_2Cl_2:EtOAc (65:30:5)]. ^1H NMR (500 MHz, $CDCl_3$) δ 8.92 (1H, d, J = 2.7 Hz), 8.52 (1H, dd, J = 9.3, 2.7 Hz), 7.57 (1H, d, J = 9.3 Hz), 6.21 (1H, dd, J = 5.7, 3.1 Hz), 5.97 (1H, dd, J = 5.7, 2.8 Hz), 3.32 (3H, s), 3.18 (1H, dt, J = 9.1, 3.9 Hz), 3.12 (1H, br s), 2.89 (1H, br s), 1.84 (1H, ddd, J = 11.4, 9.2, 3.7), 1.47–1.33 (2H, m), 1.26 (1H, d, J = 8.1 Hz); ^{13}C NMR (126 MHz, $CDCl_3$) δ 156.5, 142.3, 138.6, 137.9, 135.8, 132.1, 130.0, 122.8, 115.3, 49.9, 45.7, 42.7, 42.6, 36.7, 30.2. Data in accordance with the literature.[8]

Synthesis of 52 and 78. General Procedure 2 – GP2

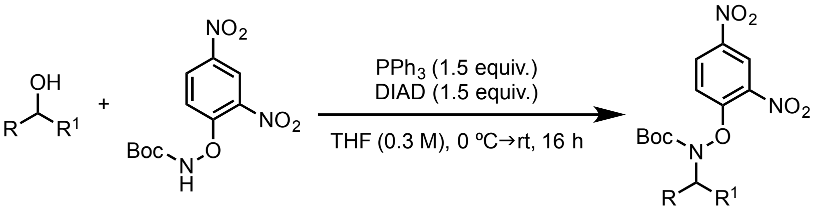

A solution of the appropriate alcohol (10 mmol), *tert*-butyl 2,4-dinitrophenoxycarbamate[3] 3.59 g, 12 mmol, 1.2 equiv.) and PPh_3 (3.93 g, 15 mmol, 1.5 equiv.) in THF (30 mL) was cooled to 0 ºC, then diisopropyl azodicarboxylate (2.95 mL, 15 mmol, 1.5 equiv.) was added dropwise over 15 min. After the addition, the mixture was stirred at room temperature for 16

h and then evaporated. The residue was purified by column chromatography to give the desired product.

***tert*-Butyl (2,4-dinitrophenoxy)(pent-4-en-1-yl)carbamate (52)**

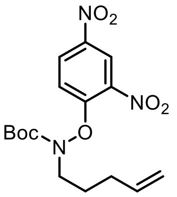

Following **GP2**, 4-pente-1-ol (10 mmol, 1.03 mL) gave **47** (3.340 g, 91%) as an oil. FT-IR ν_{max} (film)/cm^{-1} 2978, 2836, 1727, 1641, 1604, 1532, 1474, 1394, 1368, 1342, 1281, 1260, 1146, 1066; ^{1}H NMR (500 MHz, $CDCl_3$) δ 8.83 (1H, d, *J* = 2.7 Hz), 8.42 (1H, dd, *J* = 9.3, 2.7 Hz), 7.57 (1H, d, *J* = 9.3 Hz), 5.78 (1H, ddt, *J* = 16.9, 10.2, 6.6 Hz), 5.09–4.95 (2H, m), 3.77–3.59 (2H, m), 2.12 (2H, q, *J* = 7.2 Hz), 1.80 (2H, p, *J* = 7.4 Hz), 1.43 (9H, s); ^{13}C NMR (126 MHz, $CDCl_3$) δ 158.1, 156.1, 141.6, 137.1, 136.7, 129.3, 122.2, 116.3, 115.9, 84.3, 51.6, 30.9, 28.1, 25.8; HRMS (ESI) Found MNa^+ 390.1263 $C_{16}H_{21}N_3O_7Na$ requires 390.1272.

***tert*-Butyl (2,4-dinitrophenoxy)(non-1-en-5-yl)carbamate (78)**

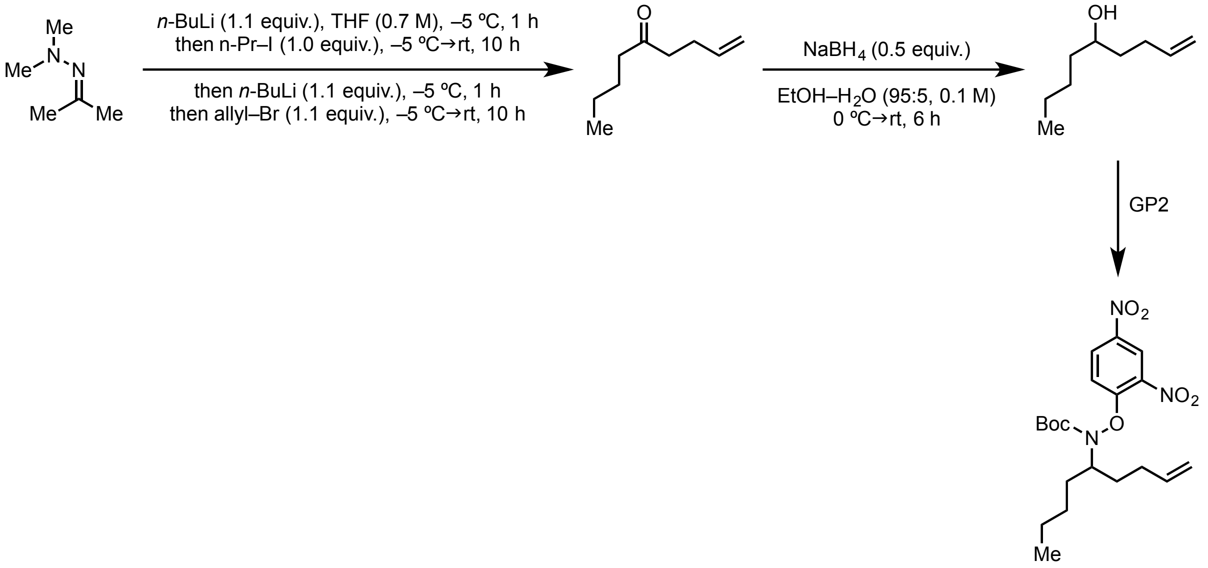

To a solution of acetone dimethylhydrazone (5,00 g, 50 mmol) in degassed and anhydrous THF (75 mL, 0.67 M) *n*-BuLi (1.6 M in hexane, 34 mL, 55 mmol, 1.1 equiv.) was added dropwise over 5 min at -5 °C. After 1 h, *n*-propyl iodide (4.88 mL, 50 mmol, 1.0 equiv.) was added in one portion and the mixture was stirred at room temperature for 10 h. After this time, the reaction mixture was cooled to -5 °C again and *n*-BuLi (1.6 M in hexane, 34 mL, 55 mmol, 1.1 equiv.) was added dropwise over 5 min. After 1 h, allyl bromide (4.32 mL, 50

mmol, 1.0 equiv.) was added in one portion and the mixture was stirred at room temperature for 10 h. Upon completion, the reaction mixture was poured in a 1L conical flask containing ice (350 g) and HCl (36%, 30 mL) under vigorous stirring for 15 min. Then, pentane (200 mL, 5×) was added and the organic material was extracted (5×). The combined organic layers were washed with brine, dried over magnesium sulphate and concentrate *in vacuo* to give non-1-en-5-one as a light brown oil (6.17 g), which was subsequently used without further purification. ^{1}H NMR (500 MHz, $CDCl_3$) δ 5.76 (1H, ddt, *J* = 16.8, 10.2, 6.5 Hz), 4.98 (1H, dq, *J* = 17.1, 1.7 Hz), 4.93 (1H, dq, *J* = 10.2, 1.4 Hz), 2.46 (2H, t, *J* = 7.4 Hz), 2.37 (2H, t, *J* = 7.5 Hz), 2.28 (2H, dtt, *J* = 7.7, 6.3, 1.3 Hz), 1.52 (2H, p, *J* = 7.4 Hz), 1.27 (2H, dq, *J* = 14.6, 7.3 Hz), 0.86 (3H, t, *J* = 7.3 Hz); ^{13}C NMR (126 MHz, $CDCl_3$) δ 210.5, 137.3, 115.2, 42.7, 41.8, 27.9, 26.0, 22.4, 13.9. Data in accordance with the literature.[9]

To a stirred solution of non-1-en-5-one (4.21 g, 30 mmol) in EtOH (270 mL), a solution of $NaBH_4$ (567.5 mg, 15 mmol, 0.5 equiv.) in H_2O (30 mL) was added dropwise over 15 min at 0 °C and the mixture was stirred at room temperature for additional 6 h. After this time, the reaction was gently treated with acetone (50 mL), diluted with H_2O (100 mL), acidified to pH 4 with HCl (6%) and then the organic solvents were concentrated *in vacuo*. Et_2O (100 mL, 3×) was added, the organic material extracted (3×), the combined organic layers were washed with brine, dried over magnesium sulphate and concentrate under reduced pressure to provide a crude oil, which was purified by column chromatography [petrol:EtOAc (80:20)] affording non-1-en-5-ol as colourless oil (56%, 3.16 g). ^{1}H NMR (500 MHz, $CDCl_3$) δ 5.84 (1H, ddt, *J* = 16.9, 10.1, 6.6 Hz), 5.04 (1H, dd, *J* = 17.2, 1.9 Hz), 4.96 (1H, dt, *J* = 10.2, 1.5 Hz), 3.61 (1H, tt, *J* = 8.5, 4.6 Hz, 1H), 2.34 – 2.00 (2H, m), 1.64 – 1.23 (8H, m), 0.90 (3H, t, *J* = 7.0 Hz); ^{13}C NMR (126 MHz, $CDCl_3$) δ 138.8, 114.8, 71.6, 37.3, 36.6, 30.2, 27.9, 22.9, 14.2. Data in accordance with the literature.[10]

Following **GP2** with 4-pente-1-ol (10 mmol, 1.42 g) gave **73** (56%) as a pale yellow oil. FT-IR ν_{max} (film)/cm^{-1} 2932, 2862, 2360, 1726, 1606, 1538, 1475, 1394, 1343, 1259, 1157, 1065; ^{1}H NMR (500 MHz, $CDCl_3$) δ 8.84 (1H, d, *J* = 2.7 Hz), 8.42 (1H, dd, *J* = 9.4, 2.7 Hz), 7.50 (1H, d, *J* = 9.3 Hz), 5.77 (1H, ddt, *J* = 16.9, 10.1, 6.6 Hz), 5.15–4.77 (2H, m), 4.16 (1H, tt, *J* = 8.6, 5.4 Hz), 2.12 (2H, q, *J* = 7.4 Hz), 1.78–1.51 (4H, m), 1.44 (9H, s), 1.39–1.17 (4H, m), 0.88 (3H, t, *J* = 6.7 Hz); ^{13}C NMR (126 MHz, $CDCl_3$) δ 158.5, 156.5, 141.4, 137.5, 136.4, 129.1, 122.2, 116.3, 115.6, 84.1, 62.1, 31.9, 31.6, 30.8, 28.7, 28.1, 22.6, 14.0; HRMS (ESI) Found MNa^+ 446.1898 $C_{20}H_{29}N_3O_7Na$ requires 446.1890.

3.2 Preparation of Organozinc Reagents

Stock solutions of the following organozincs have been prepared according to the procedures reported in the literature, from the corresponding commercially available Grignard reagents [11]. Tridecylmagnesium bromide has been prepared from the corresponding 1-bromotridecane following a reported procedure.[12] All the solutions of Grignard reagents and the organolithium compounds used for the synthesis, were titrated before use, according to the procedure reported in the literature.[13]

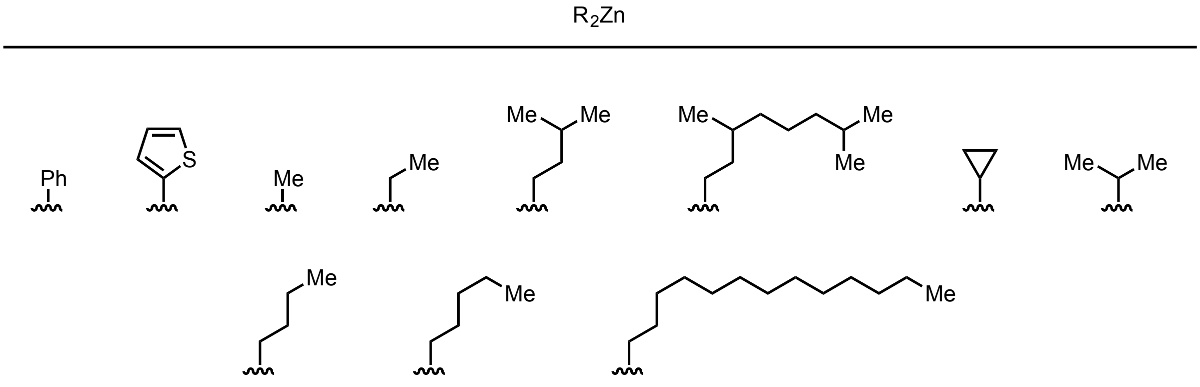

4 Umpolung *N*-Arylations and Cyclization–Cascades

4.1 Reactions Optimization

Table S1.

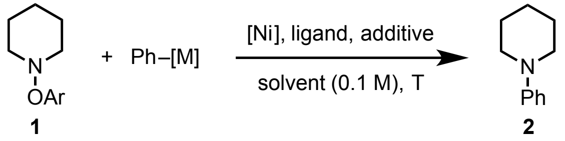

Entry	[M] (equiv.)	[Ni] (mol%)	Ligand (mol%)	Additive (eq.)	Solvent (M)	T (ºC)	Yield (%)
**1**	ZnPh (1.5)	$NiCl_2$glyme (20)	–	–	DMF–THF (1.1.) (0.1)	rt	–
**2**	ZnPh (1.5)	$NiCl_2$glyme (20)	dtbpy (20)	–	DMF–THF (1.1.) (0.1)	rt	90
**3**	ZnPh (1.5)	$NiCl_2$glyme (10)	dtbpy (10)	–	DMF–THF (1.1.) (0.1)	rt	74
**4**	ZnPh (1.5)	$NiCl_2$glyme (5)	dtbpy (5)	–	DMF–THF (1.1.) (0.1)	rt	69
**5**	ZnPh (1.5)	$NiCl_2$•6H_2O (5)	dtbpy (5)	–	DMF–THF (1.1.) (0.1)	rt	99
**6**	ZnPh (1.5)	$NiCl_2$•6H_2O (5)	bpy (5)	–	DMF–THF (1.1.) (0.1)	rt	84
**7**	ZnPh (1.5)	$NiCl_2$•6H_2O (5)	dMeObpy (5)	–	DMF–THF	rt	79

					(1.1.) (0.1)		
**8**	ZnPh (1.5)	$NiCl_2$•$6H_2O$ (5)	batophen (5)	–	DMF–THF (1.1.) (0.1)	rt	71
**9**	ZnPh (1.5)	$NiCl_2$•$6H_2O$ (2)	dtbpy (4)	–	DMF–THF (1.1.) (0.1)	rt	81
**10**	ZnPh (1.5)	$NiCl_2$•$6H_2O$ (1)	dtbpy (2)	–	DMF–THF (1.1.) (0.1)	rt	61
**11**	ZnCl (1.5)	$NiCl_2$•$6H_2O$ (5)	dtbpy (10)	–	DMF–THF (1.1.) (0.1)	rt	85
**12**	ZnPh (1.5)	(dtbpy)$NiCl_2$ (5)	–	–	DMF–THF (1.1.) (0.1)	rt	92
**13**	$B(OH)_2$ (2.0)	$NiCl_2$glyme (20)	dtbpy (20)	–	1,4-dioxane–DMF (9:1) (0.05 M)	rt	–
**14**	$B(OH)_2$ (2.0)	$NiCl_2$glyme (20)	dtbpy (20)	–	1,4-dioxane–DMF (9:1) (0.05 M)	75	–
**15**	$B(OH)_2$ (2.0)	$NiCl_2$glyme (20)	dtbpy (20)	Et_3N (3.5)	1,4-dioxane–DMF (9:1) (0.05 M)	75	traces
**16**	$B(OH)_2$ (2.0)	$NiCl_2$glyme (20)	dtbpy (20)	Et_3N (3.5)	THF–DMF (1:1) (0.05 M)	75	–
**17**	$B(OH)_2$ (2.0)	$NiCl_2$glyme (20)	dtbpy (20)	Et_3N (3.5)	MTBE–DMF (9:1) (0.05 M)	75	75

**18**	$B(OH)_2$ (2.0)	$NiCl_2$glyme (20)	dtbpy (20)	Et_3N (2.0)	toluene–DMF (9:1) (0.05 M)	75	31
**19**	$B(OH)_2$ (2.0)	$NiCl_2$glyme (20)	dtbpy (20)	Cs_2CO_3 (2.0)	1,4-dioxane–DMF (9:1) (0.05 M)	75	85
**20**	$B(OH)_2$ (2.0)	$NiCl_2$glyme (5)	dtbpy (5)	Cs_2CO_3 (2.0)	1,4-dioxane–DMF (9:1) (0.05 M)	75	62
**21**	$B(OH)_2$ (2.0)	$NiCl_2$glyme (5)	dtbpy (5)	Cs_2CO_3 (2.0)	MTBE (0.05 M)	75	90
**22**	$B(OH)_2$ (2.0)	$NiCl_2$glyme (20)	dtbpy (20)	K_2CO_3 (2.0)	1,4-dioxane–DMF (9:1) (0.05 M)	75	17
**23**	$B(OH)_2$ (2.0)	$NiCl_2$glyme (20)	dtbpy (20)	CsF	1,4-dioxane–DMF (9:1) (0.05 M)	75	13
**24**	$B(OH)_2$ (2.0)	$NiCl_2 \cdot 6H_2O$ (20)	dtbpy (20)	Cs_2CO_3 (3.5)	1,4-dioxane–DMF (9:1) (0.05 M)	75	50
**25**	$B(OH)_2$ (2.0)	–	–	–	toluene	50	–
**26**	$Si(OEt)_3$ (2.0)	$NiCl_2$glyme (20)	dtbpy (20)	–	1,4-dioxane–DMF (9:1) (0.05 M)	75	–
**27**	$Si(OEt)_3$ (2.0)	$NiCl_2$glyme (20)	dtbpy (20)	TBAF (2.0)	1,4-dioxane–DMF (9:1) (0.05 M)	75	–
**28**	$Si(OEt)_3$ (2.0)	$NiCl_2$glyme (20)	dtbpy (20)	CsF (2.0)	1,4-dioxane–DMF (9:1) (0.05 M)	75	–
**29**	$Si(OEt)_3$ (2.0)	$NiCl_2$glyme (20)	dtbpy (20)	TBAT (2.0)	1,4-dioxane–DMF	75	20

					(9:1) (0.05 M)		
**30**	$Si(OEt)_3$ (2.0)	$NiCl_2$glyme (20)	dtbpy (20)	TBAT (2.0)	DMSO–DMF (9:1) (0.05 M)	75	48
**31**	$Si(OEt)_3$ (2.0)	$NiCl_2$glyme (20)	dtbpy (20)	TBAT (2.0)	DMF (0.05 M)	75	32
**32**	$Si(OEt)_3$ (2.0)	–	–	TBAT (2.0)	DMSO–DMF (9:1) (0.05 M)	75	–
**33**	$Si(OEt)_3$ (2.0)	$NiCl_2$glyme (20)	dtbpy (20)	TBAT (2.0)	DMSO (0.05 M)	75	68
**34**	$Si(OEt)_3$ (2.0)	$NiCl_2$glyme (20)	dtbpy (20)	TBAT (2.0)	DMSO (0.05 M)	50	88
**35**	$Si(OEt)_3$ (2.0)	$NiCl_2$glyme (5)	dtbpy (5)	TBAT (2.0)	DMSO (0.05 M)	50	86
**36**	$SiMe_3$	$NiCl_2$glyme (20)	dtbpy (20)	TBAT (2.0)	DMSO (0.05 M)	50	–
**37**	SiMeCB	$NiCl_2$glyme (20)	dtbpy (20)	TBAT (2.0)	DMSO (0.05 M)	50	–

Table S2.

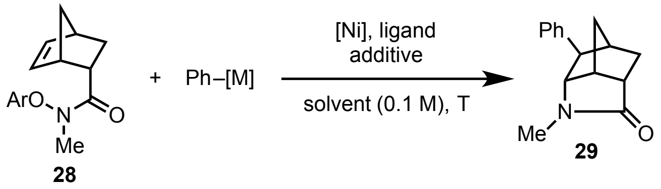

Entry	[M] (equiv.)	[Ni] (mol%)	Ligand (mol%)	Additive (eq.)	Solvent (M)	T (ºC)	Yield (%)
**1**	ZnPh (1.5)	$NiCl_2$•$6H_2O$ (20)	dtbpy (20)	–	THF–DMF (1:1) (0.1)	rt	61
**2**	ZnPh (1.5)	$NiBr_2$•$6H_2O$ (20)	dtbpy (20)	–	THF–DMF (1:1) (0.1)	rt	71
**3**	ZnPh (1.5)	(dtbpy)$NiBr_2$ (20)	–	–	THF–DMF (1:1) (0.1)	rt	76
**4**	ZnPh (1.5)	(dtbpy)$NiBr_2$ (5)	–	–	THF–DMF (1:1) (0.1)	rt	52
**5**	$B(OH)_2$ (2.0)	(dtbpy)$NiBr_2$ (20)	–	Cs_2CO_3 (2.0)	THF–DMF (1:1) (0.1)	75	47
**6**	$B(OH)_2$ (2.0)	(dtbpy)$NiBr_2$ (20)	–	Cs_2CO_3 (2.0)	MTBE (0.05 M)	75	83
**7**	$Si(OEt)_3$ (2.0)	(dtbpy)$NiBr_2$ (20)	–	TBAT (2.0)	DMSO (0.05 M)	50	51

Table S3.

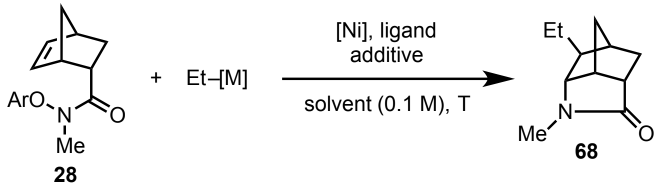

Entry	[M] (equiv.)	[Ni] (mol%)	Ligand (mol%)	Additive (eq.)	Solvent (M)	T (ºC)	Yield (%)
**1**	ZnEt (1.5)	$NiCl_2$•$6H_2O$ (20)	dtbpy (20)	–	THF–DMF (1:1) (0.1)	rt	34
**2**	ZnEt (1.5)	$NiBr_2$•$6H_2O$ (20)	dtbpy (20)	–	THF–DMF (1:1) (0.1)	rt	39
**3**	ZnEt (1.5)	(dtbpy)$NiBr_2$ (20)	–	–	THF–DMF (1:1) (0.1)	rt	60
**4**	ZnEt (1.5)	NiI_2 (20)	dtbpy (20)	–	THF–DMF (1:1) (0.1)	rt	33
**5**	ZnEt (1.5)	Ni(acac)$_2$•$4H_2O$ (20)	dtbpy (20)	–	THF–DMF (1:1) (0.1)	rt	32
**6**	ZnEt (1.5)	$NiBr_2$•$6H_2O$ (20)	dtbpy (20)	–	THF–DMF (1:1) (0.1)	rt	39
**7**	B(OH)$_2$ (2.0)	$NiCl_2$glyme (5)	dtbpy (5)	Cs_2CO_3 (2.0)	MTBE (0.05 M)	75	–
**8**	B(OH)$_2$ (2.0)	(dtbpy)$NiBr_2$ (20)	–	Cs_2CO_3 (2.0)	THF–DMF (1:1) (0.1)	rt	–

**9**	$B(OH)_2$ (2.0)	(dtbpy)$NiBr_2$ (20)	–	Cs_2CO_3 (2.0)	MTBE (0.05 M)	rt	–

4.2 GP3 – General Procedure for *N*-arylation and cyclization-arylation with organozincs

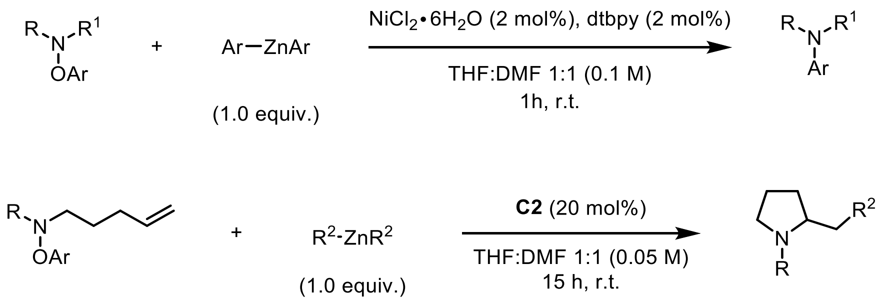

- *N-Arylation:* An oven-dried 10 mL crimp-cap microwave vial equipped with a stirring bar was charged with the aryloxyamine (0.1 mmol, 1.0 equiv.), sealed, evacuated and refilled with N_2 for three times. Then a solution of $NiCl_2$·$6H_2O$:dtbpy 1:1 in DMF (0.2 µmol, 2 mol%) and a solution of organozinc in THF (0.1 mmol, 1.0 equiv.) were added (DMF:THF 1:1, 0.1 M). The reaction was stirred at room temperature for 1h, the crude was then absorbed on silica and purified on silica gel column.
- *Cyclization-arylation–alkylation:* An oven-dried 10 mL crimp-cap microwave vial equipped with a stirring bar was charged with the aryloxyamide (0.1 mmol, 1.0 equiv.), and the catalyst **C2** (2 µmol, 20 mol%), sealed, evacuated and refilled with N_2 for three times. Then DMF and a solution of organozinc in THF (0.1 mmol, 1.0 equiv.) were added (DMF:THF 1:1, 0.025M). The reaction was stirred at room temperature for 15 h the crude was absorbed on silica and purified on silica gel column.

4.3 GP4 – General Procedure for *N*-arylation and cyclization-arylation with aryl boronic acids

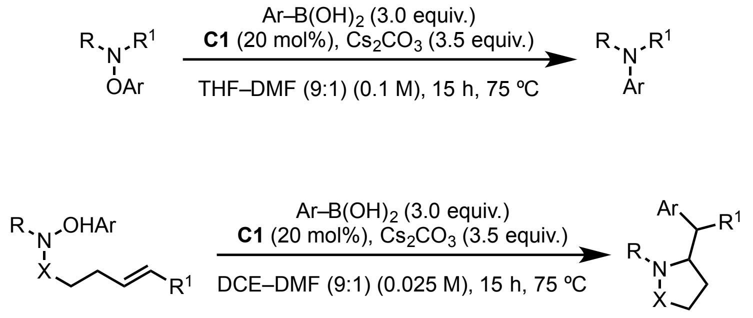

Unless otherwise stated, an oven-dried 10 mL crimp-cap microwave vial equipped with a stirring bar was charged with nickel(II)-catalyst **C1** (2 µmol, 20 mol%), the appropriate aryloxyamine/amide (0.1 mmol, 1.0 equiv.), the appropriate arylboronic acid (0.3 mmol, 3.0 equiv.) and Cs_2CO_3 (0.35 equiv.). The vial was sealed, evacuated and refilled with N_2 (3×),

and the following anhydrous solvents were added. The reaction was stirred at the stated temperature for 15 h.

- *N-Arylation*: THF:DMF (9:1, 0.1 M), 75 °C.
- *Cyclization-arylation/1,5-HAT-arylation*: DCE:DMF (9:1, 0.025 M), 95 °C.

Upon completion, the crude was diluted with water and extracted with EtOAc (3×) and then purified by column chromatography.

4.4 GP5 – General Procedure for *N*-arylation and cyclization-arylation with organo silanes

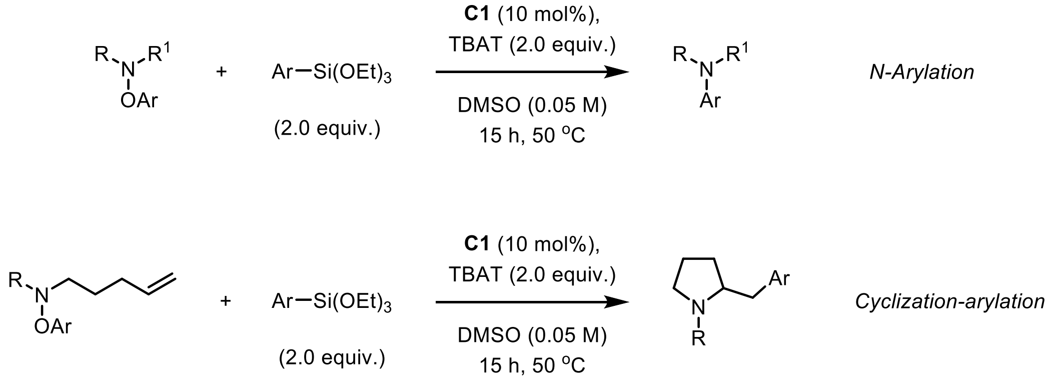

A dry tube equipped with a stirring bar was charged with the aryloxyamine/amide (0.1 mmol, 1.0 equiv.), sealed, evacuated and refilled with N_2 for three times. Then a stock solution of $NiCl_2{\cdot}6H_2O$:dtbpy 1:1 in DMF were added (1.0 µmol, 10 mol%), followed by the addition of a stock solution of the arylsilane and TBAT in DMSO (0.2 mmol, 2.0 equiv.). The reaction was stirred at 50 °C for 15h, the crude was then absorbed on silica and purified on silica gel column.

5 Analysis of Chan-Lam Couplings with Heteroaromatic Organometallics

As the use of arylboronic acids and, to a lesser extent, arylsilanes and arylzincs in this methodology would deliver products identical to the widely used Chan-Lam coupling, we performed a literature survey to identify potential areas of complementarity (Figure S1). We realized that amination of C-3 and C-4-zincated pyridines and C-2-zincated 5-membered ring heterocyles (e.g. thiophene) has not been reported. In the case of aryl boronic acids no precedent was found to access C-4 aminated pyridines. Aryl silanes are considerably less used in aromatic amination.

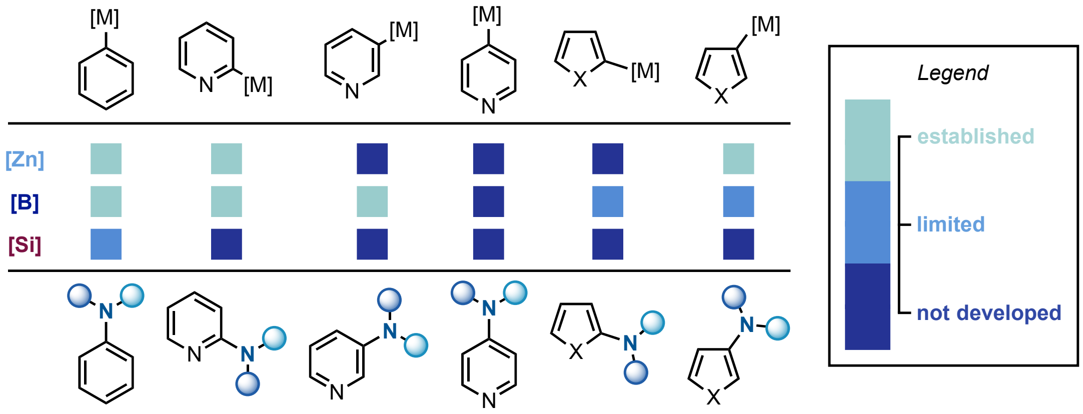

Figure S1.

6 Mechanistic Considerations

The following experiments using substrates **3–5** were performed following the general procedures **GP3**, **GP4** or **GP5**. As shown in Table S4, although the *N*-arylation product **6** was obtained in several cases, product **7**, which would result from a radical 5-*exo*-*trig* cyclisation, was exclusively obtained when using **3**, regardless from the nature of the organometallic partner (entries 1–6). The presence of both **6** and **7** in these reactions suggests that the rates of cyclisation (1.9 10^5 s^{-1}) and *N*-arylation are comparable.[1]

Table S4.

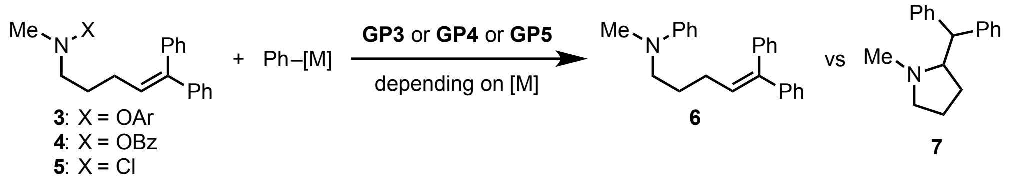

Entry	X	[M]	6 (%)	7 (%)
**1**	OAr	ZnPh	30	22
**2**	OBz	ZnPh	13	–
**3**	Cl	ZnPh	31	–
**4**	OAr	$B(OH)_2$	8	9
**5**	OBz	$B(OH)_2$	traces	–
**6**	Cl	$B(OH)_2$	–	–

Indeed, the ratio between **6** and **7** varies in favor of **7** at low Ni-catalyst loading (Table S5, entries 1–4) and also at higher dilution (entries 5–8), which further supports the intermediacy of a N-radical in our process.

[1] The C-radical formed upon *N*-radical cyclization could intercept the Ni(II)-aryl complex. However, as this radical is di-benzylic we believe other pathways might be operative (e.g. 1,5-HAT, oxidation…). Furthermore, the addition of benzylic radicals to Ni-complexes has been described to be reversible, see: O. Gutierrez, J. C. Tellis, D. N. Primer, G. A. Molander, M. C. Kozlowski, *J. Am. Chem. Soc.* **2015**, *137*, 4896.

Table S5.

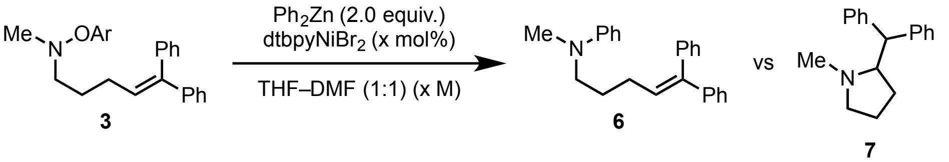

Entry	[ ] (M)	[Ni] (mol%)	6 (%)	7 (%)
**1**	0.08	20	45	9
**2**	0.05	20	34	22
**3**	0.03	20	25	29
**4**	0.01	20	19	38
**5**	0.05	10	18	22
**6**	0.05	5	9	22
**7**	0.05	2	4	23
**8**	0.05	1	traces	29

We subjected precursors **3** and **4** to the optimum conditions identified in Table S5 favouring cyclization over *N*-arylation (Table S5, entry 8). As shown in Table S6 precursor **3** only led to the cyclized product **7** (entry 1) while **6** underwent *N*-arylation in poor yield. (entry 2).

Table S6.

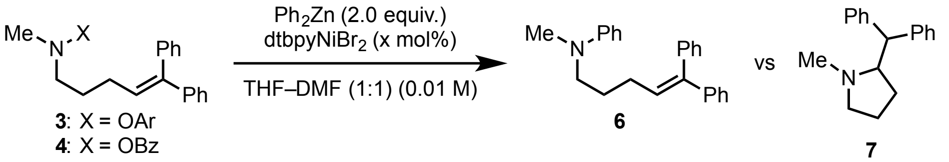

Entry	X	6 (%)	7 (%)
**1**	OAr	–	29
**2**	OBz	10	–

Using precursor **3** product **7** was detected in 29% yield and no **6** was found. Using precursor **2** no cyclization was found and only 10% of *N*-arylated product **6** was found.

7 Products Characterization

1-Phenylpiperidine (2)

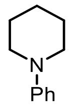

S1 (27 mg, 0.1 mmol) gave **2** following **GP3** (99%), **GP4** (85%) and **GP5** (86%). ^{1}H NMR (400 MHz, $CDCl_3$) δ 7.28–7.19 (2H, m), 6.93 (2H, d, *J* = 8.2 Hz), 6.81 (1H, t, *J* = 7.3 Hz), 3.17–3.10 (4H, m), 1.70 (4H, p, *J* = 5.7 Hz), 1.56 (2H, dtd, *J* = 9.0, 5.3, 4.8, 2.2 Hz); ^{13}C NMR (125 MHz, $CDCl_3$) δ 152.5, 129.2, 119.4, 116.8, 50.9, 26.1, 24.5. Data in accordance with the literature.[14]

N-(5,5-diphenylpent-4-en-1-yl)-*N*-methylaniline (6)

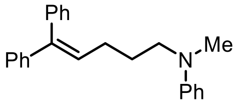

3 gave **6** following **GP3** (30%) and **GP4** (8%) as an oil. FT-IR ν_{max} (film)/cm^{-1} 2961, 2788, 1597, 1491, 1448, 1377, 1313, 1258, 1173, 1082, 1031; ^{1}H NMR (400 MHz, $CDCl_3$) δ 7.37 (2H, t, *J* = 7.3 Hz), 7.31 (1H, t, *J* = 7.2 Hz), 7.28 – 7.13 (10H, m), 6.75 – 6.60 (3H, m), 6.09 (1H, t, *J* = 7.4 Hz), 3.35 – 3.20 (2H, m), 2.87 (3H, s), 2.15 (2H, q, *J* = 7.5 Hz), 1.72 (2H, p, *J* = 7.5 Hz); ^{13}C NMR (126 MHz, $CDCl_3$) δ 149.3, 142.8, 142.3, 140.2, 130.0, 129.3, 129.2, 128.3, 128.2, 127.4, 127.1, 127.0, 116.1, 112.3, 52.5, 38.5, 27.5, 27.0; HRMS (ASAP): Found MH^{+} 328.2062 $C_{24}H_{26}N$ requires 328.2060.

2-Benzhydryl-1-methylpyrrolidine (7)

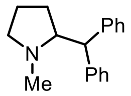

Following **GP3**, **3** gave **7** (22%) as an oil. FT-IR ν_{max} (film)/cm^{-1} 2917, 2361, 1598, 1574, 1504, 1442, 1361, 1259, 1192, 1116, 1073, 1031; ^{1}H NMR (400 MHz, $CDCl_3$) δ 7.64 (2H, d, *J* = 8.2 Hz), 7.53 (2H, d, *J* = 8.2 Hz), 7.27 (4H, t, *J* = 7.7 Hz), 7.14 (2H, t, *J* = 7.2 Hz), 3.68 (1H, dd, *J* = 9.6, 4.3 Hz), 3.17 (1H, t, *J* = 6.3 Hz), 2.48 (1H, q, *J* = 9.5 Hz), 1.87 (3H, s), 1.99 – 1.88 (1H, m) 1.79 – 1.57 (3H, m), 1.35 – 1.19 (1H, m); ^{13}C NMR (126 MHz, $CDCl_3$) δ 146.7, 128.3 & 128.2, 126.4 & 126.3, 125.6 & 125.5, 72.4, 59.3, 43.2, 29.9, 24.1, 1.2.

4,4-Difluoro-1-phenylpiperidine (8)

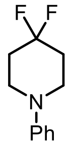

Following **GP3**, **S2** (37 mg, 0.1 mmol) gave **8** (73%) as an oil. FT-IR ν_{max} (film)/cm^{-1} 3617, 2962, 1634, 1257, 1050, 997 cm^{-1}; ^{1}H NMR (500 MHz, $CDCl_3$) δ 7.28 (2H, t, *J* = 7.9 Hz), 6.95 (2H, d, *J* = 8.1 Hz), 6.89 (1H, t, *J* = 7.3 Hz), 3.66–3.13 (4H, m), 2.10 (4H, tt, *J* = 13.3, 5.6 Hz); ^{13}C NMR (126 MHz, $CDCl_3$) δ 129.4, 124.0, 122.1 (t, *J* = 241.7 Hz), 120.3, 117.0, 46.9 (t, *J* = 5.2 Hz), 33.8 (t, *J* = 22.8 Hz); ^{19}F NMR ($CDCl_3$, 376 MHz) δ -97.6; HRMS (HESI): Found MH^+ 198.1089 $C_{11}H_{14}NF_2$ requires 198.1089.

4-Phenylmorpholine (9)

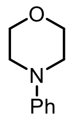

Following **GP3** for 3h, **S10** (27 mg, 0.1 mmol) gave **9** (95%). ^{1}H NMR (500 MHz, $CDCl_3$) δ 7.31–7.25 (2 H, m), 6.95–6.83 (3H, m), 3.93–3.82 (4H, m), 3.23–3.10 (4H, m); ^{13}C NMR (101 MHz, $CDCl_3$) δ 151.5, 129.4, 120.3, 115.9, 67.2, 49.6. Data in accordance with the literature.[14]

4-Phenylthiomorpholine (10)

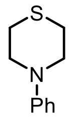

Following **GP3** with 5 mol% of **C1** for 3h, **S3** (0.1 mmol) gave **10** (85%). ^{1}H NMR (500 MHz, $CDCl_3$) δ 7.33–7.21 (2H, m), 7.03–6.81 (3H, m), 3.57–3.49 (4H, m), 2.76–2.70 (4H, m); ^{13}C NMR (101 MHz, $CDCl_3$) δ 151.5, 129.4, 120.0, 117.3, 52.3, 27.0. Data in accordance with the literature.[15]

Benzyl 4-phenylpiperazine-1-carboxylate (11)

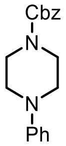

Following **GP3** for 3h, **S4** (40 mg, 0.1 mmol) gave **11** (95%). ^{1}H NMR (500 MHz, $CDCl_3$) δ 7.43–7.32 (2H, m), 6.98–6.86 (3H, m), 3.71–3.63 (4H, m), 3.15 (4H, br s); ^{13}C NMR (101 MHz, $CDCl_3$) δ 155.0, 151.0, 136.4, 129.0, 128.4, 127.9, 127.8, 120.3, 116.6, 67.2, 49.5, 43.8. Data in accordance with the literature.[16]

Furan-2-yl(4-phenylpiperazin-1-yl)methanone (12)

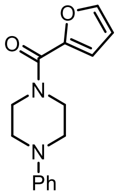

Following **GP3**, **S5** (36 mg, 0.1 mmol) gave **12** (83%). ^{1}H NMR (500 MHz, $CDCl_3$) δ 7.50 (1H, s), 7.29 (2H, t, *J* = 7.8 Hz), 7.05 (1H, d, *J* = 2.8 Hz), 6.95 (2H, d, *J* = 8.0 Hz), 6.91 (1H, t, *J* = 7.3 Hz), 6.55–6.46 (1H, m), 3.97 (4H, s), 3.32–3.21 (4H, m); ^{13}C NMR (101 MHz, $CDCl_3$) δ 159.2, 151.0, 148.0, 143.9, 129.4, 120.6, 116.7, 116.7, 111.5, 49.8. Data in accordance with the literature.[17]

***N*,*N*-Diethylaniline (13)**

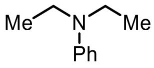

Following **GP3**, **S6** (25 mg, 0.1 mmol) gave **13** (88%). ^{1}H NMR (400 MHz, $CDCl_3$) δ 7.19 (2H, t, *J* = 7.9 Hz), 6.67 (2H, d, *J* = 8.4 Hz), 6.62 (1H, t, *J* = 7.2 Hz), 3.34 (4H, q, *J* = 7.0 Hz), 1.15 (6H, t, *J* = 7.1 Hz); ^{13}C NMR (400 MHz, $CDCl_3$) δ 146.8, 128.2, 114.3, 110.9, 43.3, 11.5. Data in accordance with the literature.[18]

***N*-Methyl-*N*-phenethylaniline (14)**

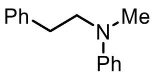

Following **GP3**, **S7** (32 mg, 0.1 mmol) gave **14** (95%). ^{1}H NMR (500 MHz, $CDCl_3$) δ 7.38–7.06 (7H, m), 6.87–6.62 (3H, m), 3.57 (2H, t, *J* = 7.8 Hz), 2.92–2.76 (5H, m); ^{13}C NMR (101

MHz, $CDCl_3$) δ 148.8, 139.8, 129.2, 128.8, 128.5, 126.2, 116.1, 112.1, 54.7, 38.4, 32.9. Data in accordance with the literature.[19]

***N,N*-Dibenzylaniline (15)**

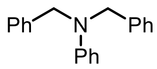

Following **GP3**, **S8** (38 mg, 0.1 mmol) gave **15** (88%). ^{1}H NMR (500 MHz, $CDCl_3$) δ 7.33 (4H, t, *J* = 7.7 Hz, 4H), 7.27–7.23 (6H, m), 7.17 (2H, t, *J* = 7.7 Hz), 6.75 (2H, d, *J* = 7.7 Hz), 6.70 (1H, t, *J* = 7.7 Hz), 4.66 (4H, s); ^{13}C NMR (126 MHz, $CDCl_3$) δ 149.3, 138.8, 129.4, 128.8, 127.0, 126.8, 116.9, 112.6, 54.3. Data in accordance with the literature.[20]

2-(Piperidin-1-yl)pyridine (16)

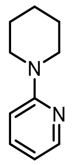

A solution of 2-bromopyridine (316 mg, 2.0 mmol, 1.0 equiv.) in THF (8.0 ml) was cooled at –78 ºC and *n*-BuLi (1,25 ml of a solution 1.6 M in hexane, 2.0 mmol, 1.0 equiv.) was added by dropwise over 20 min. The mixture was stirred at –78 ºC for 30 minutes and a solution of $ZnCl_2$ (1.0 ml of a solution 1.0 M in THF, 1.0 mmol, 0.5 equiv.) was added. The mixture was stirred for 30 minutes at –78 ºC, then at room temperature for 30 minutes. The solution was titrated as 0.1 M, and used for the reaction.
Following **GP3**, in DMF:THF 1:2 (0.06 M), and with 10 mol% of catalyst, **S1** (27 mg, 0.1 mmol) gave **16** (41%). ^{1}H NMR (500 MHz, $CDCl_3$) δ 8.16 (1H, dd, *J* = 5.2, 1.9 Hz), 7.42 (1H, ddd, *J* = 8.8, 7.1, 2.0 Hz), 6.62 (1H, d, *J* = 8.6 Hz), 6.54 (1H, dd, *J* = 7.1, 4.9 Hz), 3.51 (4H, br s), 1.63 (6H, s); ^{13}C NMR, (101 MHz, $CDCl_3$) δ 159.8, 148.0, 137.5, 112.5, 107.3, 46.5, 25.7, 24.9. Data in accordance with the literature.[21]

3-(Piperidin-1-yl)pyridine (17)

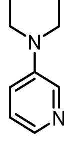

A dry, nitrogen charged round bottomed flask was charged with *i*PrMgCl·LiCl (1.0 ml of a titrated solution 1.0 M in THF, 1.0 mmol, 0.5 equiv.) and diluted with 1.0 ml of anhydrous

THF. The mixture was cooled at 0 ºC and 3-bromopyridine (316 mg, 2.0 mmol, 1.0 equiv.) was added in one portion. The solution was stirred at r.t. for 30 min before a solution of $ZnCl_2$ in THF (1.0 ml of a solution 1.0 M, 1.0 mmol, 0.5 equiv.) was added in. After 30 min stirring, the solution was titrated as 0.1 M.

Following **GP3** in DMF:THF 1:2 (0.06 M), and with 10 mol% of catalyst, **S1** (27 mg, 0.1 mmol) gave **17** (37%). ^{1}H NMR (500 MHz, $CDCl_3$) δ 8.31 (1H, d, *J* =2.7 Hz), 8.06 (1H, dd, *J* = 4.5, 1.3 Hz), 7.14–7.20 (2H, m), 3.19 (4H, t, *J* = 5.4 Hz), 1.71–1.75 (4H, m), 1.61–1.69 (2H, m); ^{13}C NMR (101 MHz, $CDCl_3$) δ 147.7, 140.0, 139.0, 123.3, 122.6, 49.9, 25.6, 24.1. Data in accordance with the literature.[22]

4-(Piperidin-1-yl)pyridine (18)

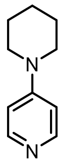

A dry flask was charged with 4-iodopyridine (102 mg, 0.5 mmol, 1.0 equiv.) and THF (2.0 ml) and was cooled at –78 ºC. *n*-BuLi (0,31 ml of a solution 1.6 M in hexane, 0.5 mmol, 1.0 equiv.) was then added by dropwise over 20 min. The mixture was stirred for 30 min at –78 ºC and a solution of $ZnCl_2$ (0.5 ml of a solution 1.0 M in THF, 0.5 mmol, 1 equiv.) was added. After stirring for 30 min at –78 ºC the solution was warmed up at room temperature, and used for the reaction.

Following **GP3** in DMF:THF 1:2 (0.06 M), and with 10 mol% of catalyst, **S1** (27 mg, 0.1 mmol) gave **18** (29%). ^{1}H NMR (500 MHz, $CDCl_3$) δ 8.25 (2H, br s), 6.74 (2H, br d, *J* = 5.80 Hz), 3.51–3.39 (4H, m), 1.77–1.56 (6H, m); ^{13}C NMR (101 MHz, $CDCl_3$) δ 155.6, 146.2, 107.5, 47.3, 25.2, 24.0. Data in accordance with the literature.[23]

2-Methoxy-4-(piperidin-1-yl)pyridine (19)

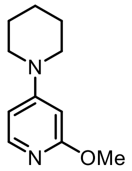

Following **GP4**, **S1** (27 mg, 0.1 mmol) gave **19** (49%). ^{1}H NMR (400 MHz, $CDCl_3$) δ 7.83 (1H, d, J = 6.1 Hz), 6.36 (1H, dd, *J* = 6.2, 2.4 Hz), 6.02 (1H, d, *J* = 2.3 Hz), 3.87 (3H, s), 3.27 (4H, t, *J* = 4.5 Hz), 1.67–1.53 (6H, m); ^{13}C NMR (101 MHz, $CDCl_3$) δ 165.9, 157.5, 146.8, 104.2, 92.6, 53.1, 47.5, 25.0, 24.3. Data in accordance with the literature.[24]

1-(Thiophen-2-yl)piperidine (20)

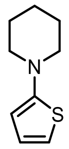

S1 (27 mg, 0.1 mmol) gave **20** (83%) following **GP3** with 10 mol% of catalyst and following **GP5** (29%). ^{1}H NMR (500 MHz, $CDCl_3$) δ 6.75 (1H, dd, *J* = 5.5, 3.7 Hz), 6.56 (1H, dd, *J* = 5.5, 1.3 Hz), 6.09 (1H, dd, *J* = 5.2, 1.3 Hz), 3.13–3.09 (4H, m), 1.72 (4H, p, *J* = 5.8 Hz), 1.62–1.52 (2H, m); ^{13}C NMR (101 MHz, $CDCl_3$) δ 160.4, 126.2, 111.8, 104.9, 53.0, 25.55, 23.94. Data in accordance with the literature.[25]

1-(Thiophen-3-yl)piperidine (21)

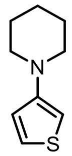

Following **GP4**, **S1** (27 mg, 0.1 mmol) gave **21** (38%). ^{1}H NMR (500 MHz, $CDCl_3$) δ 7.22 (1H, dd, *J* = 5.2, 3.0 Hz), 6.89 (1H, dd, *J* = 5.2, 1.7 Hz), 6.18 (1H, dd, *J* = 3.0, 1.7 Hz), 3.07 (4H, t, *J* = 7.0 Hz), 1.76–1.68 (4H, m),1.60–1.52 (2H, m); ^{13}C NMR, (101 MHz, $CDCl_3$) δ 153.5, 125.1, 120.7, 100.1, 51.9, 25.8, 24.3. Data in accordance with the literature.[26]

1-(*p*-Tolyl)piperidine (22)

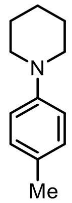

Following **GP5**, **S1** (27 mg, 0.1 mmol) gave **22** (71%). ^{1}H NMR (500 MHz, $CDCl_3$) δ 7.16 (2H, d, *J* = 8.4 Hz), 6.96 (2H, d, *J* = 8.4 Hz), 3.18 (4H, t, *J* = 5.4 Hz), 2.37 (3H, s), 1.85–1.77 (4H, m), 1.69–1.61 (2H, m); ^{13}C NMR (125 MHz, $CDCl_3$) δ 150.2, 129.5, 128.7, 116.9, 51.3, 25.9, 24.2, 20.4. Data in accordance with the literature.[27]

1-(4-Chlorophenyl)piperidine (23)

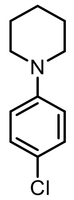

Following **GP5**, **S1** (27 mg, 0.1 mmol) gave **23** (83%). ^{1}H NMR (500 MHz, $CDCl_3$) δ 7.19–7.28 (2H, m), 6.85–6.88 (2H, m), 3.14 (4H, t, *J* = 5.4 Hz), 1.69–1.75 (4H, m), 1.58–1.61 (2H, m); ^{13}C NMR (101 MHz, $CDCl_3$) δ 150.8, 128.8, 123.9, 117.6, 50.6, 25.7, 24.2. Data in accordance with the literature.[28]

1-Methyl-6-phenylhexahydro-3,5-methanocyclopenta[b]pyrrol-2(1H)-one (29)

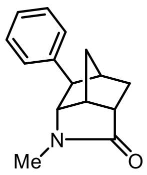

28 (33 mg, 0.1 mmol) gave **29** following **GP6** (76%) with **C1**, **GP4** (83%) with 10 mol% of **C1** and **GP5** (45%) as a white solid. FT-IR ν_{max} (film)/cm^{-1} 2962, 2360, 1682, 1400, 1257, 1018; ^{1}H NMR (500 MHz, $CDCl_3$) δ 7.34 (2H, t, *J* = 7.7 Hz), 7.22 (1H, t, *J* = 7.4 Hz), 7.18 (2H, d, *J* = 7.7 Hz), 3.76 (1H, d, *J* = 4.8 Hz), 3.12–3.01 (1H, m), 2.89 (3H, s), 2.69 (1H, br s), 2.69–2.66 (1H, m), 2.47 (1H, dd, *J* = 10.8, 4.5 Hz), 2.04–1.96 (1H, m), 1.87 (1H, d, *J* = 10.9 Hz), 1.72 (1H, d, *J* = 13.0 Hz), 1.49 (1H, d, *J* = 11.0 Hz); ^{13}C NMR (126 MHz, $CDCl_3$) δ 179.6, 142.9, 128.7, 127.3, 126.4, 68.3, 52.1, 45.2, 43.4, 42.4, 35.5, 34.6, 29.0; HRMS (HESI): Found MH^+ 282.1380 $C_{15}H_{18}NO$ requires 228.1383.

6-(4-Methoxyphenyl)-1-methylhexahydro-3,5-methanocyclopenta[b]pyrrol-2(1H)-one (30)

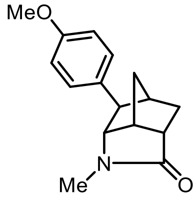

Following **GP4** at 75 ºC, **28** (33 mg, 0.1 mmol) gave **30** (52%) as a grey solid. [Petrol:EtOAc (20:80)]; FT-IR ν_{max} (film)/cm^{-1}2953, 1692, 1610, 1512, 1464, 1431, 1396, 1307, 1266,

1243, 1179, 1111, 1065, 1032, 1018; ^{1}H NMR, (500 MHz, $CDCl_3$) δ 7.09 (2H, d, *J* = 8.6 Hz), 6.87 (2H, d, *J* = 8.6 Hz), 3.80 (3H, s), 3.71 (1H, d, *J* = 4.4 Hz), 3.04 (1H, tq, *J* = 4.8, 1.6 Hz), 2.88 (3H, s), 2.63 (1H, br s), 2.62 (1H, br s), 2.46 (1H, dd, *J* = 10.7, 4.8 Hz), 2.00 (1H, ddd, *J* = 13.0, 10.9, 4.1 Hz), 1.84 (1H, dd, *J* = 11.0, 1.9 Hz), 1.74–1.68 (1H, m), 1.47 (1H, dq, *J* = 11.0, 1.8 Hz); ^{13}C NMR (126 MHz, $CDCl_3$) δ 179.6, 158.1, 135.0, 128.3, 114.1, 68.5, 55.4, 51.3, 45.1, 43.6, 42.4, 35.5, 34.6, 29.0; HRMS (HESI): Found MNa^+ 279.9174 $C_{16}H_{19}NO_2Na$ requires 279.9175.

6-(4-Fluorophenyl)-1-methylhexahydro-3,5-methanocyclopenta[b]pyrrol-2(1H)-one (31)

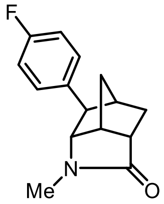

Following **GP4**, **28** (33 mg, 0.1 mmol) gave **31** (78%) as a solid. [Petrol:EtOAc (20:80)]; FT-IR ν_{max} (film)/cm^{-1} 2962, 2924, 2362, 1412, 1259, 1024; ^{1}H NMR (500 MHz, $CDCl_3$) δ 7.13 (2H, dd, *J* = 8.3, 5.3 Hz), 7.02 (2H, t, *J* = 8.4 Hz), 3.70 (1H, d, *J* = 4.7 Hz), 3.16–3.01 (1H, m), 2.89 (3H, s), 2.66 (1H, br s), 2.63 (1H, br s), 2.47 (1H, dd, *J* = 11.1, 4.5 Hz), 2.09–1.96 (1H, m), 1.82 (1H, d, *J* = 11.0 Hz), 1.71 (1H, d, *J* = 13.0 Hz), 1.50 (1H, d, *J* = 11.0 Hz); ^{13}C NMR (126 MHz, $CDCl_3$) δ 179.5, 161.4 (d, *J* = 245.2 Hz), 138.6 (d, *J* = 3.3 Hz), 128.7 (d, *J* = 7.8 Hz), 115.5 (d, *J* = 21.1 Hz), 68.5, 51.4, 45.1, 43.5, 42.3, 35.5, 34.5, 29.0; ^{19}F NMR (376 MHz, $CDCl_3$): δ –116.8; HRMS (HESI): Found MH^+ 246.1289 $C_{15}H_{17}FNO$ requires 246.1287.

6-(4-Chlorophenyl)-1-methylhexahydro-3,5-methanocyclopenta[b]pyrrol-2(1H)-one] (32)

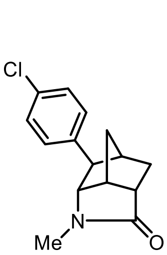

Following **GP4** in THF:DMF (9:1, 0.025 M) at 75 °C , **28** (33 mg, 0.1 mmol) gave **32** (73%) as a pale yellow solid. [Petrol:EtOAc (20:80)]; FT-IR ν_{max} (film)/cm^{-1} 2962, 1679, 1532,

1494, 1477, 1426, 1398, 1317, 1259, 1238, 1219, 1160; ^{1}H NMR (500 MHz, $CDCl_3$) δ 7.28 (2H, d, *J* = 8.3 Hz), 7.09 (2H, d, *J* = 8.2 Hz), 3.68 (1H, d, *J* = 4.3 Hz), 3.05 (1H, br s), 2.86 (3H, s), 2.63 (1H, br s), 2.62 (1H, br s), 2.45 (1H, dd, *J* = 10.4, 3.5 Hz), 2.00 (1H, td, *J* = 12.9, 4.1 Hz), 1.79 (1H, d, *J* = 10.8 Hz), 1.69 (1H, d, *J* = 13.0 Hz), 1.48 (1H, d, *J* = 11.5 Hz); ^{13}C NMR (126 MHz, $CDCl_3$) δ 179.4, 141.2, 132.1, 128.7, 128.6, 68.3, 51.5, 45.1, 43.3, 42.2, 35.3, 34.5, 29.0; HRMS (HESI): Found MH^+ 262.0993 $C_{15}H_{17}NOCl$ requires 262.0989.

4-(1-Methyl-2-oxooctahydro-3,5-methanocyclopenta[b]pyrrol-6-yl)benzonitrile (33)

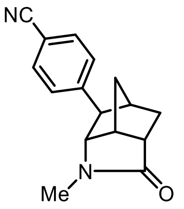

Following **GP4** in THF:DMF (9:1, 0.025 M) at 75 ºC, **28** (33 mg, 0.1 mmol) gave **33** (74%) as a yellow solid. [Petrol:EtOAc (20:80)]; FT-IR ν_{max} (film)/cm^{-1} 2961, 2357, 2227, 1695, 1606, 1505, 1479, 1432, 1398, 1319, 1260, 1092, 1066, 1011; ^{1}H NMR (500 MHz, $CDCl_3$) δ 7.60 (2H, d, *J* = 8.1 Hz), 7.27 (2H, d, *J* = 8.1 Hz), 3.72 (1H, d, *J* = 4.4 Hz), 3.08 (1H, dt, *J* = 4.7, 2.4 Hz), 2.87 (3H, s), 2.71 (1H, br s), 2.66 (1H, d, *J* = 3.2 Hz), 2.47 (1H, dd, *J* = 10.8, 4.6 Hz), 2.02 (1H, ddd, *J* = 12.4, 11.0, 4.1 Hz), 1.77 (1H, dq, *J* = 11.1, 1.9 Hz), 1.72 (1H, dt, *J* = 13.1, 2.1 Hz), 1.52 (1H, dq, *J* = 11.1, 1.7 Hz); ^{13}C NMR ($CDCl_3$, 126 MHz) δ 179.2, 148.2, 132.4, 128.1, 118.7, 110.3, 68.0, 52.2, 45.1, 43.1, 42.0, 35.2, 34.5, 29.0; HRMS (HESI): Found MH^+ 235.1335 $C_{16}H_{16}N_2O$ requires 235.1333.

6-(2-Methoxyphenyl)-1-methylhexahydro-3,5-methanocyclopenta[b]pyrrol-2(1H)-one (34)

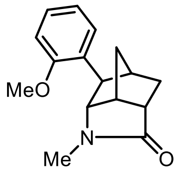

Following **GP4** with **C2**, **28** (33 mg, 0.1 mmol) gave **34** (26%) as an oil. [Petrol:EtOAc (20:80)]; FT-IR ν_{max} (film)/cm^{-1} 2959, 2362, 1620, 1598, 1585, 1526, 1435, 1398, 1311, 1165, 1103; ^{1}H NMR (500 MHz, $CDCl_3$) δ 7.23 (1H, td, *J* = 7.9, 1.4 Hz), 7.14 (1H, d, *J* = 7.5 Hz), 6.97–6.90 (1H, m), 6.88 (1H, d, *J* = 8.2 Hz), 3.84 (3H, s), 3.60 (1H, d, *J* = 4.3 Hz), 3.04–

2.97 (1H, m), 2.93 (3H, br s), 2.85 (1H, br s), 2.68 (1H, d, *J* = 3.3 Hz), 2.44 (1H, dd, *J* = 10.9, 4.5 Hz), 2.00 (1H, ddd, *J* = 13.0, 10.9, 4.2 Hz), 1.91 (1H, br dq, *J* = 10.8, 1.8 Hz), 1.72 (1H, dt, *J* = 13.0, 2.2 Hz), 1.52 (1H, dd, *J* = 10.8, 1.8 Hz); ^{13}C NMR (126 MHz, $CDCl_3$) δ 179.8, 157.3, 130.7, 127.5, 126.8, 120.2, 110.3, 68.1, 54.9, 46.5, 45.7, 42.6, 41.7, 35.2, 35.2, 29.4; HRMS (HESI): Found MH$^+$ 258.1488 $C_{16}H_{20}NO_2$ requires 258.1489.

6-(3-Fluorophenyl)-1-methylhexahydro-3,5-methanocyclopenta[b]pyrrol-2(1H)-one (35)

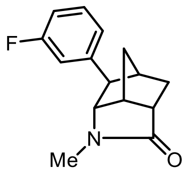

Following **GP4**, **28** (33 mg, 0.1 mmol) gave **35** (84%) as an off-white solid. [Petrol:EtOAc (20:80)]; FT-IR ν_{max} (film)/cm^{-1} 2961, 2360, 1684, 1411, 1257, 1016; ^{1}H NMR (500 MHz, $CDCl_3$) δ 7.30 (1H, td, *J* = 8.0, 6.2 Hz), 6.98–6.94 (1H, m), 6.94–6.83 (2H, m), 3.72 (1H, dd, *J* = 5.0, 1.4 Hz), 3.07 (1H, tq, *J* = 4.8, 1.5 Hz), 2.89 (3H, s), 2.70–2.63 (2H, m), 2.48 (1H, dd, *J* = 10.9, 4.7 Hz), 2.02 (1H, ddd, *J* = 13.0, 10.9, 4.1 Hz), 1.83 (1H, br dq, *J* = 11.0, 1.9 Hz), 1.71 (1H, dt, *J* = 12.9, 2.1 Hz), 1.51 (1H, dq, *J* = 11.2, 1.8 Hz); ^{13}C NMR (126 MHz, $CDCl_3$) δ 179.4, 163.1 (d, *J* = 246.0 Hz), 145.5 (d, *J* = 6.8 Hz), 130.2 (d, *J* = 8.3 Hz), 123.0 (d, *J* = 2.7 Hz), 114.3 (d, *J* = 21.6 Hz), 113.3 (d, *J* = 21.1 Hz), 68.4, 51.9, 45.1, 43.3, 42.2, 35.4, 34.6, 29.02; ^{19}F (376 MHz, $CDCl_3$) δ –112.7; HRMS (HESI): Found MH$^+$ 246.1287 $C_{15}H_{17}FNO$ requires 246.1289.

1-Methyl-6-(3-nitrophenyl)hexahydro-3,5-methanocyclopenta[b]pyrrol-2(1H)-one (36)

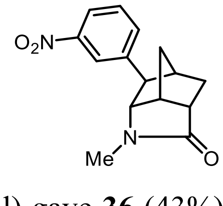

Following **GP4**, **28** (33 mg, 0.1 mmol) gave **36** (43%) as a pale yellow solid. [Petrol:EtOAc (20:80)]; FT-IR ν_{max} (film)/cm^{-1} 2961, 1691, 1524, 1479, 1433, 1347, 1320, 1258, 1082, 1019; ^{1}H NMR (500 MHz, $CDCl_3$) δ 8.10 (1H, ddd, *J* = 5.8, 4.1, 2.4 Hz), 8.05 (1H, s), 7.54–7.51 (2H, m), 3.78 (1H, d, *J* = 4.5 Hz), 3.18–3.09 (1H, m), 2.91 (3H, s), 2.78 (1H, br s), 2.72 (1H, d, *J* = 3.9 Hz), 2.52 (1H, dd, *J* = 11.0, 4.7 Hz), 2.07 (1H, ddd, *J* = 13.1, 10.9, 4.1 Hz), 1.81 (1H, dd, *J* = 11.3, 1.9 Hz), 1.76 (1H, dt, *J* = 13.2, 2.3 Hz), 1.57 (1H, dd, *J* = 11.2, 1.7 Hz); ^{13}C NMR (126 MHz, $CDCl_3$) δ 179.2, 148.6, 144.9, 133.8, 129.7, 122.0, 121.6, 68.1,

51.8, 45.2, 43.4, 42.1, 35.3, 34.5, 29.1; HRMS (HESI): Found MH+ 273.1230 $C_{15}H_{17}N_2O_3$ requires 273.1234.

1-Methyl-6-(naphthalen-2-yl)hexahydro-3,5-methanocyclopenta[b]pyrrol-2(1H)-one (37)

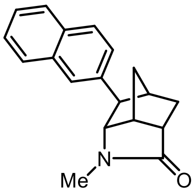

Following **GP4** with THF:DMF (9:1, 0.025 M) at 75 °C, **28** (33 mg, 0.1 mmol) gave **37** (65%) as a white solid. [Petrol:EtOAc (20:80)]; FT-IR ν_{max} (film)/cm^{-1} 2959, 1696, 1689, 1682, 1674, 1668, 1599, 1529, 1435, 1259, 1019; ^{1}H NMR (500 MHz, $CDCl_3$) δ 7.87–7.77 (3H, m), 7.57 (1H, s), 7.49 (1H, td, *J* = 7.0, 1.5 Hz), 7.46 (1H, td, *J* = 7.0, 1.3 Hz), 7.31 (1H, dd, *J* = 8.5, 1.9 Hz), 3.89 (1H, d, *J* = 4.7 Hz), 3.11 (1H, td, *J* = 4.7, 1.5 Hz), 2.94 (3H, s), 2.84 (1H, br s), 2.78 (1H, br s), 2.51 (1H, dd, *J* = 10.7, 4.4 Hz), 2.06 (1H, ddd, *J* = 13.0, 11.0, 4.1 Hz), 1.95 (1H, dd, *J* = 11.0, 1.9 Hz), 1.78 (1H, d, *J* = 13.0 Hz), 1.53 (1H, dd, *J* = 11.0, 1.8 Hz); ^{13}C NMR (126 MHz, $CDCl_3$) δ 179.6, 140.2, 133.4, 132.0, 128.5, 127.8, 127.7, 126.7, 126.5, 125.9, 124.9, 68.0, 52.2, 45.2, 43.5, 42.4, 35.5, 34.6, 29.1; HRMS (HESI): Found MH+ 278.1539 $C_{19}H_{20}NO_2$ requires 278.1535.

6-(Dibenzo[b,d]furan-4-yl)-1-methylhexahydro-3,5-methanocyclopenta[b]pyrrol-2(1H)-one (38)

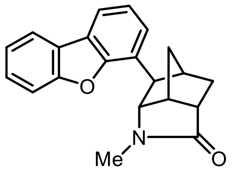

Following **GP4**, **28** (33 mg, 0.1 mmol) gave **38** (27%) as a yellow solid. [Petrol:EtOAc (20:80)]; FT-IR ν_{max} (film)/cm^{-1} 2959, 1682, 1475, 1451, 1184, 1020; ^{1}H NMR (500 MHz, $CDCl_3$) δ 7.96 (1H, d, *J* = 7.4 Hz), 7.87–7.82 (1H, m), 7.57 (1H, d, *J* = 8.2 Hz), 7.53–7.44 (1H, m), 7.41–7.34 (1H, m), 7.31 (1H, t, *J* = 7.6 Hz), 7.26 (1H, d, *J* = 7.4 Hz), 3.92 (1H, d, *J* = 4.4 Hz), 3.22 (1H, br s), 3.13 (3H, s), 3.11–3.08 (1H, m), 2.88 (1H, d, *J* = 3.7 Hz), 2.53 (1H, dd, *J* = 10.6, 4.4 Hz), 2.09 (1H, ddd, *J* = 13.1, 10.9, 4.1 Hz), 2.01 (1H, dd, *J* = 10.7, 2.1 Hz), 1.87 (1H, dt, *J* = 13.1, 2.1 Hz), 1.62–1.58 (1H, m); ^{13}C NMR (126 MHz, $CDCl_3$) δ 179.8, 156.0, 154.3, 127.4, 126.9, 124.4, 124.4, 124.3, 123.0, 122.8, 120.9, 119.0, 111.7,

67.7, 46.9, 45.5, 42.4, 42.0, 35.3, 35.2, 29.4; HRMS (HESI): Found MH^+ 318.1479 $C_{21}H_{20}NO_2$ requires 318.1489.

6-(2-Methoxypyridin-4-yl)-1-methylhexahydro-3,5-methanocyclopenta[b]pyrrol-2(1H)-one (39)

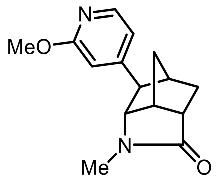

Following **GP4** with THF:DMF (9:1, 0.025 M) at 75 °C, **28** (33 mg, 0.1 mmol) gave **39** (60%) as a solid. [Petrol:EtOAc (20:80)]; FT-IR ν_{max} (film)/cm^{-1} 2974, 2876, 2360, 1695, 1607, 1553, 1480, 1447, 1393, 1318, 1241, 1217, 1187, 1154, 1046; ^{1}H NMR (500 MHz, $CDCl_3$) δ 8.10 (1H, d, *J* = 5.4 Hz), 6.69 (1H, dd, *J* = 5.4, 1.2 Hz), 6.53 (1H, s), 3.93 (3H, s), 3.70 (1H, d, *J* = 4.4 Hz), 3.14–2.99 (1H, m), 2.88 (3H, s), 2.67 (1H, d, *J* = 3.2 Hz), 2.59 (1H, br s), 2.47 (1H, dd, *J* = 10.8, 4.5 Hz), 2.02 (1H, ddd, *J* = 13.1, 10.9, 4.3 Hz), 1.78 (1H, dq, *J* = 11.1, 1.9 Hz), 1.71 (1H, dt, *J* = 13.1, 2.1 Hz), 1.51 (1H, dq, *J* = 11.2, 1.8 Hz); ^{13}C NMR (126 MHz, $CDCl_3$) δ 179.2, 164.6, 154.2, 147.0, 116.2, 109.1, 67.6, 53.4, 51.3, 44.9, 42.6, 42.0, 35.2, 34.6, 28.8; HRMS (HESI): Found MNa^+ 281.1258 $C_{15}H_{18}N_2O_2Na$ requires 281.1260.

6-(6-Fluoropyridin-3-yl)-1-methylhexahydro-3,5-methanocyclopenta[b]pyrrol-2(1H)-one (40)

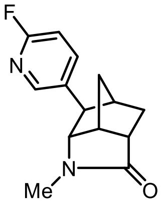

Following **GP4** with MTBE:DMF (9:1, 0.025 M) at 75 ºC, **28** (33 mg, 0.1 mmol) gave **40** (67%) as a yellow oil. [Petrol:EtOAc (20:80)]; FT-IR ν_{max} (film)/cm^{-1} 2962, 2361, 2353, 2342, 2339, 1694, 1593, 1485, 1435, 1398, 1257; ^{1}H NMR (500 MHz, $CDCl_3$) δ 8.03 (1H, s), 7.58 (1H, td, *J* = 8.1, 2.5 Hz), 6.90 (1H, dd, *J* = 8.5, 3.0 Hz), 3.69 (1H, d, *J* = 4.7 Hz), 3.10 (1H, t, *J* = 4.1 Hz), 2.87 (3H, s), 2.68 (1H, br s), 2.64 (1H, br s), 2.48 (1H, dd, *J* = 10.7, 4.4 Hz), 2.03 (1H, ddd, *J* = 13.1, 11.1, 4.1 Hz), 1.78 (1H, d, *J* = 11.2 Hz), 1.73 (1H, d, *J* = 13.1 Hz), 1.54 (1H, d, *J* = 11.3 Hz); ^{13}C NMR (126 MHz, $CDCl_3$) δ 179.2, 162.4 (d, *J* = 238.7 Hz), 146.3 (d, *J* = 14.5 Hz), 140.1 (d, *J* = 7.9 Hz), 135.8 (d, *J* = 4.6 Hz), 109.5 (d, *J* = 37.4

Hz), 68.1, 49.4, 45.2, 43.3, 42.0, 35.3, 34.4, 29.0; ^{19}F NMR (376 MHz, $CDCl_3$) δ –71.20; HRMS (HESI): Found MH^+ 247.1241 $C_{14}H_{16}N_2OF$ requires 247.1242.

6-(6-Chloropyridin-3-yl)-1-methylhexahydro-3,5-methanocyclopenta[b]pyrrol-2(1H)-one (41)

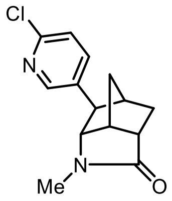

Following **GP4**, **28** (33 mg, 0.1 mmol) gave **41** (32%) as an oil. [Petrol:EtOAc (20:80)]; FT-IR ν_{max} (film)/cm^{-1} 2961, 2361, 2339, 2330, 1260, 1088, 1026; ^{1}H NMR (500 MHz, $CDCl_3$) δ 8.18 (1H, d, *J* = 2.5 Hz), 7.41 (1H, dd, *J* = 8.3, 2.5 Hz), 7.23 (1H, d, *J* = 6.3 Hz), 3.65 (1H, d, *J* = 4.7 Hz), 3.06 (1H, td, *J* = 4.8, 1.3 Hz), 2.82 (3H, s), 2.63 (1H, br s), 2.61–2.58 (1H, m), 2.44 (1H, dd, *J* = 10.8, 4.5 Hz), 1.99 (1H, ddd, *J* = 13.0, 10.9, 4.1 Hz), 1.70 (2H, br t, *J* = 13.0 Hz), 1.50 (1H, d, *J* = 11.1 Hz); ^{13}C NMR (126 MHz, $CDCl_3$) δ 179.1, 149.6, 148.8, 137.7, 137.1, 124.2, 67.9, 49.5, 45.1, 43.2, 41.9, 35.2, 34.4, 29.0; HRMS (HESI): Found MH^+ 263.0942 $C_{14}H_{16}N_2OCl$ requires 263.0946.

1-Methyl-6-(quinolin-3-yl)hexahydro-3,5-methanocyclopenta[b]pyrrol-2(1H)-one (42)

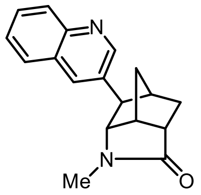

Following **GP4**, **28** (33 mg, 0.1 mmol) gave **42** (77%) as a yellow oil. [Petrol:EtOAc (20:80)]; FT-IR ν_{max} (film)/cm^{-1} 2963, 2370, 2366, 2355, 2340, 1649, 1259, 1022; ^{1}H NMR (500 MHz, $CDCl_3$) δ 8.80 (1H, s), 8.09 (1H, d, *J* = 8.4 Hz), 7.86 (1H, s), 7.80 (1H, d, *J* = 8.1 Hz), 7.70 (1H, ddd, *J* = 8.4, 6.8, 1.5 Hz), 7.57 (1H, t, *J* = 7.4 Hz), 3.88 (1H, d, *J* = 5.3 Hz), 3.21–3.12 (1H, m), 2.93 (3H, s), 2.89 (1H, br s), 2.78 (1H, d, *J* = 4.0 Hz), 2.53 (1H, dd, *J* = 5.1, 4.5 Hz), 2.09 (1H, ddd, *J* = 13.1, 10.9, 4.1 Hz), 1.90 (1H, d, *J* = 10.9 Hz), 1.82 (1H, d, *J* = 13.1 Hz), 1.57 (1H, d, *J* = 12.2 Hz); ^{13}C NMR (126 MHz, $CDCl_3$) δ 179.2, 151.4, 146.8, 135.2, 132.5, 129.2, 129.2, 127.6, 127.5, 127.1, 67.6, 49.9, 45.2, 43.1, 42.0, 35.2, 34.3, 29.0; HRMS (HESI): Found MH^+ 279.1484 $C_{18}H_{19}N_2O$ requires 279.1492.

1-Methyl-6-(thiophen-3-yl)hexahydro-3,5-methanocyclopenta[b]pyrrol-2(1H)-one (43)

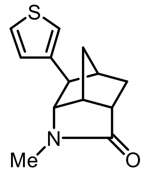

Following **GP4** with THF:DMF (9:1, 0.025 M) at 75 °C, **28** (33 mg, 0.1 mmol) gave **43** (60%) as a yellow oil. [Petrol:EtOAc (20:80)]; FT-IR ν_{max} (film)/cm^{-1} 2360, 1694, 1477, 1432, 1398, 1260, 1240, 1093, 1066, 1019; ^{1}H NMR (500 MHz, $CDCl_3$) δ 7.30 (1H, dd, *J* = 4.9, 2.9 Hz), 6.94–6.92 (1H, m), 6.91 (1H, dd, *J* = 3.2, 1.7 Hz), 3.67 (1H, d, *J* = 4.7 Hz), 3.02 (1H, dt, *J* = 4.7, 2.4 Hz), 2.86 (3H, s), 2.66 (1H, br s), 2.62 (1H, br s), 2.42 (1H, br s), 1.97 (1H, ddd, *J* = 13.0, 10.9, 4.1 Hz), 1.86–1.77 (1H, m), 1.67 (1H, d, *J* = 13.0 Hz), 1.49–1.43 (1H, m); ^{13}C NMR (126 MHz, $CDCl_3$) δ 179.4, 143.9, 127.4, 126.1, 119.5, 68.2, 48.2, 44.8, 43.6, 42.1, 35.1, 34.7, 28.9; HRMS (HESI): Found MH$^+$ 234.0947 $C_{13}H_{16}NOS$ requires 234.0946.

1-Methyl-6-(thiophen-2-yl)hexahydro-3,5-methanocyclopenta[b]pyrrol-2(1H)-one (44)

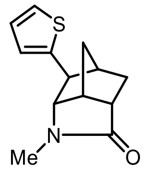

Following **GP3** with 10 mol% of **C2** and for 1h, **28** (33 mg, 0.1 mmol) gave **44** (64%) as a yellow oil. [Petrol:EtOAc (50:50)]; FT-IR ν_{max} (film)/cm^{-1} 2957, 1691, 1532, 1475, 1432, 1397, 1238, 1065, 1092, 1017 cm^{-1}; ^{1}H NMR (500 MHz, $CDCl_3$) δ 7.16 (1H, d, *J* = 5.0 Hz), 6.97–6.91 (1H, m), 6.78 (1H, d, *J* = 3.2 Hz), 3.71 (1H, d, *J* = 4.5 Hz), 3.11–2.93 (1H, m), 2.86 (3H, s), 2.83 (1H, br s), 2.65 (1H, br s), 2.45 (1H, dd, *J* = 10.6, 4.1 Hz), 1.97 (1H, ddd, *J* = 13.0, 10.9, 4.1 Hz), 1.90 (1H, d, *J* = 10.9 Hz), 1.68 (1H, d, *J* = 13.1 Hz), 1.51 (1H, d, *J* = 10.9 Hz); ^{13}C NMR (126 MHz, $CDCl_3$) δ 179.4, 147.0, 127.0, 123.6, 123.3, 69.5, 48.1, 44.9, 44.7, 41.9, 35.0, 34.9, 29.0; HRMS (HESI): Found MH$^+$ 234.0947 $C_{13}H_{16}NOS$ requires 234.0947.

1-Methyl-6-(1-methyl-1H-indol-5-yl)hexahydro-3,5-methanocyclopenta[b]pyrrol-2(1H)-one (45)

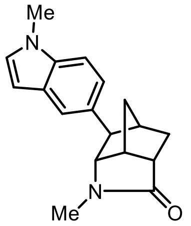

Following **GP4** 75 ºC, **28** (33 mg, 0.1 mmol) gave **45** (23%) as a yellow oil. [Petrol:EtOAc (20:80)]; FT-IR ν_{max} (film)/cm^{-1} 2959, 2923, 2854, 1727, 1691, 1512, 1480, 1444, 1431, 1394, 1328, 1318, 1261, 1248, 1241, 1118, 1087, 1069, 1019; ^{1}H NMR (500 MHz, $CDCl_3$) δ 7.40 (1H, br s), 7.30 (1H, d, *J* = 8.5 Hz), 7.09–7.02 (2H, m), 6.45 (1H, d, *J* = 2.9 Hz), 3.85 (1H, d, *J* = 4.7 Hz), 3.79 (3H, s), 3.07 (1H, t, *J* = 4.2 Hz), 2.91 (3H, s), 2.81 (1H, br s), 2.70 (1H, d, *J* = 3.9 Hz,), 2.48 (1H, dd, *J* = 10.8, 4.6 Hz), 2.08–1.92 (2H, m), 1.74 (1H, dt, *J* = 13.1, 2.1 Hz), 1.48 (1H, d, *J* = 10.9 Hz); ^{13}C NMR (126 MHz, $CDCl_3$) δ 179.7, 135.4, 133.9, 129.5, 128.6, 121.8, 118.6, 109.5, 100.9, 68.7, 52.1, 45.2, 44.0, 42.5, 35.7, 34.6, 33.0, 29.1; HRMS (HESI): Found M 280.1304 $C_{18}H_{20}N_2O$ requires 280.1376.

5-Benzyl-1-methylpyrrolidin-2-one (46)

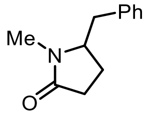

Following **GP6** with MTBE:DMF (9:1, 0.025 M) at 75 °C, **S13** (29 mg, 0.1 mmol) gave **46** (73%) as a pale yellow oil. [Petrol:EtOAc (20:80)]; FT-IR ν_{max} (film)/cm^{-1} 2925, 1683, 1453, 1423, 1399, 1308, 1253, 1113; ^{1}H NMR (400 MHz, $CDCl_3$) δ 7.35–7.27 (2H, m), 7.26–7.21 (1H, m), 7.18–7.10 (2H, m), 3.74 (1H, hept, *J* = 4.3 Hz), 3.02 (1H, dd, *J* = 13.5, 4.4 Hz), 2.87 (3H, s), 2.63 (1H, dd, *J* = 13.5, 8.2 Hz), 2.24–2.07 (2H, m), 1.98 (1H, ddt, *J* = 13.0, 9.9, 7.6 Hz), 1.73 (1H, dddd, *J* = 13.2, 9.1, 5.9, 4.2 Hz); ^{13}C NMR (100 MHz, $CDCl_3$) δ 175.3, 137.0, 129.3, 128.7, 126.8, 61.2, 39.5, 29.8, 28.3, 23.6. HRMS (HESI): Found MNa^+ 212.1043 $C_{12}H_{15}NONa$ requires 212.1046.

5-Benzyl-1-phenethylpyrrolidin-2-one (47)

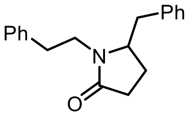

Following **GP4** with **C2** in MTBE:DMF (9:1, 0.025 M) at 70 °C, **S14** (39 mg, 0.1 mmol) gave **47** (20.5 mg, 76%) as an oil. [Petrol:EtOAc (50:50)]; FT-IR ν_{max} (film)/cm^{-1} 2924, 1679, 1496, 1453, 1418, 1367, 1270, 1154, 1030; ^{1}H NMR (400 MHz, $CDCl_3$) δ 7.39–7.23 (8H, m), 7.12 (2H, d, *J* = 7.1 Hz), 4.04 (1H, dt, *J* = 13.9, 7.1 Hz), 3.69–3.59 (1H, m), 3.20 (1H, dt, *J* = 14.3, 7.4 Hz), 3.02–2.81 (3H, m), 2.57 (1H, dd, *J* = 13.1, 8.6 Hz), 2.28–2.13 (2H, m), 1.98–1.81 (1H, m), 1.78–1.67 (1H, m); ^{13}C NMR (100 MHz, $CDCl_3$) δ 175.2, 139.1, 137.1, 129.3, 128.9, 128.7, 128.7, 126.9, 126.6, 59.2, 42.3, 39.5, 34.2, 29.9, 24.0.; HRMS (APCI): Found MH$^+$ 280.1703 $C_{19}H_{22}ON$ requires 280.1696.

***tert*-Butyl 8-benzyl-7-methyl-6-oxo-1,7-diazaspiro[4.4]nonane-1-carboxylate (48)**

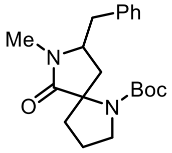

Following **GP4** with **C2** in MTBE:DMF (9:1, 0.025 M) at 70 °C, **S15** (59 mg, 0.13 mmol) gave **48** (26 mg, 58%) as an amorphous solid. d.r 2.5:1 [Petrol:EtOAc (10:90)]; FT-IR ν_{max} (film)/cm^{-1} 2924, 1686, 1479, 1447, 1390, 1360, 1281, 1168, 1144, 1089; ^{1}H NMR (500 MHz, $CDCl_3$, diastereomers and rotamers) δ 7.37–7.25 (2.4H, m), 7.25–7.23 (0.5H, m), 7.21 (0.6H, d, *J* = 7.6 Hz), 7.17 (0.9H, d, *J* = 7.4 Hz), 7.13 (0.6H, d, *J* = 7.3 Hz), 3.94 (0.1H, tt, *J* = 8.3, 3.8 Hz), 3.74 (0.2H, dq, *J* = 9.5, 3.8 Hz), 3.57 – 3.40 (2.7H, m), 3.33 (0.9H, td, *J* = 12.9, 4.5 Hz), 3.01 (0.3H, ddd, *J* = 25.2, 13.8, 4.5 Hz), 2.95 (0.4H, s), 2.93 (0.7H, s), 2.93 (0.6H, s), 2.91 (1.3H, s), 2.70 (0.3H, t, *J* = 11.4 Hz), 2.68–2.59 (0.2H, m), 2.55 – 2.44 (0.6H, m), 2.39 (0.2H, dd, *J* = 13.5, 9.7 Hz), 2.26 (0.3H, dd, *J* = 12.5, 8.6 Hz), 2.06 (0.5H, dd, *J* = 12.5, 9.2 Hz), 2.05–1.95 (1H, m), 1.94–1.84 (1H, m), 1.79–1.63 (3.5H, m), 1.46 (6.5H, s), 1.40 (1H, s), 1.38 (1.5H, s); ^{13}C NMR (126 MHz, $CDCl_3$, diastereomers and rotamers) δ 175.2^M &175.1^M, 175.1^m & 175.0^m, 153.5^m &153.3^m, 153.5^M &153.3^M, 137.6^M &136.8^M, 137.2^m & 136.9^m, 129.6^m & 129.6^m, 129.3^M & 129.2^M, 128.9^M & 128.7^M, 128.7^m & 128.7^m, 127.1^m & 126.8^m, 127.0^M & 126.7^M, 80.4^M, 79.6^M, 79.8^m & 79.6^m, 66.8^M & 66.8^M, 66.2^m & 66.1^m, 57.9^m & 57.9^m, 57.5^M & 57.1^M, 47.9^M & 47.6^M, 47.8^m & 47.5^m, 40.7^M & 40.1^M, 40.2^m &

39.8^{m}, 39.7^{m} & 39.5^{m}, 38.5^{M} & 37.1^{M}, 36.8^{M} & 36.5^{M}, 36.3^{m} & 35.9^{m}, 29.8^{M} & 28.8^{M}, 29.2^{m} & 28.6^{m}, $28.7^{M\&m}$, 28.6^{M} & 28.5^{M}, 23.7^{m} & 23.1^{m}, 23.5^{M} & 22.7^{M}.; HRMS (APCI): Found MH^{+} 345.2166 $C_{20}H_{29}O_{3}N_{2}$ requires 345.2173.

1-(1,1-Dioxidotetrahydrothiophen-3-yl)-5-methylpyrrolidin-2-one (49)

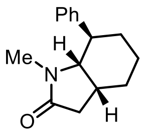

Following **GP4** with **C2** in MTBE:DMF (9:1, 0.025 M) at 70 °C, **S16** (34 mg, 0.10 mmol) gave **49** (96%) as an oil. dr 4:1. [Petrol:EtOAc (50:50)]; FT-IR ν_{max} (film)/cm^{-1} 2936, 1687, 1601, 1456, 1395, 1284, 1263, 1236, 1108, 1083; ^{1}H NMR (400 MHz, $CDCl_3$, diastereomers) δ 7.33–7.23 (3.5H, m), 7.22–7.14 (1.5H, m), 3.76 (0.2H, d, *J* = 5.9 Hz), 3.61 (0.8H, br s), 3.24 (0.8H, br s), 2.95–2.91 (2.4H, m), 2.76 (0.2H, d, *J* = 4.2 Hz), 2.47 (0.2H, s (br)), 2.44 (1.2H, s, (br)), 2.33–1.93 (2H, m), 1.92–1.68 (4H, m), 1.63–1.47 (1.2H, m), 1.19–1.06 (0.7H, m), 0.84 (0.7H, dq, *J* = 12.6, 7.1 Hz); ^{13}C NMR (101 MHz, $CDCl_3$, diastereomers) δ 177.8^{M} & 176.1^{m}, 143.3^{m} & 142.6^{M}, 128.8^{M} & 128.7^{m}, 127.8^{M} & 127.2^{m}, 126.7^{m} &126.4^{M}, 65.2^{m} & 63.7^{M}, 43.3^{m} & 40.7^{M}, 39.3^{m} & 37.1^{M}, 30.7^{M} & 29.8^{m}, 27.7^{M} & 25.5^{m}, 24.2^{M} & 23.0^{m}, 21.8^{M} & 20.9^{m}.; HRMS (APCI): Found MH^{+} 229.1470 $C_{15}H_{19}NO$ requires 229. 1467.

4-Benzyl-3-cyclohexyloxazolidin-2-one (50)

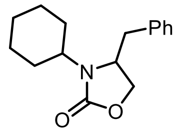

Following **GP4** with **C2** in MTBE:DMF (9:1, 0.025 M) at 70 °C, **S17** (37 mg, 0.1 mmol) gave **50** (19 mg, 73%) as an oil. [Petrol:EtOAc (75:25)]; FT-IR ν_{max} (film)/cm^{-1} 2931, 2855, 1742, 1496, 1447, 1423, 1288, 1081; ^{1}H NMR (500 MHz, $CDCl_3$) δ 7.33 (2H, t, *J* = 7.3 Hz), 7.30–7.23 (1H, m), 7.17 (2H, d, *J* = 6.8 Hz), 4.06–3.95 (3H, m), 3.55–3.47 (1H, m), 3.23 (1H, dd, *J* = 13.5, 3.2 Hz), 2.70–2.62 (1H, m), 2.04–1.95 (1H, m), 1.92–1.75 (4H, m), 1.72–1.65 (1H, m), 1.62 (1H, td, *J* = 12.7, 3.7 Hz), 1.41–1.28 (2H, m), 1.19 (1H, tt, *J* = 13.1, 3.5 Hz); ^{13}C NMR (101 MHz, $CDCl_3$) δ 157.8, 136.0, 129.2, 129.1, 127.3, 66.8, 56.0, 54.5, 40.9, 32.0, 30.6, 26.1, 26.0, 25.5; HRMS (ESI): Found MH^{+} 260.1642 $C_{16}H_{22}O_{2}N$: requires 260.1645.

4-Benzyl-3-methylthiazolidin-2-one (51)

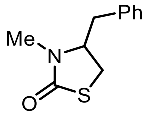

Following Following **GP4** with **C2** in DCE:DMF (9:1, 0.025 M) at 70 °C, **S18** (31 mg, 0.10 mmol) gave **51** (10 mg, 48%) as an oil. [Petrol:EtOAc (50:50)]; FT-IR ν_{max} (film)/cm^{-1} 2924, 1665, 1604, 1527, 1497, 1454, 1418, 1386, 1343, 1285, 12227, 1069; ^{1}H NMR (500 MHz, $CDCl_3$) 7.36–7.27 (3H, m), 7.20 (2H, d, *J* = 7.4 Hz), 3.92–3.85 (1H, m), 3.23 (1H, dd, *J* = 11.3, 7.4 Hz), 3.15 (1H, dd, *J* = 13.5, 4.5 Hz), 2.95 (1H, dd, *J* = 11.2, 4.1 Hz), 2.93 (3H, s), 2.81 (1H, dd, *J* = 13.5, 9.6 Hz); ^{13}C NMR (126 MHz, $CDCl_3$) δ 171.7, 136.4, 129.4, 129.1, 127.3, 62.7, 37.6, 30.8, 30.3; HRMS (ESI): Found M^{+} 207.0715 $C_{11}H_{13}NOS$ requires 207.0718.

***tert*-Butyl 2-Benzylpyrrolidine-1-carboxylate (53)**

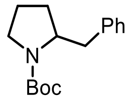

Following **GP4** with MTBE:DMF (9:1, 0.025 M), **52** (37 mg, 0.1 mmol) gave **53** (73%) as a pale yellow oil. [Petrol:CH_2Cl_2 (20:80)]; FT-IR ν_{max} (film)/cm^{-1} 2962, 1697, 1392, 1257, 1015; ^{1}H NMR (500 MHz, $CDCl_3$, rotamers) δ 7.33–7.23 (2H, m), 7.25–7.13 (3H, m), 4.03 (0.4H, br s), 3.95 (0.6H, br s), 3.36 (1.2H, br s), 3.28 (0.8H, s), 3.16 (0.4H, d, 12.9 Hz), 3.05 (0.6H, d, 12.1 Hz), 2.62–2.46 (1H, m), 1.82–1.61 (4H, m), 1.51 (9H, s); ^{13}C NMR (126 MHz, $CDCl_3$, rotamers) δ 154.7, 139.3, 129.7 & 129.5, 128.5 & 128.4, 126.3 & 126.2, 79.4 & 79.2, 59.0 & 58.8, 47.0 & 46.4, 40.7 & 39.7, 29.9 & 29.8, 28.8, 22.8; HRMS (HESI): Found MH^{+} 262.1800 $C_{16}H_{24}NO_2$ requires 262.1802. Data in accordance with the literature[30].

***tert*-Butyl 2-(4-Methoxybenzyl)pyrrolidine-1-carboxylate (54)**

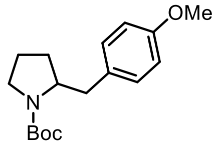

Following **GP4** with MTBE:DMF (9:1, 0.025 M), **52** (37 mg, 0.1 mmol) gave **54** (61%) as a pale yellow oil. [Petrol:CH_2Cl_2 (20:80)]; FT-IR ν_{max} (film)/cm^{-1} 2962, 1695, 1512, 1393, 1258, 1172, 1016; ^{1}H NMR (500 MHz, $CDCl_3$, rotamers) δ 7.16 – 6.98 (2H, m), 6.83 (2H, d,

J = 7.9 Hz), 3.99 (0.4H, br s), 3.90 (0.6H, s), 3.78 (3H, s), 3.35 (1.2H, br s), 3.27 (0.8H, br s), 3.07 (0.4H, d, J = 12.1 Hz), 2.97 (0.6H, d, J = 12.3 Hz), 2.51 (1H, m), 1.78 – 1.69 (4H, m), 1.51 (9H, s); ^{13}C NMR (126 MHz, $CDCl_3$, rotamers) δ 158.2, 154.7, 131.4, 130.6 & 130.4, 113.9 & 113.8, 79.3 & 79.1, 59.1 & 58.9, 55.4, 46.9 & 46.5, 39.7 & 38.73, 29.7, 28.8, 23.6 & 22.8; HRMS (HESI): Found MH^+ 292.1905 $C_{17}H_{26}NO_3$ requires 292.1907. Data in accordance with the literature.[31]

***tert*-Butyl 2-Benzylpyrrolidine-1-carboxylate (55)**

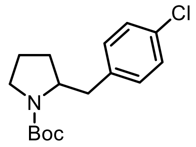

Following **GP4** with MTBE:DMF (9:1, 0.025 M), **52** (37 mg, 0.1 mmol) gave **55** (75%) as a pale yellow oil. [Petrol:CH_2Cl_2 (20:80)]; FT-IR ν_{max} (film)/cm^{-1} 2961, 1674, 1410, 1258, 1016; ^{1}H NMR (500 MHz, $CDCl_3$, rotamers) δ 7.24 (2H, s), 7.11 (2H, dd, J = 20.7, 8.1 Hz), 4.00 (0.5H, br s), 3.91 (0.5H, br s), 3.35 (1H, br s), 3.27 (1H, br s), 3.10 (0.5H, d, J = 13.3 Hz), 2.99 (0.5H, d, J = 13.1 Hz), 2.54 (1H, dt, J = 21.8, 11.7 Hz), 1.70 (4H, m), 1.49 (9H, s); ^{13}C NMR (126 MHz, $CDCl_3$, rotamers) δ 154.6, 137.8, 132.2 & 132.0, 131.0 & 130.8, 128.7 & 128.5, 79.6 & 79.2, 58.8 & 58.6, 47.0 & 46.5, 40.1 & 39.1, 29.9, 28.7, 22.8. HRMS (HESI): Found MH-*t*Bu$^+$ 240.0780 $C_{12}H_{15}NO_2Cl$ requires 240.0786.

***tert*-Butyl 2-(4-Cyanobenzyl)pyrrolidine-1-carboxylate (56)**

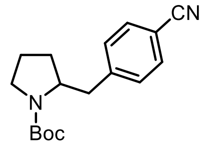

Following **GP4** with MTBE:DMF (9:1, 0.025 M), **52** (37 mg, 0.1 mmol) gave **56** (73%) as a pale yellow oil. [Petrol:CH_2Cl_2 (20:80)]; FT-IR ν_{max} (film)/cm^{-1} 2970, 2931, 2873, 2227, 1725, 1689, 1608, 1454, 1392, 1365, 1342, 1256, 1169, 1109; ^{1}H NMR (500 MHz, $CDCl_3$, rotamers) δ 7.56 (2H, s), 7.37–7.21 (2H, m), 4.02 (0.5H, br s), 3.95 (0.5H, br s), 3.43–3.21 (2H, m), 3.18 (0.5H, d, J = 14.0 Hz), 3.06 (0.5H, d, J = 11.3 Hz), 2.64 (1H, p, J = 10.7 Hz), 1.90–1.66 (3H, m), 1.64–1.56 (1H, m), 1.47 (9H, s); ^{13}C NMR (126 MHz, $CDCl_3$, rotamers) δ 154.7 & 154.4, 145.0, 132.3 & 132.2, 130.4 & 130.3, 119.0, 110.3 & 110.2, 79.7 & 79.4, 58.5 & 58.4, 47.0 & 46.4, 41.0 & 40.0, 30.0 & 29.2, 28.7, 23.6 & 22.8; HRMS (HESI):

Found MNa$^+$ 309.1568 $C_{17}H_{22}N_2O_2Na$ requires 309.1573. Data in accordance with the literature.[32]

***tert*-Butyl 2-(Quinolin-3-ylmethyl)pyrrolidine-1-carboxylate (57)**

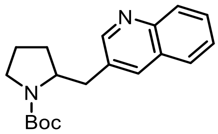

Following **GP4** with MTBE:DMF (9:1, 0.025 M), **52** (37 mg, 0.1 mmol) gave **57** (55%) as a pale yellow oil. [CH_2Cl_2:Et_2O (95:5)]; FT-IR ν_{max} (film)/cm^{-1} 2959, 2927, 2874, 1725, 1691, 1494, 1391, 1365, 1259, 1168, 1116, 1089, 1071; ^{1}H NMR (500 MHz, $CDCl_3$, rotamers) δ 8.77 (1H, d, *J* = 2.1 Hz), 8.07 (1H, d, *J* = 8.4 Hz), 8.02–7.84 (1H, m), 7.76 (1H, d, *J* = 8.1 Hz), 7.66 (1H, t, *J* = 7.8 Hz), 7.52 (1H, t, *J* = 7.6 Hz), 4.13 (0.5H, br s), 4.05 (0.5H, br s), 3.51–3.14 (3H, m), 2.97–2.85 (0.5H, m), 2.81–2.70 (0.5H, m), 1.93–1.55 (4H, m), 1.48 (9H, s); ^{13}C NMR (126 MHz, $CDCl_3$, rotamers) δ 154.8 & 154.6, 152.5 & 152.3, 147.1, 136.0 & 135.6, 132.0, 129.3, 129.0 & 128.9, 128.2, 127.5, 126.8 & 126.7, 79.8 & 79.4, 58.7 & 58.4, 47.0 & 46.5, 38.3 & 36.9, 30.1 & 29.2, 28.7, 23.7 & 22.9; HRMS (HESI): Found MNa$^+$ 335.1726 $C_{19}H_{24}N_2O_2Na$ requires 335.1730.

***tert*-Butyl 2-((1-methyl-1H-indol-5-yl)methyl)pyrrolidine-1-carboxylate (58)**

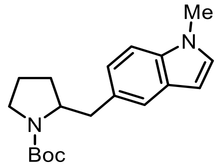

Following **GP4**, **52** (37 mg, 0.1 mmol) gave **58** (28%) as a pale yellow oil. [Petrol:CH_2Cl_2 (5:95)];FT-IR ν_{max} (film)/cm^{-1} 2957, 2929, 2870, 2857, 1726, 1696, 1393, 1271, 1119; ^{1}H NMR (500 MHz, $CDCl_3$, rotamers) δ 7.54–7.36 (1H, m), 7.25 (1H, d, *J* = 9.1 Hz), 7.18–6.94 (2H, m), 6.42 (1H, s, 1H), 4.08 (0.4H, br s), 3.98 (0.6H, br s), 3.47–3.10 (3H, m), 2.68–2.53 (1H, m), 1.75 (4H, br s), 1.55 & 1.52 (9H, s); ^{13}C NMR (126 MHz, $CDCl_3$, rotamers) δ 154.8, 135.7, 130.1 & 129.1, 128.9 & 128.7, 123.7 & 123.3, 121.5 & 121.3, 109.2 & 109.0, 100.6, 79.3 & 79.0, 59.7 & 59.6, 47.0 & 46.5, 40.6, 38.3, 33.0 & 31.4, 29.8 & 29.6, 28.8, 23.5 & 22.8; HRMS (HESI): Found MNa$^+$ 337.1883 $C_{19}H_{26}N_2O_2Na$ requires 337.1886.

***tert*-Butyl 2-(Benzo[b]thiophen-2-ylmethyl)pyrrolidine-1-carboxylate (59)**

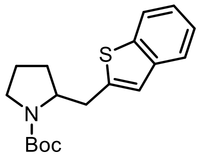

Following **GP4**, **52** (37 mg, 0.1 mmol) gave **59** (30%) as a pale yellow oil. [Petrol:CH_2Cl_2 (10:90)]; FT-IR ν_{max} (film)/cm^{-1} 2962, 1698, 1259, 1094, 1081, 1072, 1059, 1037, 1023; ^{1}H NMR (500 MHz, $CDCl_3$, rotamers) δ 7.77 (1H, d, *J* = 7.9 Hz), 7.68 (1H, d, *J* = 7.7 Hz), 7.36–7.22 (2H, m), 7.03 (1H, d, *J* = 10.7 Hz), 4.14 (0.4H, br s), 4.05 (0.6H, br s), 3.47–3.19 (3H, m), 3.12–3.03 (0.4H, m), 3.01–2.92 (0.6H, m), 1.84–1.67 (3H, m), 1.58 (1.5H, s), 1.52 (7.5H, s); ^{13}C NMR (126 MHz, $CDCl_3$, rotamers) δ 154.6, 142.4, 140.1 & 140.0, 124.3 & 124.1, 123.8 & 123.6, 122.9, 122.5 & 122.3, 122.2, 79.7 & 79.4, 58.5 & 58.1, 47.2 & 46.7, 35.8 & 34.6, 30.4 & 29.6, 28.8, 23.7 & 23.0; HRMS (HESI): Found MNa^+ 340.1338 $C_{18}H_{23}NO_2SNa$ requires 340.1342.

1,6-Dimethylhexahydro-3,5-methanocyclopenta[b]pyrrol-2(1H)-one (60)

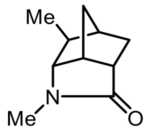

Following **GP3**, **28** (35 mg, 0.1 mmol) gave **60** (88%) as a pale yellow oil. [Et_2O:EtOAc (95:5)]; FT-IR ν_{max} (film)/cm^{-1} 2958, 1694, 1683, 1455, 1429, 1396, 1261, 1239, 1063, 1009; ^{1}H NMR (500 MHz, $CDCl_3$) δ 3.01 (1H, d, *J* = 4.7 Hz), 2.90 (1H, t, *J* = 4.0 Hz), 2.77 (3H, s), 2.33 (1H, dd, *J* = 10.7, 4.2 Hz), 2.09 (1H, br s), 1.84 (1H, ddd, *J* = 12.8, 10.9, 4.0 Hz), 1.74 (1H, d, *J* = 12.3 Hz), 1.55–1.44 (2H, m), 1.38 (1H, d, *J* = 10.9 Hz), 0.91 (3H, d, *J* = 7.4 Hz); ^{13}C NMR (126 MHz, $CDCl_3$) δ 179.9, 68.8, 44.8, 43.9, 41.9, 41.5, 35.4, 33.7, 28.9, 19.2; HRMS (HESI): Found MH^+ 166.1227 $C_{10}H_{16}NO$ requires 166.1226.

6-Ethyl-1-methylhexahydro-3,5-methanocyclopenta[b]pyrrol-2(1H)-one (61)

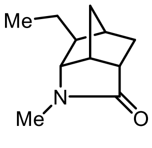

Following **GP3**, **28** (35 mg, 0.1 mmol) gave **61** (60%) as a pale yellow oil. [Et_2O:EtOAc (95:5)]; FT-IR ν_{max} (film)/cm^{-1} 2960, 2873, 2363, 1699, 1456, 1429, 1397, 1259, 1237, 1221,

1062, 1010; ^{1}H NMR (500 MHz, $CDCl_3$) δ 3.06 (1H, d, *J* = 4.8 Hz), 2.93–2.86 (1H, m), 2.75 (3H, s), 2.35 (1H, dd, *J* = 10.8, 4.5 Hz), 2.20 (1H, br s), 1.85 (1H, ddd, *J* = 12.8, 10.8, 4.0 Hz), 1.68 (1H, dd, *J* = 10.9, 1.9 Hz), 1.48 (1H, dt, *J* = 12.8, 2.1 Hz), 1.37 (1H, d, *J* = 10.9 Hz), 1.34–1.07 (3H, m), 0.91 (3H, t, *J* = 7.1 Hz); ^{13}C NMR (126 MHz, $CDCl_3$) δ 179.6, 66.8, 49.7, 44.8, 42.4, 42.2, 35.6, 34.2, 28.6, 25.7, 11.8; HRMS (HESI): Found MH^+ 180.1383 $C_{11}H_{18}NO$ requires 180.1383.

6-Isopentyl-1-methylhexahydro-3,5-methanocyclopenta[b]pyrrol-2(1H)-one (62)

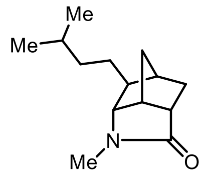

Following **GP3**, **28** (35 mg, 0.1 mmol) gave **62** (56%) as a pale yellow oil. [Et_2O:EtOAc (95:5)]; FT-IR ν_{max} (film)/cm^{-1} 2957, 1703, 1427, 1396, 1258, 1017; ^{1}H NMR (500 MHz, $CDCl_3$) δ 3.05 (1H, d, *J* = 4.8 Hz), 2.89 (1H, tq, *J* = 4.7, 1.5 Hz), 2.75 (3H, s), 2.34 (1H, dd, *J* = 10.8, 4.6 Hz), 2.18 (1H, d, *J* = 3.0 Hz), 1.84 (1H, ddd, *J* = 12.8, 10.9, 4.0 Hz), 1.69 (1H, dq, *J* = 10.8, 1.9 Hz), 1.54–1.43 (2H, m), 1.37 (1H, dd, *J* = 10.9, 1.7 Hz), 1.25–1.06 (5H, m), 0.86 (3H, d, *J* = 6.6 Hz), 0.85 (3H, d, *J* = 6.6 Hz); ^{13}C NMR (126 MHz, $CDCl_3$) δ 179.6, 67.3, 48.0, 44.8, 42.7, 42.2, 36.6, 35.6, 34.2, 30.8, 28.7, 28.1, 22.7; HRMS (HESI): Found MH^+ 222.1851 $C_{14}H_{23}NO$ requires 222.1852.

6-(3,7-Dimethyloctyl)-1-methylhexahydro-3,5-methanocyclopenta[b]pyrrol-2(1H)-one (63)

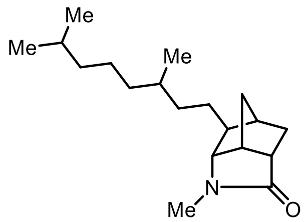

Following **GP3**, **28** (35 mg, 0.1 mmol) gave **63** (57%) as a pale yellow oil. [Et_2O:EtOAc (95:5)]; dr 1:1. FT-IR ν_{max} (film)/cm^{-1} 2955, 2938, 2366, 2357, 2339, 1704, 1259, 1096, 1025; ^{1}H NMR (500 MHz, $CDCl_3$, diastereomers) δ 3.05 (1H, d, *J* = 4.8 Hz), 2.89 (1H, br t, *J* = 4.8 Hz), 2.74 (3H, s), 2.34 (1H, dd, *J* = 10.7, 4.5 Hz), 2.18 (1H, br s), 1.84 (1H, ddd, *J* = 12.8, 11.0, 4.0 Hz), 1.69 (1H, d, *J* = 10.9 Hz), 1.56–1.45 (2H, m), 1.39–1.02 (13H, m), 0.84 (6H, d, *J* = 6.7 Hz), 0.83 (3H, d, *J* = 6.5 Hz), 0.82 (3H, d, *J* = 6.5 Hz); ^{13}C NMR (126 MHz, $CDCl_3$, diastereomers) δ 179.6, 67.4 & 67.3, 48.1 & 48.0, 44.8 & 44.7, 42.8 & 42.6, 42.2,

39.4, 37.3, 35.6, 34.8 & 34.7, 34.3 & 34.2, 32.9 & 32.8, 30.4, 28.7, 28.0, 24.9 & 24.8, 22.8 & 22.7, 19.8; HRMS (HESI): Found MH$^+$ 292.2625 $C_{19}H_{34}NO$ requires 292.2635.

6-Cyclopropyl-1-methylhexahydro-3,5-methanocyclopenta[b]pyrrol-2(1H)-one (65)

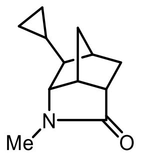

Following **GP3**, **28** (35 mg, 0.1 mmol) gave **65** (83%) as a pale yellow oil. [Et_2O:EtOAc (95:5)]; FT-IR ν_{max} (film)/cm^{-1} 2960, 1699, 1428, 1397, 1257, 1015; ^{1}H NMR (500 MHz, $CDCl_3$) δ 3.28 (1H, d, *J* = 4.8 Hz), 2.97–2.89 (1H, m), 2.72 (3H, s), 2.35 (2H, br q, *J* = 5.5 Hz), 1.90–1.72 (3H, m), 1.49–1.37 (2H, m), 0.63–0.46 (3H, m), 0.23–0.10 (1H, m), 0.06–(–0.02) (1H, m); ^{13}C NMR (126 MHz, $CDCl_3$) δ 179.6, 67.7, 53.8, 44.6, 43.1, 42.2, 35.4, 34.7, 28.6, 14.2, 5.3, 4.3; HRMS (HESI): Found MH$^+$ 192.1383 $C_{12}H_{18}NO$ requires 192.1383.

***tert*-Butyl 2-ethylpyrrolidine-1-carboxylate (66)**

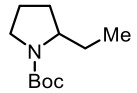

Following **GP3**, **52** (37 mg, 0.1 mmol) gave **66** (44%). ^{1}H NMR (500 MHz, $CDCl_3$) δ 3.95–3.56 (m, 1H), 3.55–3.12 (m, 2H), 2.15–1.74 (m, 4H), 1.74–1.9 (m, 2H), 1.59–1.40 (s, 9H), 0.80 (t, *J* = 7.7 Hz, 3H); ^{13}C NMR (126 MHz, $CDCl_3$) δ 154.8, 78.8, 58.7, 46.6, 46.2, 30.2, 29.3, 28.6, 27.5, 26.8, 23.8, 23.1, 10.6. Data in accordance with the literature.[33]

***tert*-Butyl 2-propylpyrrolidine-1-carboxylate (67)**

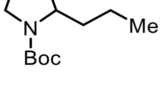

Following **GP3**, **52** (37 mg, 0.1 mmol) gave **67** (58%). ^{1}H NMR (500 MHz, $CDCl_3$, rotamers) δ 3.59–3.83 (1H, m), 3.36 (0.55H, br s), 3.29 (1.45H, br s), 1.56–1.95 (5H, m), 1.45 (9H, s), 1.28–1.36 (3H, m), 0.91 (3H, t, *J* = 7.2 Hz); ^{13}C NMR (126 MHz, $CDCl_3$) δ 154.7, 78.8, 57.2, 56.9, 46.4, 46.0, 36.9, 36.3, 30.6, 29.8, 23.8, 23.0, 19.5, 14.1. Data in accordance with the literature.[34]

***tert*-Butyl 2-(cyclopropylmethyl)pyrrolidine-1-carboxylate (68)**

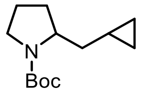

Following **GP3**, **52** (37 mg, 0.1 mmol) gave **68** (72%) as a pale yellow oil. [Petrol:CH_2Cl_2 (20:80)]; FT-IR ν_{max} (film)/cm^{-1} 2967, 2930, 1694, 1454, 1390, 1364, 1259, 1168, 1103, 1017; ^{1}H NMR (500 MHz, $CDCl_3$, rotamers) δ 3.88 (0.4H, br s), 3.80 (0.6H, br s), 3.42–3.33 (2H, m), 2.06–1.73 (4H, m), 1.44 (9H, s), 1.42–1.09 (2H, m), 0.60 (1H, m), 0.41 (2H, m), 0.20–0.00 (2H, m); ^{13}C NMR (126 MHz, $CDCl_3$, rotamers) δ 154.7, 78.9, 57.7, 46.7, 46.2, 39.5, 38.5, 30.7, 30.0, 28.7, 24.0, 23.2, 8.0, 4.9, 4.0; HRMS (HESI): Found MNa^+ 248.1616 $C_{13}H_{23}NO_2Na$ requires 248.1621.

tert-Butyl 2-tetradecylpyrrolidine-1-carboxylate (69)

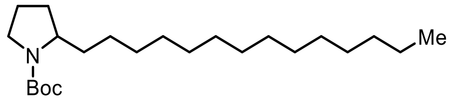

Following **GP3**, **52** (37 mg, 0.1 mmol) gave **69** (57%) as an oil. [Petrol:acetone (90:10)]; FT-IR ν_{max} (film)/cm^{-1} 2960, 2923, 2853, 1697, 1455, 1390, 1364, 1259, 1171, 1099, 1023; ^{1}H NMR (500 MHz, $CDCl_3$) δ 3.75 (0.4H, br s), 3.69 (0.6H, br s), 3.38 (0.8H, br s), 3.29 (1.2H, br s), 1.96–1.69 (3H, m), 1.69–1.56 (3H, m), 1.46 (9H, s), 1.38–1.16 (26H, m), 0.88 (3H, t, *J* = 6.8 Hz); ^{13}C NMR (126 MHz, $CDCl_3$, rotamers) δ 32.1, 29.9, 29.8, 29.7, 29.5, 28.7, 22.8, 14.3; HRMS (HESI): Found MH-*t*-Bu$^+$ 326.3055 $C_{20}H_{40}NO_2$ requires 326.3054. Further deprotection using TFA (1.0 equiv.) in CH_2Cl_2 (0.1 M), at room temperature, 1h, gave the corresponding 2-pentadecylpyrrolidin-1-ium ion. ^{1}H NMR (400 MHz, $CDCl_3$) δ 10.21 (1H, br s), 3.51 (1H, br s), 2.31–1.94 (3H, m), 1.89–1.57 (4H, m), 1.41–1.17 (27H, m), 0.87 (3H, t, *J* = 6.7 Hz). Data in accordance with the literature[35].

1-Methyl-2-tetradecylpyrrolidine (70)

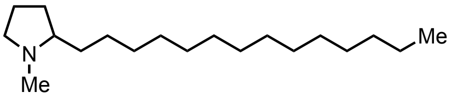

^{1}H NMR (500 MHz, $CDCl_3$) δ 3.07−3.01 (1H, m), 2.29 (3H, s), 2.12 (1H, app dd, *J* = 17.6, 8.5 Hz), 1.97−1.85 (2H, m), 1.80−1.62 (3H, m), 1.46−1.39 (1H, m), 1.32−1.15 (25H, m), 0.87 (3H, t, *J* = 6.8 Hz). Data in accordance with the literature.[46]

***tert*-Butyl 2-Isopropylpyrrolidine-1-carboxylate (71)**

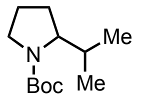

Following **GP3**, **S19** (38 mg, 0.1 mmol) gave **71** (53%) as an oil. ^{1}H NMR (400 MHz, $CDCl_3$, rotamers) δ 3.76–3.32 (2H, m), 3.23–3.12 (1H, m), 2.23–1.63 (5H, m), 1.44 (9H, s), 0.83 (3H, d, J = 7.1 Hz), 0.77 (3H, d, J = 6.9 Hz); ^{13}C NMR (126 MHz, $CDCl_3$, diastereomers and rotamers) δ 154.6, 79.0, 62.3, 47.7, 30.8, 30.2, 28.9, 26.9, 26.2, 24.8, 23.8, 20.0, 17.8. Data in accordance with the literature.

***tert*-Butyl 2-butyl-5-propylpyrrolidine-1-carboxylate (73)**

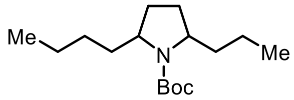

Following **GP3**, **72** (42 mg, 0.1 mmol) gave **73** (70%) as an oil. d.r 1:1. ^{1}H NMR (400 MHz, $CDCl_3$, rotamers) δ 3.80–3.63 (1.4H, m), 3.63–3.52 (0.6H, m), 1.96–1.70 (3H, m), 1.67–1.49 (3H, m), 1.41 (9H, s), 1.35–1.02 (6H, m), 0.93–0.77 (6H, m); ^{13}C NMR (126 MHz, $CDCl_3$, diastereomers and rotamers) δ 154.0, 78.8, 57.8 & 57.7, 34.1 & 33.8, 32.9 & 32.7, 32.0, 29.2 & 29.1, 28.8, 28.7 & 28.6, 27.7, 26.8, 26.7 & 26.6, 22.9, 22.8, 14.4 & 14.3, 14.2. Data in accordance with the literature.[36]

***tert*-Butyl 2-butyl-5-pentylpyrrolidine-1-carboxylate (74)**

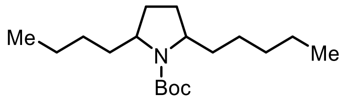

Following **GP3**, **72** (42 mg, 0.1 mmol) gave **74** (70%) as an oil. d.r 1:1. [CH_2Cl_2:Et_2O (95:5)]; FT-IR ν_{max} (film)/cm^{-1} 3342, 2960, 1694, 1668, 1388, 1365, 1258, 1016; ^{1}H NMR (500 MHz, $CDCl_3$, diastereomers and rotamers) δ 3.74–3.67 (1H, m), 3.64–3.57 (1H, m), 2.04–1.76 (3H, m), 1.74–1.52 (2H, m), 1.45 (9H, s), 1.38–1.10 (13H, m), 0.99–0.79 (6H, m); ^{13}C NMR (126 MHz, $CDCl_3$, diastereomers and rotamers) δ 154.0, 78.8, 57.8 & 57.7, 34.1 & 33.8, 32.9 & 32.7, 32.0, 29.2 & 29.1, 28.8, 28.7 & 28.6, 27.7, 26.8, 26.7 & 26.6, 22.9, 22.8, 14.4 & 14.3, 14.2; HRMS (HESI): Found MH–t-Bu^{+} 242.2113 $C_{14}H_{28}NO_2$ requires 242.2115.

tert-Butyl 2-butyl-5-heptylpyrrolidine-1-carboxylate (75)

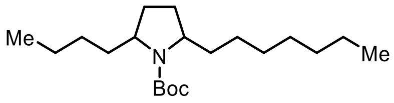

Following **GP3**, **72** (42 mg, 0.1 mmol) gave **75** (70%) as an oil. d.r 1:1; [CH_2Cl_2:Et_2O (95:5)]; FT-IR ν_{max} (film)/cm^{-1} 3387, 2959, 2927, 1694, 1389, 1258, 1173, 1019; ^{1}H NMR (500 MHz, $CDCl_3$, diastereomers and rotamers) δ 3.75–3.67 (1H, m) 3.65–3.56 (1H, m), 2.03–1.76 (3H, m), 1.77–1.60 (2H, m), 1.45 (9H, s), 1.43–1.09 (17H, m), 0.88 (6H, m); ^{13}C NMR (126 MHz, $CDCl_3$, diastereomers and rotamers) δ 153.9, 78.6, 57.6, 34.0 & 33.7, 32.8 & 32.5, 31.8, 29.7 & 29.6, 29.4 & 29.3, 29.1 & 29.0, 28.6, 28.5, 27.6, 26.9 & 26.8, 26.7, 22.7, 22.6, 14.2 & 14.1, 14.1; HRMS (HESI): Found MH–*t*-Bu$^+$ 270.2422 $C_{16}H_{32}NO_2$ requires 270.2425.

2-Butyl-5-propylpyrrolidine (76)

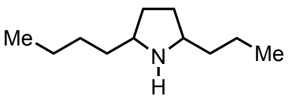

TFA (10 equiv.) was added to a solution of **73** in CH_2Cl_2 (0.1 M) at room temperature. The mixture was stirred for 2 h and NaOH (1 M in H_2O) was added until pH = 14. The layers were separated and the aqueous layer was washed with CH_2Cl_2 (x 4). The combined organic layers were dried ($MgSO_4$) and evaporated to give **76** (quant). ^{1}H NMR (500 MHz, $CDCl_3$) δ 3.66–3.44 (1H, m), 3.07–2.91 (1H, m) 1.99–186 (2H, m), 1.55–1.22 (14H, m), 1.01–0.84 (6H, m), 0.94 (3H, t, *J* = 7.3 Hz), 0.90 (3H, *t*, J = 7.4 Hz). Data in accordance with the literature.[47]

2-Butyl-5-pentylpyrrolidine (77)

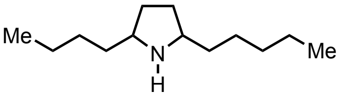

TFA (10 equiv.) was added to a solution of **74** in CH_2Cl_2 (0.1 M) at room temperature. The mixture was stirred for 2 h and NaOH (1 M in H_2O) was added until pH = 14. The layers were separated and the aqueous layer was washed with CH_2Cl_2 (x 4). The combined organic layers were dried ($MgSO_4$) and evaporated to give **77** (quant). ^{1}H NMR (500 MHz, $CDCl_3$) δ 3.44–2.99 (2H, m), 2.14 (1H, br s), 2.12–1.84 (2H, m), 1.61–1.44 (16H, m), 0.91 (3H, *t*, J = 7.4 Hz), 0.86 (3H, *t*, J = 7.1 Hz). Data in accordance with the literature.[48]

2-Butyl-5-heptylpyrrolidine (78)

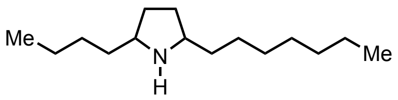

TFA (10 equiv.) was added to a solution of **75** in CH_2Cl_2 (0.1 M) at room temperature. The mixture was stirred for 2 h and NaOH (1 M in H_2O) was added until pH = 14. The layers were separated and the aqueous layer was washed with CH_2Cl_2 (x 4). The combined organic layers were dried ($MgSO_4$) and evaporated to give **78** (quant). ^{1}H NMR (500 MHz, $CDCl_3$) δ 3.29–3.19 (1H, m), 3.06–2.99 (1H), 2.05–1.89 (2H, m), 1.71–1.55 (2H, m), 1.48–1.21 (18H, m), 0.91–0.82 (6H, m), 0.88 (3H, *t*, J = 7.5 Hz), 0.86 (3H, *t*, J = 7.4 Hz). Data in accordance with the literature.[49]

8 Computational Studies

8.1 Computational Methods

Density functional theory (DFT)[37] calculations were performed using Gaussian 09 (revision E.01)[38] and the Gaussview[39] was used to generate input geometries and visualize output structures. Regarding geometry optimizations and frequency calculations for the nitrogen radicals addition and subsequent reductive elimination, B3LYP functional[40–43] was used with the UB3LYP/6-31G(d)-LANL2DZ (for Ni and Br) basis set.[44] All stationary points were characterized as minima or transitions states based on normal vibrational mode analysis. Thermal corrections were computed from unscaled frequencies, assuming a standard state of 298.15 K and 1 atm. For substrates having more than one conformations, low energy conformation of the transition state could possibly be different from the low energy ground state.[45] The structures described herein are the lowest energy-optimized conformers.

8.2 Activation Energy ($\Delta G^{\ddagger}$) and Reaction Energy (ΔG) for Radical Additions and Reductive Elimination Reactions

DFT Method: UB3LYP/6-31G(d)-LANL2DZ (for Ni and Br) [values are in Kcal mol^{-1}]

**No.**	**Radical Additions and corresponding Reductive Elimination Reactions**	**$\Delta G^{\ddagger}$**	**ΔG**
1	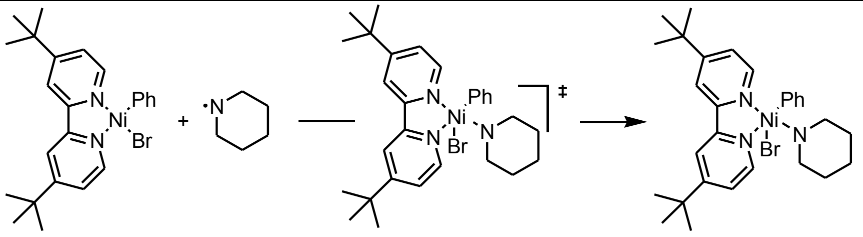	6.7	5.0
2	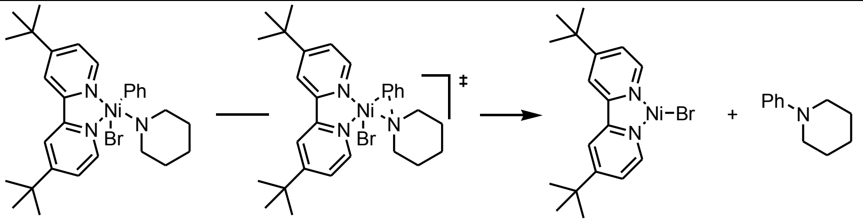	2.1	-58.6
3	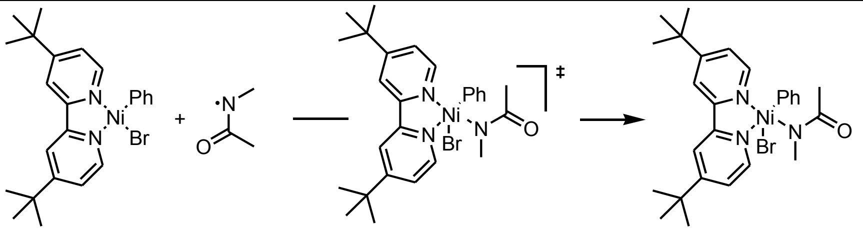	11.7	-2.6
4	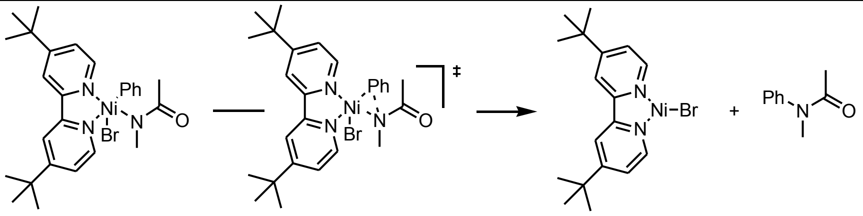	10.3	-58.9

Computed Energies [values are in Hartree]

No.	Species	Total Electronic Energy	Sum of Electronic and Zero-point Energies	Sum of Electronic and Thermal Enthalpies	Gibbs Free Energy
1	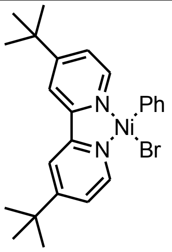	-1224.0875973	-1223.608936	-1223.579239	-1223.671024
2	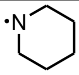	-251.2503264	-251.104655	-251.098082	-251.134045
3	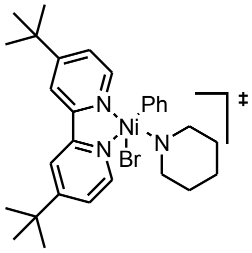	-1475.3489696	-1474.723617	-1474.687127	-1474.794432
4	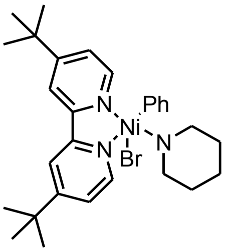	-1475.3493639	-1474.724244	-1474.686747	-1474.797033
5	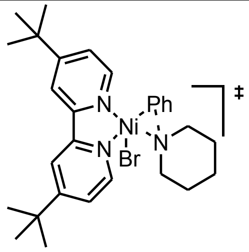	-1475.3509824	-1474.724436	-1474.688343	-1474.793710
6	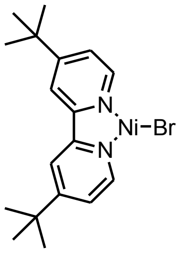	-992.4688836	-992.081943	-992.057176	-992.139638
7	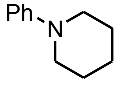	-482.9557153	-482.715047	-482.704079	-482.750721
8	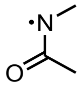	-247.851032	-247.763155	-247.755772	-247.794013

9	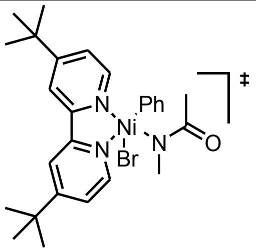	-1471.9443233	-1471.375827	-1471.339200	-1471.446345
10	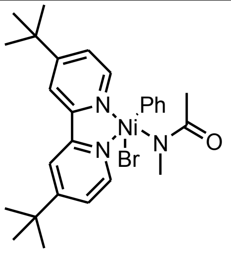	-1471.9684674	-1471.398339	-1471.361257	-1471.469115
11	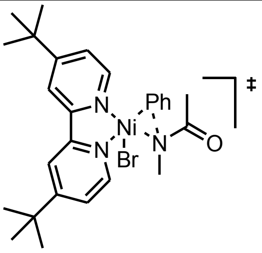	-1471.9539625	-1471.383700	-1471.347648	-1471.452672
12	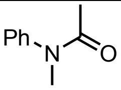	-479.5683878	-479.385072	-479.373311	-479.423381

Optimized Structures and Cartesian Coordinates

No.	Species	Optimized Structure
1	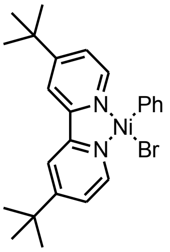	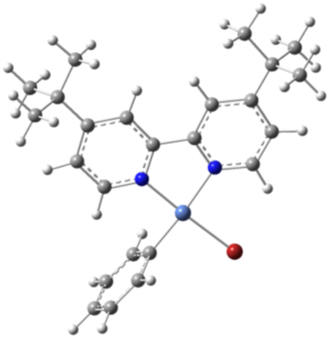

Cartesian Coordinates

```
C     1.40980000  -0.28870600  -0.00010600
C     1.19690000  -2.58919800  -0.00123600
C     2.57593900  -2.75776900  -0.00061400
C     3.42817000  -1.64319900   0.00043100
C     2.80491000  -0.39069600   0.00068000
C     0.67519900   0.99476700  -0.00019000
C    -1.40501300   2.00737300  -0.00094300
C    -0.83522300   3.27290500  -0.00107700
C     0.55894400   3.42706700  -0.00068200
C     1.30354100   2.24388400  -0.00026400
H     0.50919700  -3.42922600  -0.00192800
H     2.97281300  -3.76730900  -0.00090200
H    -2.47871700   1.87280000  -0.00119000
H    -1.49699000   4.13234900  -0.00150600
N     0.61544400  -1.38073100  -0.00097000
```

N	-0.67630200	0.87668900	-0.00044700
Ni	-1.37423200	-0.96468600	-0.00052800
C	-3.85970400	-0.16363700	1.20532700
C	-5.16073500	0.35764300	-1.20318900
C	-5.15795000	0.35963700	1.20805000
H	-3.37538500	-0.37769900	2.15606400
C	-5.81184800	0.62651000	0.00296500
H	-5.66551700	0.54875900	-2.14832700
H	-5.66053000	0.55232300	2.15404200
Br	-2.11290700	-3.22262200	-0.00134700
C	-3.86251000	-0.16568600	-1.20263300
H	-3.38045800	-0.38150600	-2.15411400
C	-3.18842000	-0.42369100	0.00077800
H	2.38405400	2.28543200	-0.00007100
H	3.40263400	0.51063100	0.00151200
H	-6.82196200	1.02920800	0.00379700
C	1.19497800	4.82385300	-0.00074400
C	4.95291900	-1.82359900	0.00117300
C	5.70256900	-0.47798400	0.00270600
C	5.36667900	-2.61617500	1.26480700
C	5.36811900	-2.61410400	-1.26327100
C	0.73276000	5.58918600	1.26302100
C	0.73338600	5.58883200	-1.26495000
C	2.73419000	4.76693900	-0.00035500
H	4.89959200	-3.60287400	-1.29965500
H	5.08752400	-2.07564300	-2.17553300
H	6.45457000	-2.75914000	-1.27452900
H	4.89825100	-3.60506300	1.29893600
H	6.45313800	-2.76102600	1.27713500
H	5.08486500	-2.07927100	2.17761400
H	5.47215700	0.12057300	-0.88637300
H	5.47107100	0.11917200	0.89245100
H	6.78263800	-0.65994400	0.00321900
H	3.12525400	4.25901300	-0.88957400
H	3.13821400	5.78487800	-0.00035700
H	3.12480400	4.25918100	0.88915900
H	1.04772400	5.07144800	2.17605600
H	1.17148200	6.59363600	1.27342400
H	-0.35568200	5.70096400	1.29866800
H	-0.35503000	5.70068400	-1.30111700
H	1.17219600	6.59324200	-1.27547200
H	1.04871800	5.07078100	-2.17768200

2	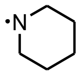	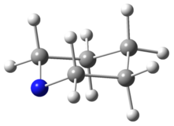

Cartesian Coordinates

C	-1.19198700	-0.81316100	0.19521500
C	1.19198700	-0.81316100	0.19521500
C	-1.25982500	0.67695800	-0.22220500
H	-1.19912500	-0.86464500	1.30034300
H	-2.07453600	-1.35181200	-0.16838800
C	1.25982400	0.67695900	-0.22220500
H	1.19912500	-0.86464500	1.30034300
H	2.07453600	-1.35181100	-0.16838800
C	0.00000000	1.42258300	0.23654300
H	-2.16703900	1.13037800	0.19568400
H	-1.34612400	0.73067500	-1.31524100
H	2.16703900	1.13037900	0.19568300
H	1.34612300	0.73067500	-1.31524100
H	0.00000000	2.44944100	-0.15057100
H	0.00000000	1.49771100	1.33359600
N	0.00000000	-1.44820200	-0.32902800

3	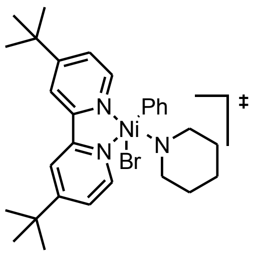	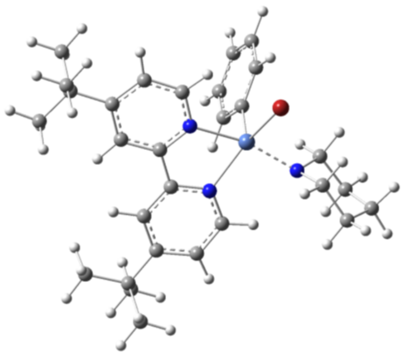

Cartesian Coordinates

C	-1.70732400	-0.27110700	-0.32754000
C	-1.12783300	-2.49303200	-0.58360500
C	-2.45769800	-2.89191800	-0.53053600
C	-3.47534000	-1.94057300	-0.36332500
C	-3.06515200	-0.60721300	-0.26489800
C	-1.20883700	1.12532700	-0.22568100
C	0.66013900	2.48075200	-0.13512200
C	-0.11287800	3.62987500	-0.03357500
C	-1.51423900	3.53296200	-0.03302000
C	-2.04765800	2.24404300	-0.13241600
H	-0.31855200	-3.20079800	-0.72907500
H	-2.68610300	-3.94850000	-0.62030800
H	1.74561100	2.51185500	-0.14256400
H	0.38434900	4.59111100	0.04095500
N	-0.75469300	-1.21085100	-0.48146400
N	0.13167000	1.25550900	-0.23007000
Ni	1.27328400	-0.58183100	-0.30739300
Br	2.09375700	-2.45544900	-1.85421200
H	-3.11976100	2.10398500	-0.14158700
H	-3.79984000	0.17495000	-0.13137600
C	-2.38297100	4.79541600	0.07041700
C	-4.94700100	-2.37398600	-0.29476800
C	-5.90168500	-1.18000800	-0.10448400
C	-5.32356600	-3.09669100	-1.61062200
C	-5.13265100	-3.34273000	0.89807400
C	-2.07321200	5.72116500	-1.13090000
C	-2.04872800	5.53284100	1.38982900
C	-3.88947200	4.47446700	0.06110800
H	-4.51168000	-4.23864600	0.79729800
H	-4.86985500	-2.85756000	1.84478100
H	-6.17829300	-3.66650400	0.95929700
H	-4.70427600	-3.98313000	-1.78145800
H	-6.36977200	-3.42220400	-1.57366400
H	-5.20421800	-2.43262600	-2.47418100
H	-5.70232600	-0.64118500	0.82920000
H	-5.83838700	-0.46770800	-0.93557100
H	-6.93519700	-1.54022600	-0.06055600
H	-4.17851000	3.83967900	0.90667600
H	-4.46188200	5.40497000	0.13900000
H	-4.19674600	3.97747300	-0.86626500
H	-2.30749400	5.22706900	-2.08056200
H	-2.67552200	6.63474700	-1.06554700
H	-1.01971000	6.01817100	-1.15771800
H	-0.99742700	5.83525500	1.43535600
H	-2.65987600	6.43858100	1.47716100
H	-2.25419000	4.89886400	2.25964800
C	1.35588900	-1.13734000	1.62436200
C	0.93713600	-0.29919000	2.66857900
C	1.79706700	-2.42763900	1.95593000
C	0.95552000	-0.73099700	4.00138800
H	0.59644900	0.71433700	2.45760000
C	1.81640000	-2.86581300	3.28580200
H	2.13602900	-3.09886300	1.16956200
C	1.39566900	-2.01857100	4.31354300
H	0.62731400	-0.06043200	4.79423700
H	2.16487400	-3.87066400	3.51849700
H	1.41253900	-2.35747600	5.34696000
C	4.02555900	0.32234500	0.73543900
C	3.77487000	0.44287500	-1.65157700

C	4.98856000	1.53276300	0.74814600
H	4.61659800	-0.60428100	0.63578900
H	3.47436400	0.26101700	1.67404900
C	4.69424800	1.68301200	-1.74008500
H	4.38137700	-0.46574200	-1.77737100
H	3.03935900	0.45175000	-2.45960300
C	5.71184900	1.68599500	-0.59401600
H	5.70005200	1.40474000	1.57330800
H	4.41051000	2.44227400	0.96434600
H	5.19293600	1.67656300	-2.71718300
H	4.08140300	2.59459000	-1.69913400
H	6.31110800	2.60566000	-0.60828300
H	6.41103600	0.84869000	-0.72923900
N	3.07877800	0.42248800	-0.36657200

4

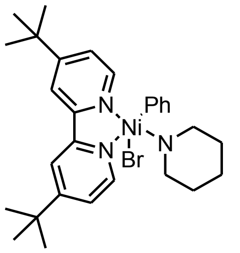

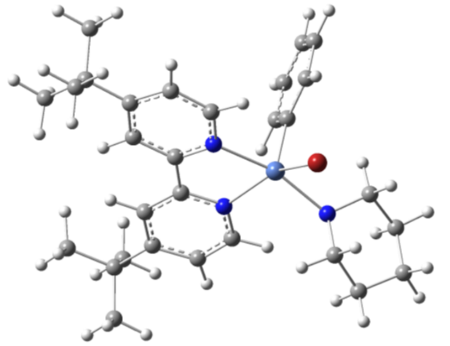

Cartesian Coordinates

C	-1.72209300	-0.30961300	-0.35100200
C	-1.12821200	-2.53005900	-0.59124700
C	-2.45688400	-2.93541400	-0.55995300
C	-3.48122100	-1.98767700	-0.41188700
C	-3.07924100	-0.65175600	-0.31154700
C	-1.22929900	1.08852000	-0.24173200
C	0.63205000	2.45213700	-0.11207500
C	-0.14714100	3.59699400	-0.01306700
C	-1.54798400	3.49387400	-0.03514000
C	-2.07367100	2.20322000	-0.14983400
H	-0.31251900	-3.23426400	-0.72058400
H	-2.67958200	-3.99313000	-0.65120100
H	1.71715100	2.49108400	-0.09003900
H	0.34466800	4.55941900	0.07967200
N	-0.76322400	-1.24628700	-0.48355400
N	0.11096800	1.22561300	-0.23143600
Ni	1.24963000	-0.59001400	-0.25545700
Br	2.21354100	-2.47957100	-1.67692400
H	-3.14494600	2.05806000	-0.16993600
H	-3.82034000	0.12694100	-0.19410100
C	-2.42353700	4.75190300	0.06405300
C	-4.95187000	-2.42774700	-0.36339000
C	-5.91547800	-1.23673200	-0.20166200
C	-5.30387600	-3.16714700	-1.67671300
C	-5.15174500	-3.38404700	0.83724800
C	-2.10313200	5.68411500	-1.12949500
C	-2.10905000	5.48491900	1.39069600
C	-3.92832900	4.42431400	0.03480700
H	-4.52518400	-4.27809100	0.75602100
H	-4.90573600	-2.88721400	1.78238400
H	-6.19673700	-3.71206100	0.88604400
H	-4.67744800	-4.05213800	-1.82757500
H	-6.34876700	-3.49801000	-1.65254300
H	-5.17450400	-2.51210500	-2.54572800
H	-5.73497800	-0.68794500	0.72998500
H	-5.84112800	-0.53222600	-1.03849300
H	-6.94777200	-1.60183200	-0.17202400
H	-4.22452900	3.78403800	0.87369600
H	-4.50579200	5.35188800	0.11045900
H	-4.22220800	3.93067000	-0.89870400
H	-2.32445400	5.19328800	-2.08400500
H	-2.70946100	6.59526300	-1.06741500
H	-1.05041300	5.98490800	-1.14257400
H	-1.05991800	5.79223500	1.45060200
H	-2.72556000	6.38736800	1.47448600

H	-2.32196100	4.84594900	2.25498900
C	1.32727300	-0.99574500	1.72571300
C	1.13682900	-0.01762100	2.71499300
C	1.56190900	-2.31190900	2.15986700
C	1.17386400	-0.33285500	4.07936100
H	0.96022000	1.02031700	2.43182900
C	1.59953900	-2.63796800	3.52098000
H	1.73328200	-3.09733200	1.42561900
C	1.40406500	-1.64809700	4.48707700
H	1.02412500	0.44888300	4.82295300
H	1.78700900	-3.66601600	3.82706700
H	1.43409800	-1.89843200	5.54530200
C	4.19914800	-0.03053700	0.37046700
C	3.50749100	0.73357700	-1.81054700
C	5.24190600	1.10455200	0.49041800
H	4.67685600	-0.91561900	-0.07936800
H	3.81774700	-0.31551500	1.35198200
C	4.50847200	1.91231200	-1.77266000
H	3.98433600	-0.13303400	-2.28972500
H	2.62708800	0.99442000	-2.40752900
C	5.71529000	1.56719000	-0.89201200
H	6.08017100	0.74552300	1.09962300
H	4.79067100	1.94751600	1.03158100
H	4.81527000	2.14353100	-2.80018100
H	4.00576000	2.80714900	-1.37913300
H	6.38618500	2.43128700	-0.80291800
H	6.29241900	0.76260200	-1.36879200
N	3.07837500	0.38664000	-0.45884800

5

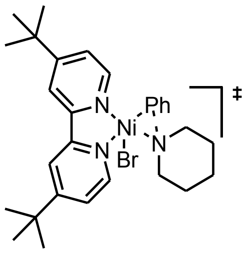

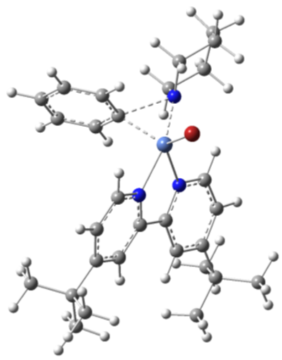

Cartesian Coordinates

C	-1.62970800	-0.45712700	-0.14994100
C	-0.65429100	-2.48481000	-0.66857600
C	-1.85818700	-3.15751500	-0.49962600
C	-3.01290300	-2.45554900	-0.11952800
C	-2.87082700	-1.07426800	0.04847600
C	-1.41779400	1.00978500	-0.03999600
C	0.12501100	2.72449500	-0.05698600
C	-0.86959900	3.69193600	0.00110600
C	-2.21921400	3.30698000	0.04750200
C	-2.47098600	1.93181600	0.01849100
H	0.25261500	-2.99526800	-0.97436400
H	-1.88178000	-4.22949600	-0.66365200
H	1.17226800	3.00122500	-0.11247800
H	-0.58212200	4.73793700	0.00031300
N	-0.53527200	-1.16381700	-0.48635200
N	-0.13014900	1.40928800	-0.06017300
Ni	1.32098400	-0.18070300	-0.44177500
Br	2.04834300	-1.31536900	-2.63160800
H	-3.48965900	1.56855200	0.01374800
H	-3.71966000	-0.47315500	0.34554100
C	-3.33173400	4.36406200	0.10271600
C	-4.34273700	-3.19506800	0.08909400
C	-5.48195300	-2.24693500	0.50888200
C	-4.74694000	-3.88920500	-1.23390600
C	-4.16050700	-4.26042900	1.19717400
C	-3.23911800	5.26144000	-1.15522500
C	-3.14170800	5.23076200	1.37113400
C	-4.73685600	3.73502900	0.14939400
H	-3.38927100	-4.99251400	0.93700300
H	-3.87514300	-3.79569800	2.14773700
H	-5.09926200	-4.80487200	1.35233900

H	-3.99370400	-4.61218200	-1.56301900
H	-5.69199700	-4.42875900	-1.10106100
H	-4.88281800	-3.15752700	-2.03826900
H	-5.26828700	-1.74494900	1.45975900
H	-5.67822900	-1.48143100	-0.25081900
H	-6.40550200	-2.82070700	0.64169800
H	-4.87300200	3.10171400	1.03371200
H	-5.49111900	4.52787600	0.19439600
H	-4.94636400	3.13413200	-0.74295600
H	-2.27472800	5.77532700	-1.22171300
H	-3.37002700	4.67281200	-2.07011300
H	-4.02375500	6.02634000	-1.12752400
H	-2.17266000	5.74038200	1.37797000
H	-3.92215400	5.99888400	1.42022900
H	-3.20778100	4.62136100	2.27964600
C	2.03160900	-1.04753000	1.19960100
C	1.43261000	-0.74220100	2.42792800
C	2.72332800	-2.25571300	1.04763600
C	1.48782800	-1.65860900	3.48275200
H	0.90740500	0.19921100	2.56781900
C	2.77484700	-3.16675000	2.10770400
H	3.19651100	-2.49695300	0.10111000
C	2.15939800	-2.87371400	3.32721800
H	1.00613300	-1.41769100	4.42814800
H	3.30354800	-4.10825900	1.97598700
H	2.20949900	-3.58107800	4.15085900
C	3.11824000	1.56877700	1.30131000
C	4.28694300	0.12008400	-0.22311700
C	3.95452200	2.79192000	0.87269100
H	3.61006200	1.09388900	2.16937100
H	2.12849600	1.89296800	1.63580700
C	5.17204400	1.27637200	-0.72532900
H	4.79661800	-0.38075500	0.62040700
H	4.14255200	-0.60642100	-1.02409700
C	5.33036900	2.35379300	0.35447400
H	4.05378600	3.47622200	1.72646000
H	3.42329000	3.33510500	0.07821500
H	6.14979800	0.87308900	-1.01979400
H	4.71359700	1.70610000	-1.62483600
H	5.89004500	3.21603600	-0.03048000
H	5.91507400	1.94556700	1.19213100
N	2.98228300	0.63590600	0.18830000

6

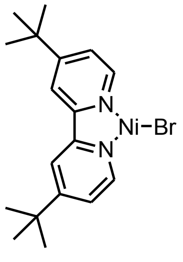

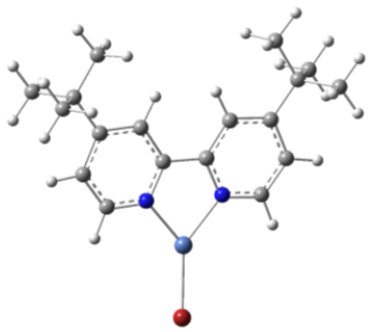

Cartesian Coordinates

C	-0.41410100	-0.74699500	0.00013700
C	0.93824000	-2.62530200	0.00058100
C	-0.16020000	-3.47365300	0.00042000
C	-1.46190800	-2.94333200	0.00006800
C	-1.56145900	-1.54814400	-0.00006500
C	-0.43697000	0.73642500	0.00002200
C	0.85664200	2.65562300	-0.00018100
C	-0.26730500	3.46977900	-0.00016200
C	-1.55205200	2.89959000	-0.00002600
C	-1.60839300	1.50200700	0.00006500
H	1.95263100	-3.01246900	0.00082700
H	0.00958900	-4.54499400	0.00055800
H	1.85851300	3.07410100	-0.00027500
H	-0.13003500	4.54585000	-0.00024200
N	0.82599400	-1.28745000	0.00043200
N	0.78584900	1.31486100	-0.00008700
Ni	2.34277200	0.03810600	0.00014000

H	-2.56664600	0.99992700	0.00020600
H	-2.53465900	-1.07581600	-0.00036800
C	-2.80246500	3.79104400	0.00003700
C	-2.68397100	-3.87354600	-0.00016500
C	-4.01319400	-3.09530900	-0.00036400
C	-2.64033100	-4.76650900	-1.26393000
C	-2.64075000	-4.76658100	1.26356200
C	-2.78693400	4.68498600	-1.26373200
C	-2.78671600	4.68510900	1.26371600
C	-4.10652500	2.97140900	0.00019100
H	-1.73509300	-5.38063200	1.30030800
H	-2.67338300	-4.16097100	2.17623200
H	-3.50261000	-5.44390200	1.27370800
H	-1.73470400	-5.38061900	-1.30037500
H	-3.50223500	-5.44376900	-1.27444000
H	-2.67258100	-4.16084100	-2.17657400
H	-4.11796000	-2.46306000	0.88898700
H	-4.11756700	-2.46285000	-0.88961200
H	-4.85058600	-3.80123900	-0.00062900
H	-4.19077300	2.33604000	0.88951200
H	-4.96574200	3.65050600	0.00022500
H	-4.19092000	2.33594200	-0.88904700
H	-2.79933000	4.07867900	-2.17642700
H	-3.66987600	5.33459400	-1.27472900
H	-1.90132600	5.32768800	-1.29961600
H	-1.90111200	5.32783100	1.29937500
H	-3.66966700	5.33470100	1.27481500
H	-2.79893000	4.07888800	2.17647100
Br	4.69413800	0.06594200	-0.00018000

7	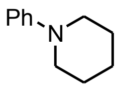	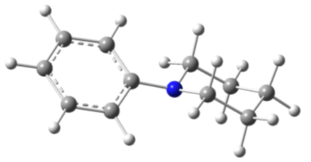

Cartesian Coordinates

C	1.26615900	1.25627200	-0.09921400
C	1.24579800	-1.06579400	0.63849700
C	2.69953500	1.10534300	-0.62422600
H	1.29337000	1.67312300	0.92741300
H	0.72190500	1.97012000	-0.72466400
C	2.67234800	-1.29112800	0.13039300
H	1.27899600	-0.78146500	1.70869400
H	0.67244500	-1.99242200	0.57555300
C	3.46671400	0.02081400	0.14137700
H	3.21202200	2.07259000	-0.54781500
H	2.65554000	0.84280400	-1.68922000
H	3.16241300	-2.04903100	0.75461600
H	2.62607100	-1.68793300	-0.89234200
H	4.46425000	-0.12496000	-0.29077600
H	3.61670300	0.34548900	1.18173200
N	0.56054300	-0.02315100	-0.14037900
C	-0.85197100	0.00077900	-0.06021800
C	-1.57463800	1.15350800	0.29620500
C	-1.58466800	-1.15919500	-0.38616700
C	-2.97148100	1.14348000	0.31879600
H	-1.05254100	2.06257000	0.57317700
C	-2.97446400	-1.16493300	-0.34562100
H	-1.05492800	-2.05243800	-0.70379800
C	-3.68333800	-0.01189800	0.00549300
H	-3.50118800	2.05108000	0.59820300
H	-3.50948600	-2.07434300	-0.60818800
H	-4.76933300	-0.01659900	0.02816700

8	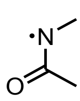	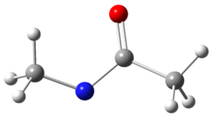

Cartesian Coordinates

C	0.46691200	0.14545300	-0.03392800
O	0.37244300	1.37211000	-0.03905100
N	-0.63296100	-0.65709900	-0.29608000
C	1.77967300	-0.59073700	0.10317000

C	-1.91897700	-0.18131900	0.14210400
H	-2.35529700	-0.95348100	0.79404700
H	-1.87948500	0.77799700	0.67003300
H	-2.59012800	-0.10490400	-0.72410100
H	2.08945300	-0.97452500	-0.87577100
H	2.54481500	0.08995200	0.48148900
H	1.67617800	-1.45261300	0.77119200

9	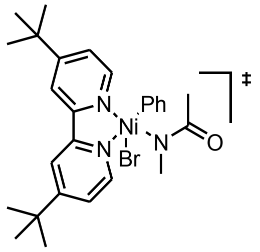	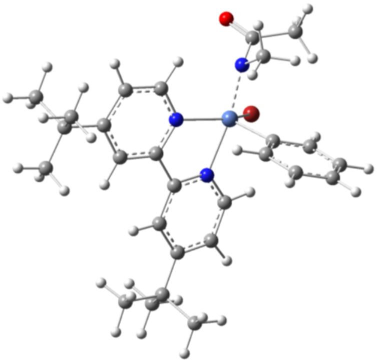

Cartesian Coordinates

C	-1.33500400	-0.71630900	-0.21065100
C	0.15516500	-2.45038400	-0.55879600
C	-0.86597700	-3.39145100	-0.53406600
C	-2.19491800	-2.98841500	-0.33290000
C	-2.40697500	-1.61543200	-0.17112200
C	-1.49791400	0.75467300	-0.07589700
C	-0.39209800	2.78424000	0.00525400
C	-1.58842300	3.48806900	0.06702800
C	-2.81098600	2.79675400	0.06040300
C	-2.73849700	1.40130200	-0.01927800
H	1.18902300	-2.72732700	-0.73225900
H	-0.60984500	-4.43597900	-0.67460200
H	0.57506000	3.27648900	-0.02410000
H	-1.55646100	4.57153600	0.10838300
N	-0.06871000	-1.13961200	-0.39775700
N	-0.34518400	1.44838100	-0.04820900
Ni	1.47229400	0.27905500	-0.25178600
Br	2.82128600	-0.91106800	-2.07798800
H	-3.64631700	0.81474600	-0.05375000
H	-3.40766500	-1.23797100	-0.01085100
C	-4.14018800	3.56431400	0.11919700
C	-3.32912000	-4.02320400	-0.30299000
C	-4.70740900	-3.37716500	-0.06808600
C	-3.36363000	-4.77121700	-1.65779800
C	-3.06182500	-5.03402600	0.83849600
C	-4.23332400	4.50512400	-1.10669800
C	-4.18139700	4.40393400	1.41897600
C	-5.36129800	2.62550200	0.10730700
H	-2.11136200	-5.56036700	0.70469400
H	-3.03367300	-4.53193400	1.81215400
H	-3.85862000	-5.78631800	0.86621900
H	-2.42239300	-5.29152200	-1.86183800
H	-4.16437500	-5.51984800	-1.65145600
H	-3.55102700	-4.07946400	-2.48667600
H	-4.75599900	-2.85316500	0.89360800
H	-4.96844800	-2.66866400	-0.86279100
H	-5.47876000	-4.15465100	-0.05685300
H	-5.36326000	1.94509100	0.96686600
H	-6.27996700	3.21961800	0.15748500
H	-5.40849900	2.02629000	-0.80929100
H	-4.20420500	3.93727900	-2.04333500
H	-5.17465200	5.06603300	-1.07776400
H	-3.41363000	5.23032600	-1.12838600
H	-3.35765100	5.12345200	1.46719700
H	-5.11972400	4.96812100	1.47079500
H	-4.12117000	3.76306000	2.30596400
C	1.98221400	-0.44397900	1.51662900
C	1.20296000	-0.15957400	2.64136300
C	3.06838200	-1.31560600	1.62991700
C	1.50783900	-0.74328300	3.87728700
H	0.35508100	0.51925300	2.57138300
C	3.37162600	-1.89789900	2.86804900

H	3.67041500	-1.55098000	0.75673200
C	2.59481300	-1.61239000	3.99248400
H	0.89478200	-0.51671300	4.74742100
H	4.21956700	-2.57486600	2.94880700
H	2.83391800	-2.06405700	4.95207700
C	3.00663200	2.37417100	1.30271200
C	3.65166300	2.05215500	-0.98314100
N	2.60563000	1.87647600	-0.01344300
H	3.33144600	3.42235100	1.20807200
H	2.15677500	2.33801200	1.98671900
H	3.83129900	1.80982700	1.76115800
O	3.40008400	2.70131000	-1.97846000
C	5.04399000	1.53041900	-0.68208200
H	5.60606800	2.28857800	-0.12022500
H	5.01413500	0.61165900	-0.09233300
H	5.56025800	1.34683500	-1.62568100

10

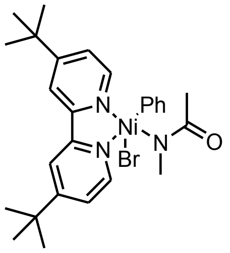

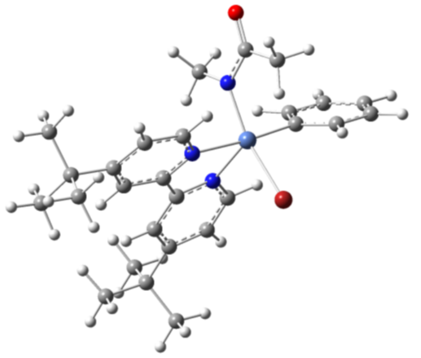

Cartesian Coordinates

C	-1.55022400	-0.46585000	0.03975800
C	-0.84633400	-2.62019000	-0.39589800
C	-2.14190900	-3.11286300	-0.31907200
C	-3.20724200	-2.24493500	-0.02830200
C	-2.87769400	-0.89864500	0.15415800
C	-1.13442400	0.95117400	0.18654100
C	0.63695400	2.42756000	0.32266700
C	-0.21515800	3.52343600	0.35006500
C	-1.60445900	3.33725500	0.28987000
C	-2.04576500	2.01331200	0.20228800
H	-0.00701900	-3.27686900	-0.59965300
H	-2.30725900	-4.17296800	-0.47734500
H	1.71333900	2.55296500	0.34443300
H	0.21805300	4.51618900	0.40413300
N	-0.55321800	-1.32759300	-0.22397000
N	0.19656000	1.16403200	0.25858900
Ni	1.40785800	-0.54798500	-0.16165600
C	4.23157700	-0.13164300	-0.84174000
C	4.78427500	1.54372300	1.32681200
C	5.50692300	0.42255800	-0.67970300
H	4.02691900	-0.75670100	-1.70290100
C	5.78605100	1.26462600	0.39767000
H	4.99141800	2.18115300	2.18339200
H	6.27833000	0.19911400	-1.41297100
Br	1.31792000	-0.19825000	-2.62084500
C	3.51030300	0.98009500	1.17954100
H	2.75929600	1.17743100	1.93801400
C	3.22819700	0.16379900	0.08315800
H	-3.10240300	1.79995100	0.11784200
H	-3.64897200	-0.18114000	0.39888700
H	6.77789700	1.69297000	0.51588600
C	-2.55585800	4.54192400	0.30119500
C	-4.64216200	-2.78087900	0.08156700
C	-5.65765000	-1.67248400	0.41703900
C	-5.04551700	-3.42145100	-1.26870000
C	-4.69901300	-3.85012200	1.19947700
C	-2.23613600	5.44783500	-0.91282300
C	-2.34383400	5.33769600	1.61186300
C	-4.03533300	4.12149400	0.21954200
H	-4.02823900	-4.69128500	0.99726500
H	-4.41896900	-3.42310000	2.16906300
H	-5.71651600	-4.24894100	1.28470300
H	-4.38373900	-4.24905500	-1.54425300

H	-6.06551400	-3.81771900	-1.20439000
H	-5.01700100	-2.68499500	-2.07959300
H	-5.44118900	-1.19609300	1.38030200
H	-5.68619400	-0.89602900	-0.35611600
H	-6.66153500	-2.10505600	0.48411500
H	-4.32958700	3.49088700	1.06669400
H	-4.67064700	5.01334600	0.23919000
H	-4.25704000	3.58185200	-0.70839700
H	-2.37039400	4.90604700	-1.85559600
H	-2.90647500	6.31515300	-0.91961700
H	-1.20778200	5.82234700	-0.88518400
H	-1.31597200	5.70251800	1.70753200
H	-3.00916300	6.20851900	1.63361800
H	-2.56477400	4.71994800	2.48975200
N	1.70752500	-1.53541200	1.47659900
C	2.49937500	-2.64365000	1.52578600
C	1.33672300	-0.97302700	2.76605800
H	2.21102900	-0.66271600	3.35422200
H	0.67933200	-0.11230700	2.61376600
H	0.80112500	-1.72080700	3.36398600
O	2.79290800	-3.20432600	2.58598800
C	2.97795100	-3.21742800	0.19718900
H	2.78404700	-4.29498000	0.18760600
H	2.51078800	-2.76395800	-0.68678400
H	4.06052100	-3.07583500	0.11188600

11

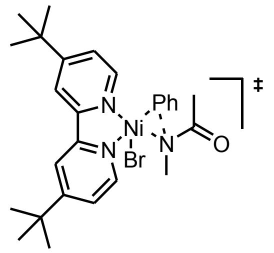

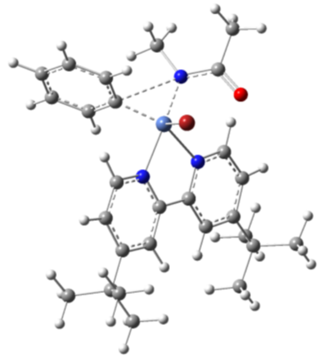

Cartesian Coordinates

C	1.16776900	0.89212500	0.09500000
C	-0.62524800	2.35115500	0.00807700
C	0.21340300	3.44995700	-0.09927100
C	1.60546200	3.27727300	-0.12657300
C	2.06369600	1.96119000	-0.02745400
C	1.60286200	-0.52161600	0.18033800
C	0.91735800	-2.72344700	0.18058500
C	2.22413200	-3.17510500	0.32250800
C	3.28540400	-2.26076100	0.39629100
C	2.94084400	-0.90713300	0.32602600
H	-1.70074700	2.46472500	-0.00091300
H	-0.23460400	4.43412300	-0.17972400
H	0.08629500	-3.40736100	0.09005300
H	2.40019600	-4.24449300	0.36768800
N	-0.16716400	1.09317300	0.11528100
N	0.60633500	-1.42324100	0.10786800
Ni	-1.39573000	-0.55104800	0.03747900
Br	-2.02056700	0.27415300	-2.34086400
H	3.70892500	-0.14894300	0.39345700
H	3.12380100	1.75346600	-0.06608100
C	4.73245200	-2.75169400	0.54808700
C	2.54066000	4.48551400	-0.27406100
C	4.02593700	4.07877300	-0.29071400
C	2.21720100	5.21336200	-1.60154600
C	2.30960300	5.45067700	0.91398000
C	5.08766900	-3.65367700	-0.65881400
C	4.85537700	-3.56751300	1.85766300
C	5.74311200	-1.59082600	0.60208500
H	1.27607500	5.80965200	0.95487400
H	2.53470300	4.96324500	1.86947300
H	2.96245100	6.32583000	0.81705900
H	1.18257700	5.56958000	-1.63144000
H	2.87379400	6.08318400	-1.71952100
H	2.36856600	4.55189800	-2.46159600

H	4.32530400	3.58059300	0.63888800
H	4.25821800	3.41435900	-1.13084900
H	4.64916800	4.97289800	-0.39878800
H	5.56095000	-0.92948800	1.45717600
H	6.75670800	-1.99199200	0.70821300
H	5.72318600	-0.98806400	-0.31319800
H	4.42819200	-4.52462000	-0.72716200
H	5.00995900	-3.10026100	-1.60127800
H	6.11633400	-4.02028500	-0.56253100
H	4.18832100	-4.43566700	1.86412100
H	5.88149000	-3.93474100	1.97568700
H	4.61301800	-2.95099900	2.73084500
C	-2.77191200	0.39732100	0.98307500
C	-2.45007200	0.65289100	2.31837100
C	-3.98519300	0.82474400	0.44790900
C	-3.35395600	1.35188500	3.12844800
H	-1.50513900	0.31806200	2.74207400
C	-4.88073700	1.52624400	1.26593100
H	-4.22572000	0.62422000	-0.58949700
C	-4.57032400	1.79007200	2.60155100
H	-3.10073700	1.55180300	4.16709300
H	-5.82783700	1.86230200	0.85019400
H	-5.27198700	2.33354400	3.22902300
N	-2.40832700	-2.18622100	0.27844500
C	-3.51815800	-2.38550700	1.20670300
H	-4.48445000	-2.02279800	0.82585500
H	-3.62883400	-3.44950600	1.44816500
H	-3.31849100	-1.86294900	2.14279100
C	-2.29122700	-3.03962800	-0.78815600
C	-3.34661900	-4.13540600	-0.97658600
H	-3.28682000	-4.89710500	-0.18921100
H	-4.36528500	-3.73223500	-0.96717100
H	-3.15253900	-4.61164900	-1.93878300
O	-1.34720500	-2.98219200	-1.58275100

12	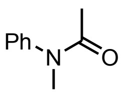	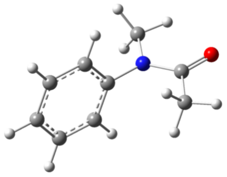

Cartesian Coordinates

C	2.04294300	-0.45946900	0.04995700
O	3.23789700	-0.19268600	0.01794200
N	1.07147700	0.52222500	-0.04962700
C	1.54837400	-1.89068100	0.20250900
C	1.47665800	1.91964600	-0.19267200
H	1.07872200	2.52367800	0.63139900
H	2.56603200	1.95065100	-0.17922800
H	1.10650800	2.33584700	-1.13742400
H	0.97372000	-2.21122400	-0.67292500
H	2.42335100	-2.53205200	0.31413500
H	0.89716500	-2.00369000	1.07530100
C	-0.33586500	0.25055100	-0.02549100
C	-1.04382500	0.31796000	1.18029800
C	-1.01852900	-0.04060100	-1.21220600
C	-2.41915300	0.08325800	1.19975800
H	-0.50761700	0.55039800	2.09638200
C	-2.39375700	-0.27698700	-1.19006800
H	-0.46326600	-0.08130500	-2.14534600
C	-3.09580200	-0.21578400	0.01533100
H	-2.96151500	0.13272900	2.14004600
H	-2.91629000	-0.50721800	-2.11456200
H	-4.16658700	-0.39924800	0.03158700

9 NMR Spectra

S9 ^{1}H NMR (400 MHz, $CDCl_3$)

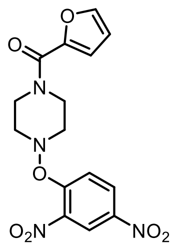

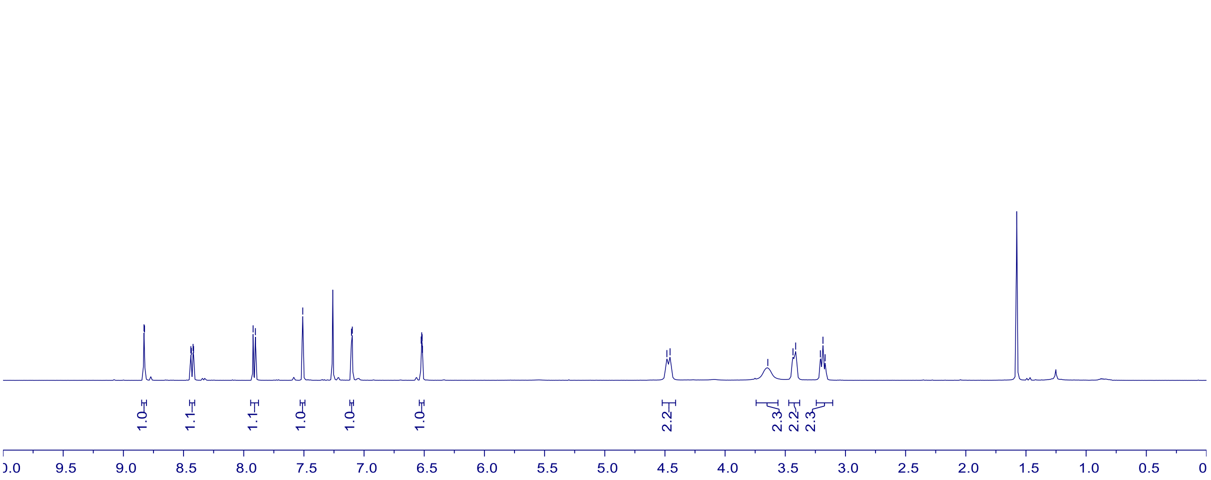

S9 ^{13}C NMR (101 MHz, $CDCl_3$)

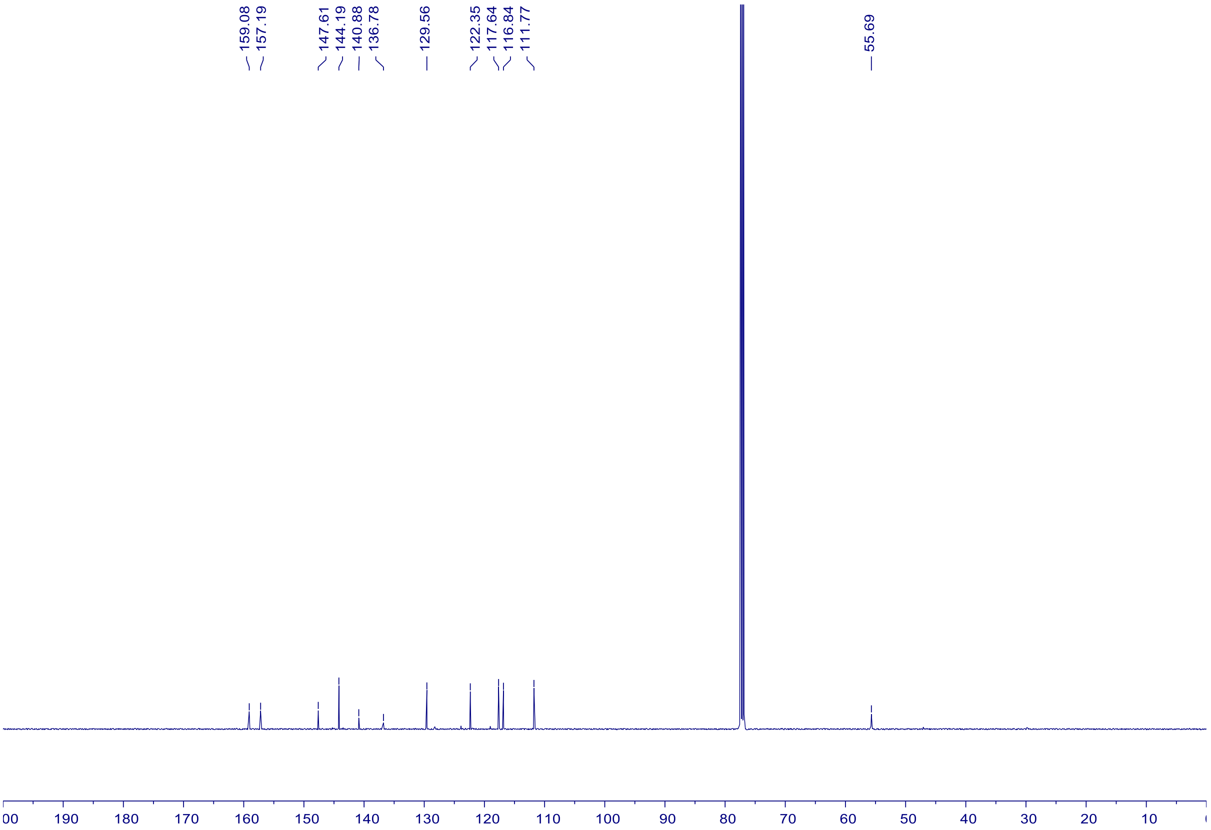

4 ^{1}H NMR (400 MHz, $CDCl_3$)

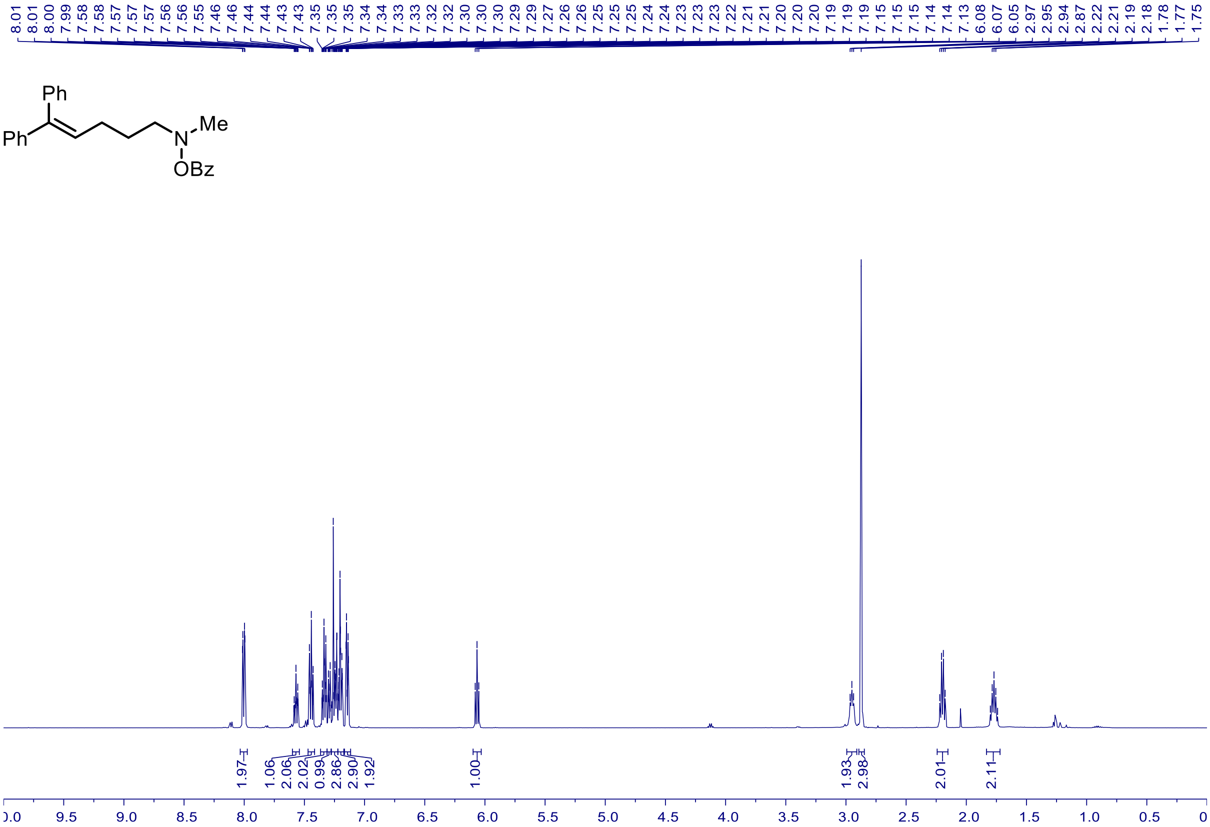

4 ^{13}C NMR (101 MHz, $CDCl_3$)

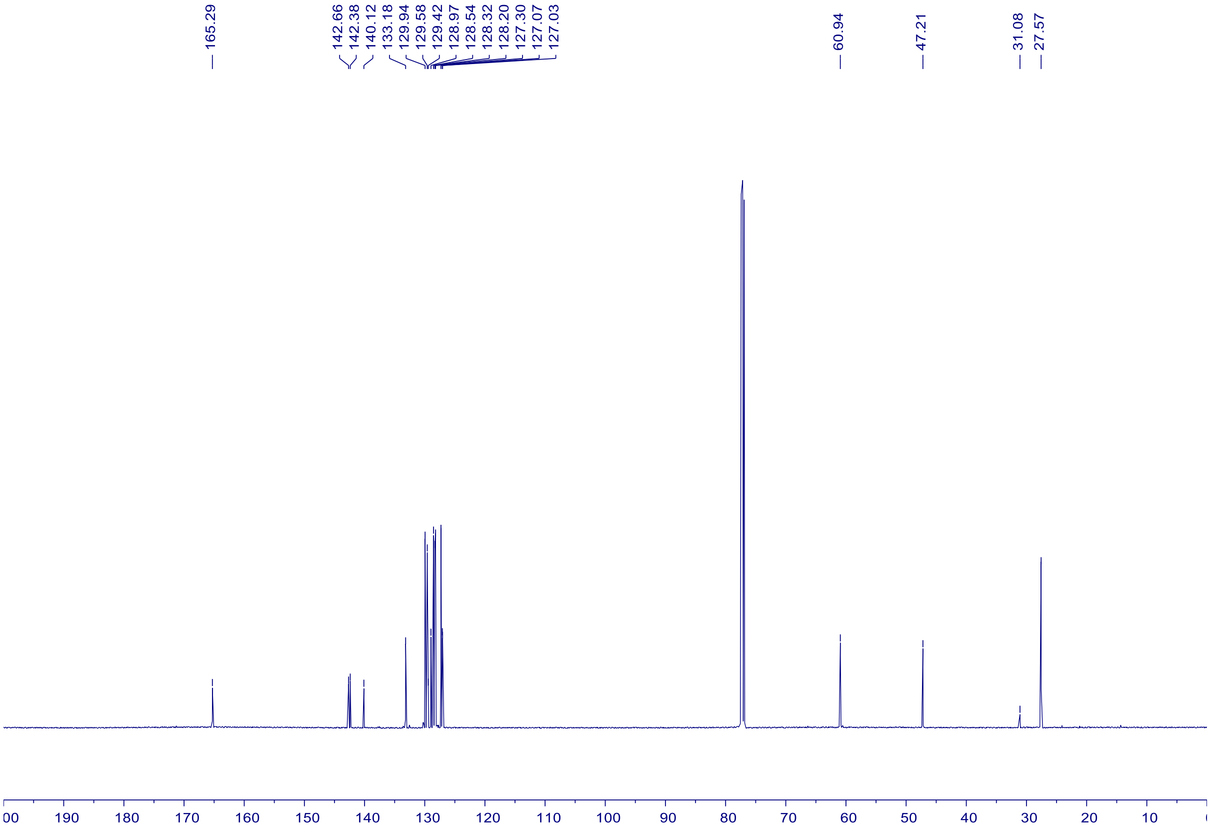

S12 ^{1}H NMR (400 MHz, $CDCl_3$)

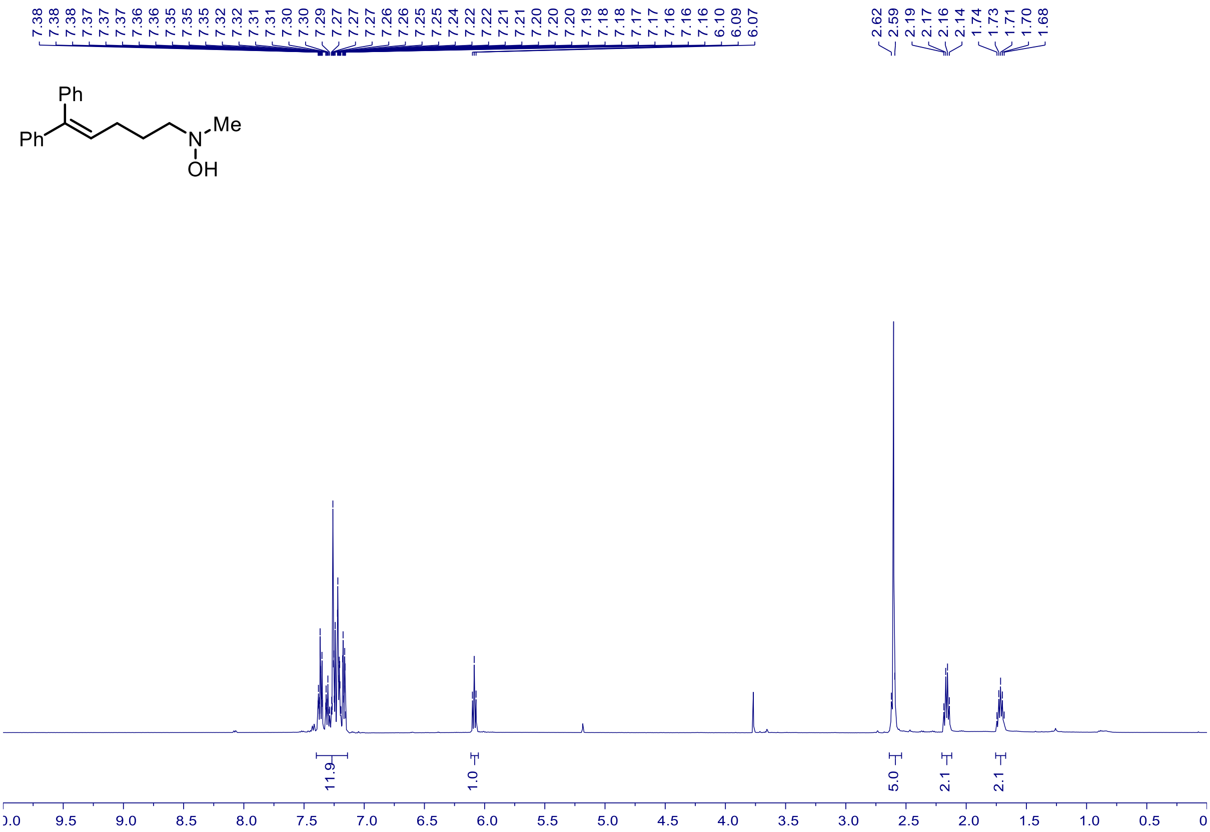

S12 ^{13}C NMR (101 MHz, $CDCl_3$)

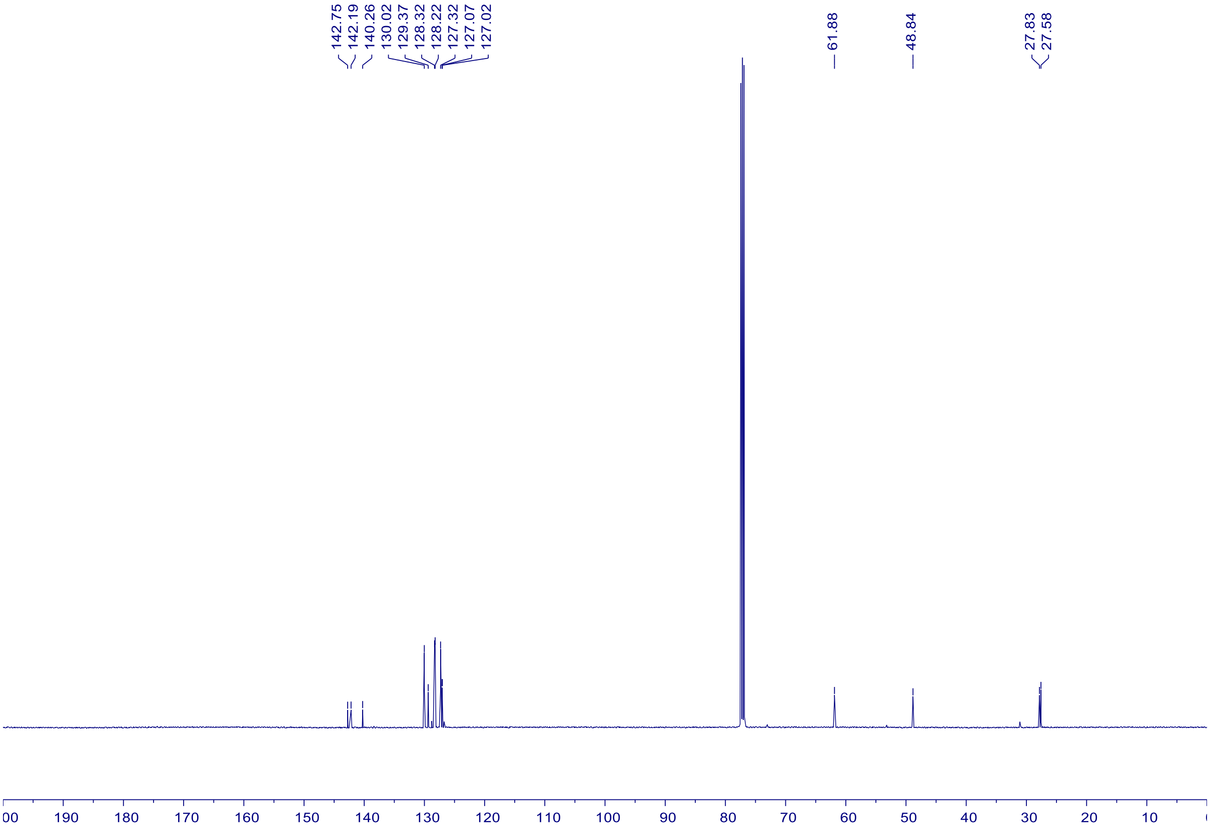

3 ^{1}H NMR (400 MHz, $CDCl_3$)

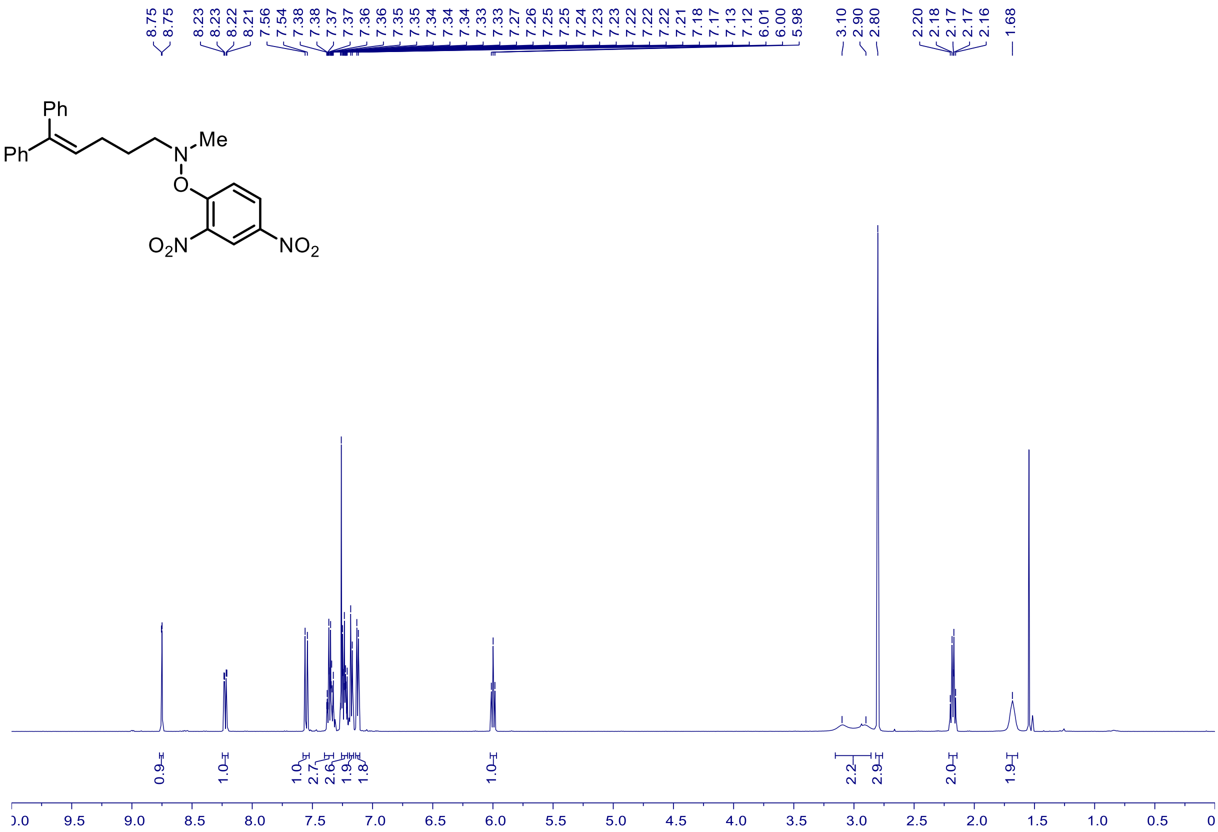

3 ^{13}C NMR (101 MHz, $CDCl_3$)

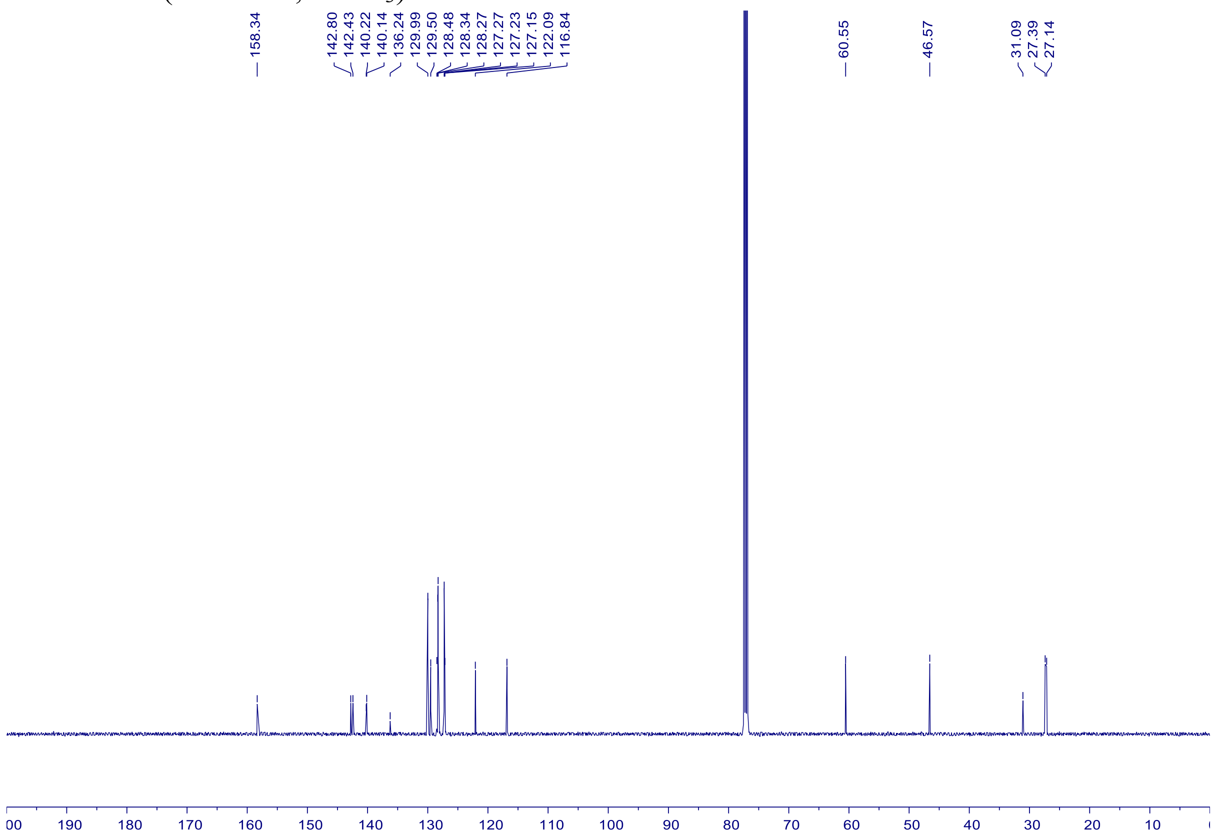

60 ^{1}H NMR (400 MHz, $CDCl_3$)

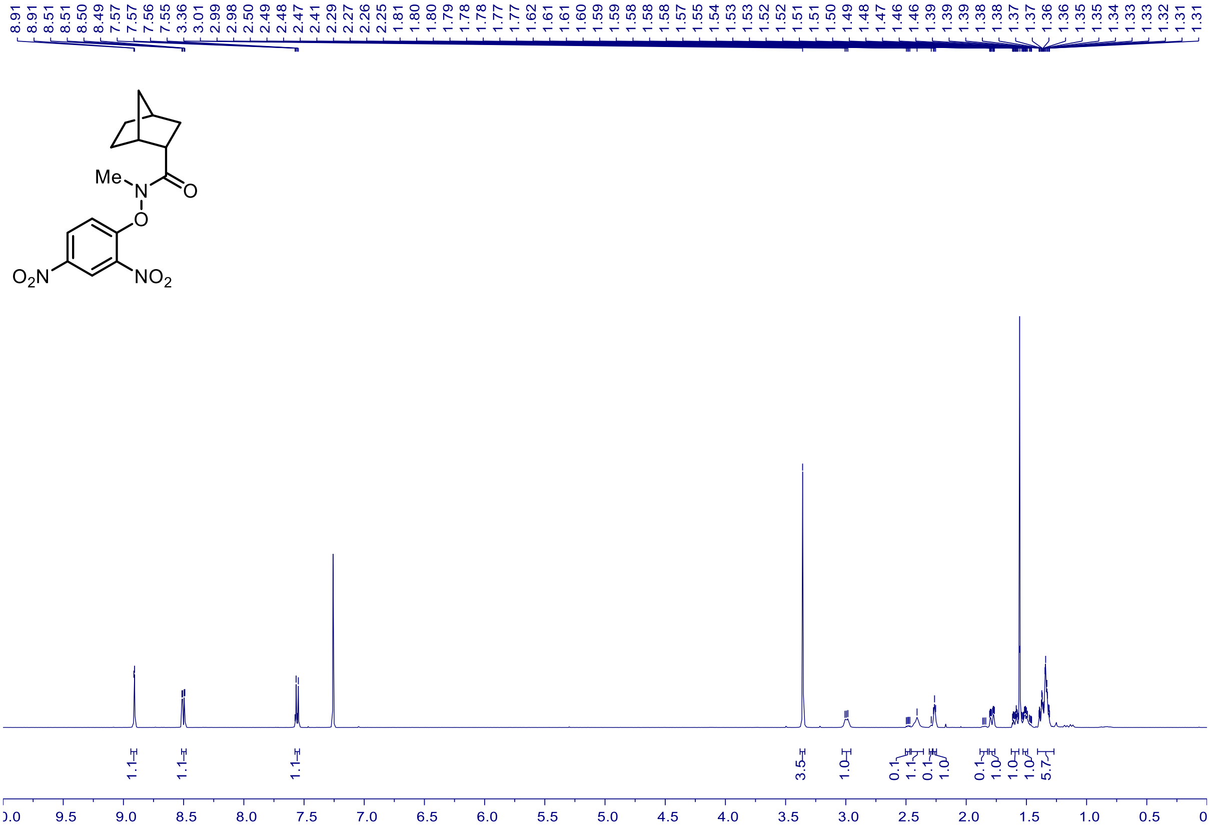

60 ^{13}C NMR (101 MHz, $CDCl_3$)

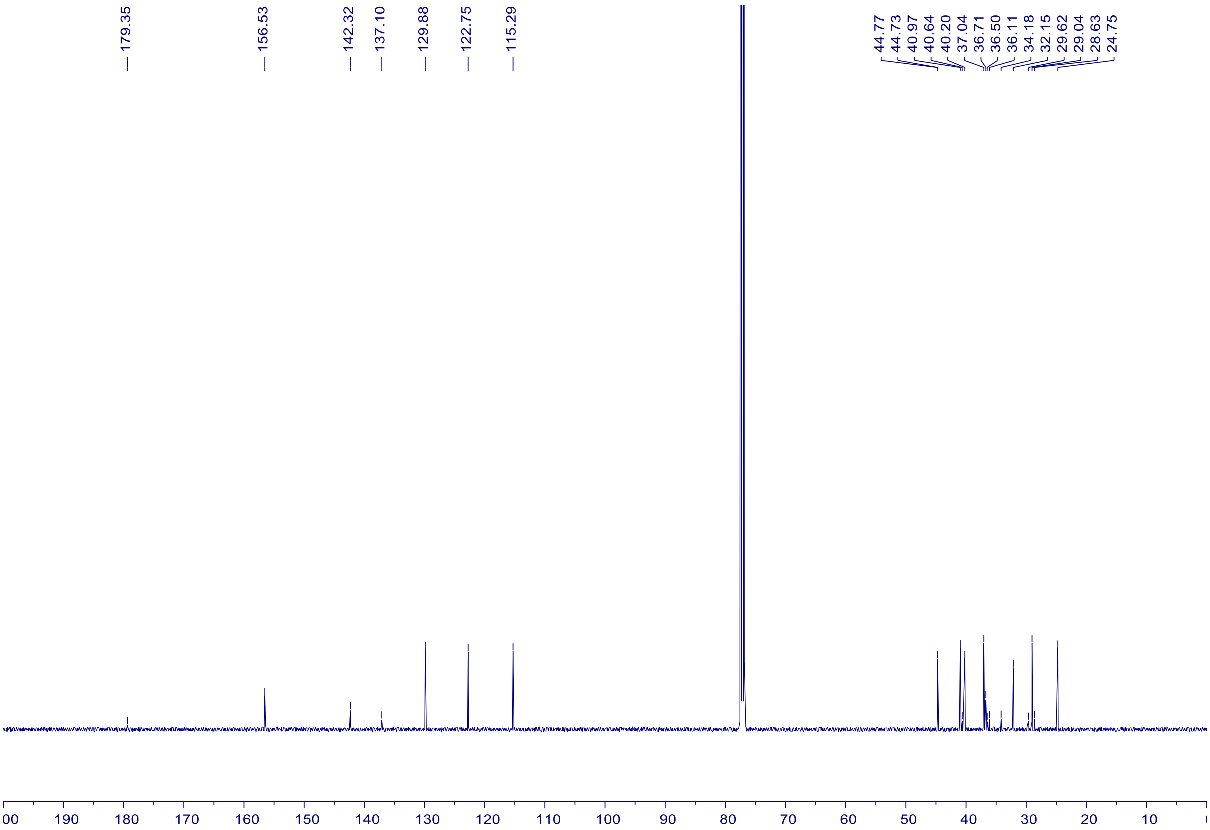

52 ^{1}H NMR (400 MHz, $CDCl_3$)

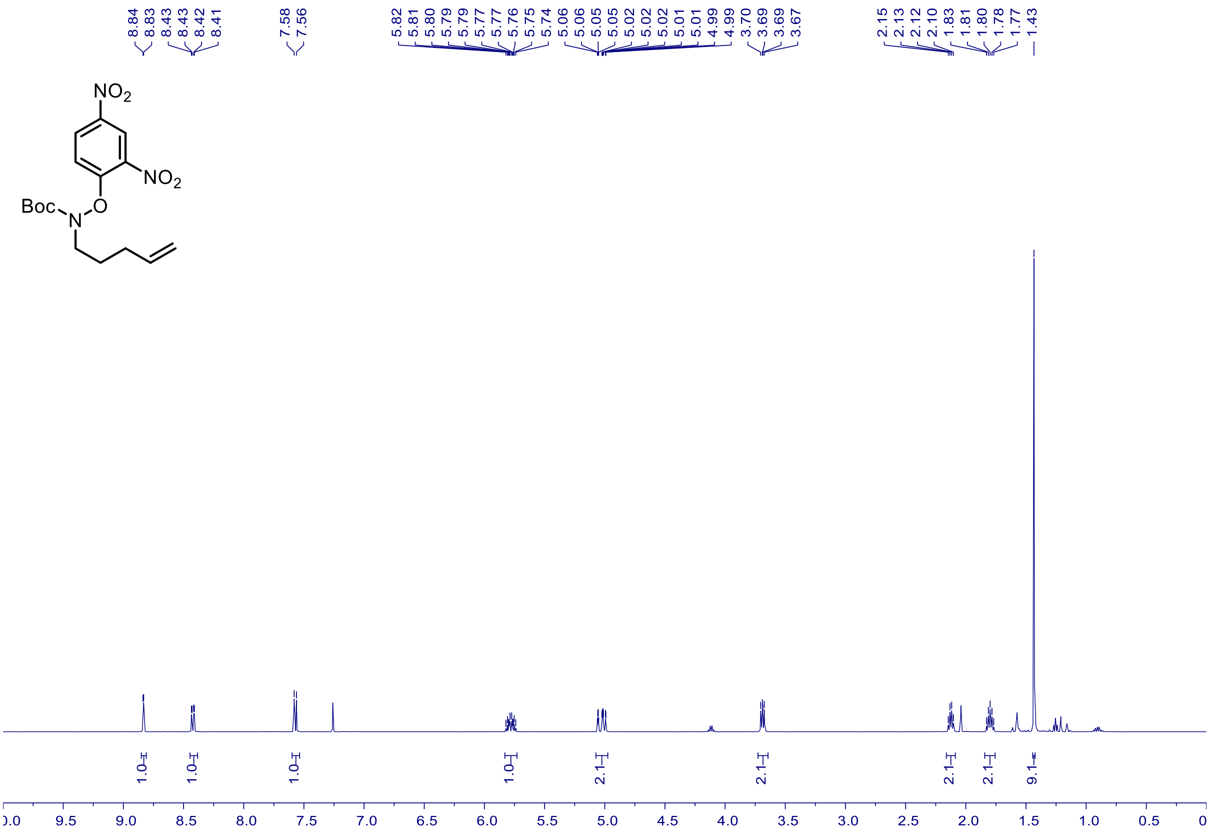

52 ^{13}C NMR (101 MHz, CDCl$_3$)

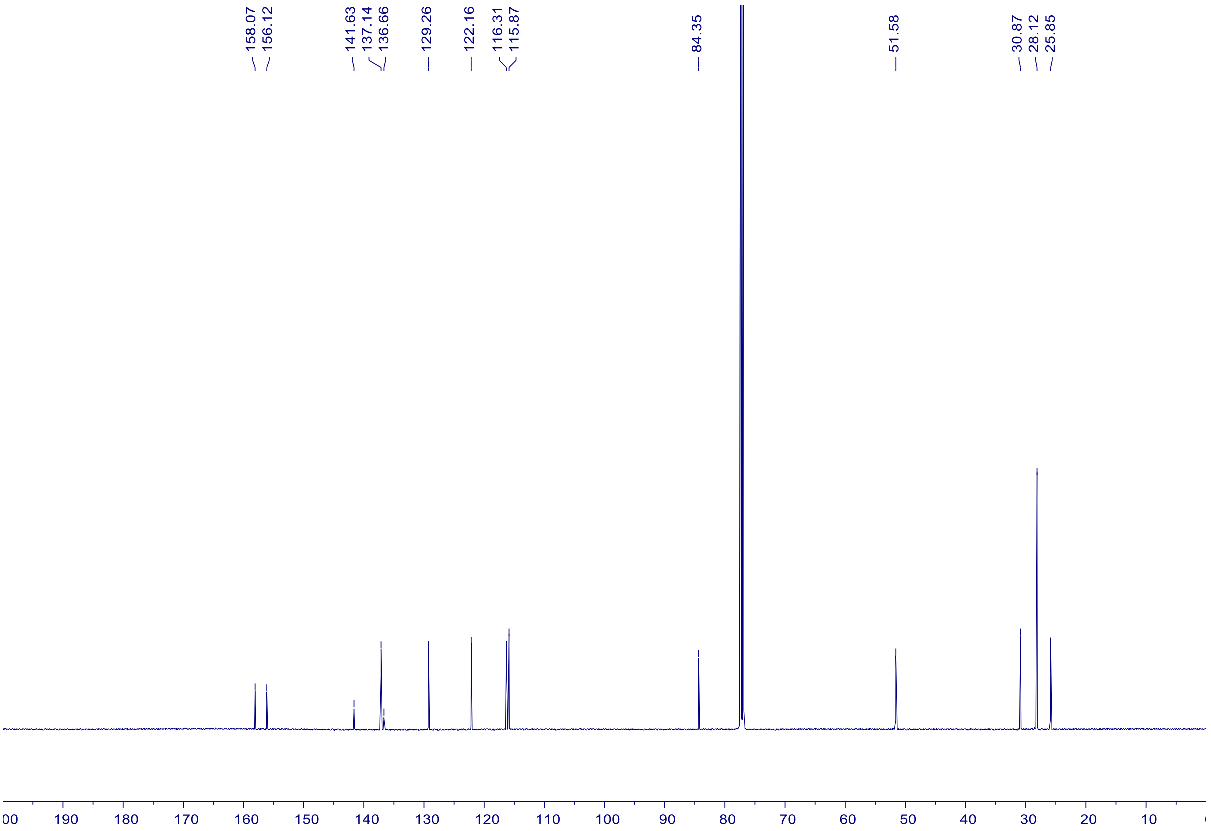

78 ^{1}H NMR (500 MHz, CDCl$_3$)

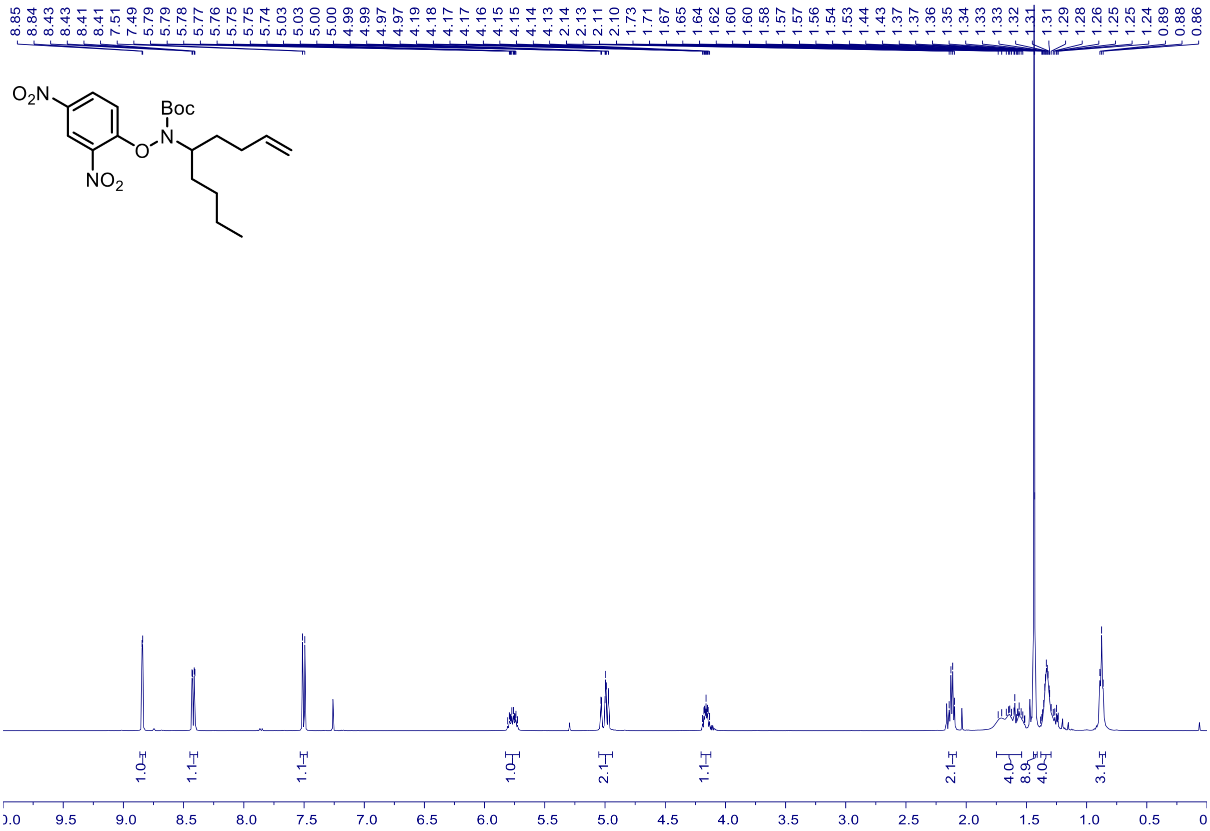

78 ^{13}C NMR (126 MHz, $CDCl_3$)

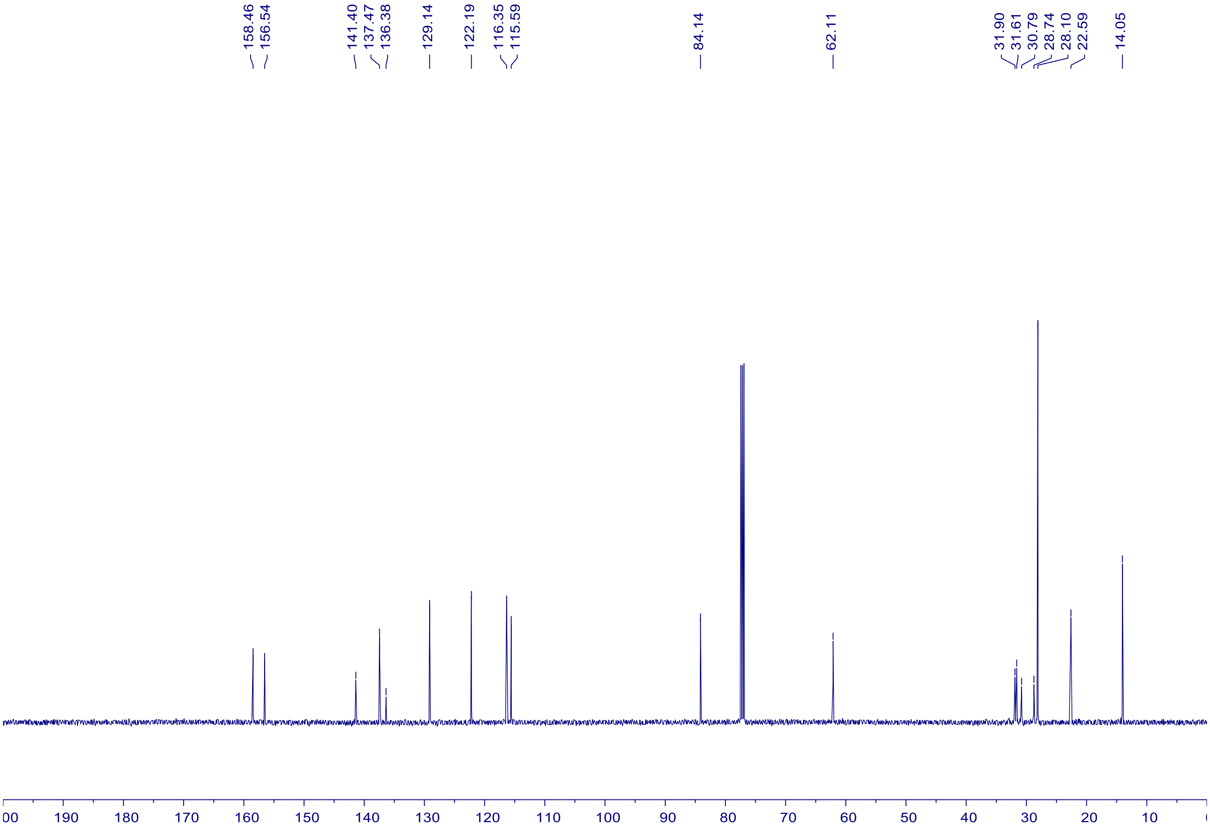

6 ^{1}H NMR (400 MHz, $CDCl_3$)

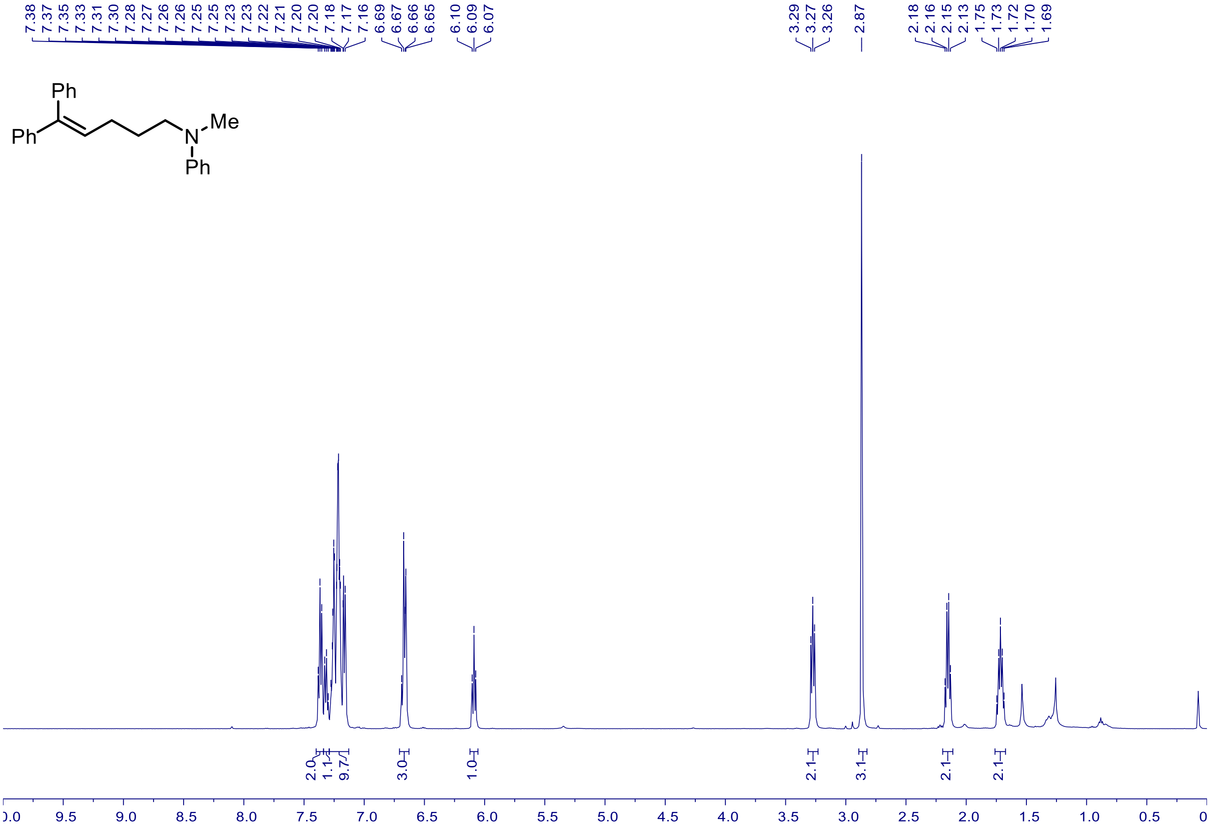

6 ^{13}C NMR (101 MHz, $CDCl_3$)

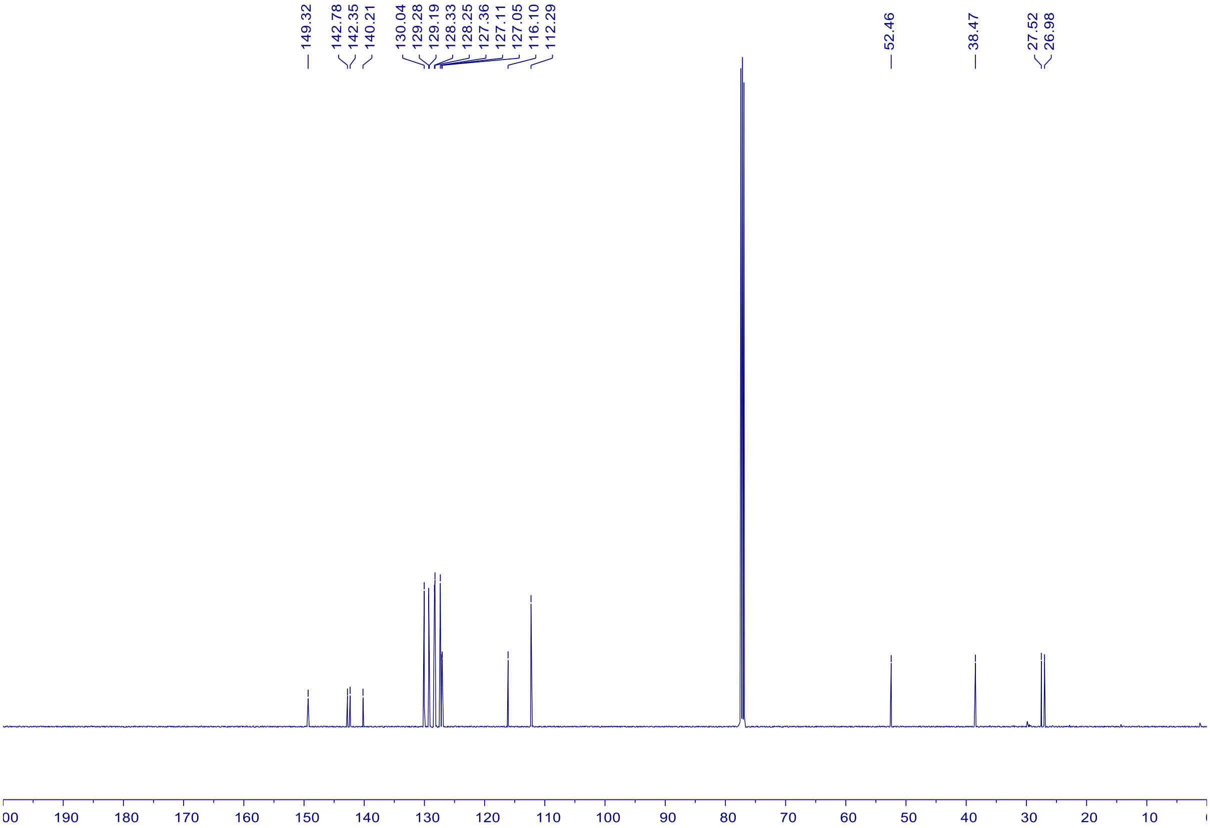

7 ^{1}H NMR (400 MHz, $CDCl_3$)

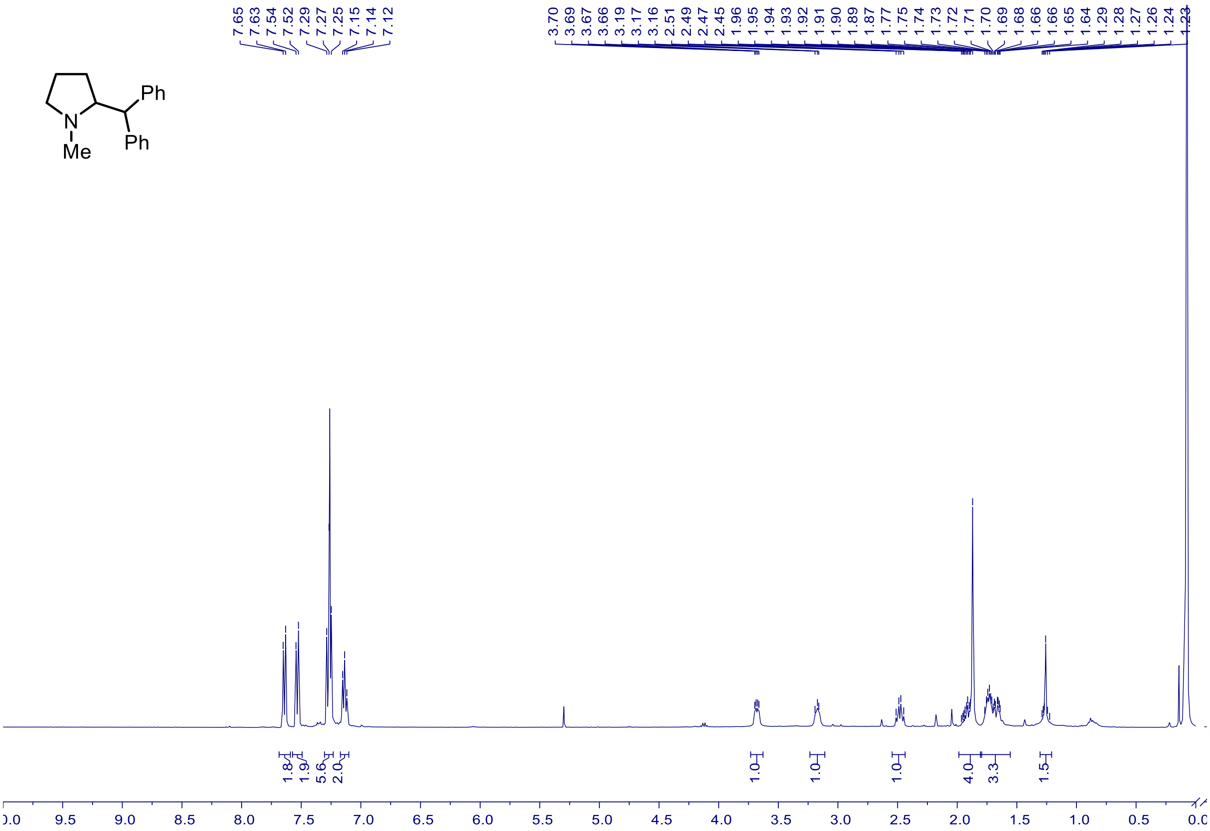

7 ^{13}C NMR (101 MHz, $CDCl_3$)

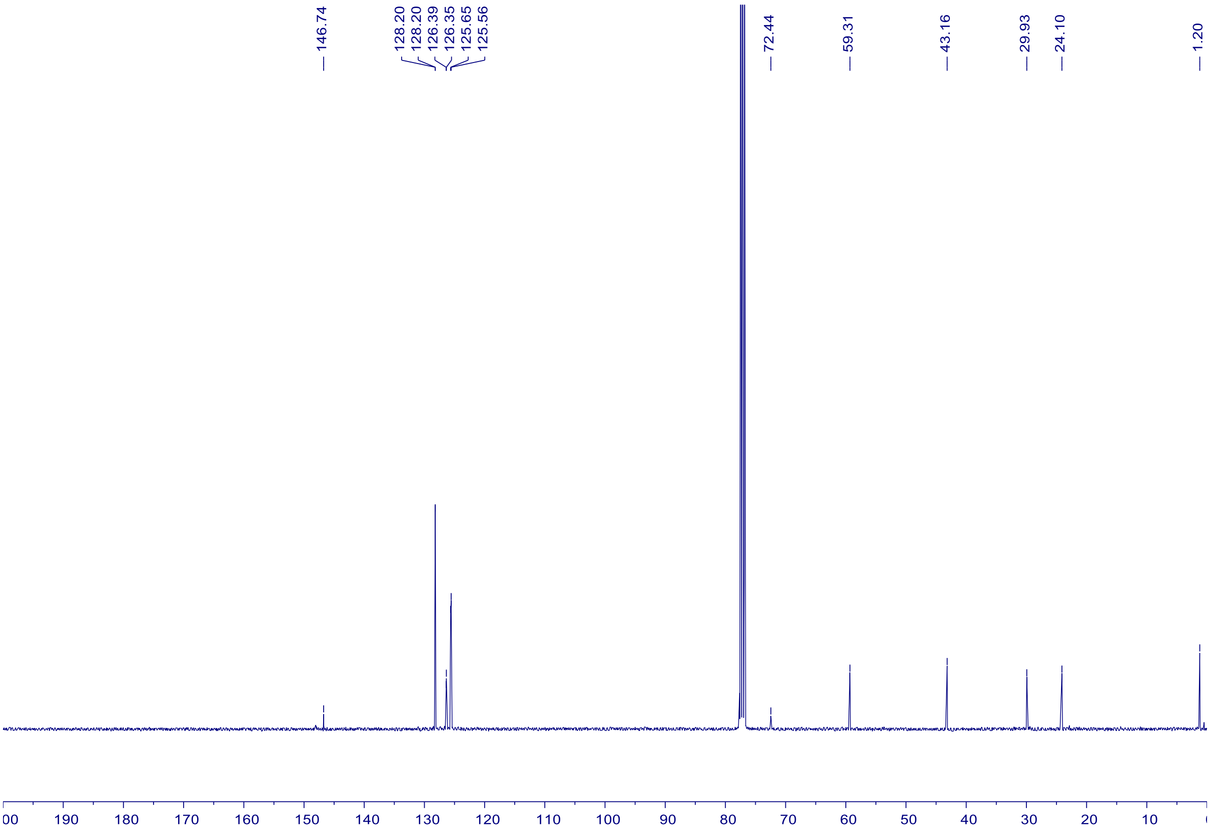

8 ^{1}H NMR (400 MHz, $CDCl_3$)

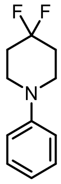

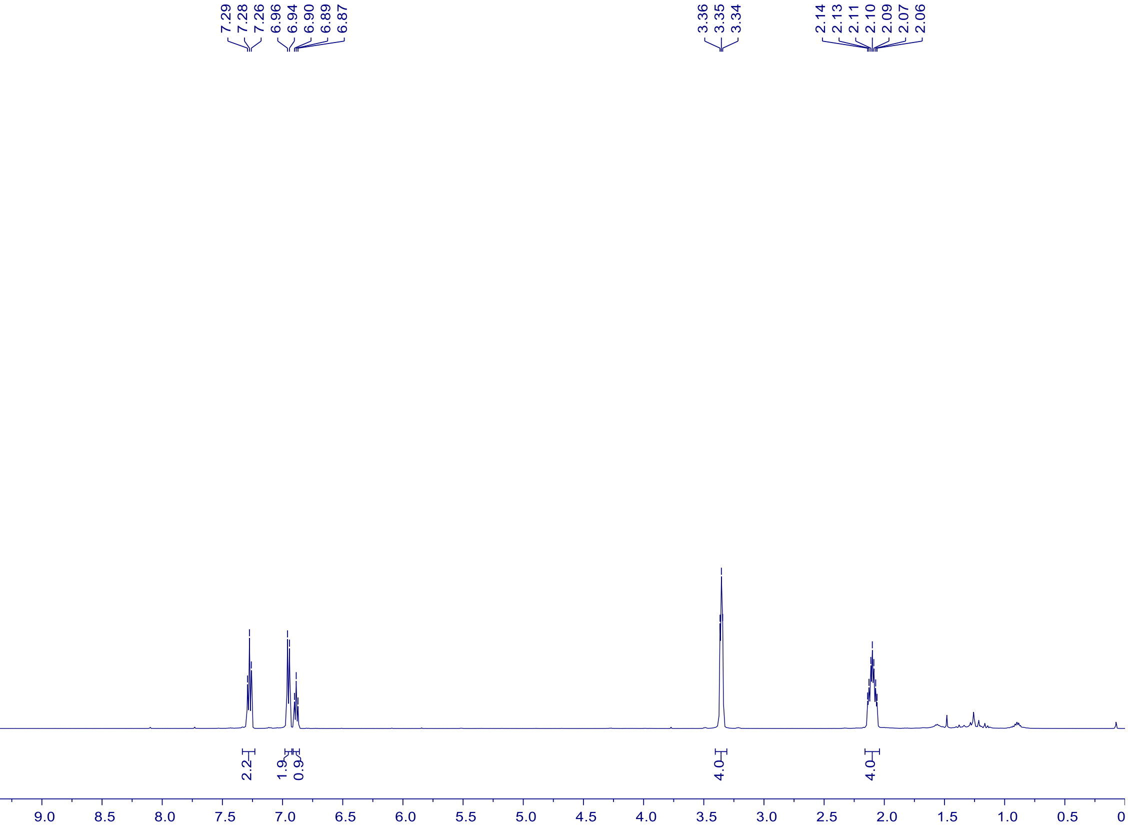

8 ^{13}C NMR (101 MHz, $CDCl_3$)

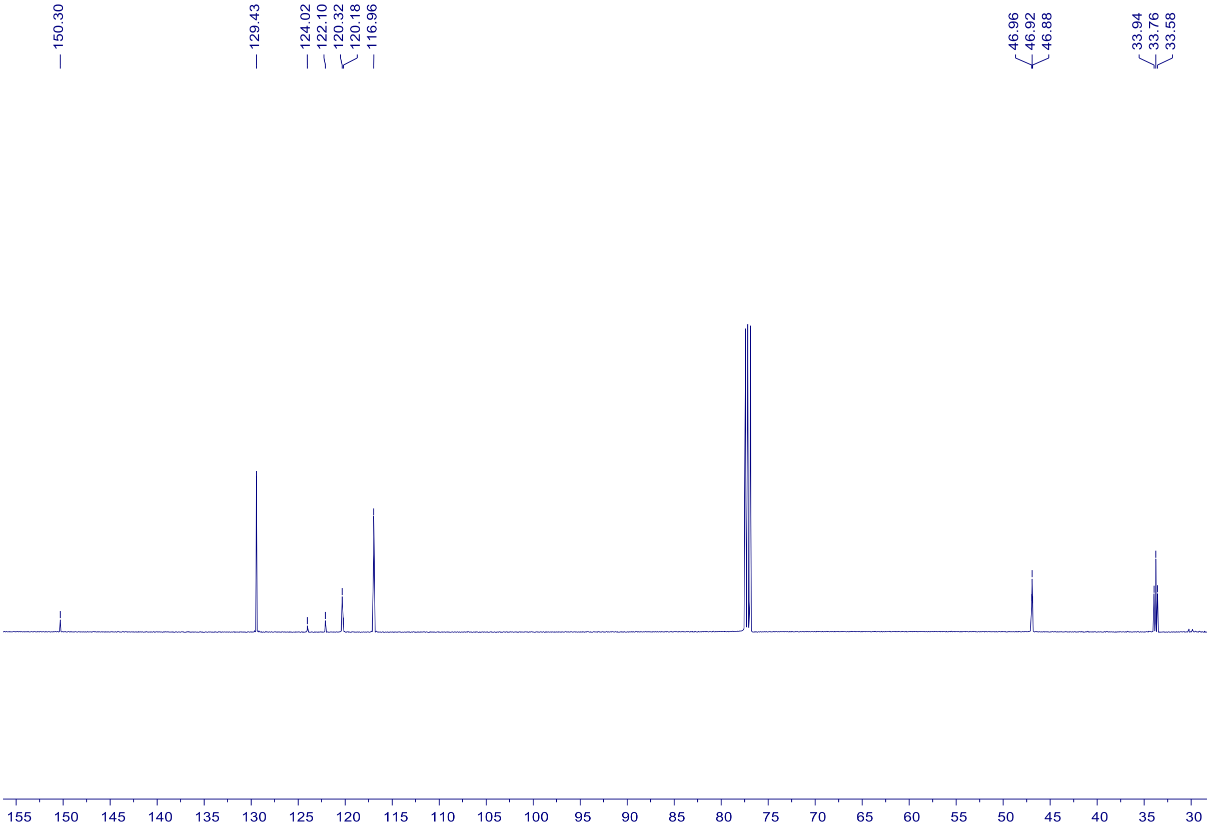

8 ^{19}F NMR (376 MHz, $CDCl_3$)

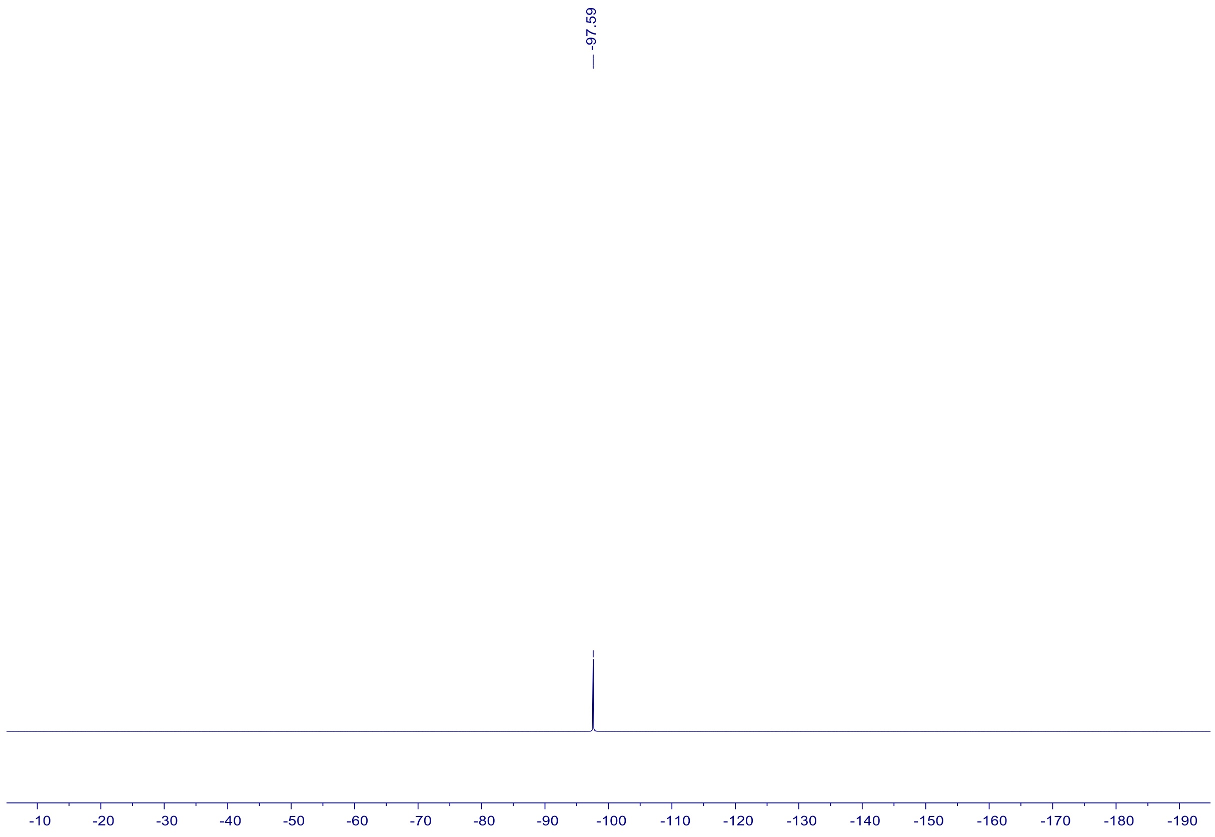
-97.59
-10
-20
-30
-40
-50
-60
-70
-80
-90
-100
-110
-120
-130
-140
-150
-160
-170
-180
-190

29 ^{1}H NMR (400 MHz, $CDCl_3$)

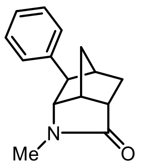

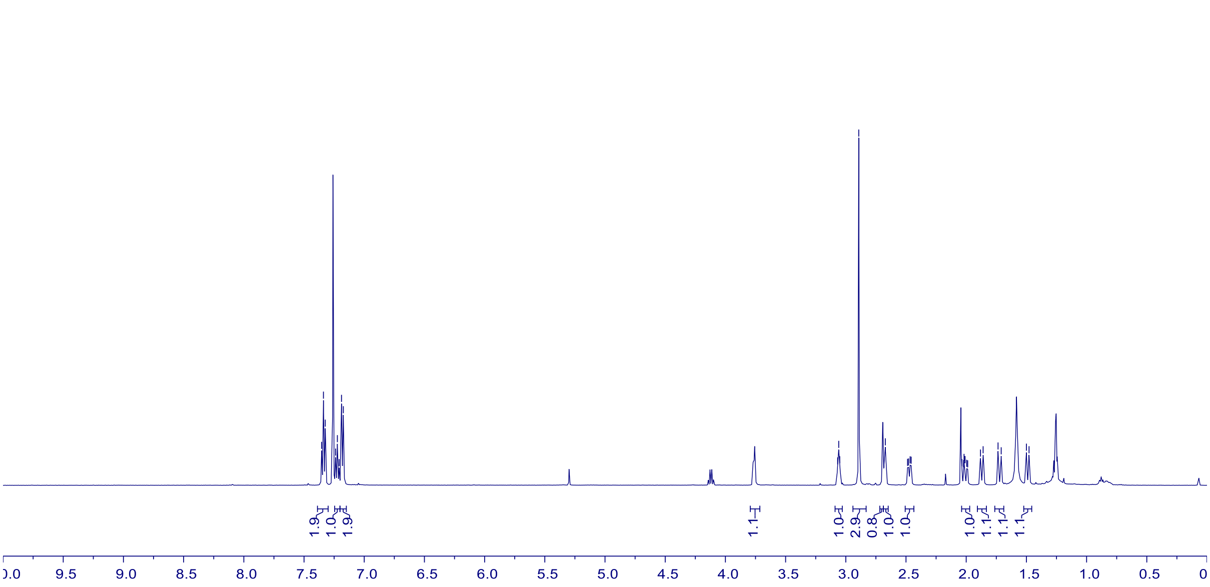

29 ^{13}C NMR (101 MHz, $CDCl_3$)

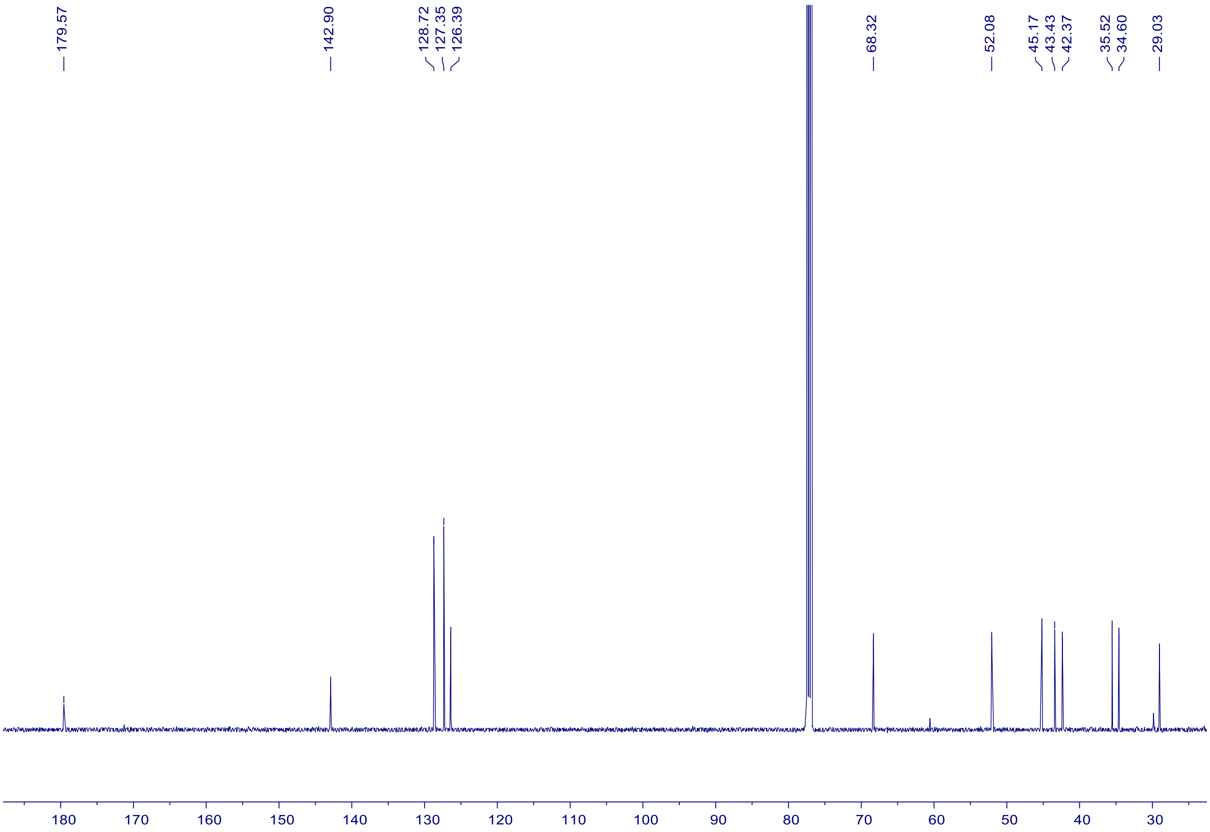

30 ^{1}H NMR (400 MHz, $CDCl_3$)

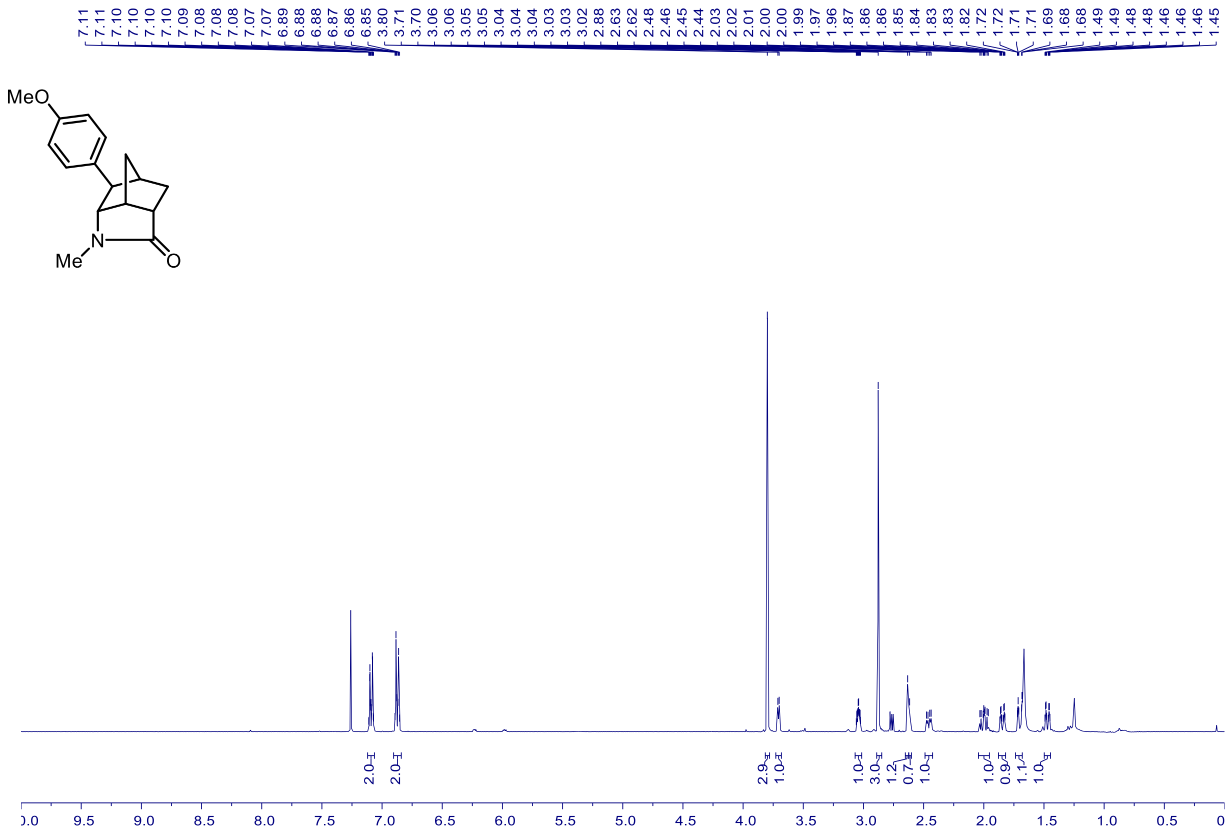

30 ^{13}C NMR (101 MHz, $CDCl_3$)

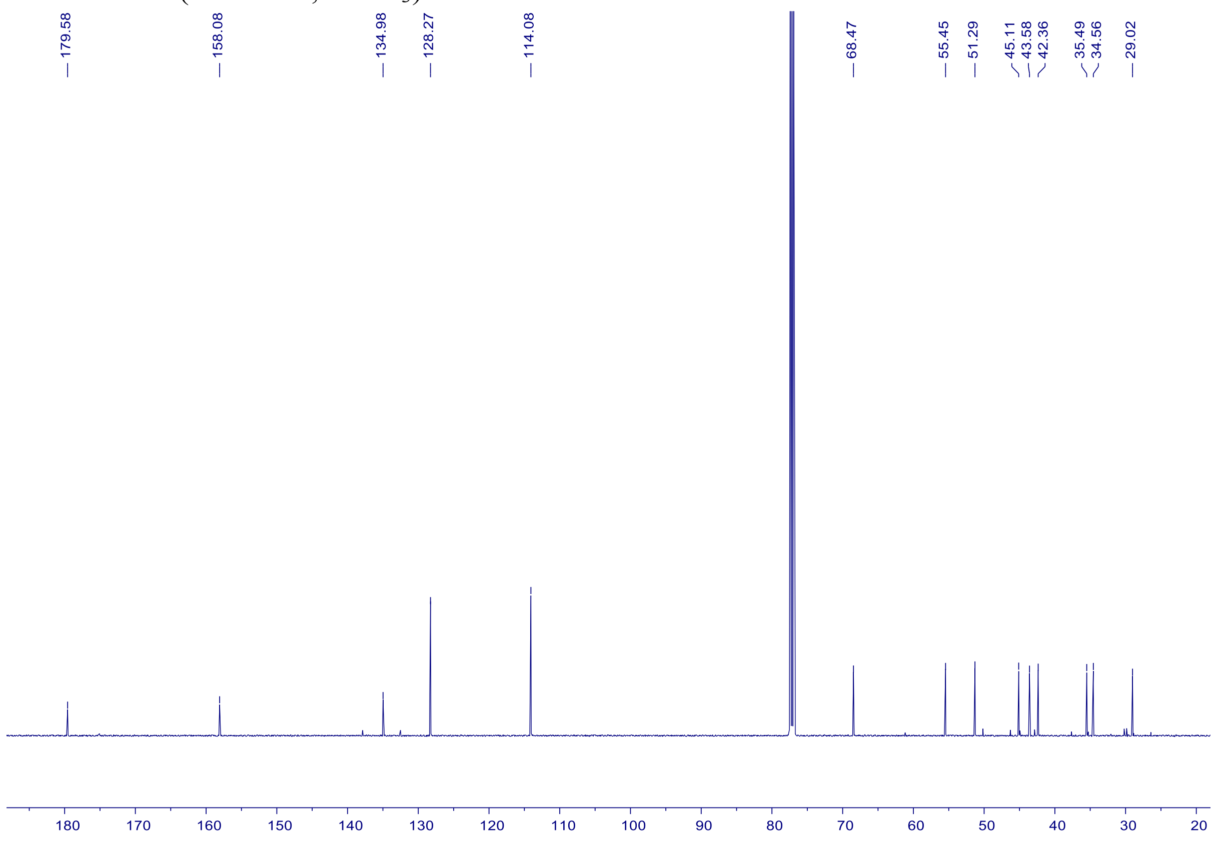

31 ^{1}H NMR (400 MHz, $CDCl_3$)

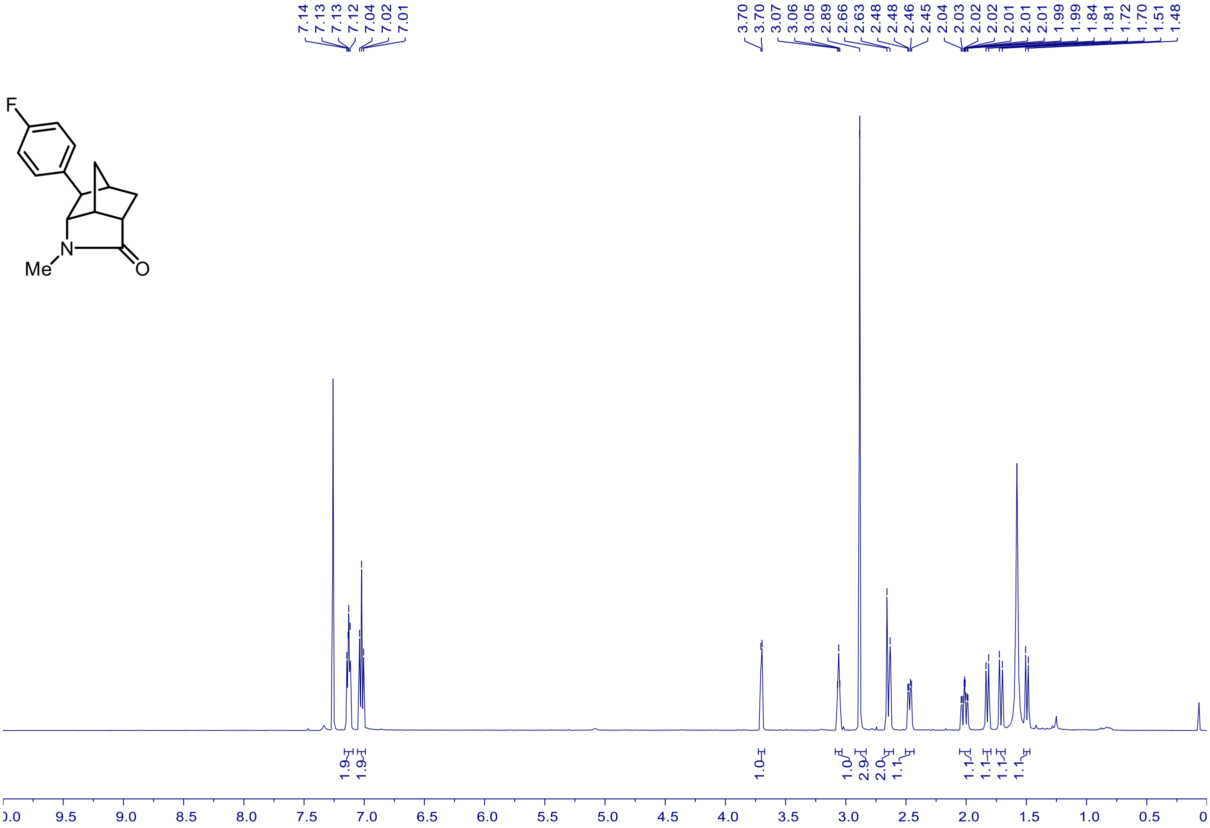

31 ^{13}C NMR (101 MHz, CDCl$_3$)

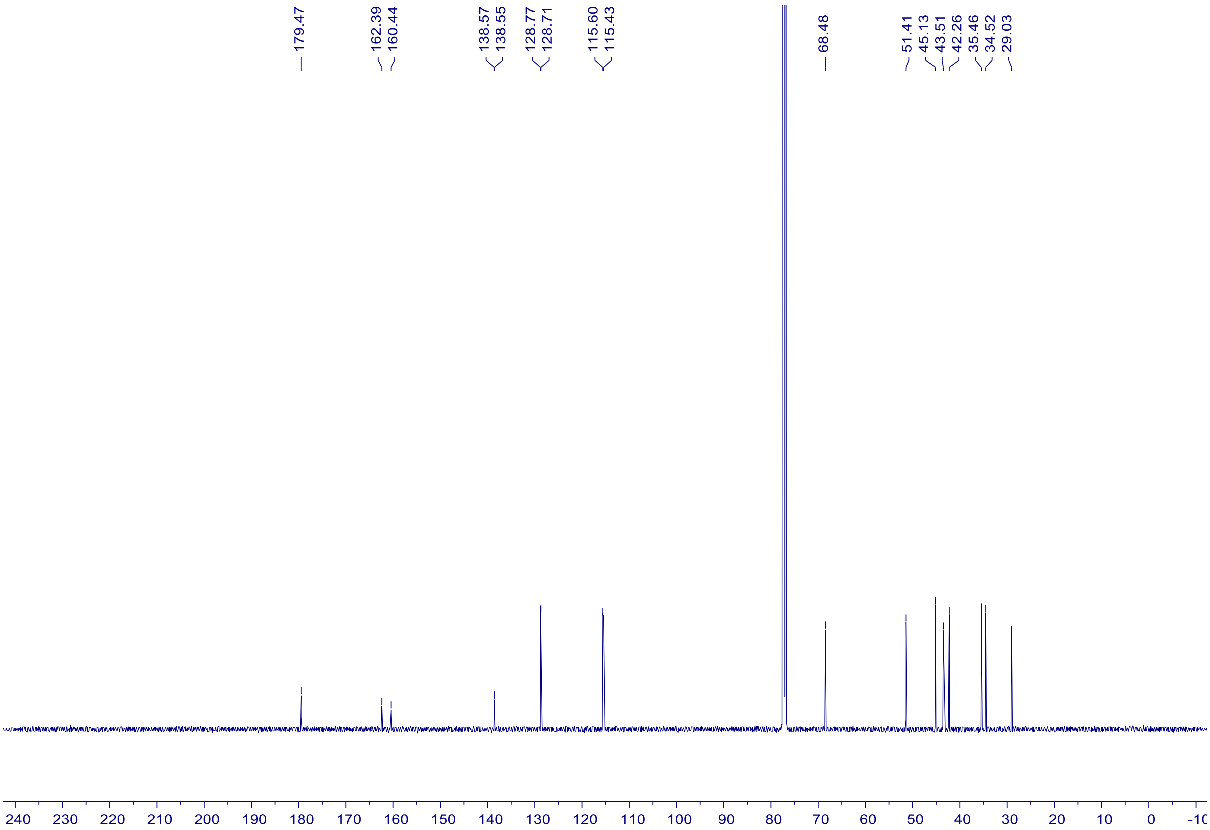

31 ^{19}F NMR (376 MHz, CDCl$_3$)

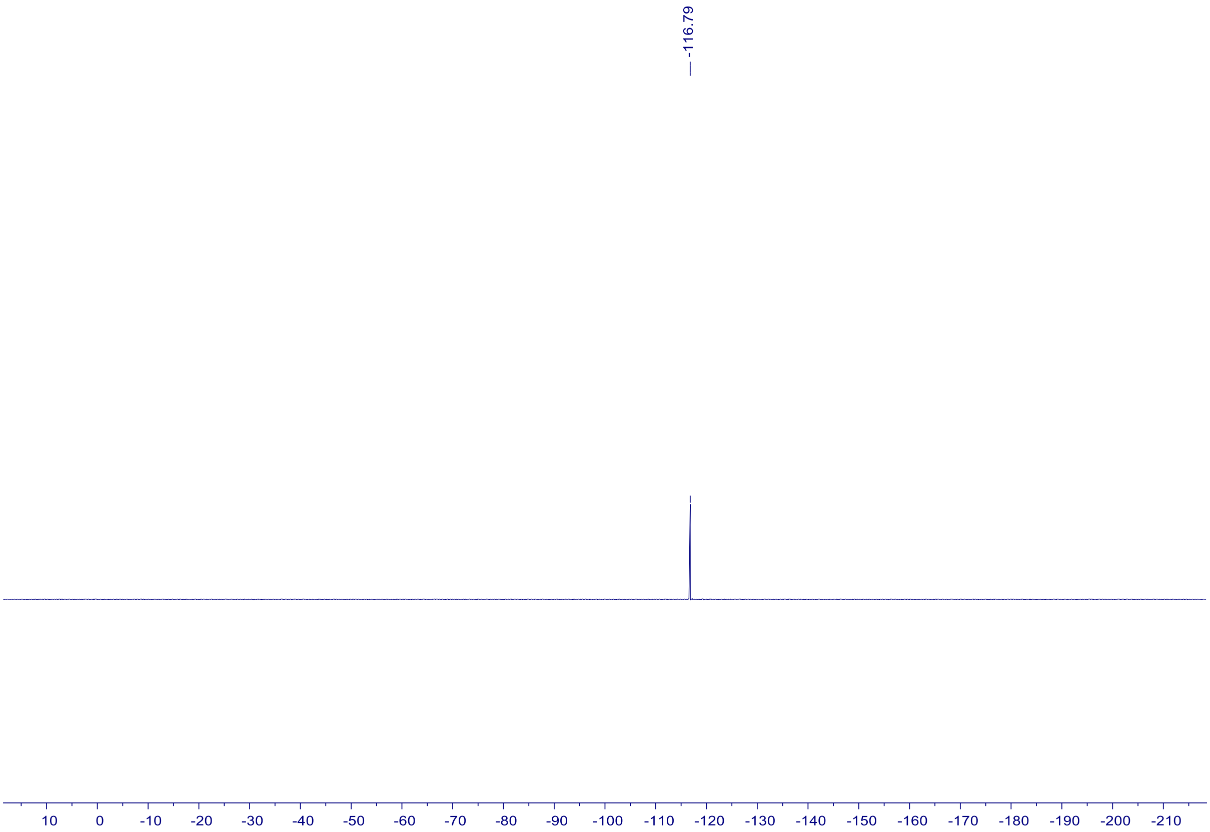
-116.79
10
0
-10
-20
-30
-40
-50
-60
-70
-80
-90
-100
-110
-120
-130
-140
-150
-160
-170
-180
-190
-200
-210

32 ^{1}H NMR (500 MHz, $CDCl_3$)

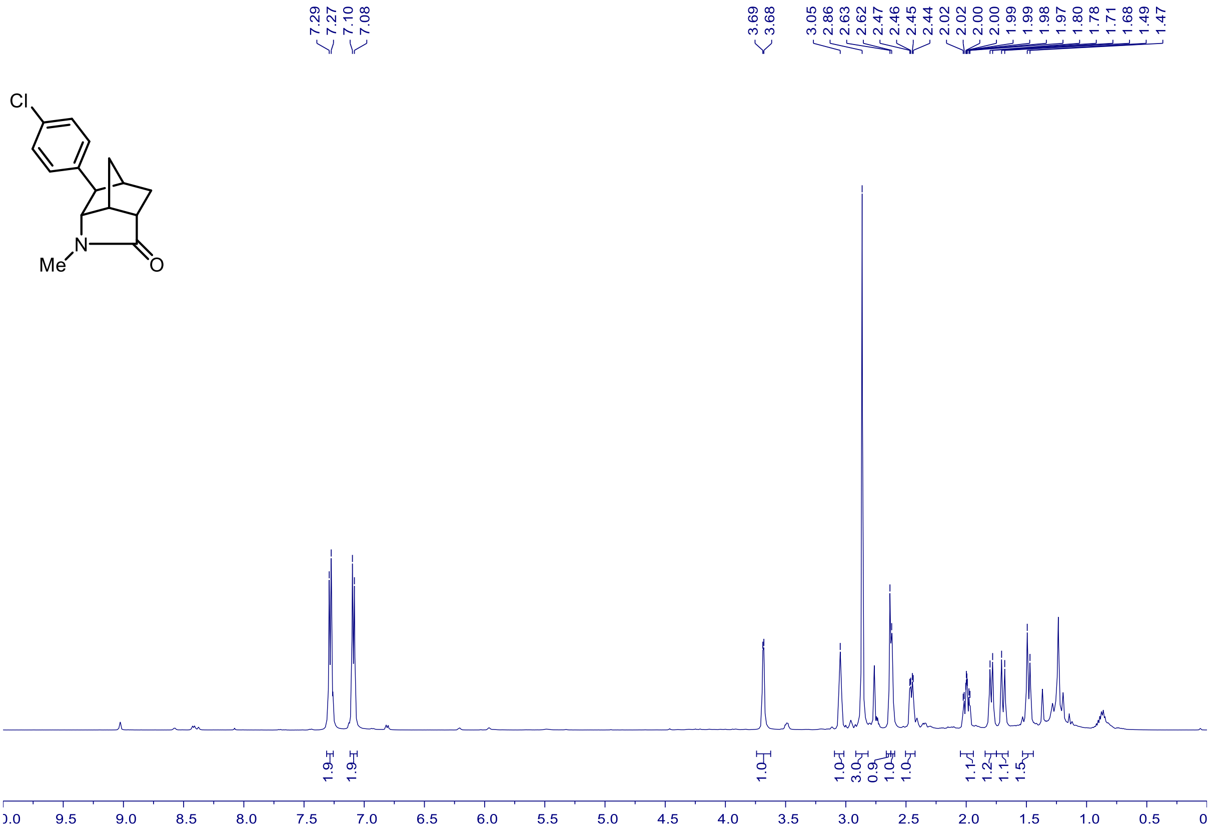

32 ^{13}C NMR (126 MHz, $CDCl_3$)

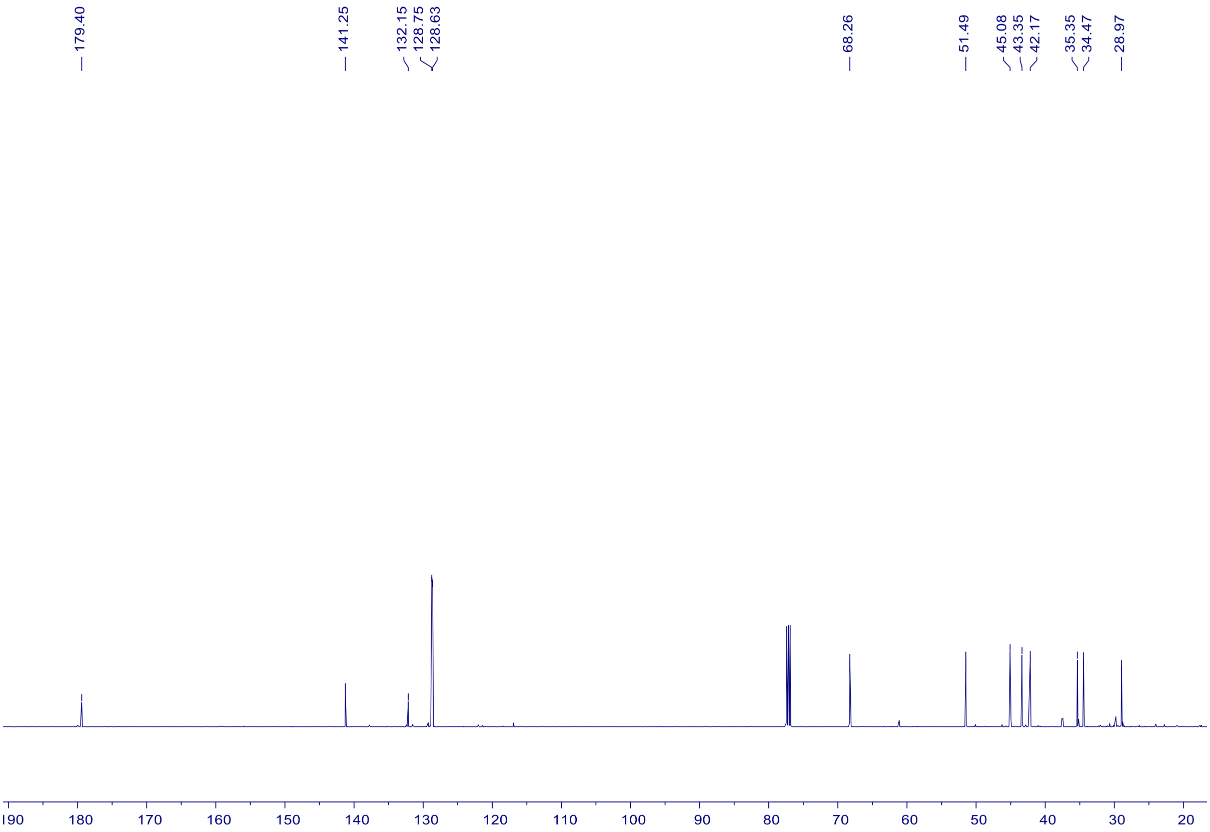

33 ^{1}H NMR (500 MHz, $CDCl_3$)

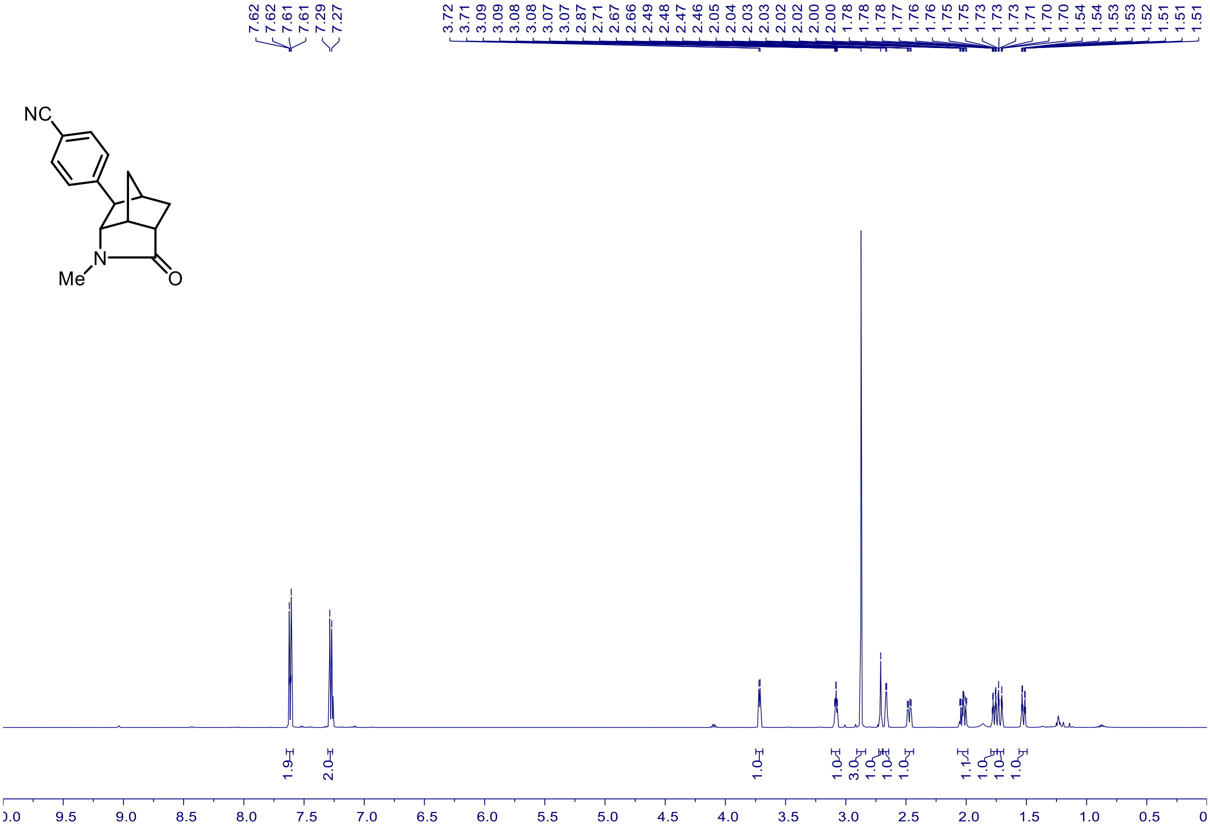

33 ^{13}C NMR (126 MHz, $CDCl_3$)

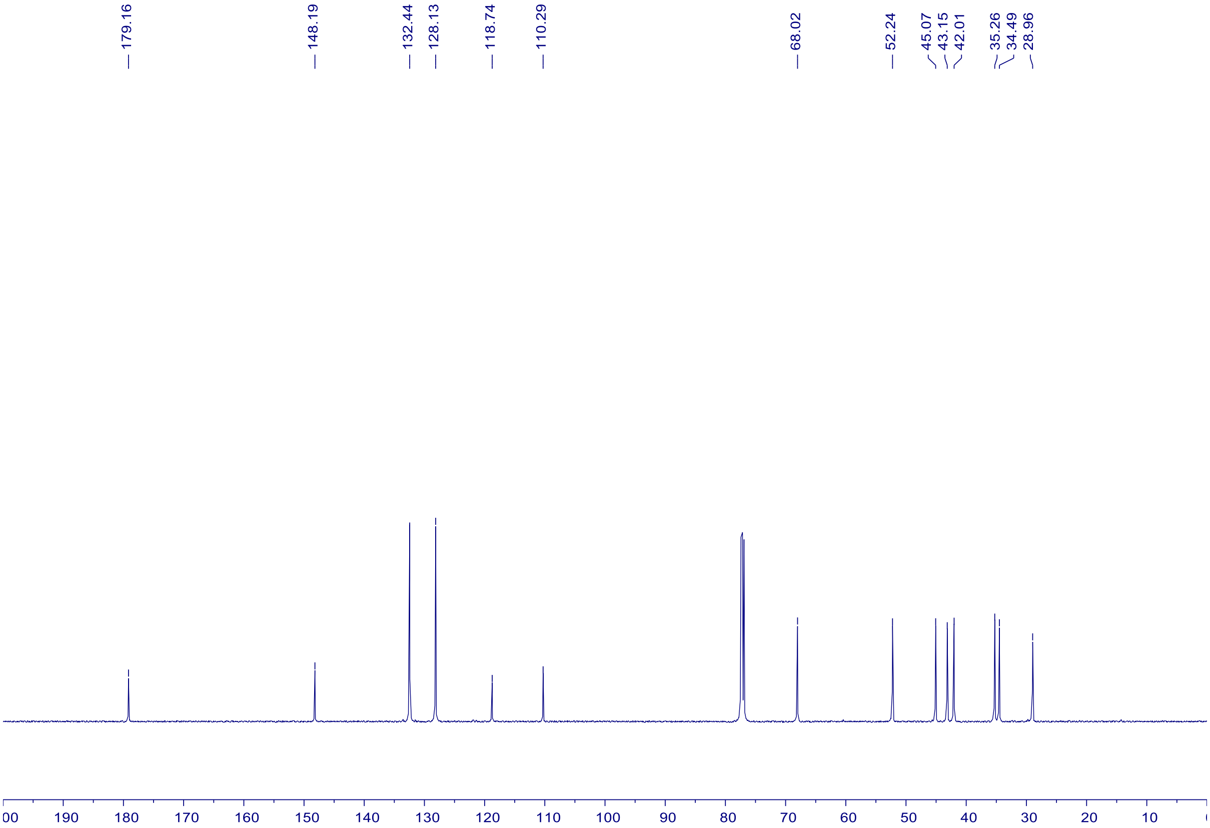

34 ^{1}H NMR (500 MHz, $CDCl_3$)

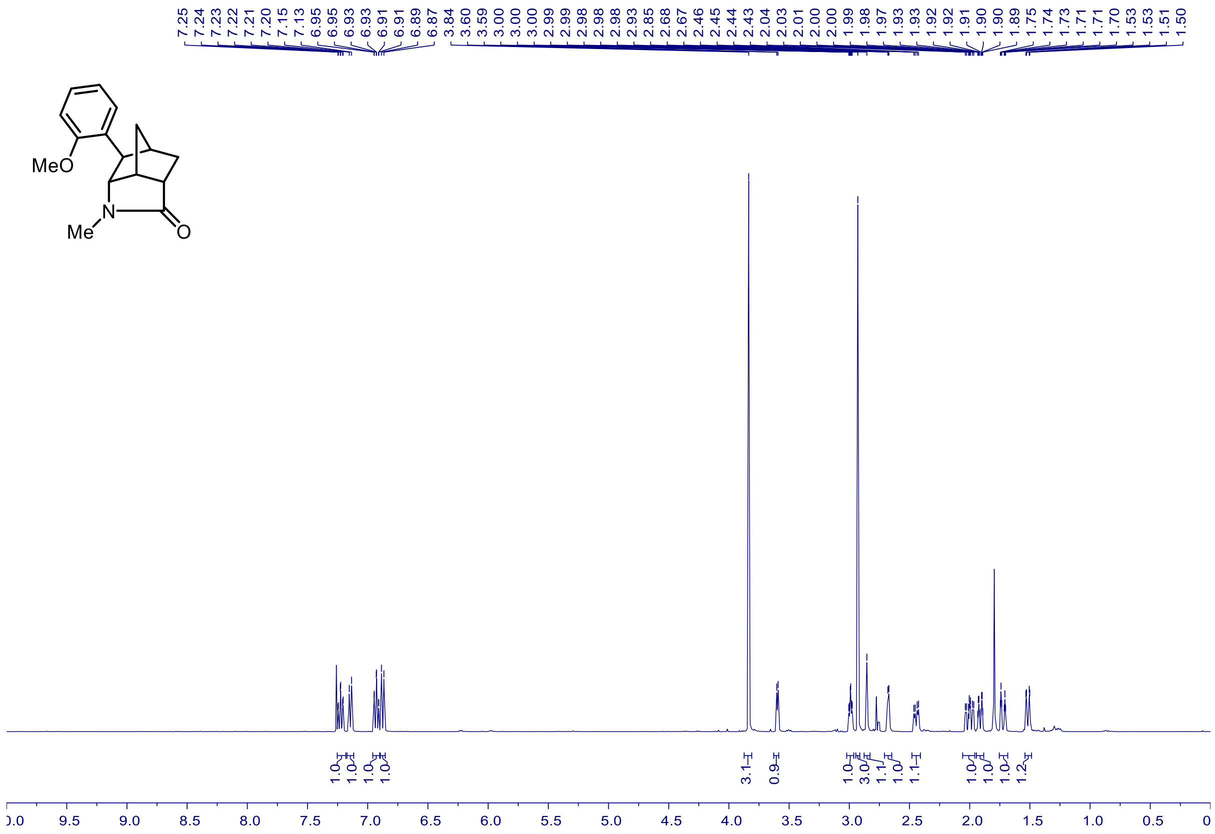

34 ^{13}C NMR (126 MHz, $CDCl_3$)

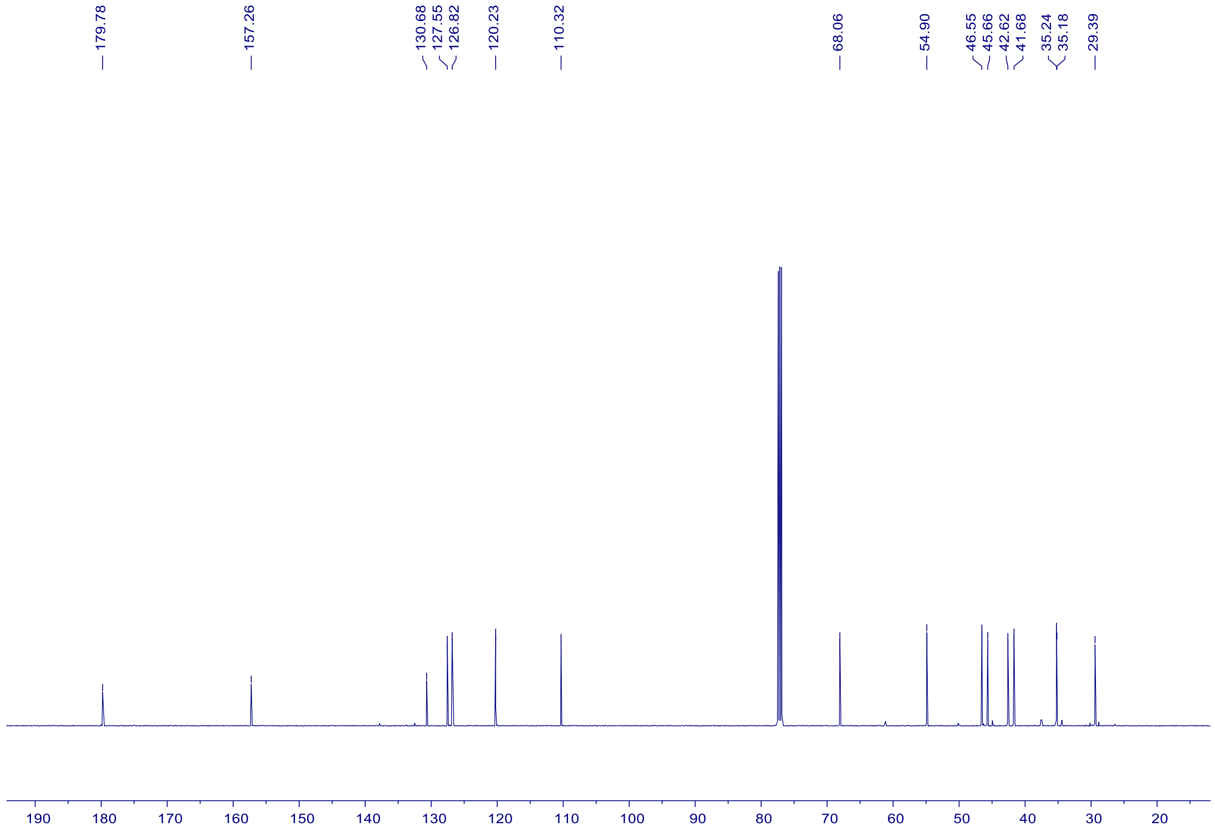

35 ^{1}H NMR (500 MHz, $CDCl_3$)

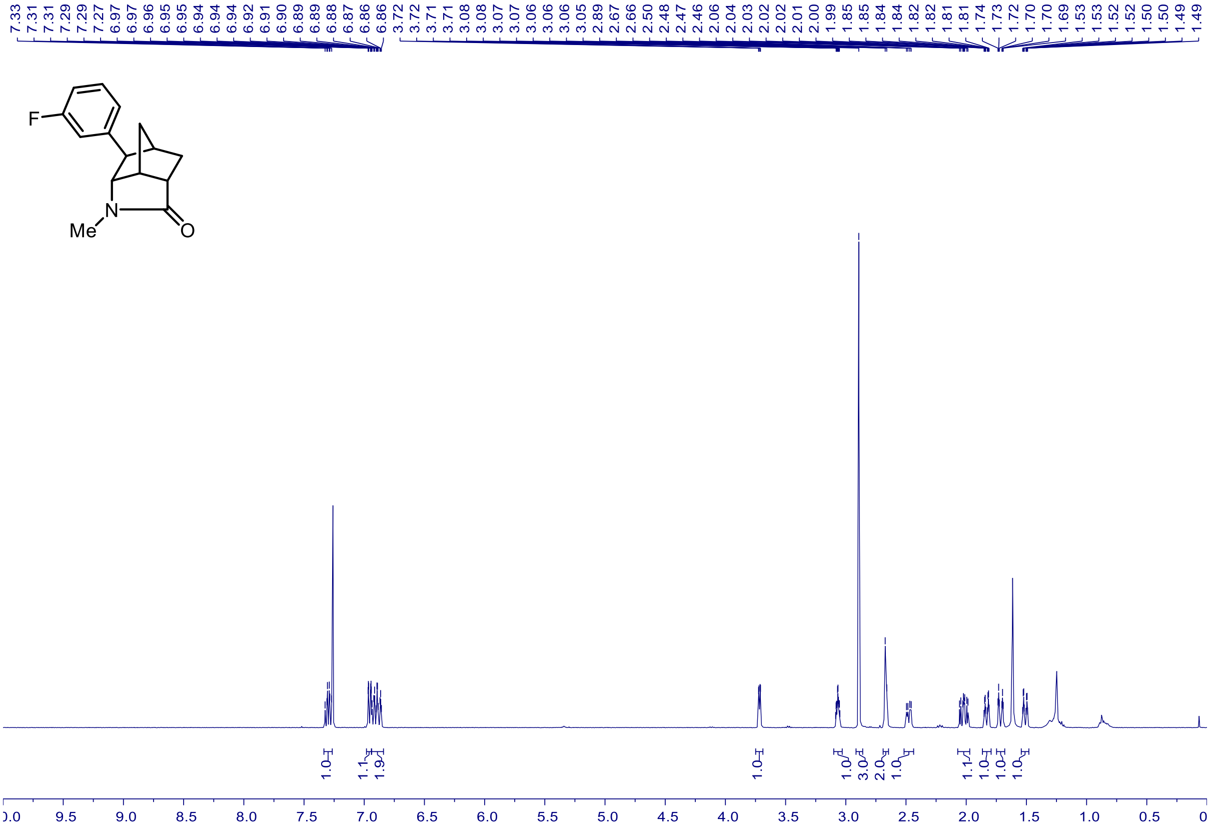

35 ^{13}C NMR (126 MHz, $CDCl_3$)

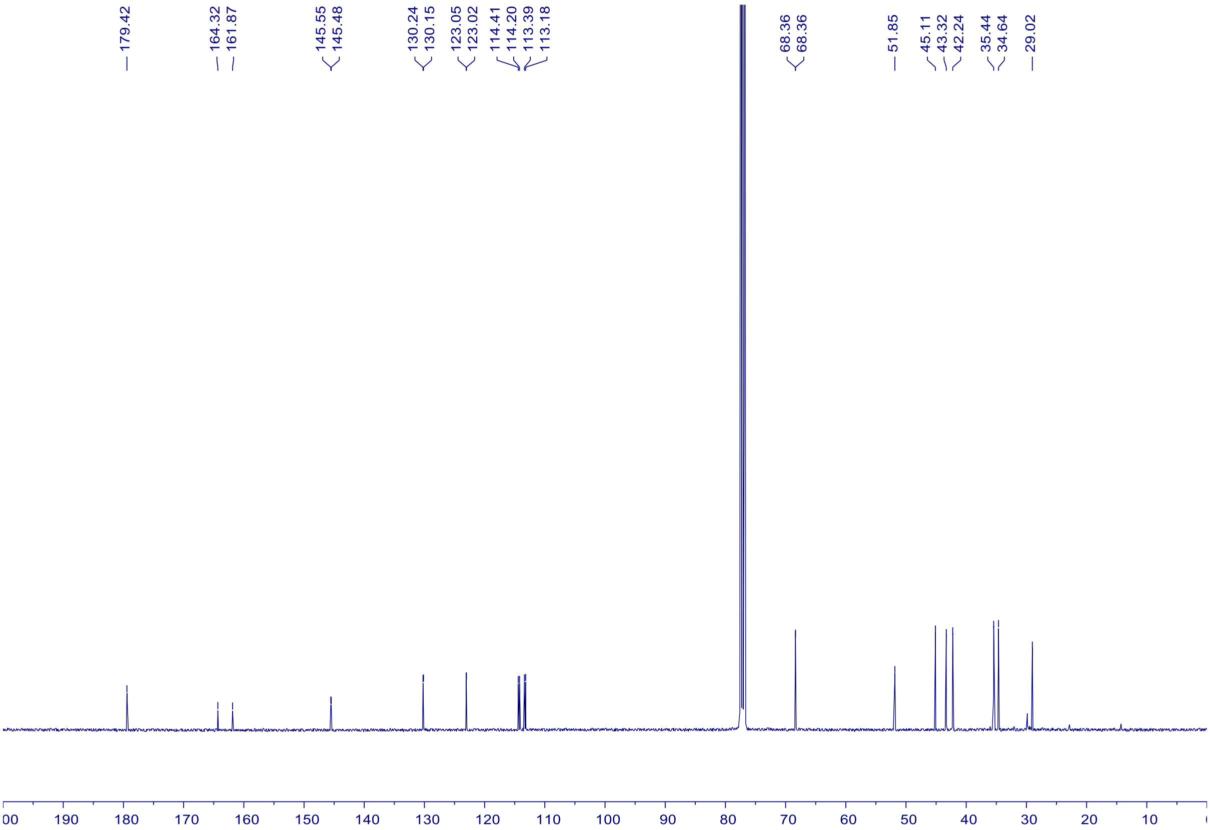

35 ^{19}F NMR (376 MHz, $CDCl_3$)

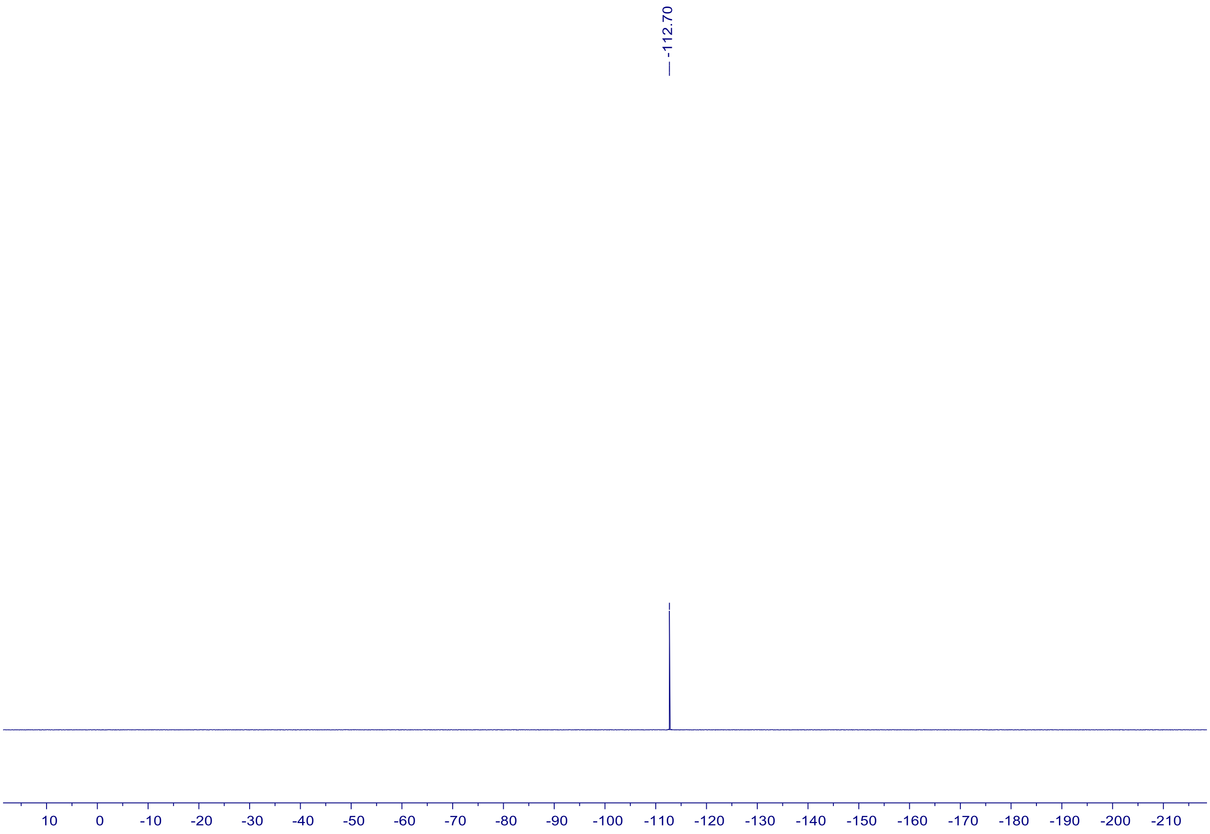
-112.70
10 0 -10 -20 -30 -40 -50 -60 -70 -80 -90 -100 -110 -120 -130 -140 -150 -160 -170 -180 -190 -200 -210

36 ^{1}H NMR (500 MHz, $CDCl_3$)

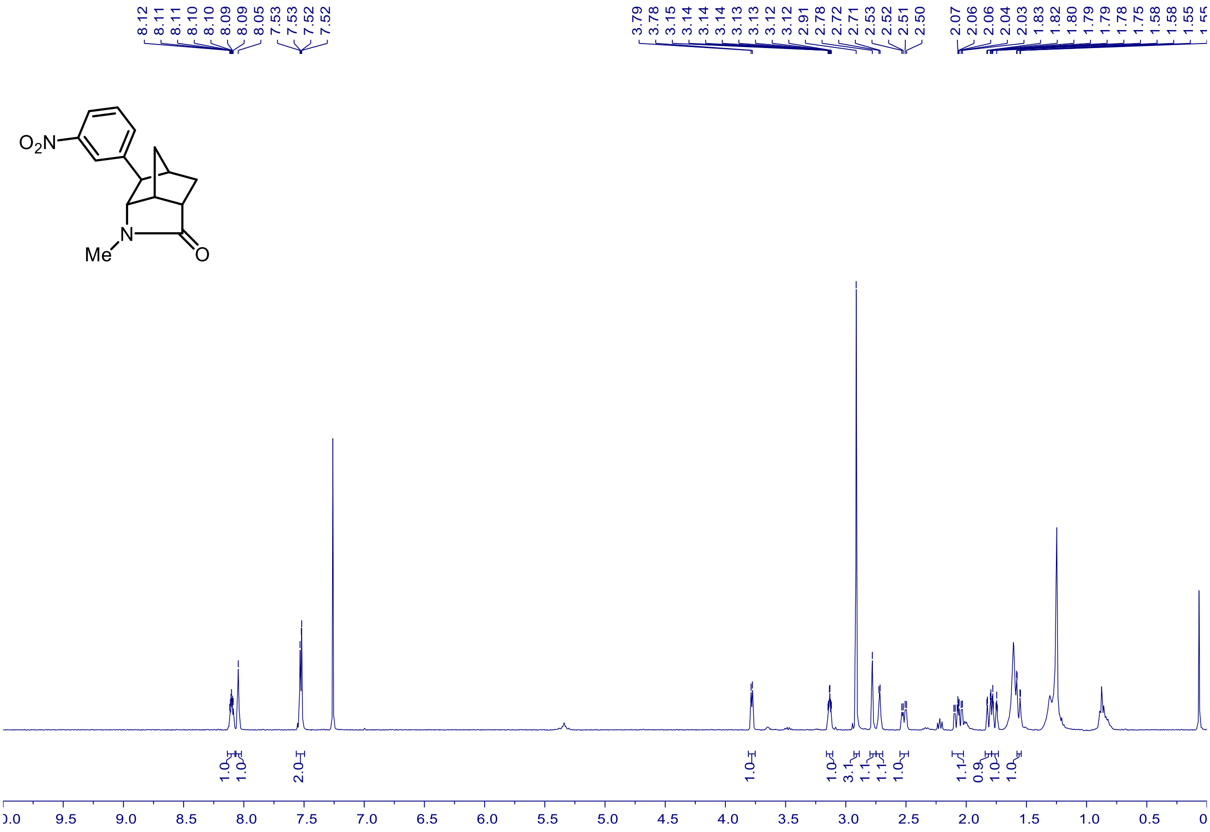

36 ^{13}C NMR (126 MHz, $CDCl_3$)

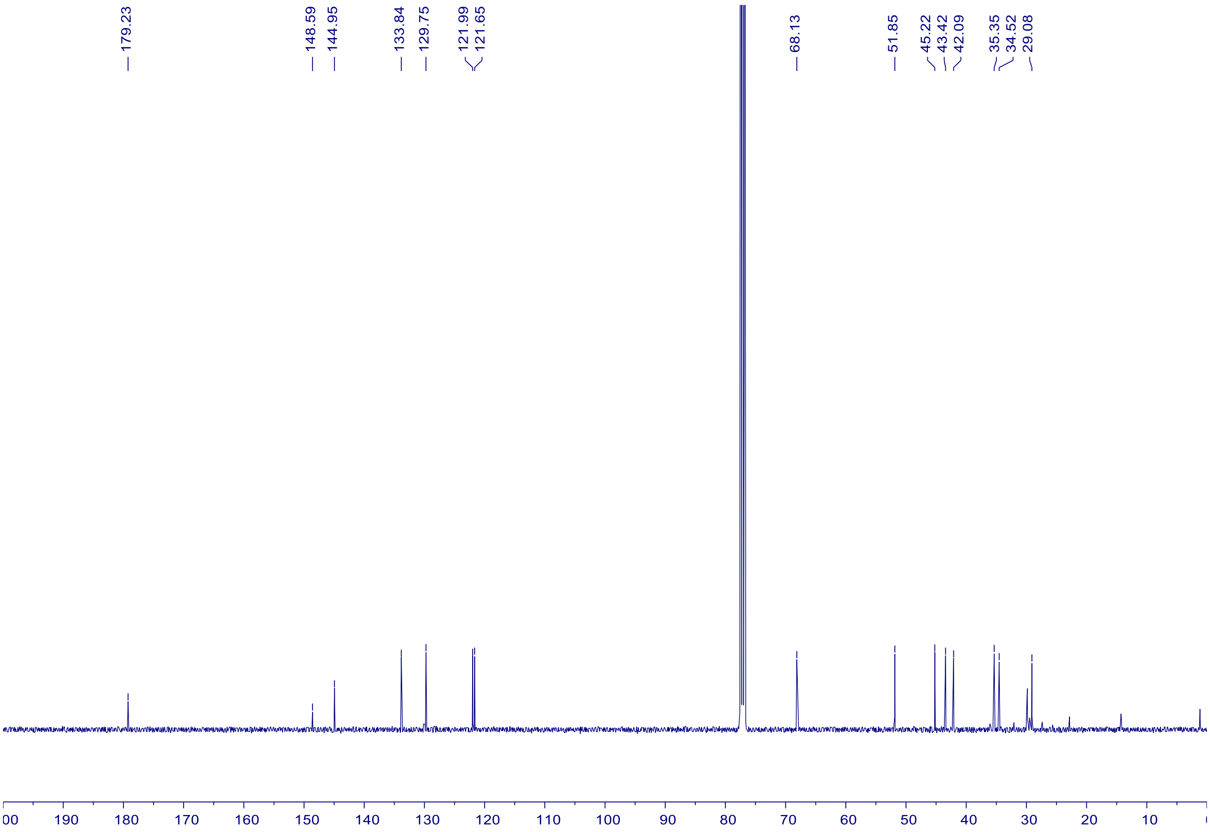

37 ^{1}H NMR (500 MHz, $CDCl_3$)

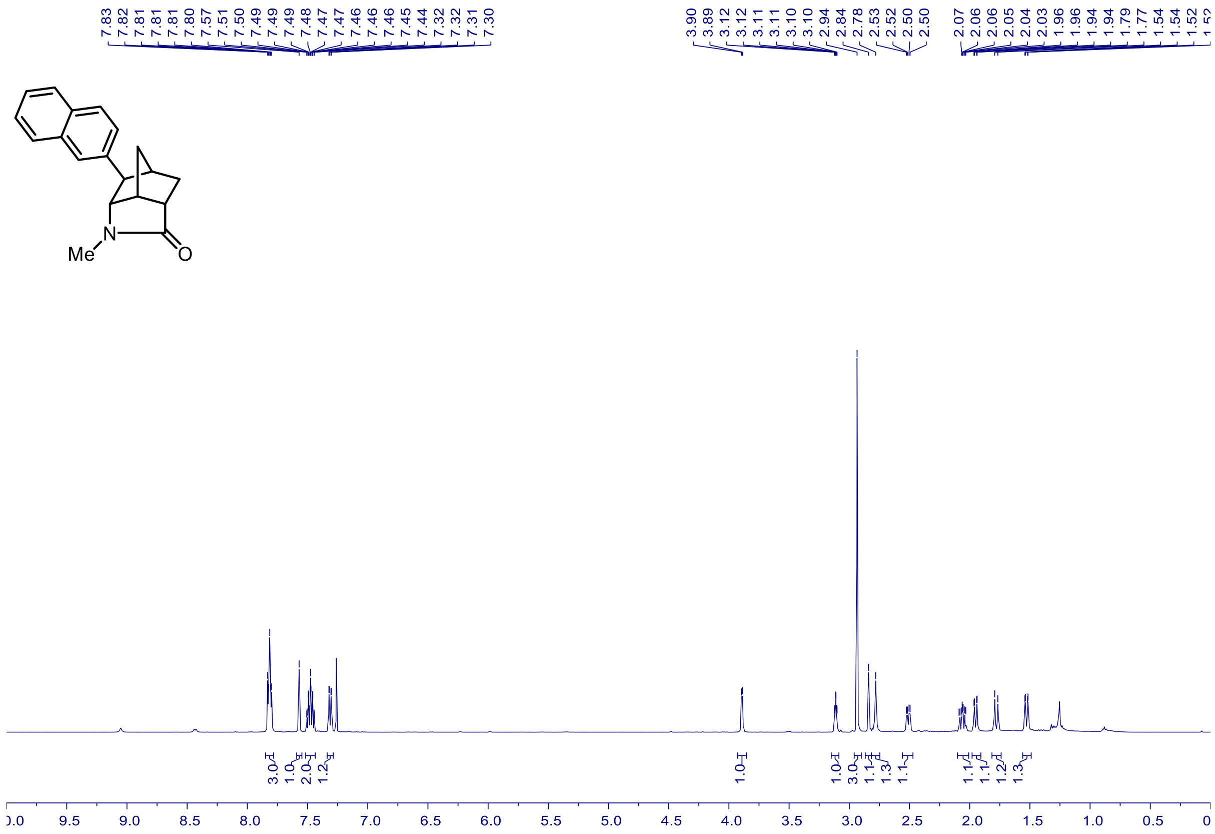

37 ^{13}C NMR (126 MHz, $CDCl_3$)

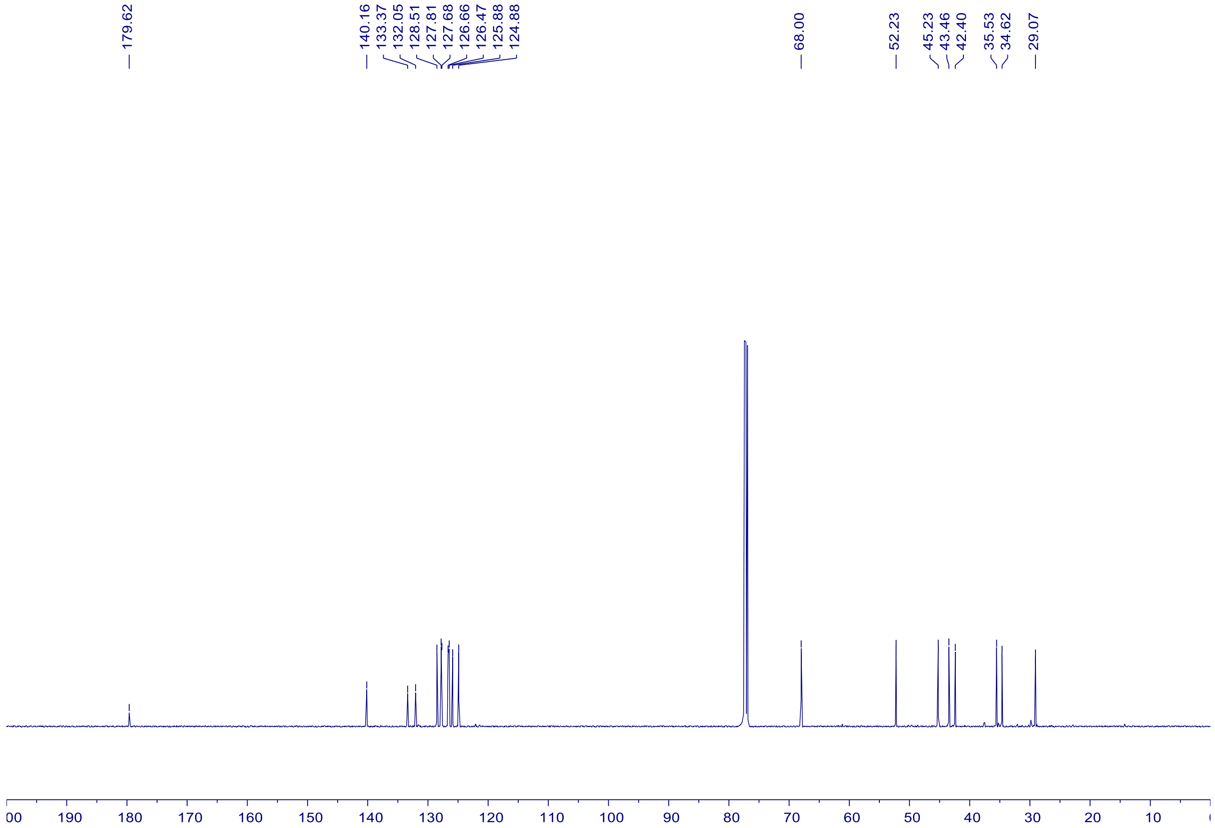

38 ^{1}H NMR (500 MHz, $CDCl_3$)

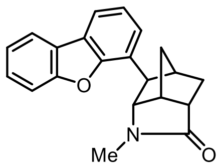

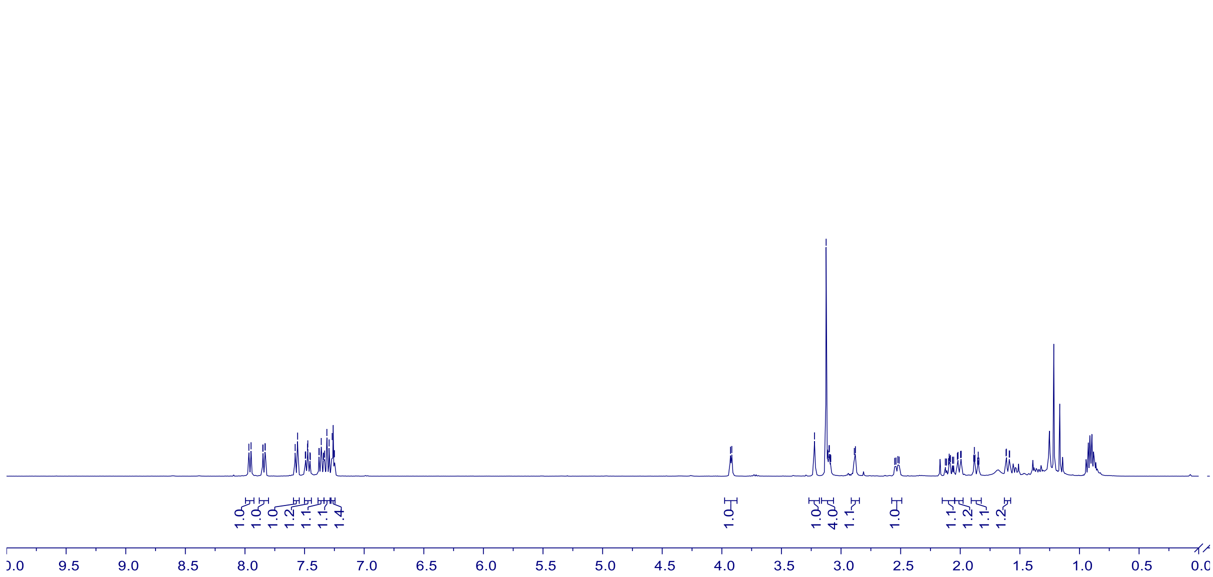

38 ^{13}C NMR (126 MHz, $CDCl_3$)

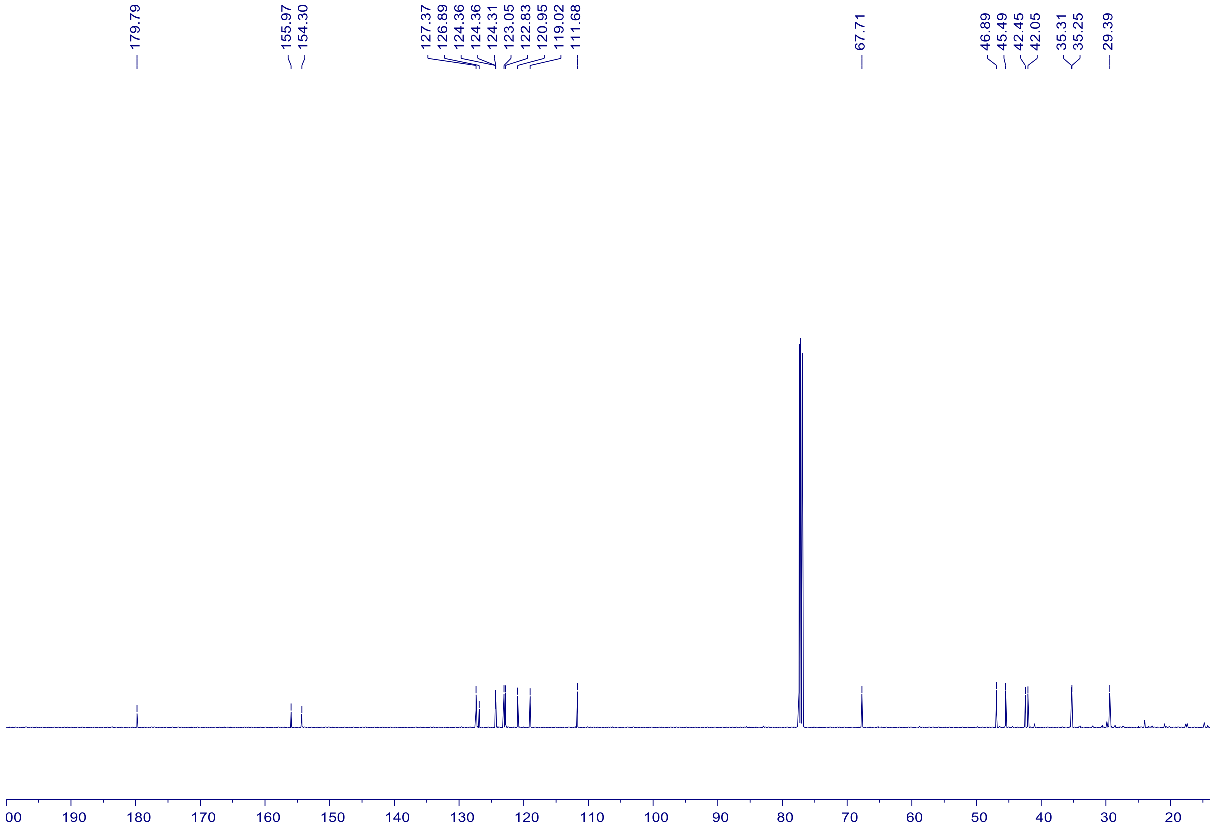

39 ^{1}H NMR (500 MHz, $CDCl_3$)

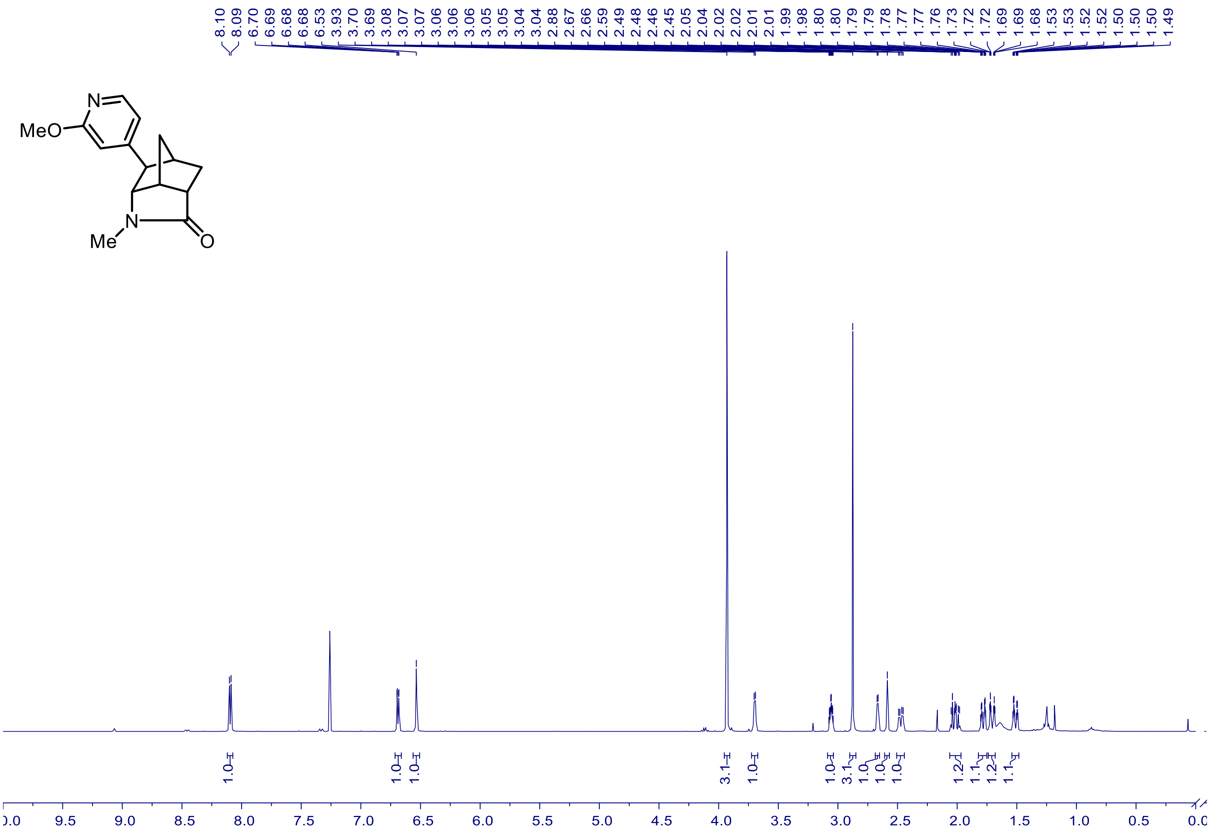

39 ^{13}C NMR (126 MHz, $CDCl_3$)

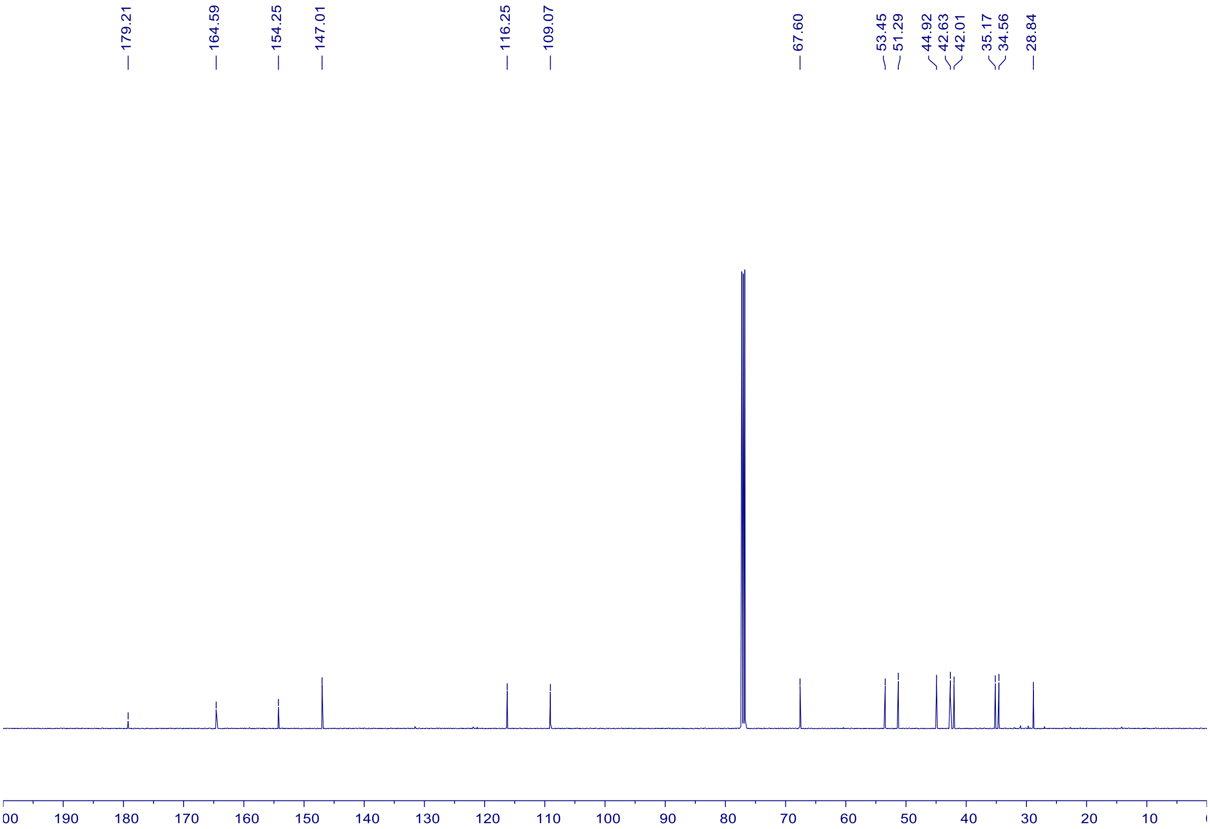

40 ^{1}H NMR (500 MHz, $CDCl_3$)

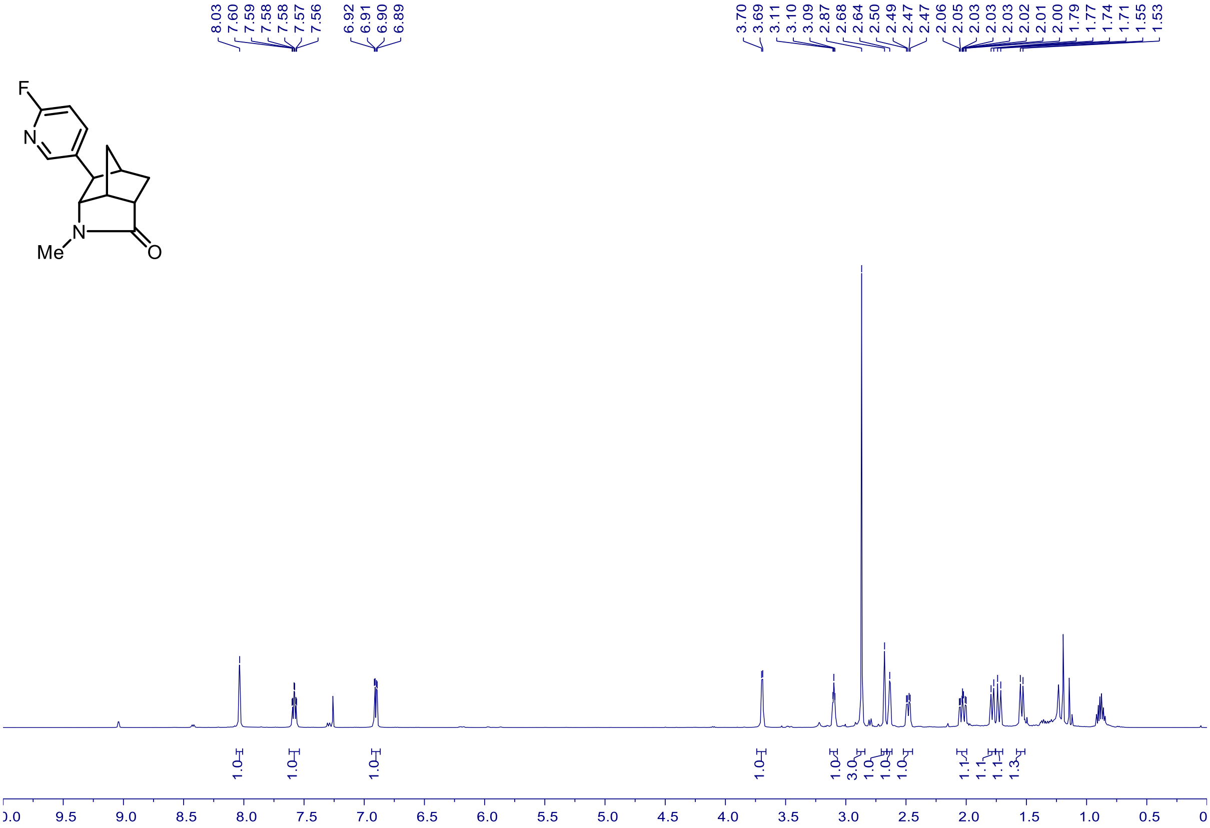

40 ^{13}C NMR (126 MHz, CDCl$_3$)

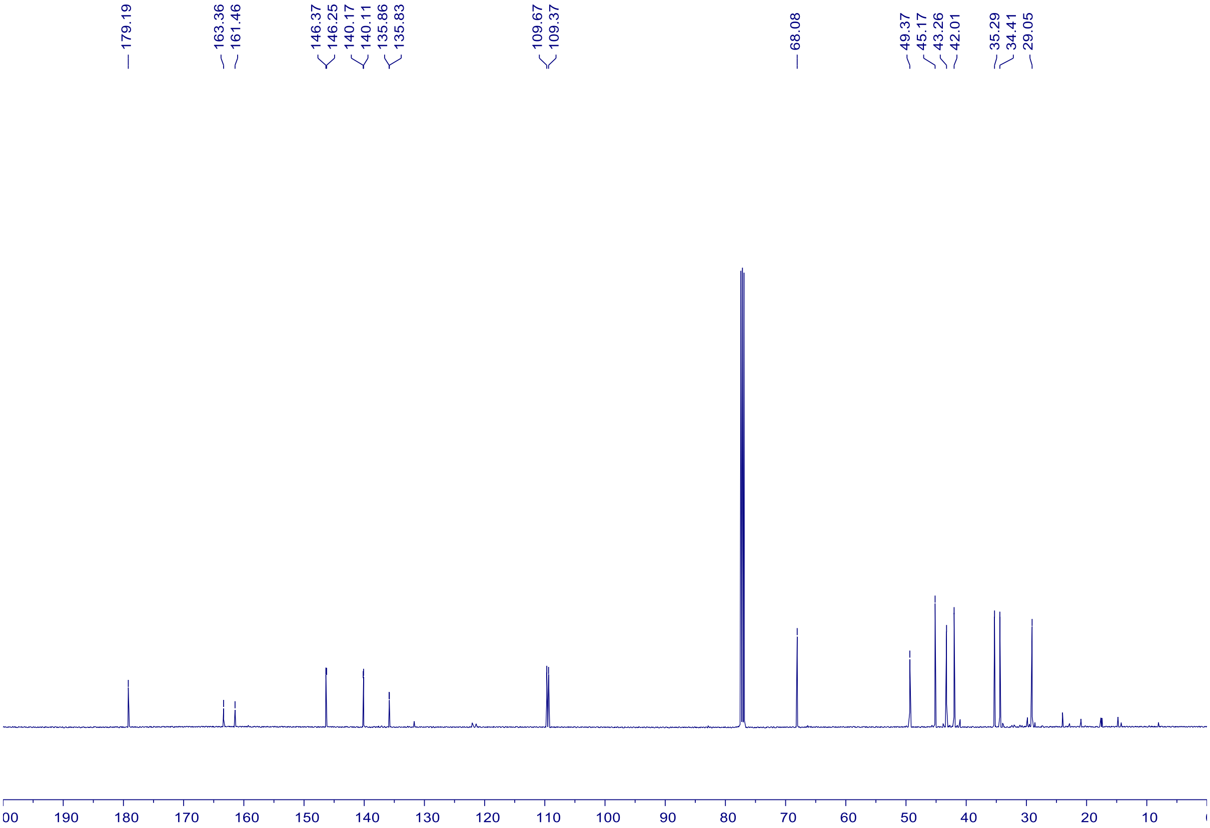

40 ^{19}F NMR (376 MHz, CDCl$_3$)

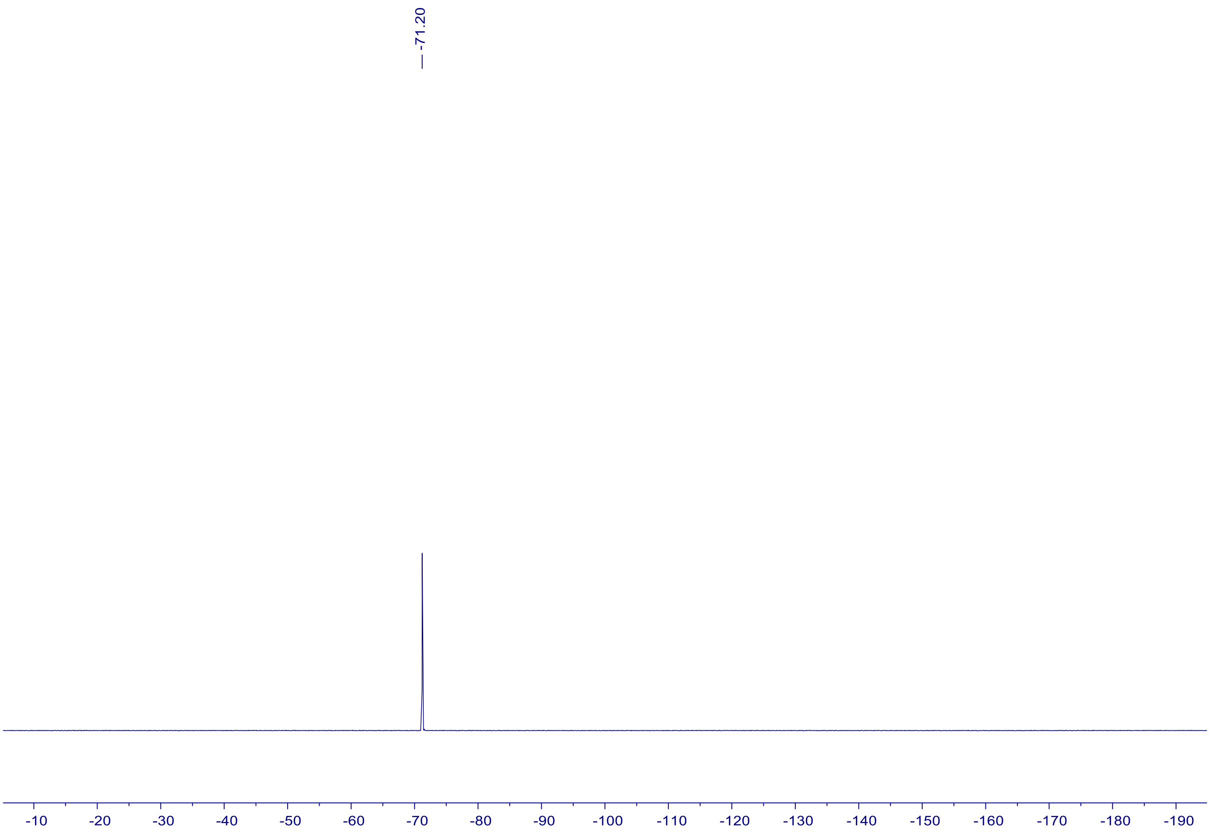
-71.20
-10
-20
-30
-40
-50
-60
-70
-80
-90
-100
-110
-120
-130
-140
-150
-160
-170
-180
-190

41 ^{1}H NMR (500 MHz, CDCl$_3$)

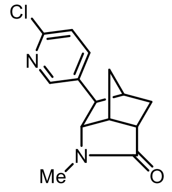

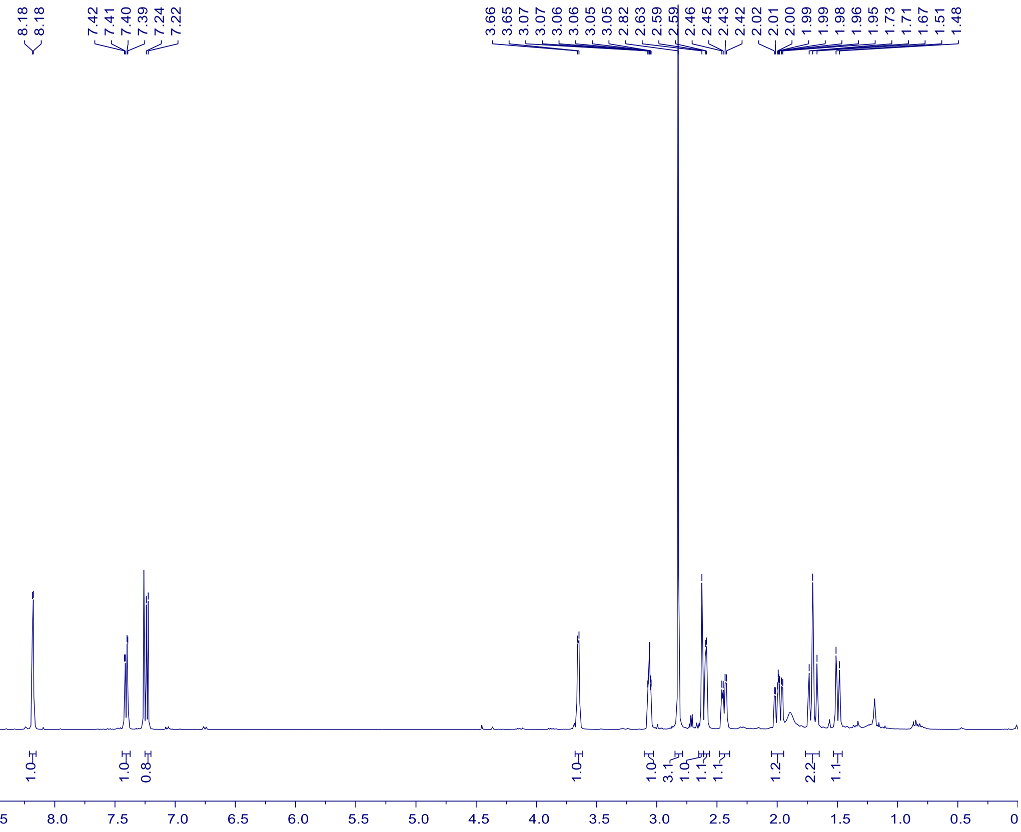

41 ^{13}C NMR (126 MHz, CDCl$_3$)

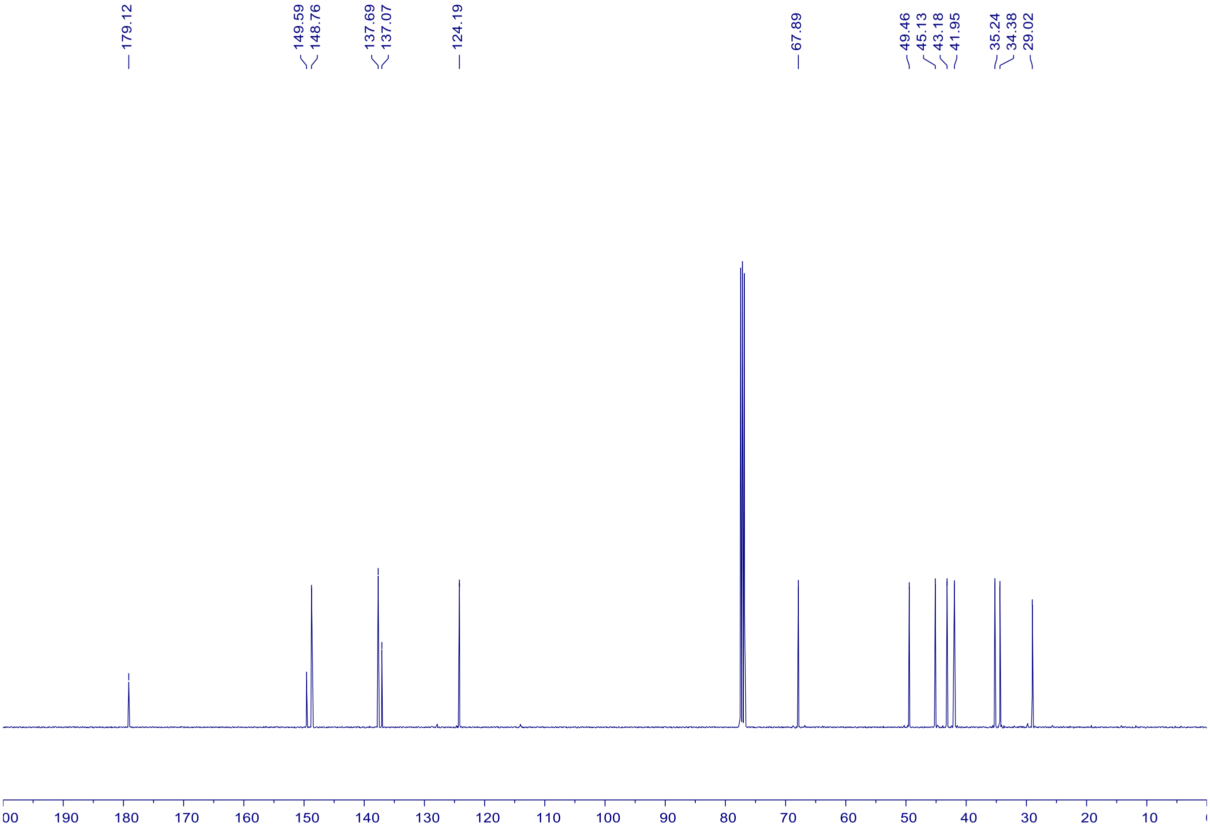

42 ^{1}H NMR (500 MHz, CDCl$_3$)

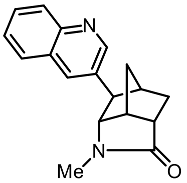

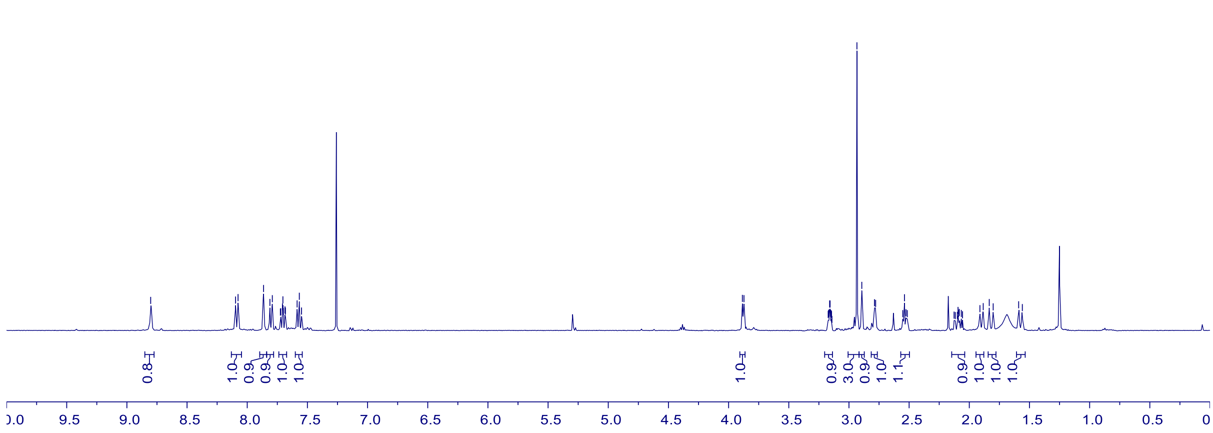

42 ^{13}C NMR (126 MHz, CDCl$_3$)

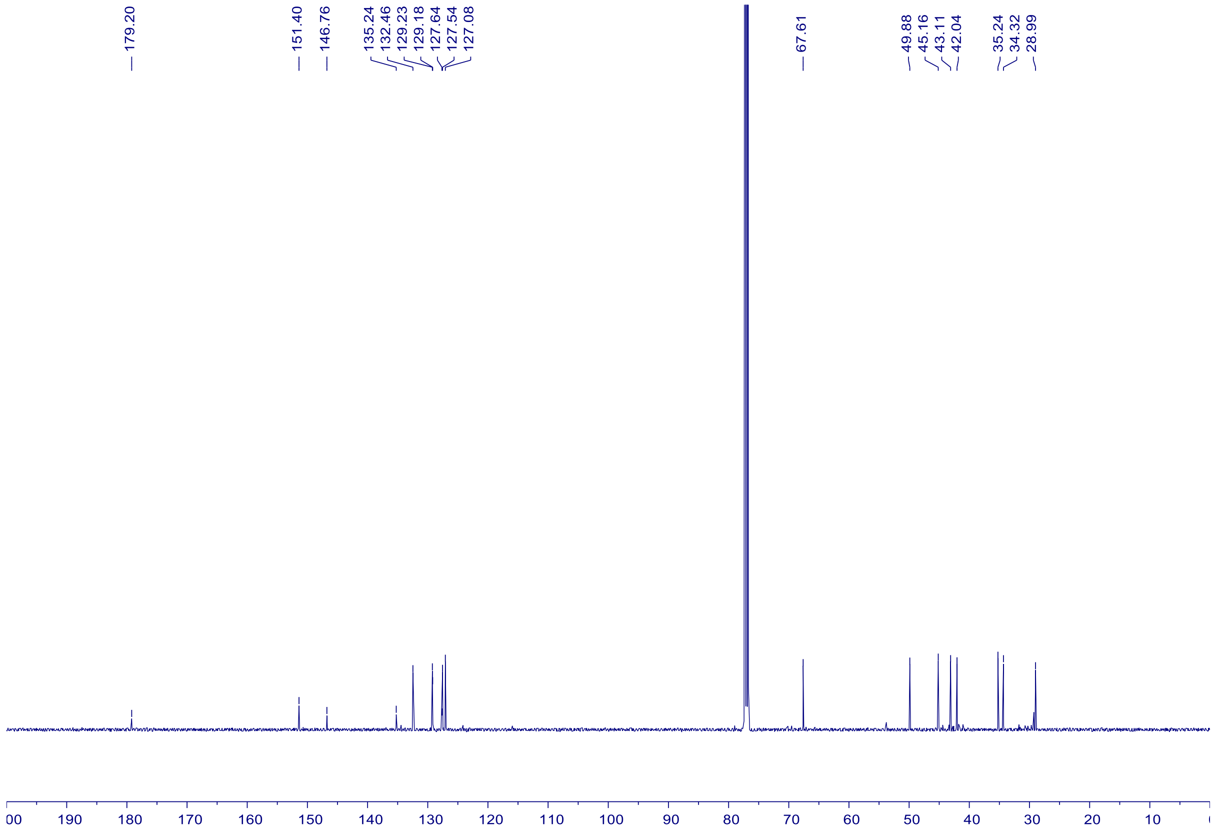

43 ^{1}H NMR (500 MHz, CDCl$_3$)

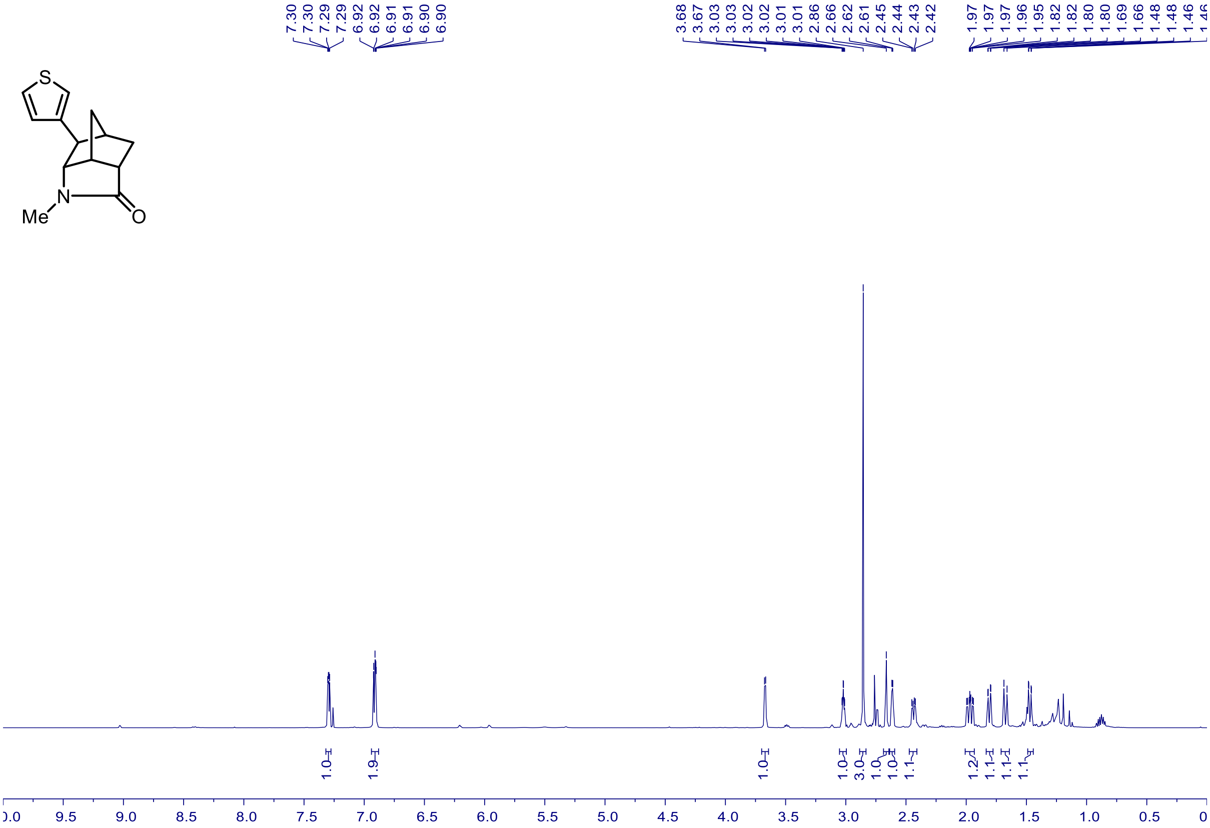

43 ^{13}C NMR (126 MHz, $CDCl_3$)

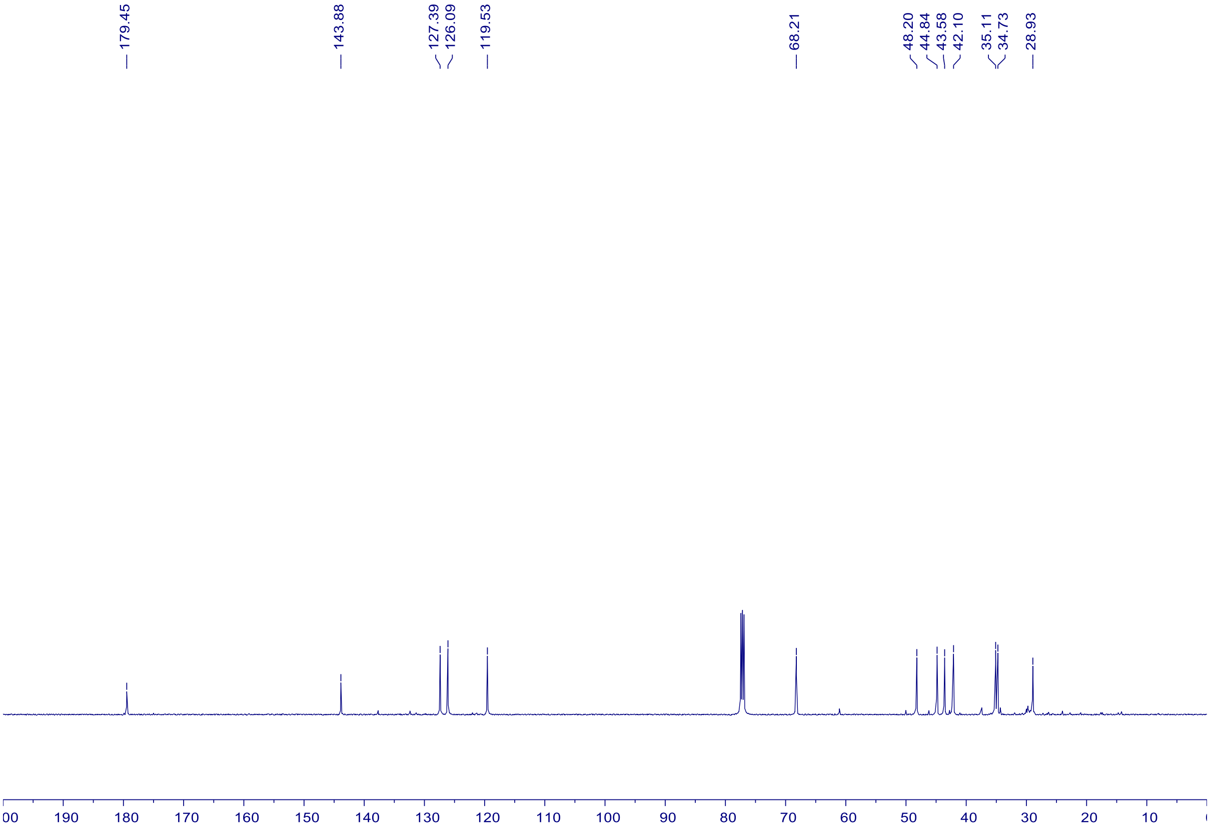

44 ^{1}H NMR (500 MHz, $CDCl_3$)

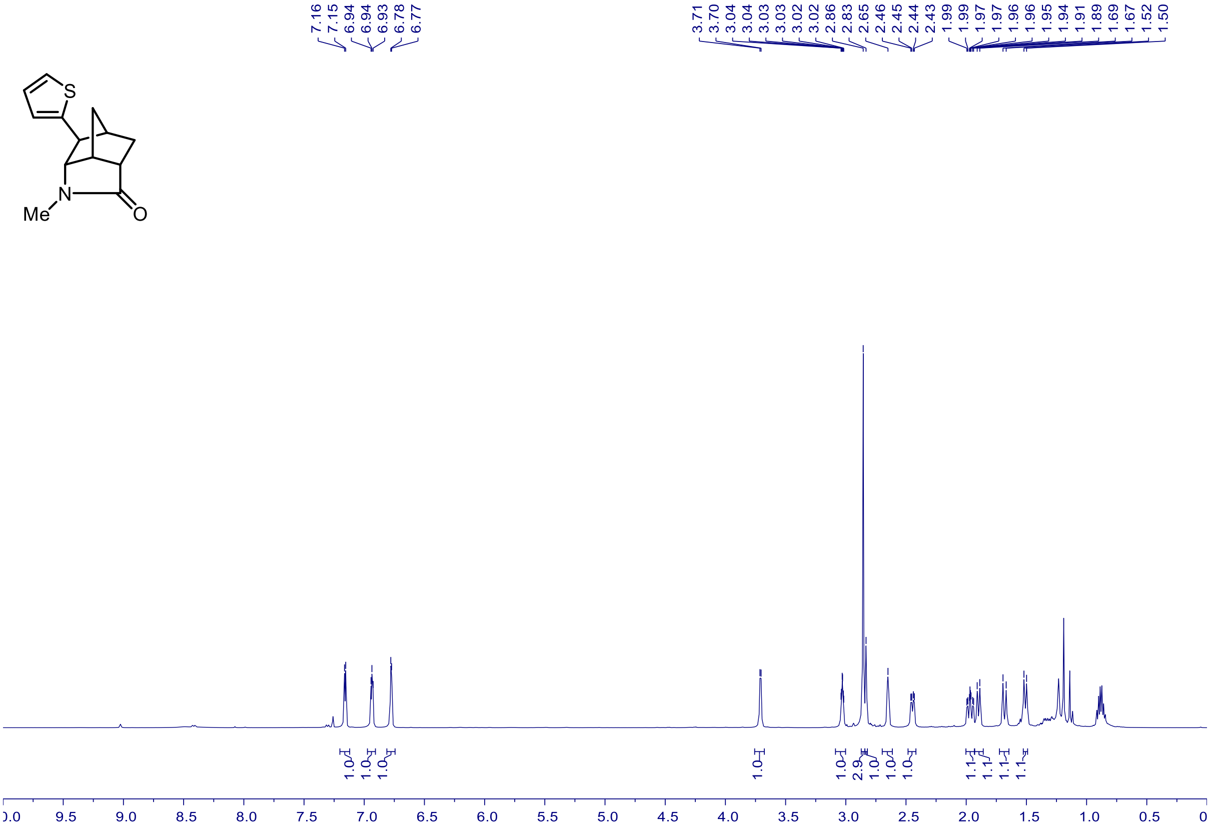

44 ^{13}C NMR (126 MHz, $CDCl_3$)

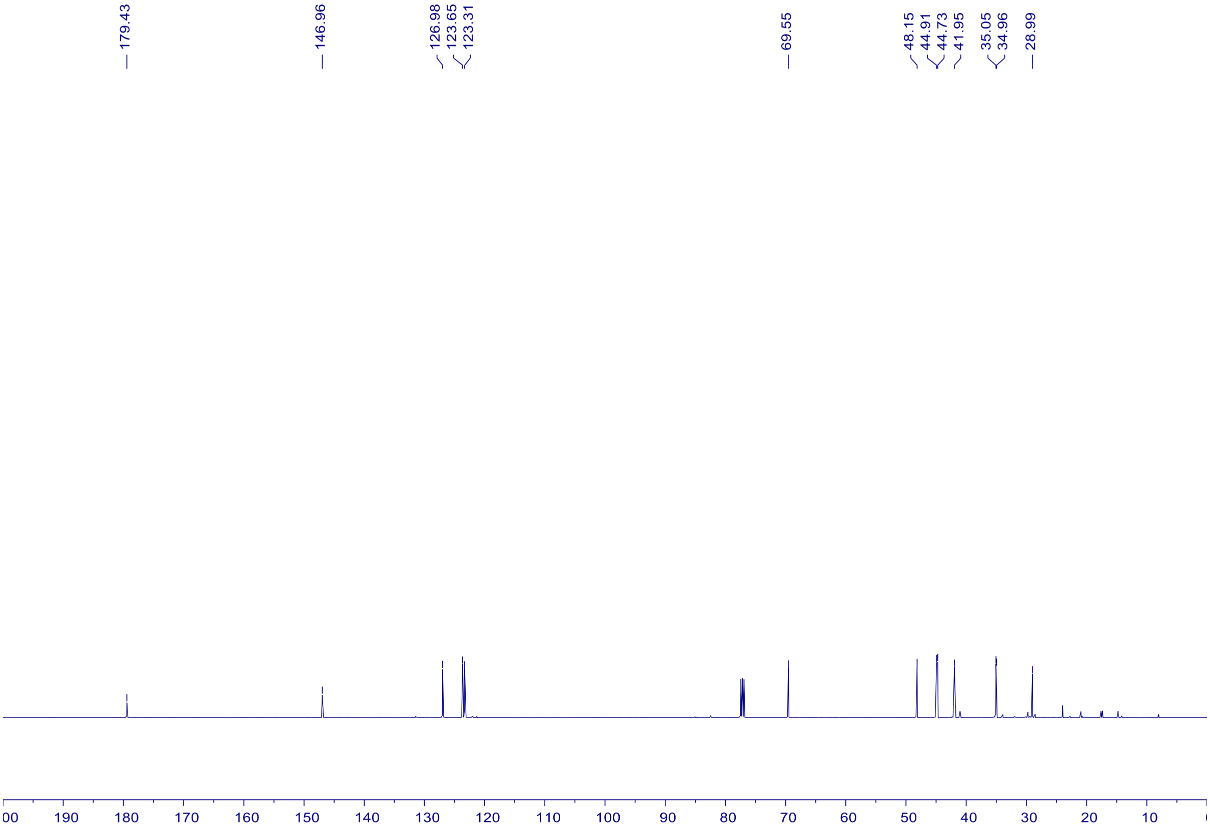

45 ^{1}H NMR (500 MHz, $CDCl_3$)

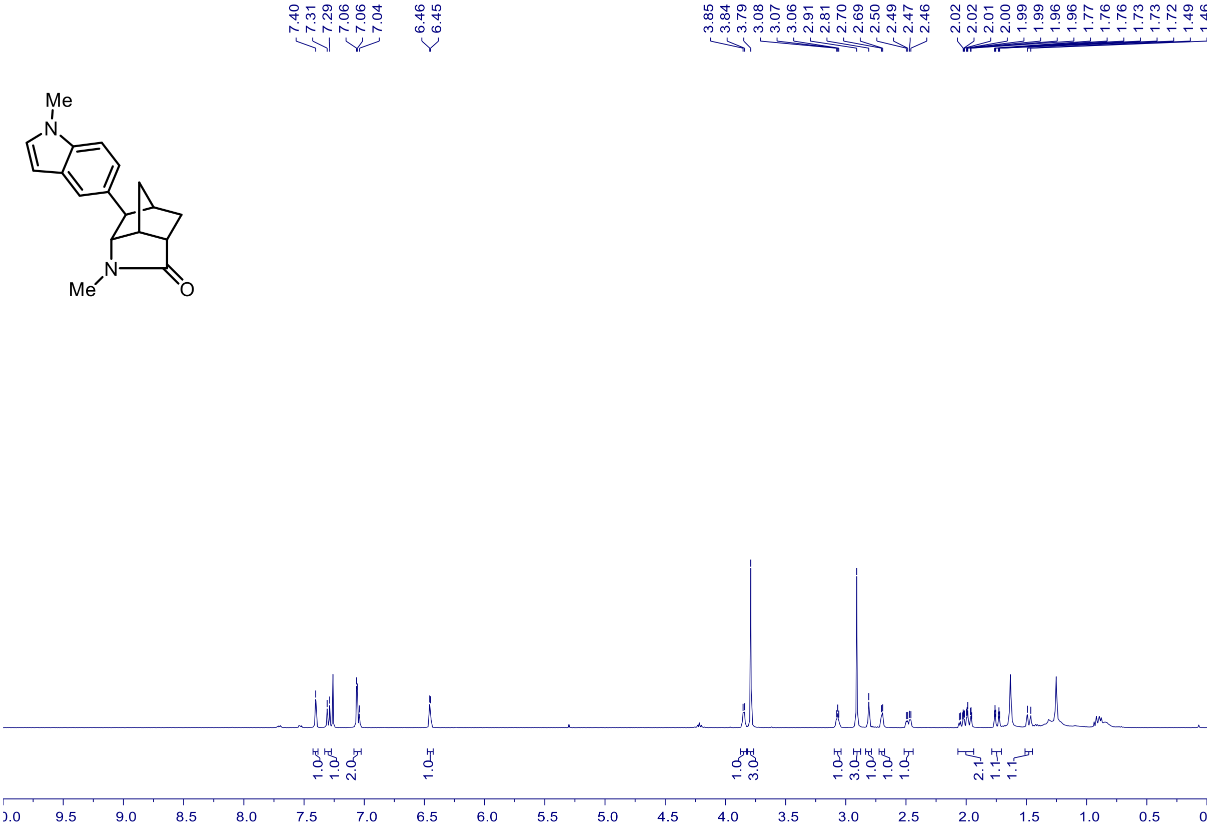

45 ^{13}C NMR (126 MHz, $CDCl_3$)

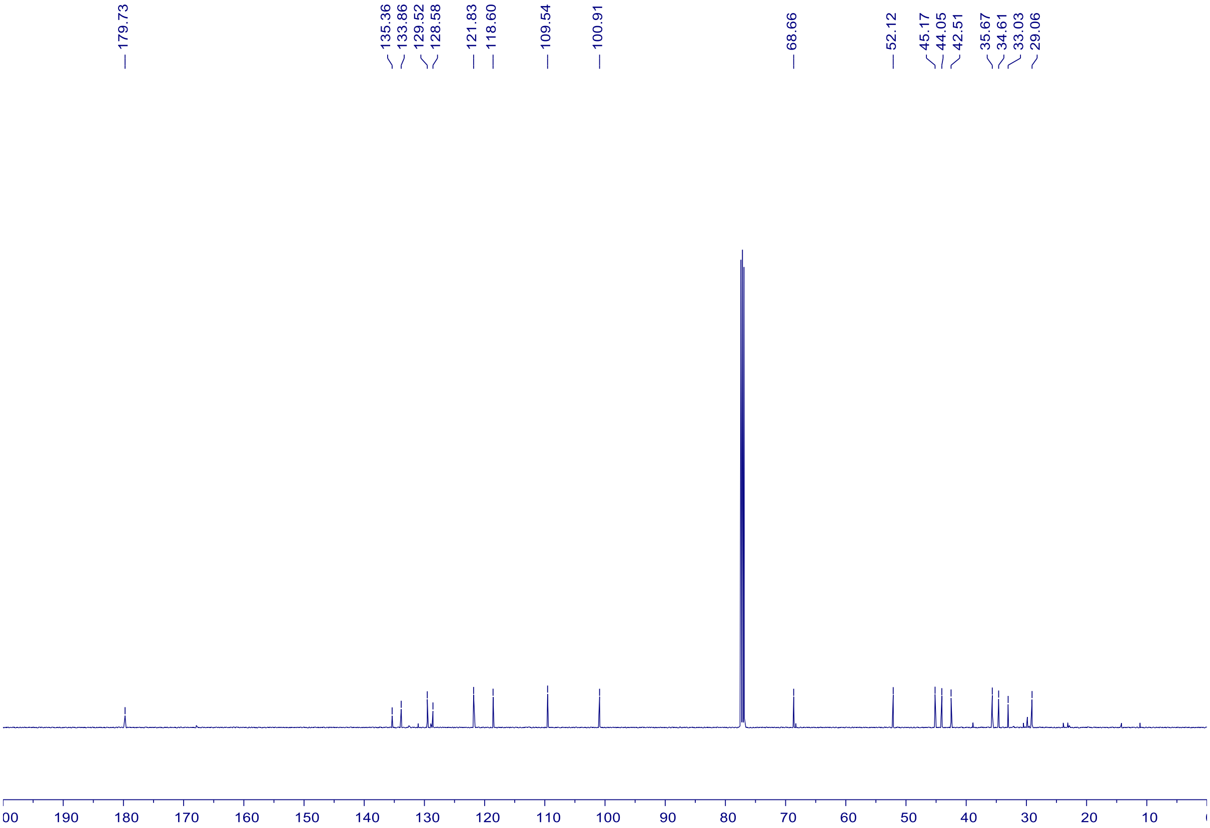

46 ^{1}H NMR (500 MHz, $CDCl_3$)

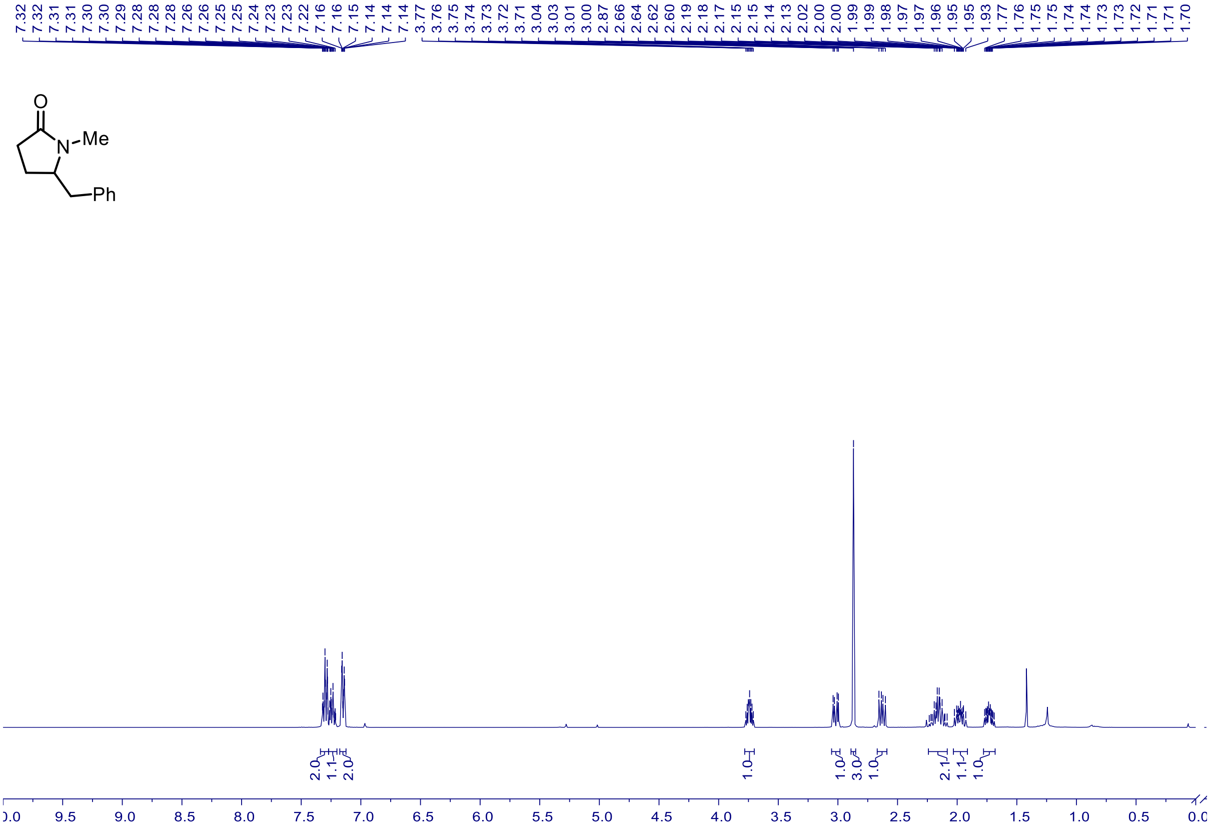

46 ^{13}C NMR (126 MHz, $CDCl_3$)

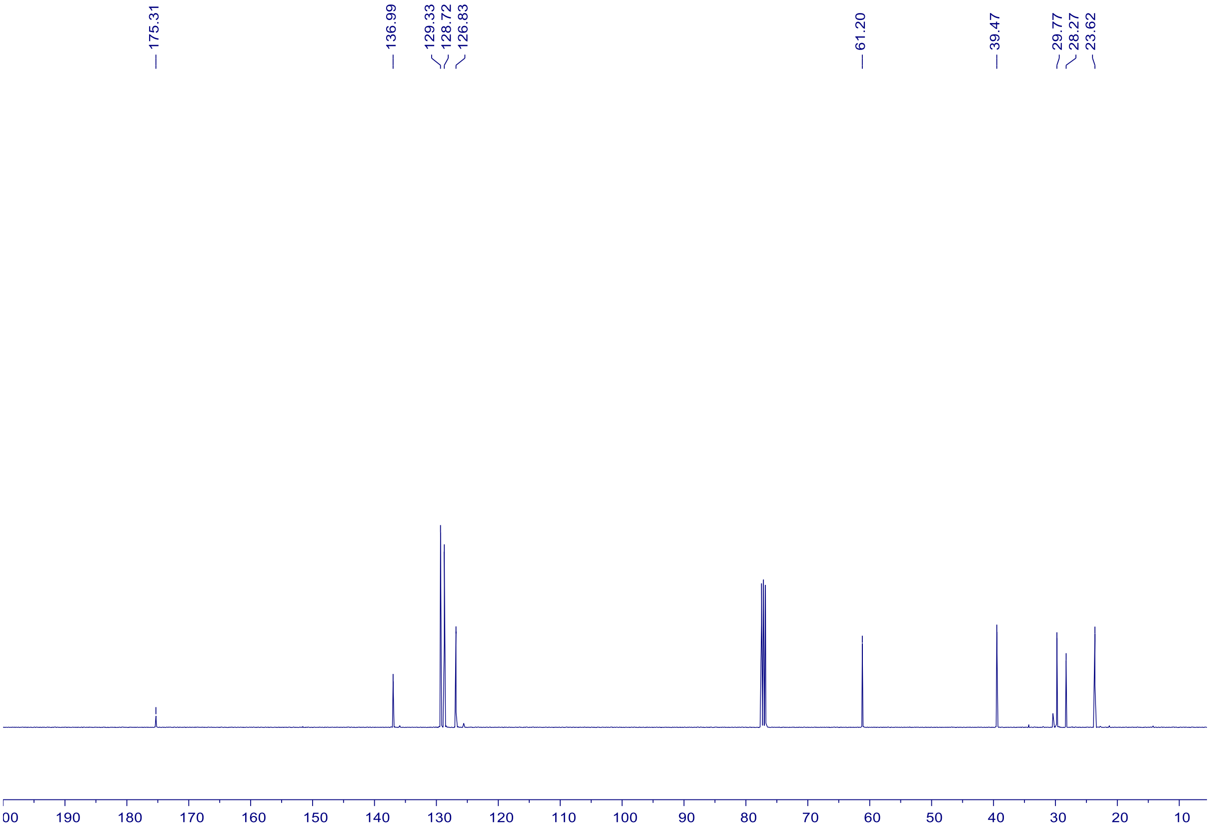

47 ^{1}H NMR (500 MHz, $CDCl_3$)

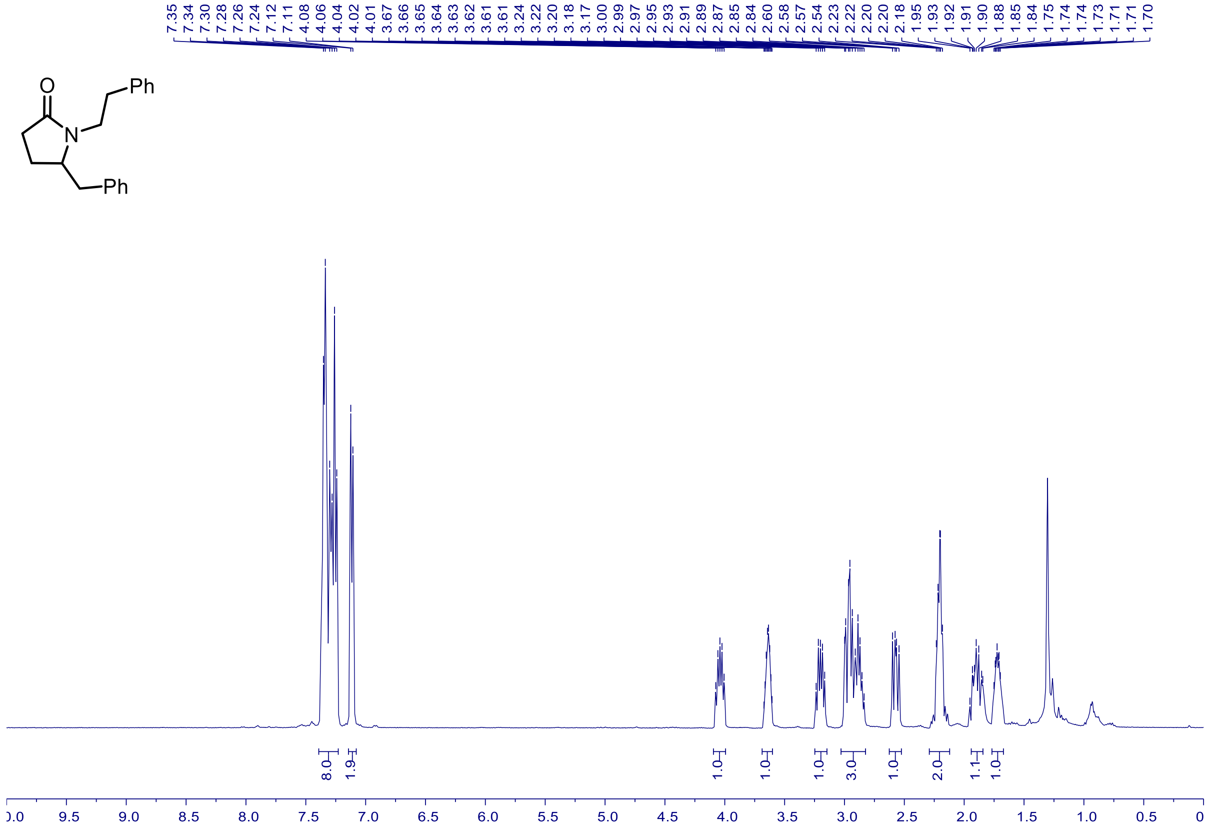

47 ^{13}C NMR (126 MHz, $CDCl_3$)

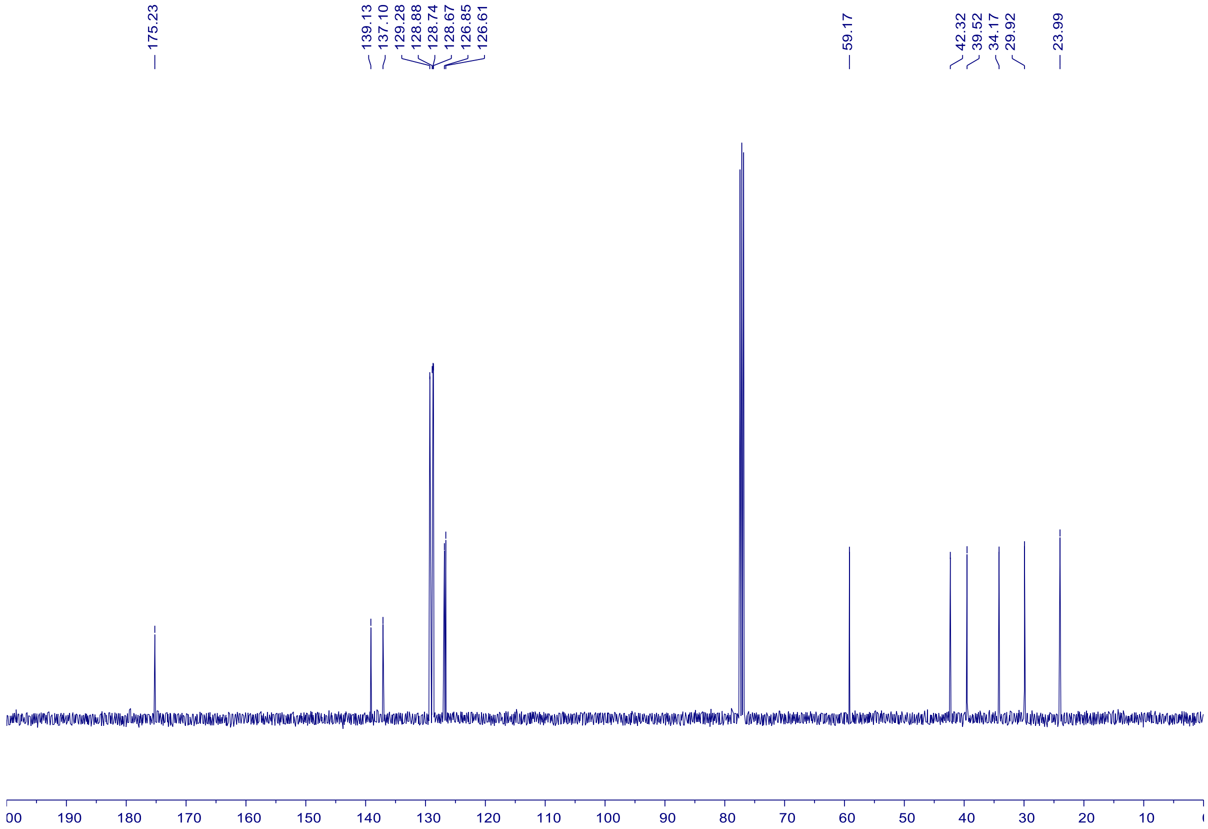

48 ^{1}H NMR (500 MHz, $CDCl_3$)

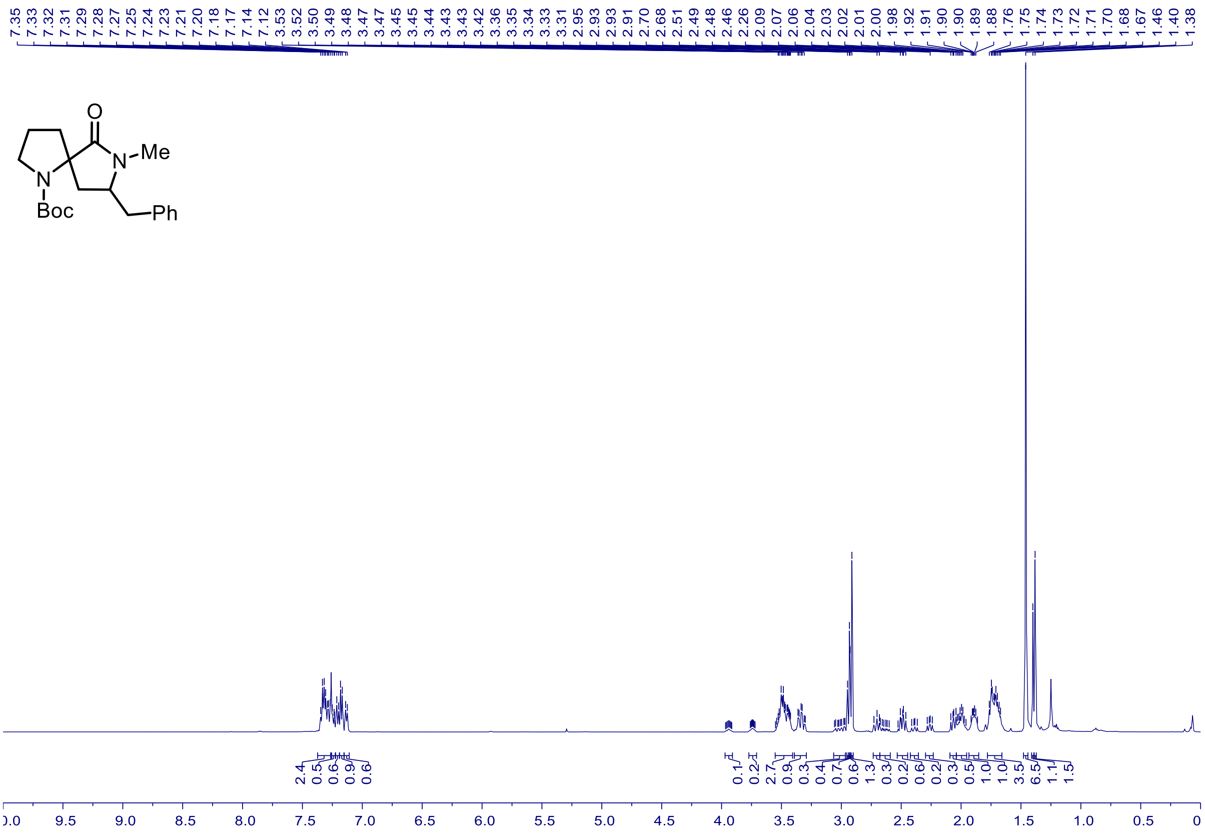

48 ^{13}C NMR (126 MHz, $CDCl_3$)

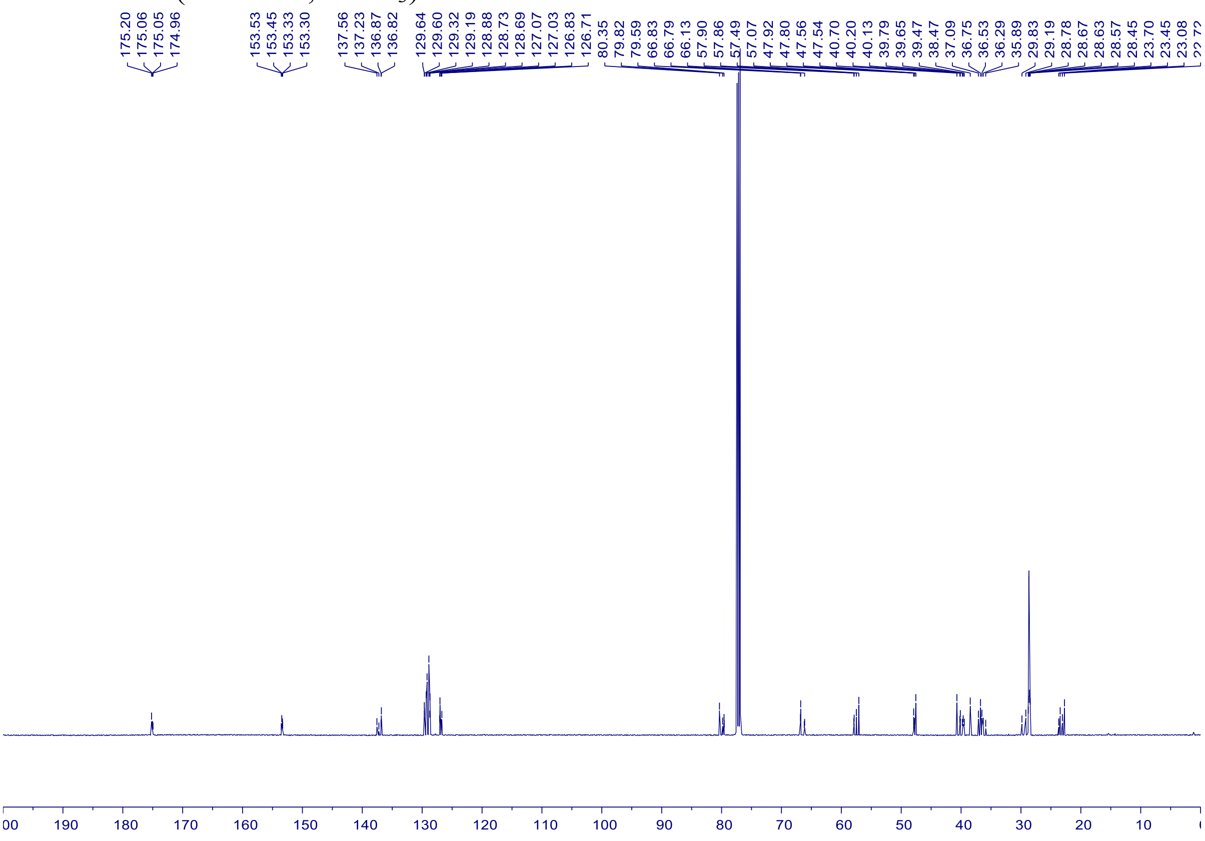

49 ^{1}H NMR (500 MHz, $CDCl_3$)

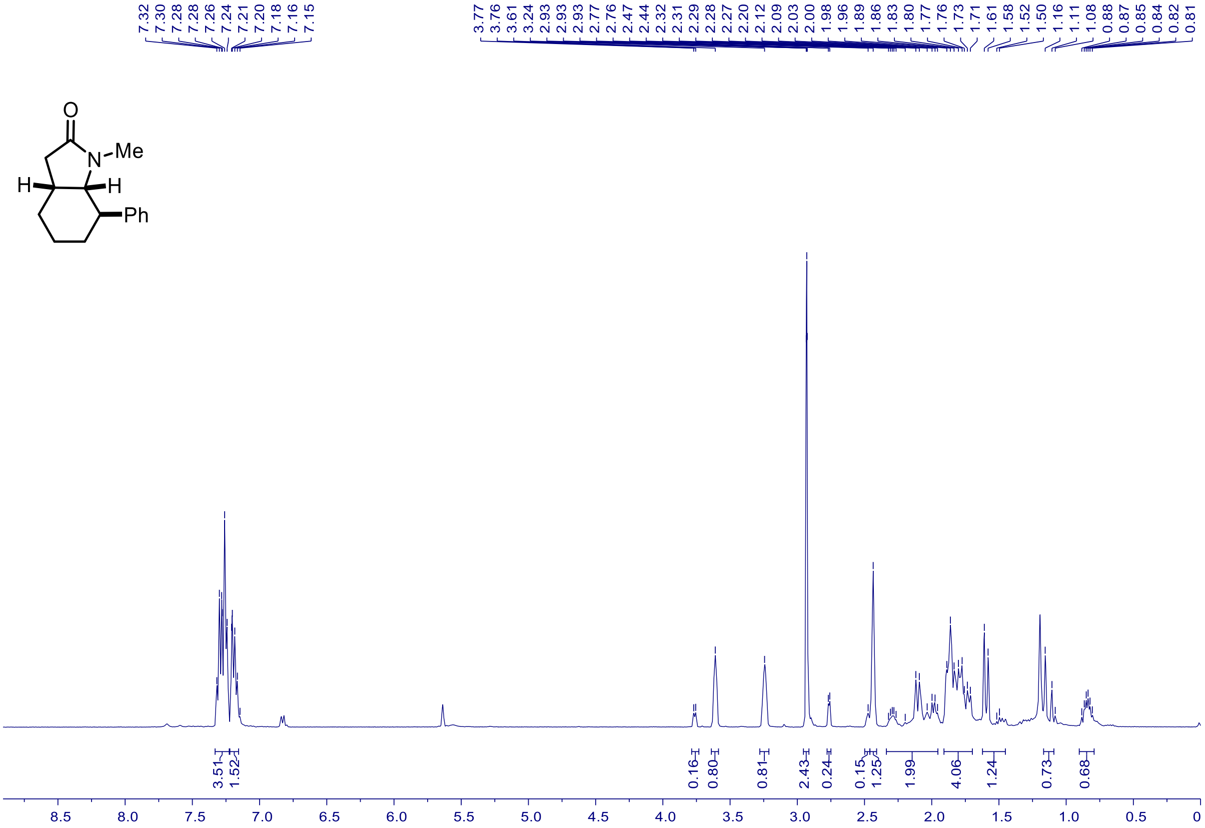

49 ^{13}C NMR (126 MHz, $CDCl_3$)

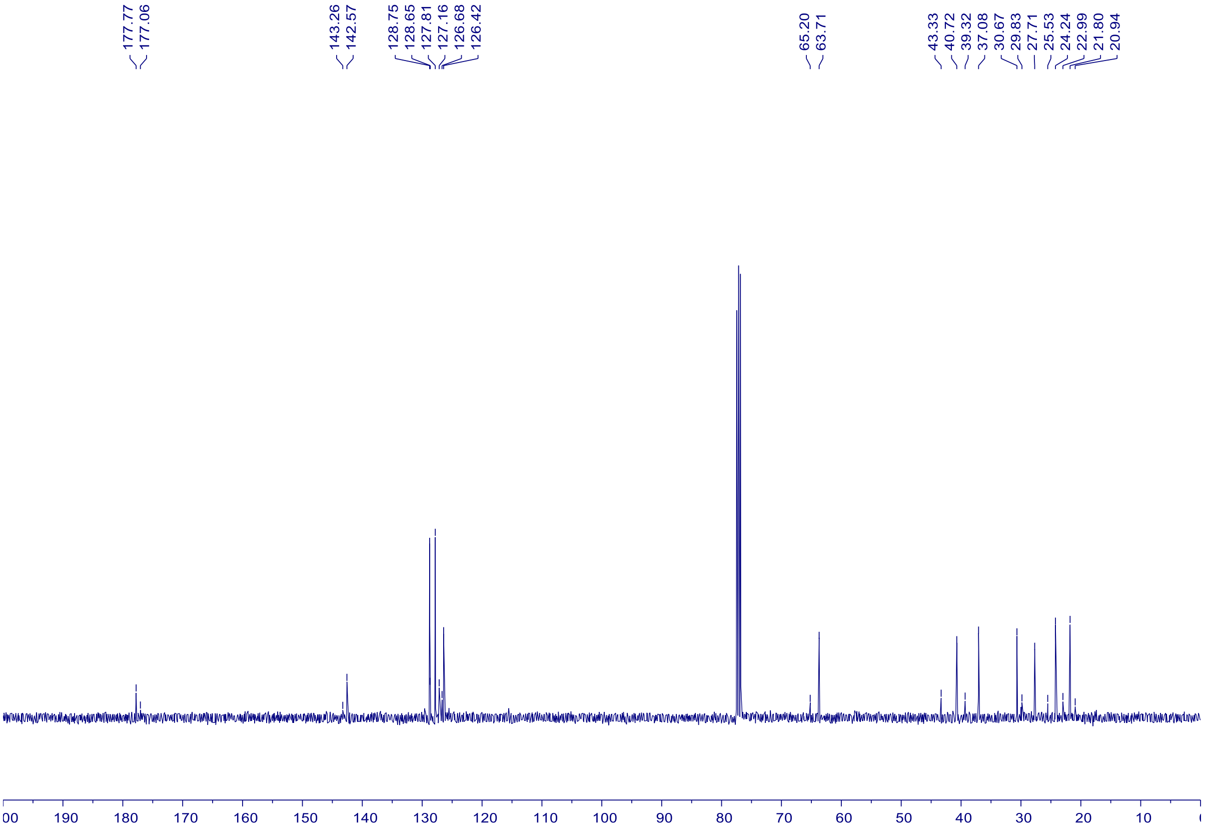

50 ^{1}H NMR (500 MHz, $CDCl_3$)

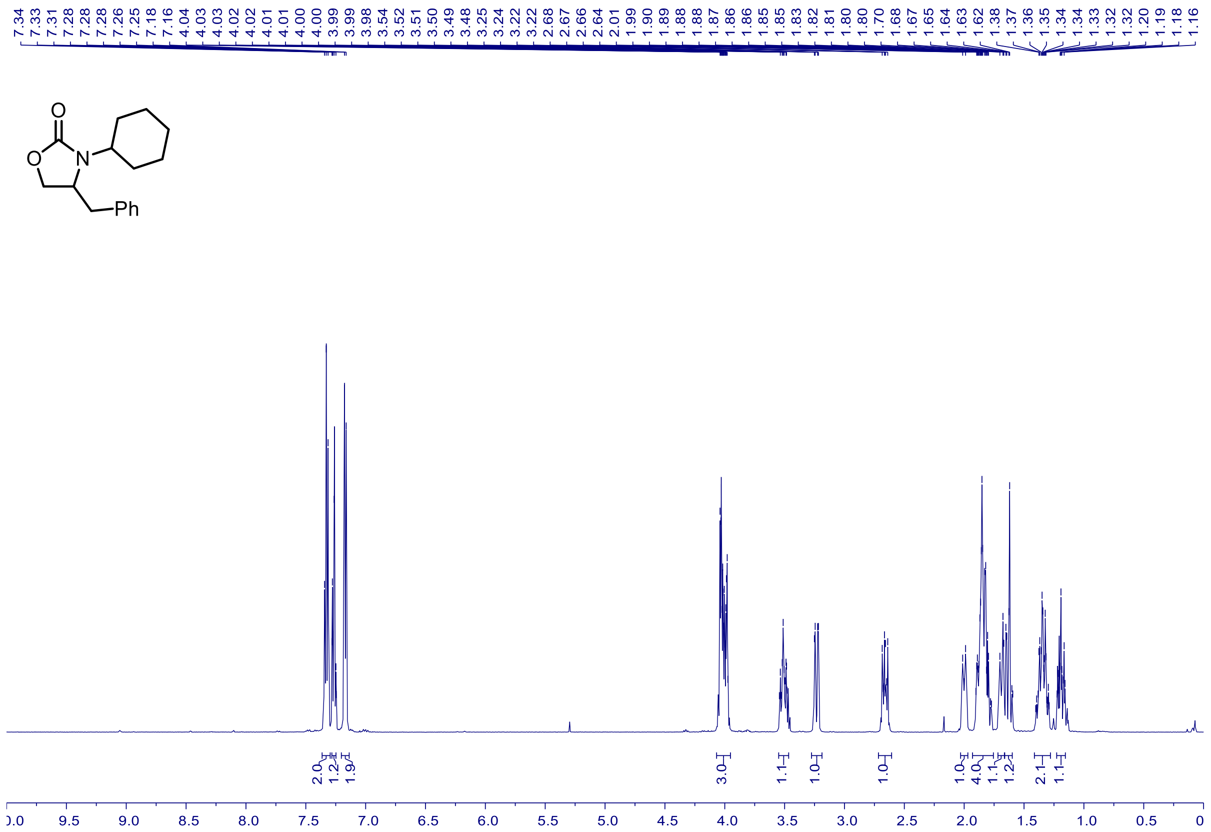

50 ^{13}C NMR (126 MHz, $CDCl_3$)

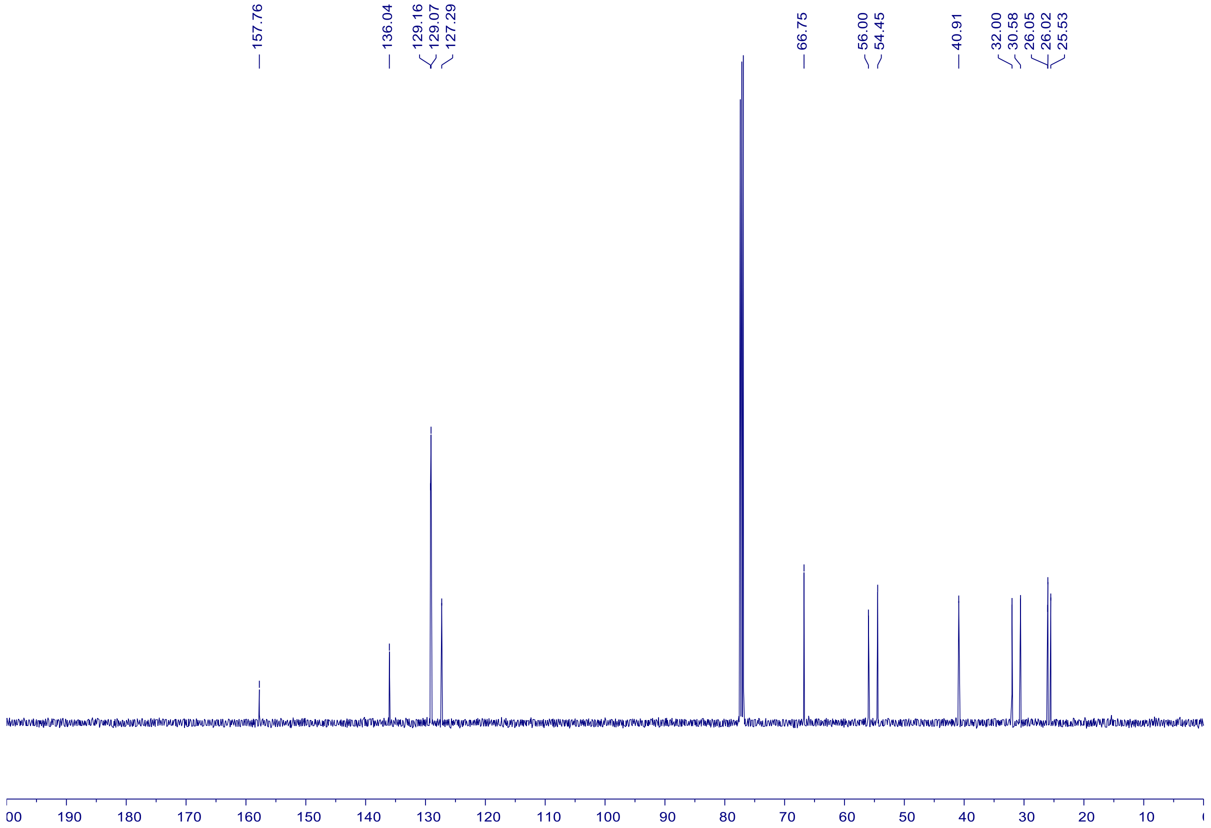

51 ^{1}H NMR (500 MHz, $CDCl_3$)

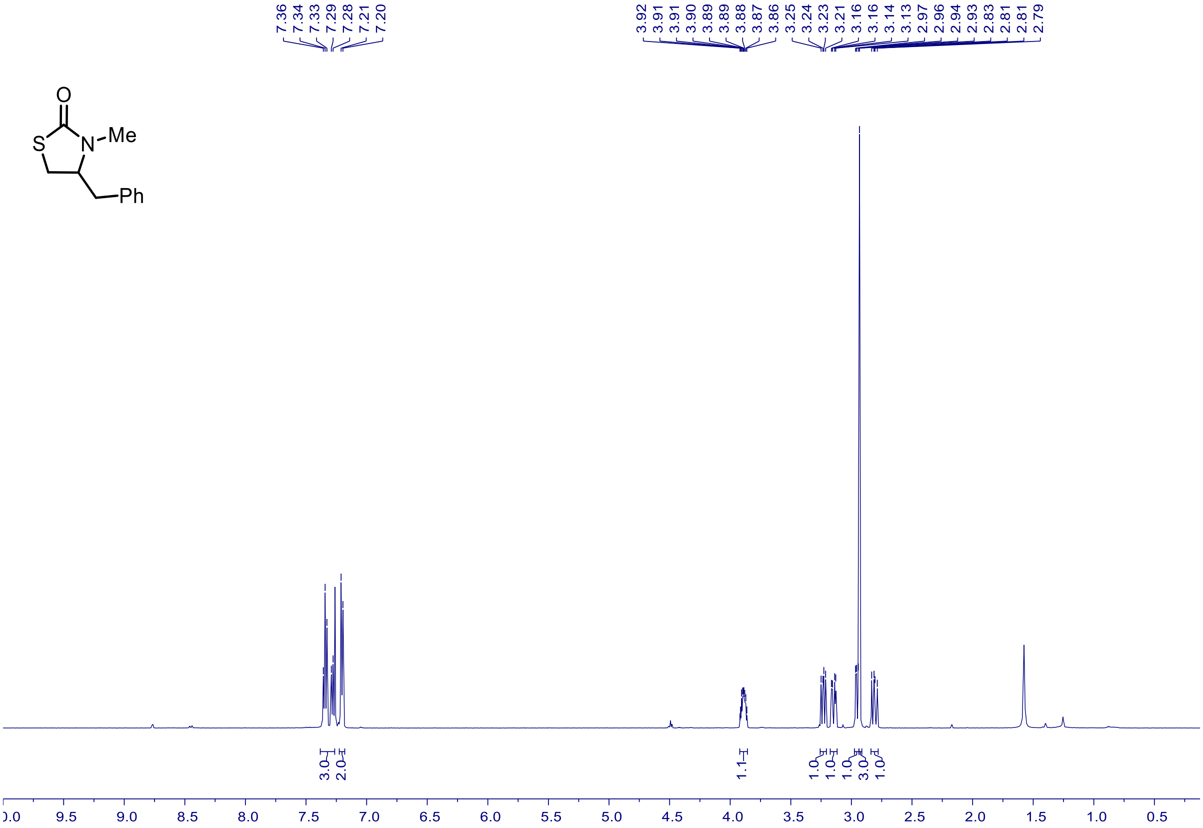

51 ^{13}C NMR (126 MHz, $CDCl_3$)

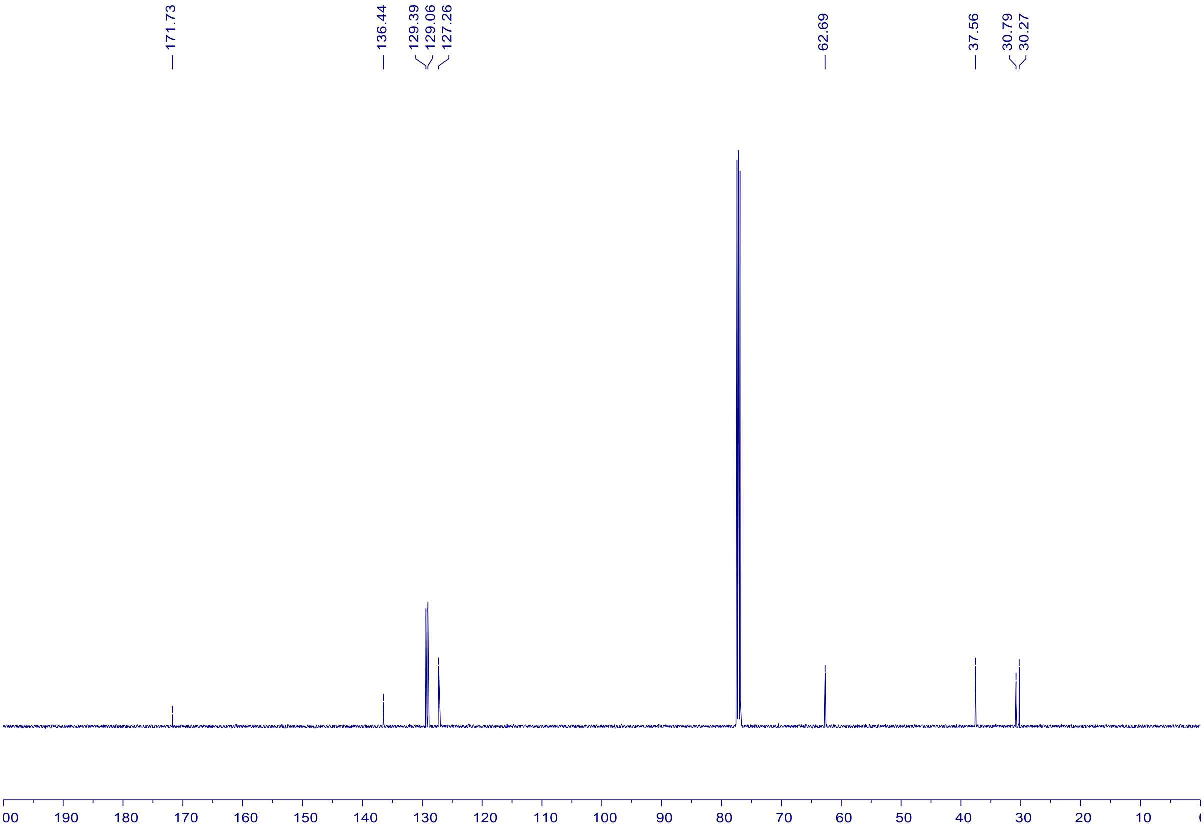

53 ^{1}H NMR (500 MHz, $CDCl_3$)

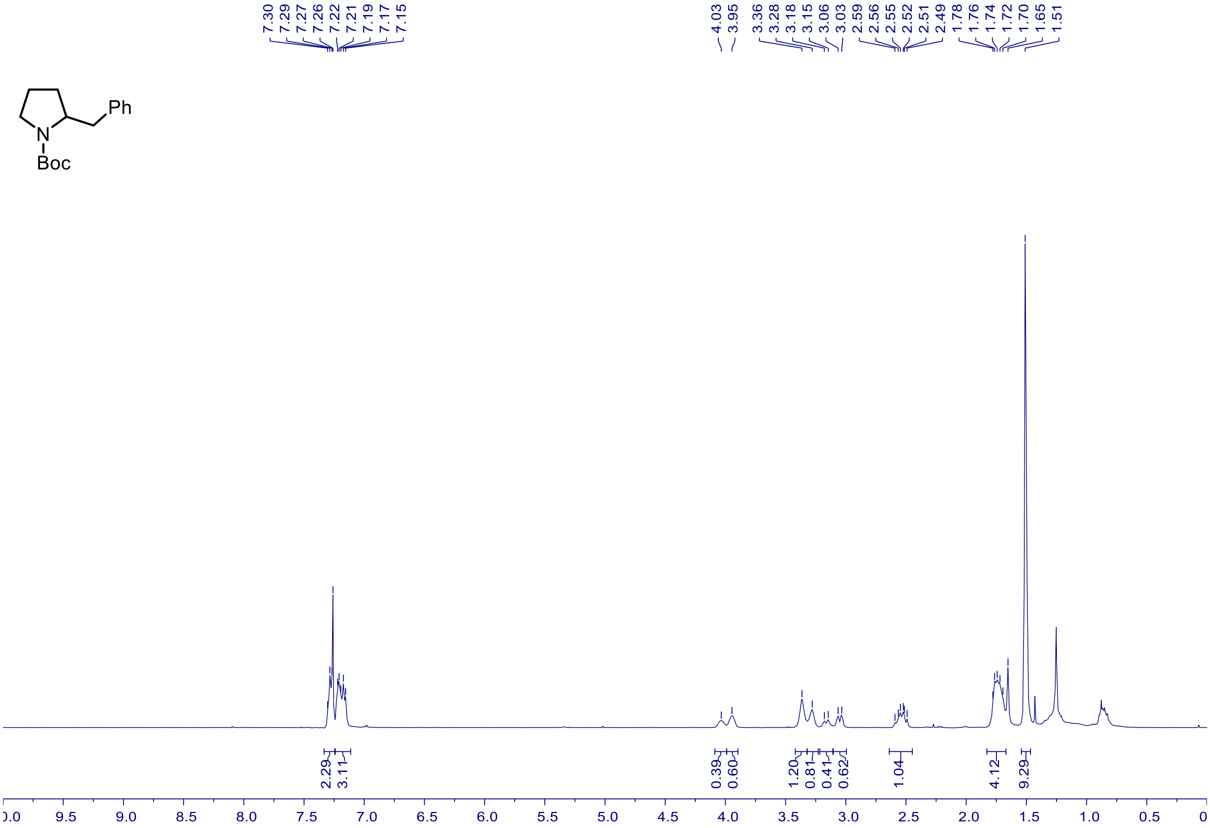

53 ^{13}C NMR (126 MHz, $CDCl_3$)

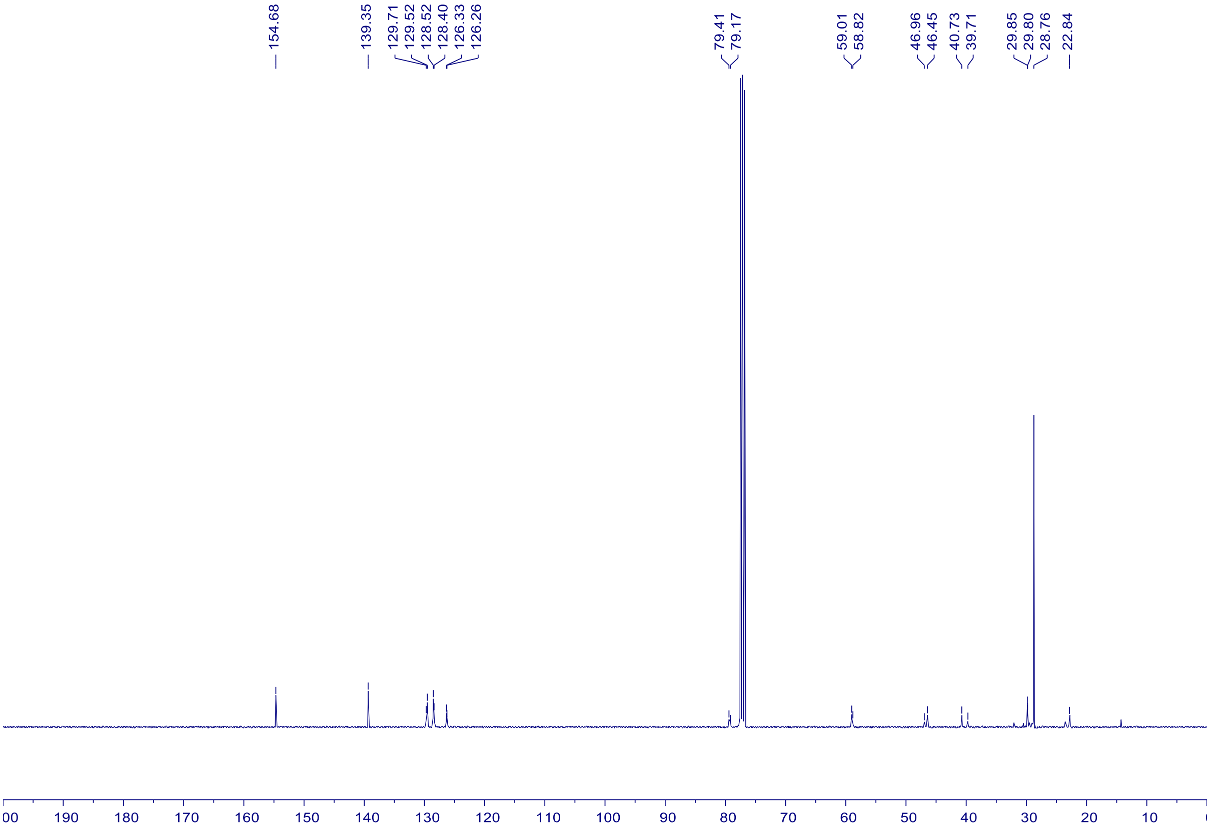

54 ^{1}H NMR (500 MHz, $CDCl_3$)

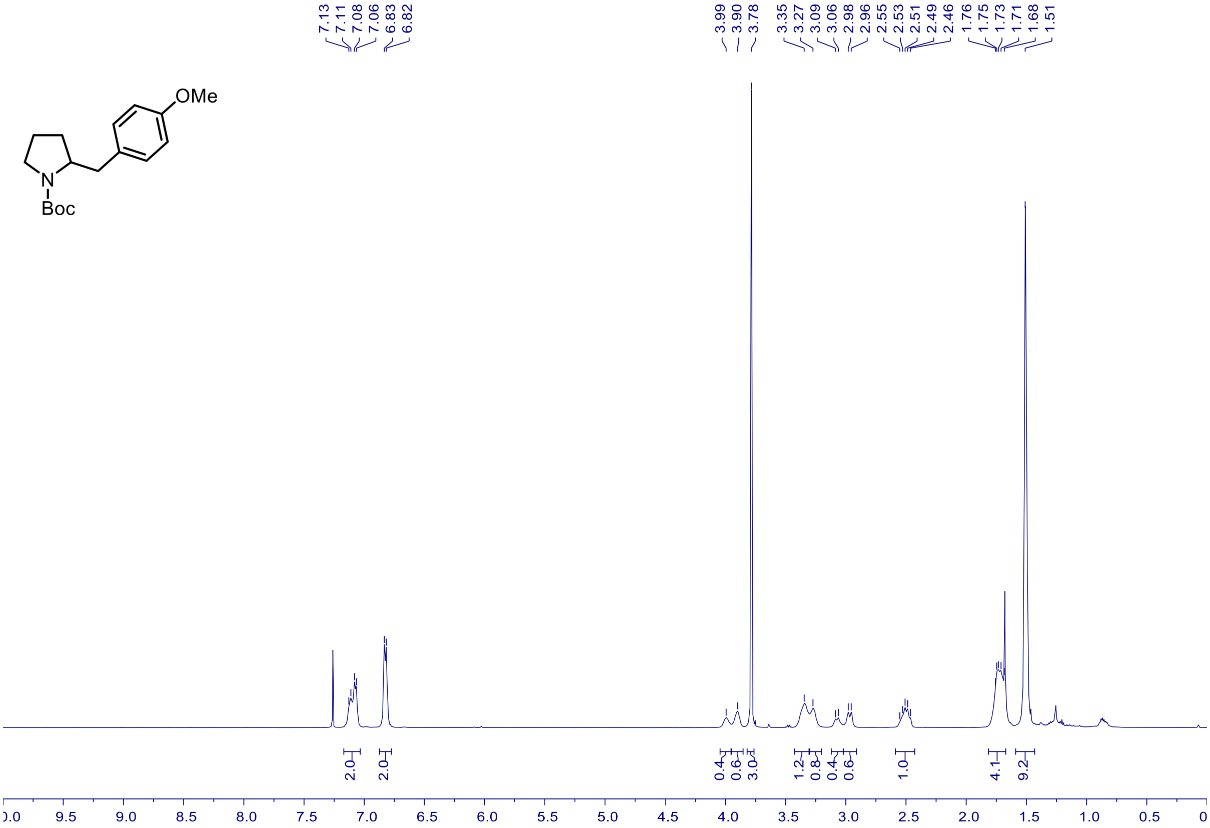

54 ^{13}C NMR (126 MHz, $CDCl_3$)

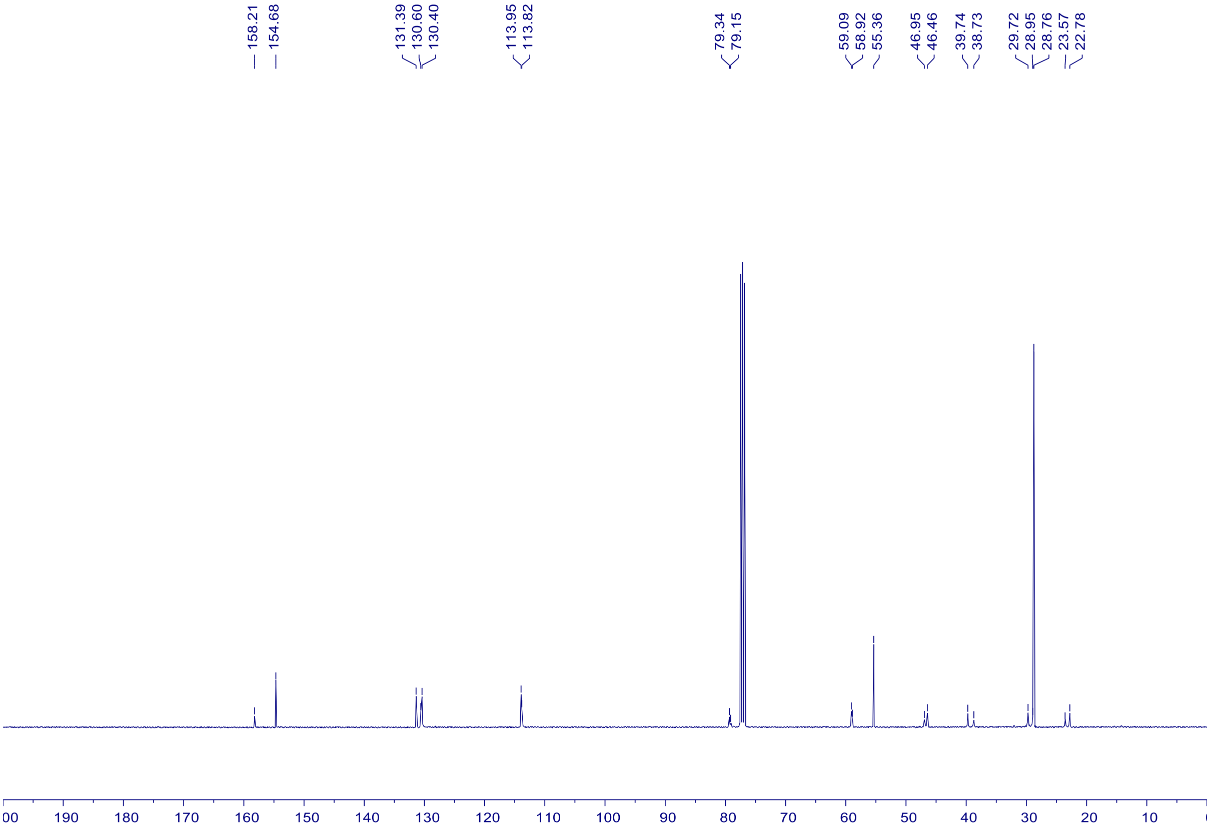

55 ^{1}H NMR (500 MHz, $CDCl_3$)

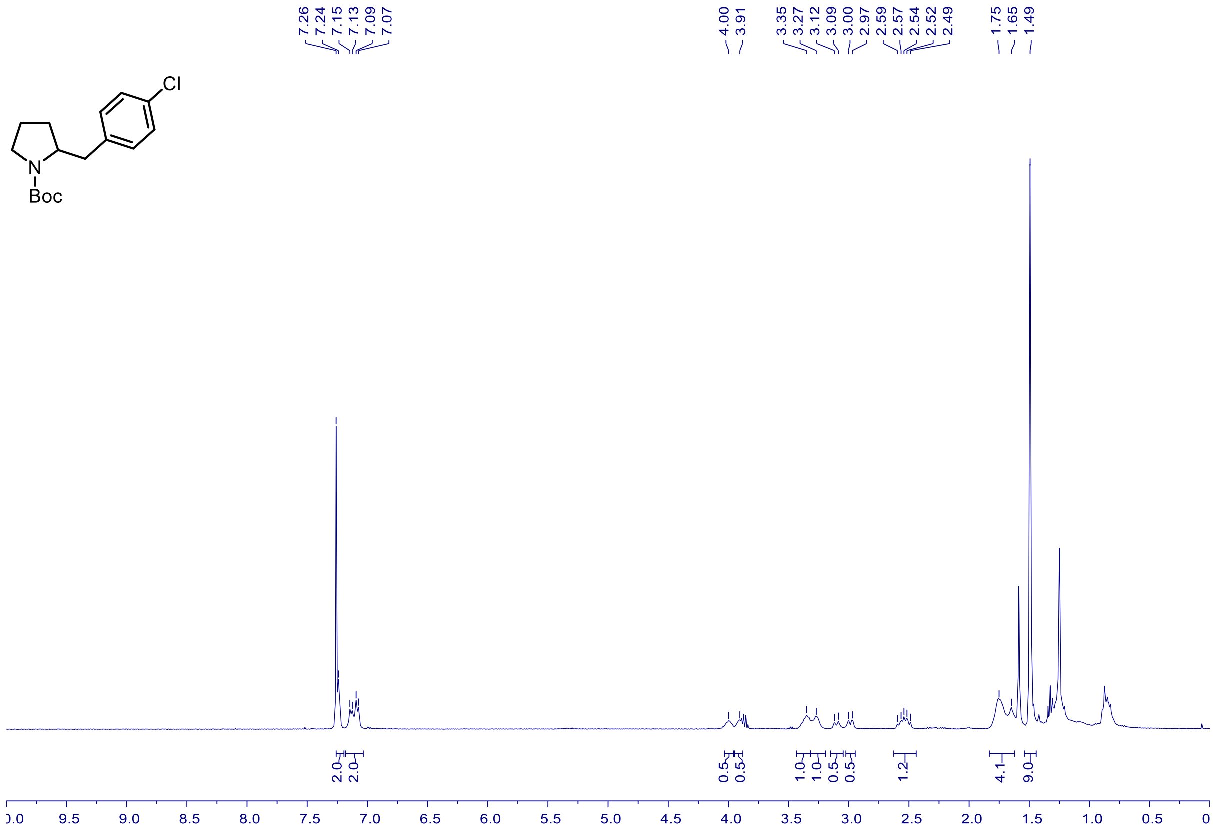

55 ^{13}C NMR (126 MHz, $CDCl_3$)

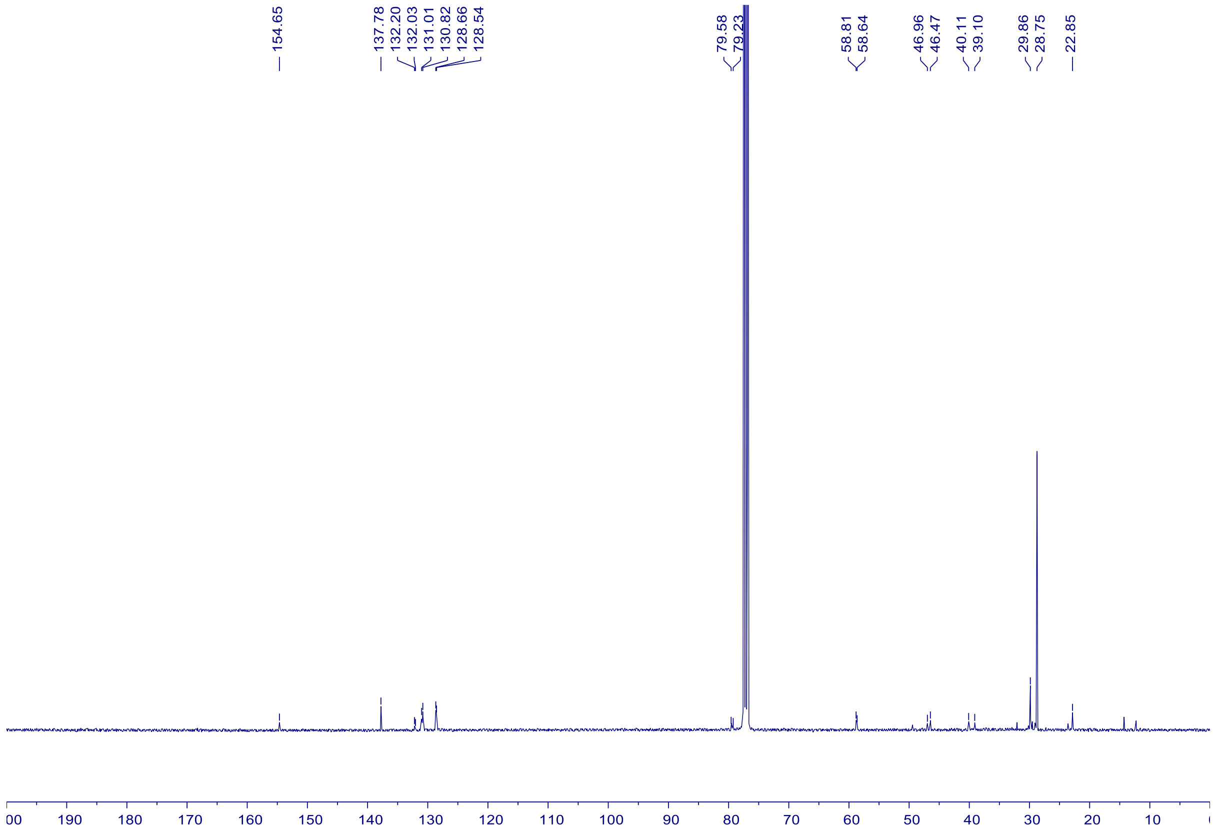

56 ^{1}H NMR (500 MHz, $CDCl_3$)

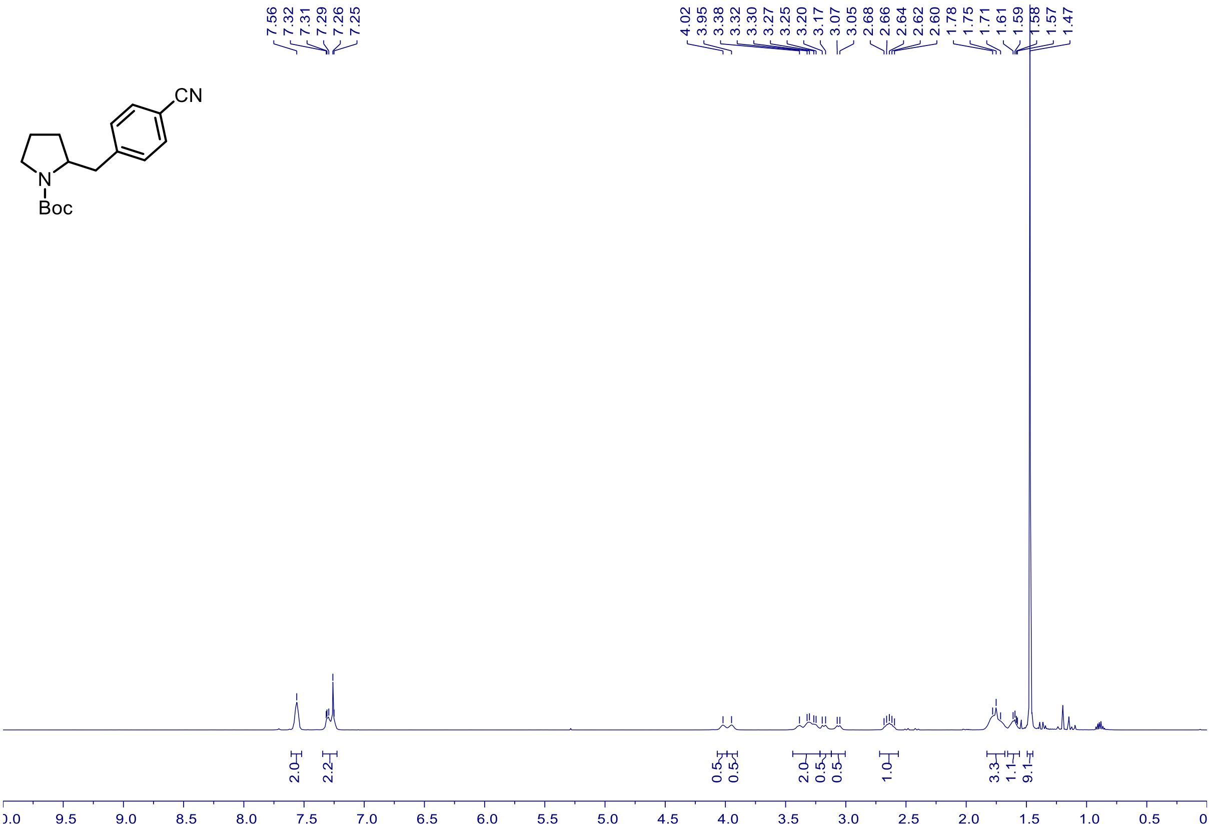

56 ^{13}C NMR (126 MHz, $CDCl_3$)

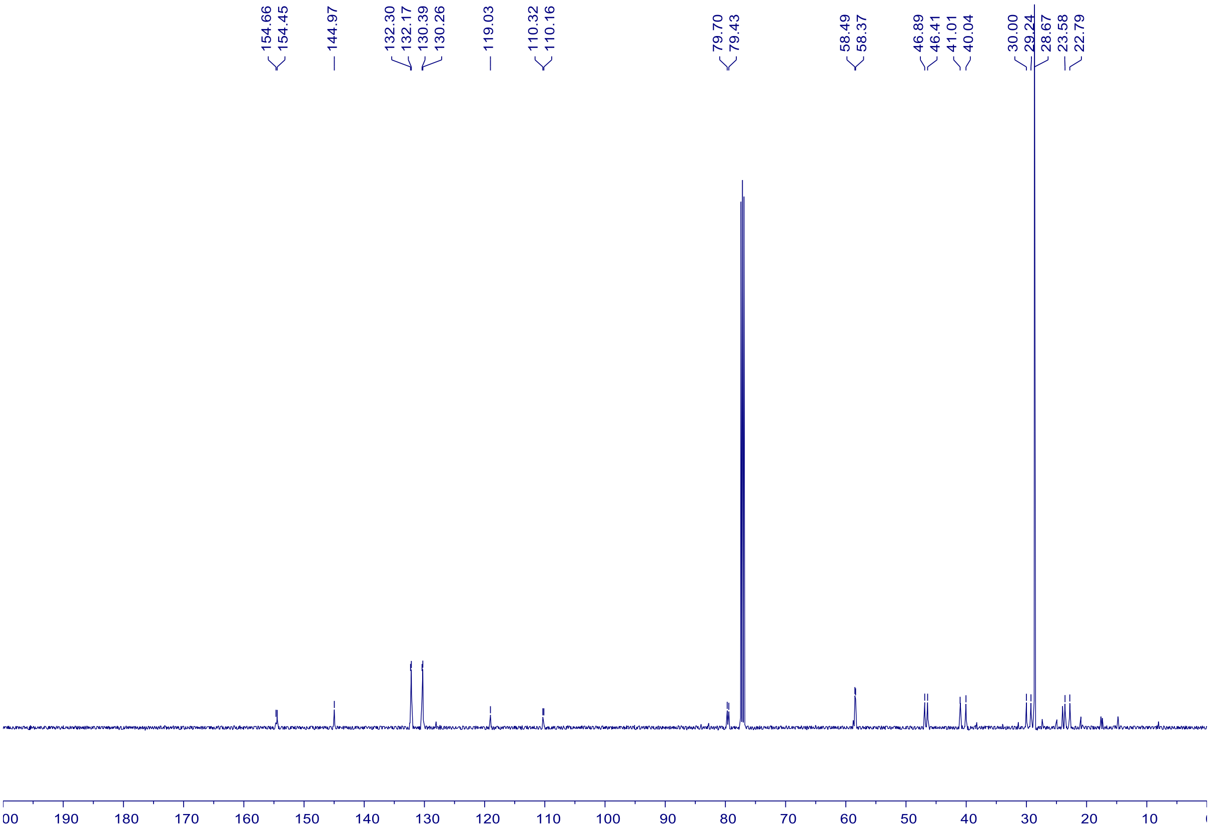

57 ^{1}H NMR (500 MHz, $CDCl_3$)

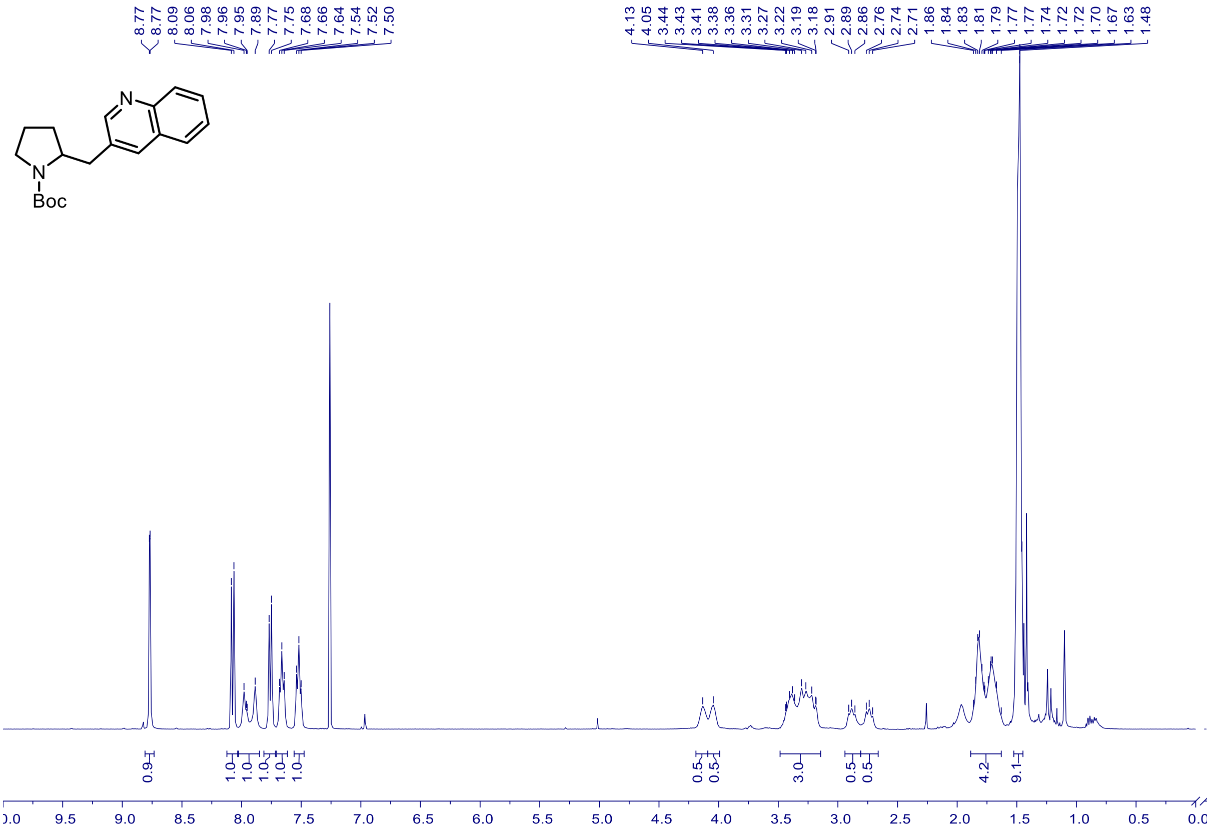

57 ^{13}C NMR (126 MHz, $CDCl_3$)

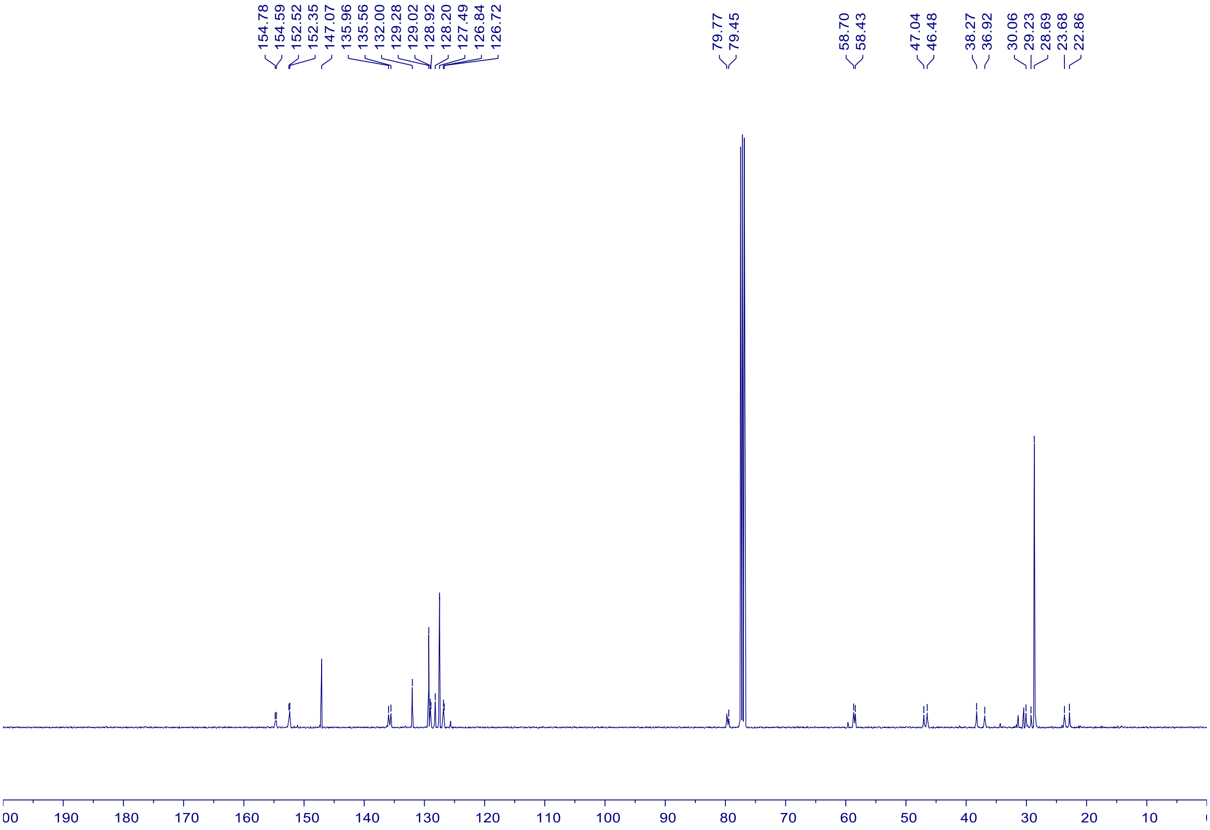

58 ^{1}H NMR (500 MHz, $CDCl_3$)

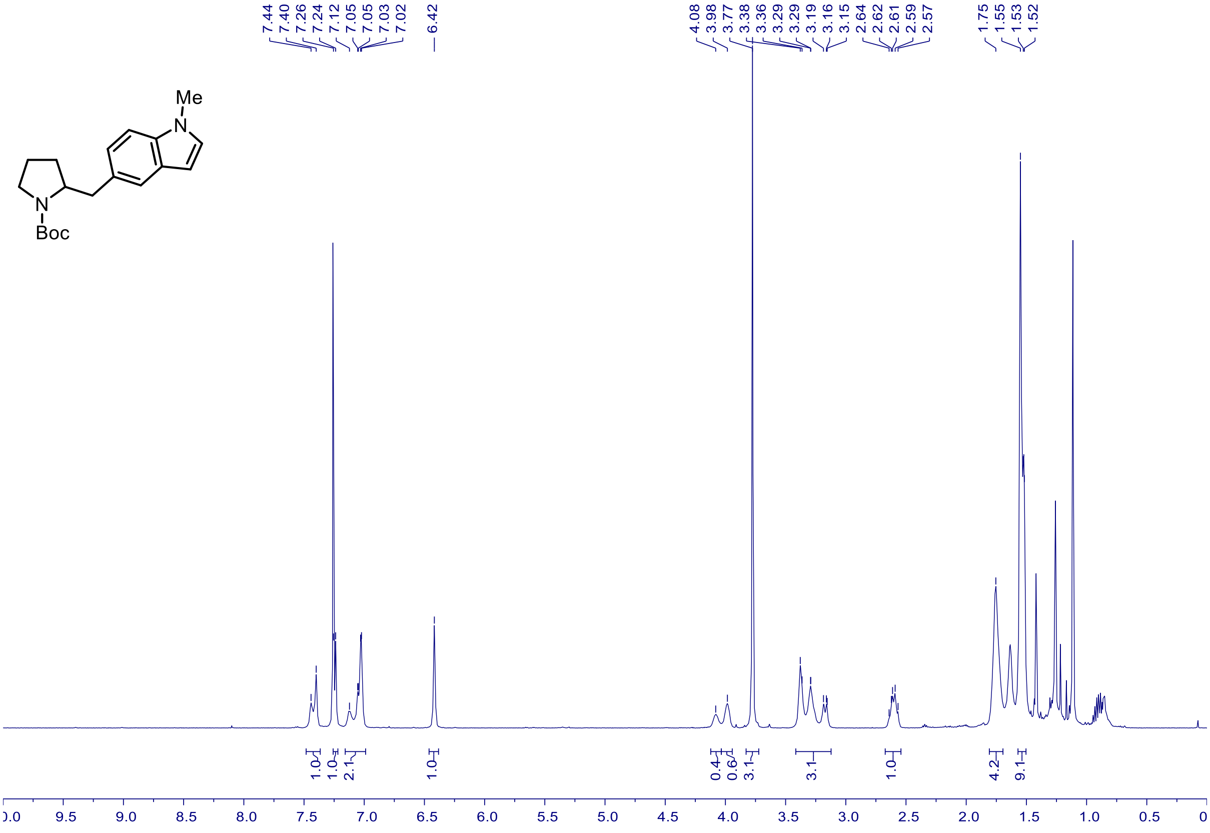

58 ^{13}C NMR (126 MHz, CDCl$_3$)

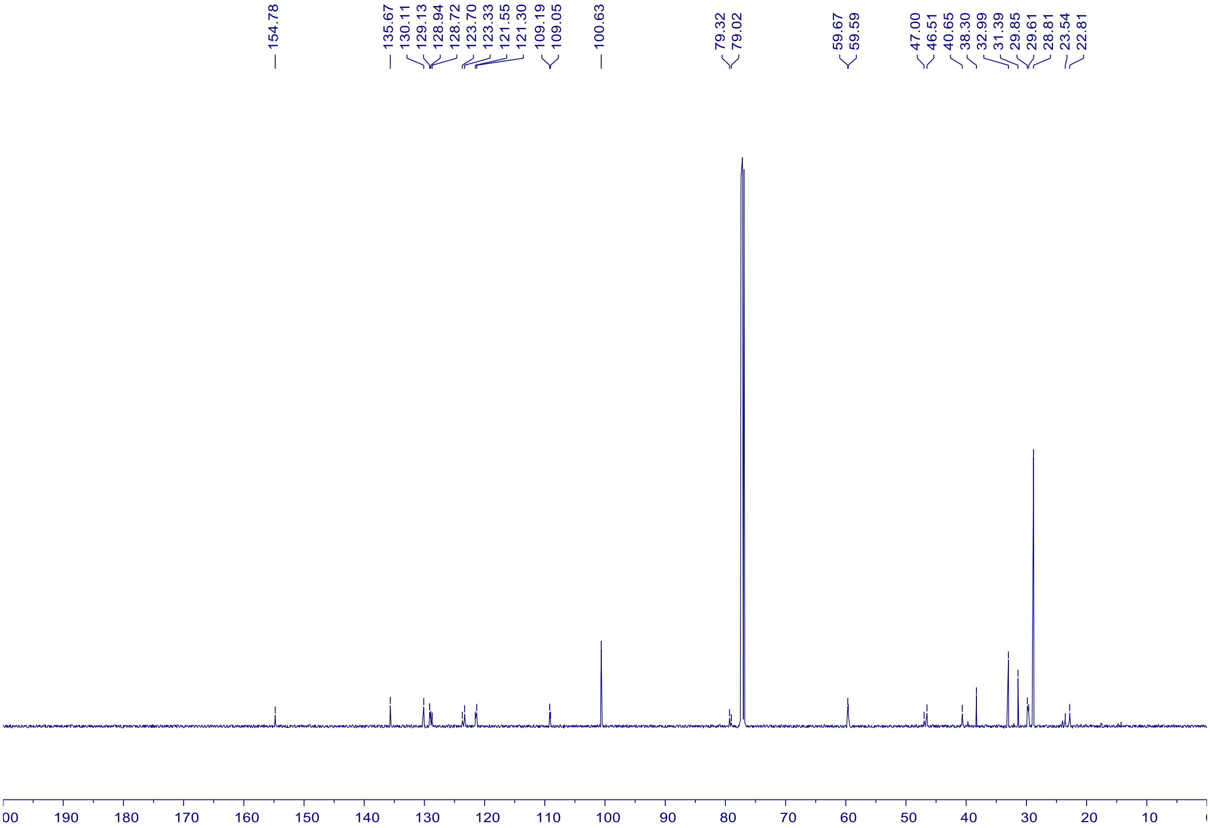

59 ^{1}H NMR (500 MHz, CDCl$_3$)

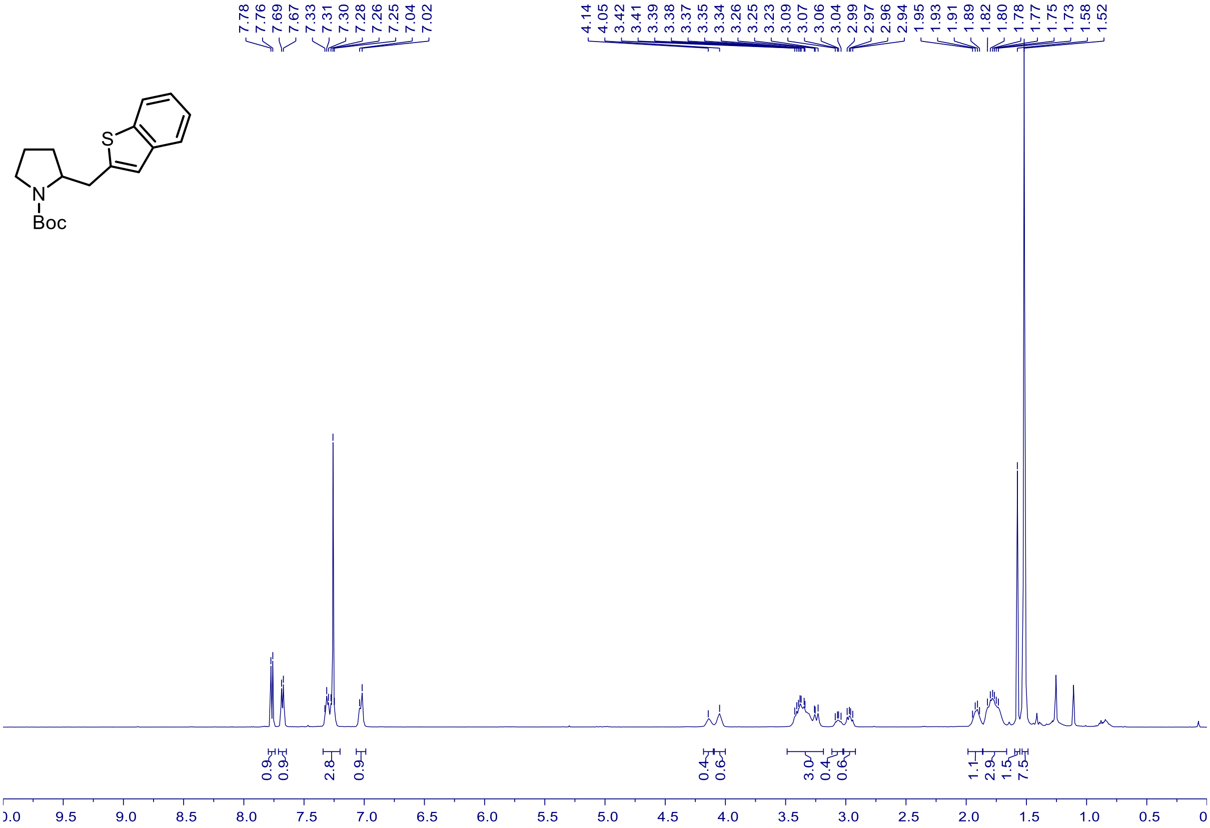

59 ^{13}C NMR (126 MHz, $CDCl_3$)

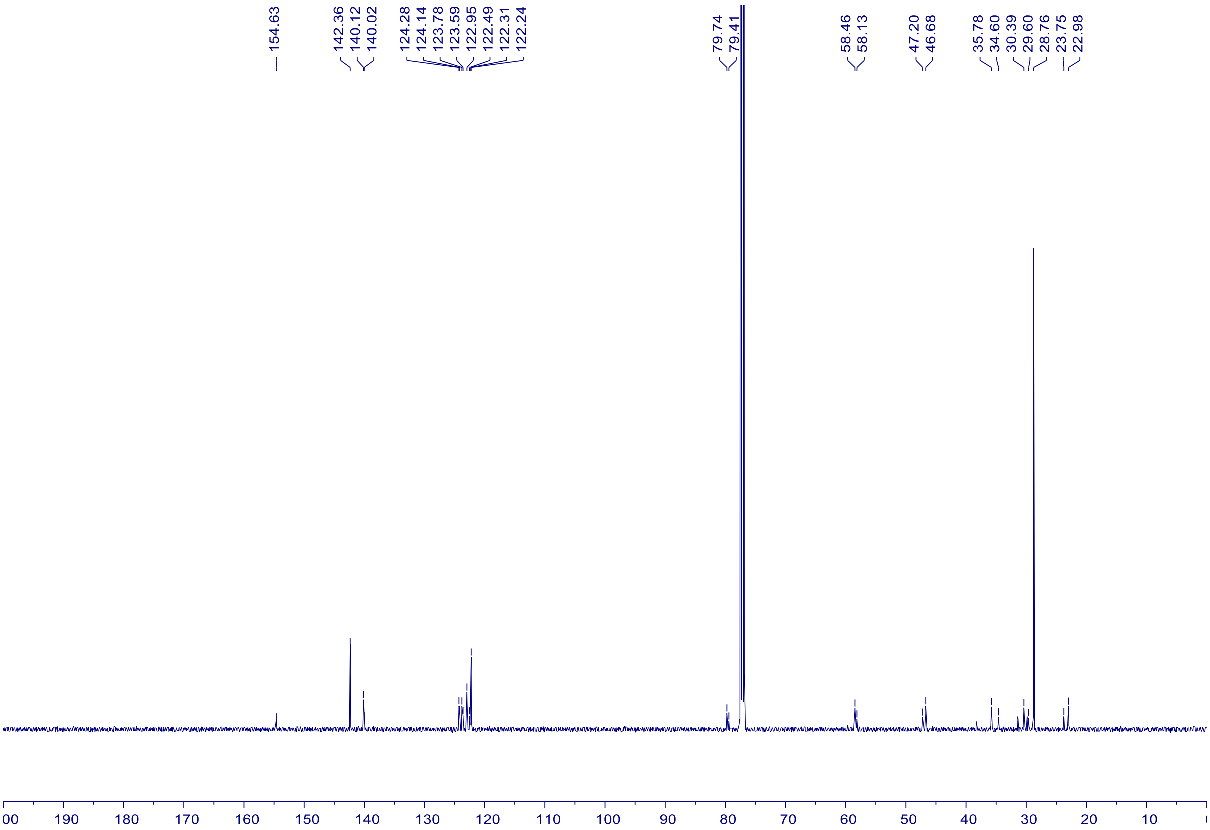

61 ^{1}H NMR (500 MHz, $CDCl_3$)

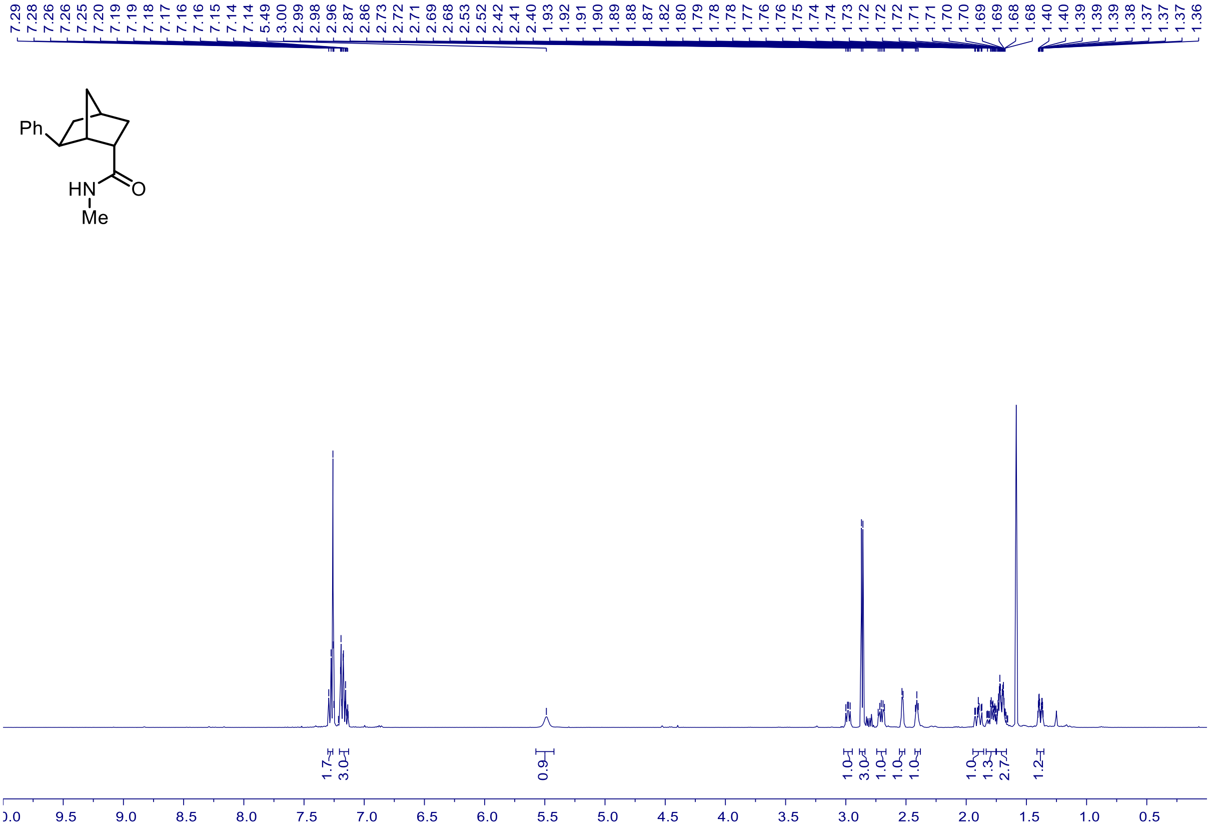

61 ^{13}C NMR (126 MHz, $CDCl_3$)

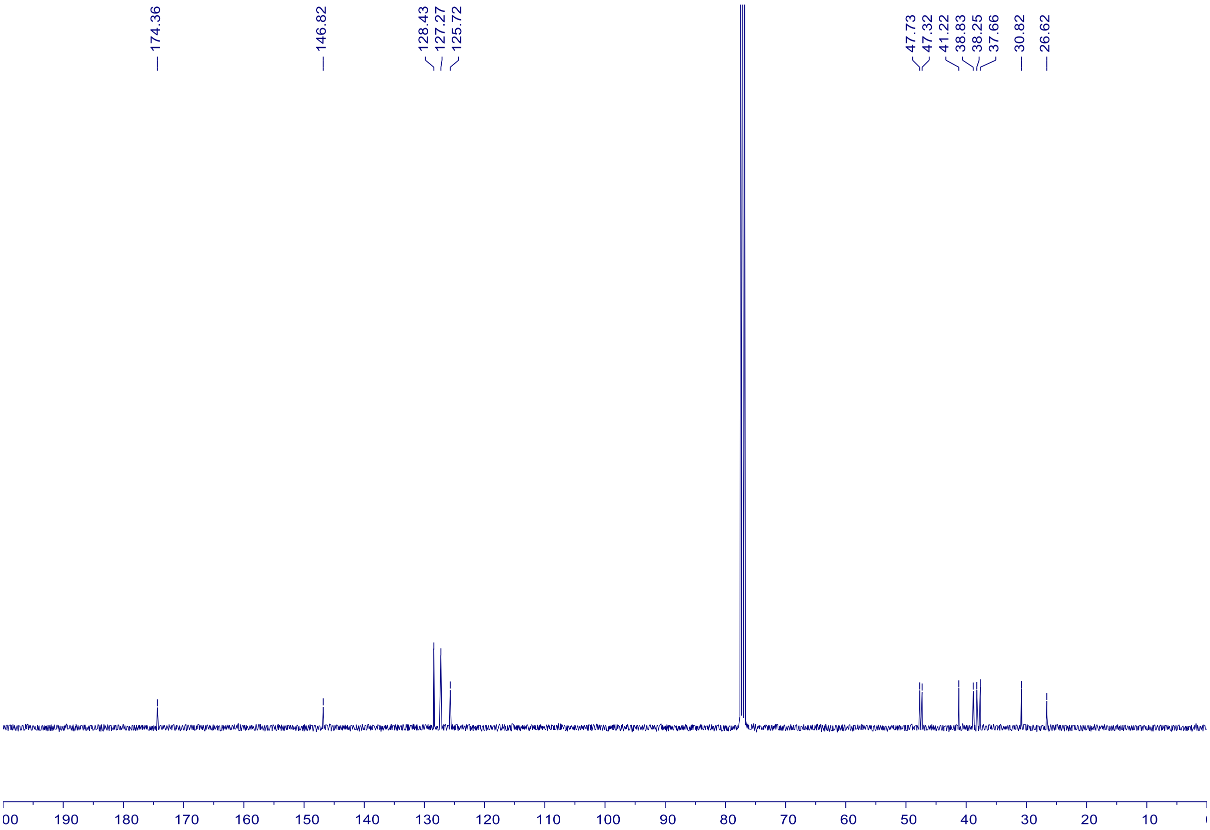

62 ^{1}H NMR (500 MHz, $CDCl_3$)

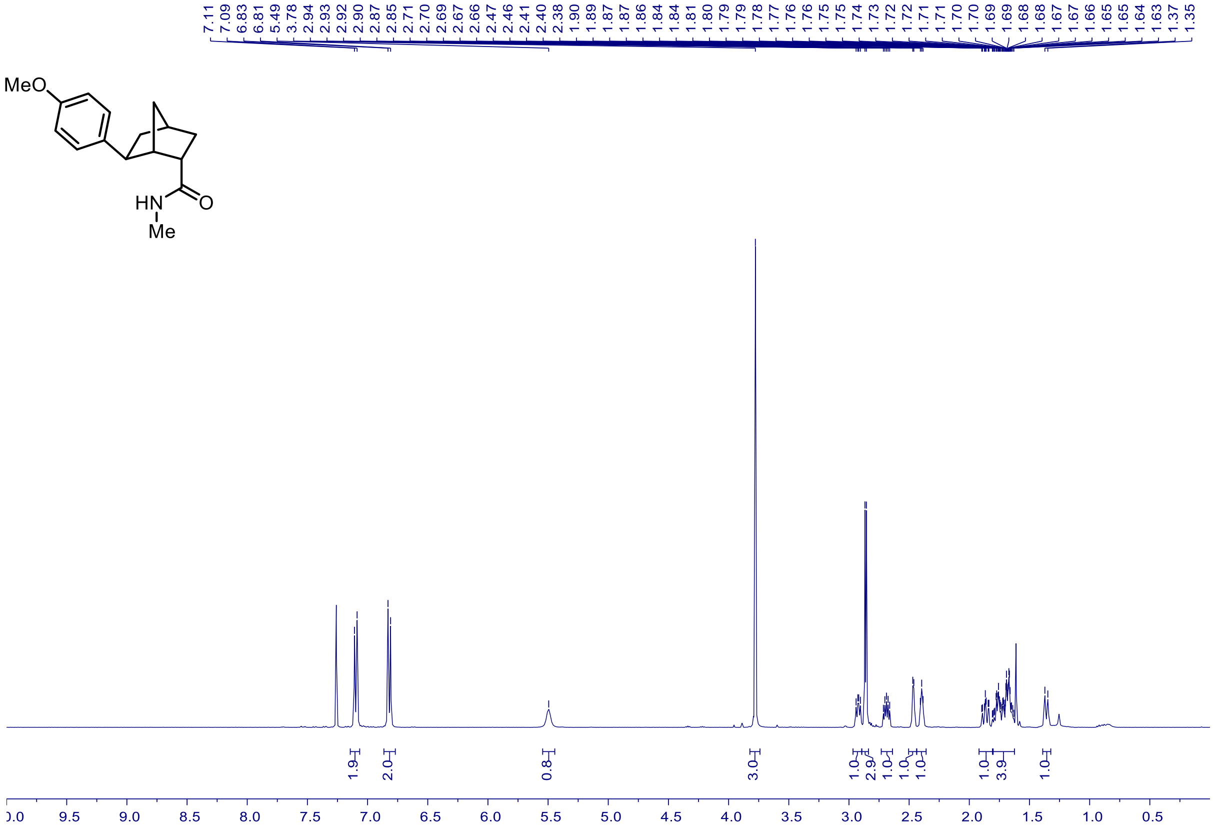

62 ^{13}C NMR (126 MHz, $CDCl_3$)

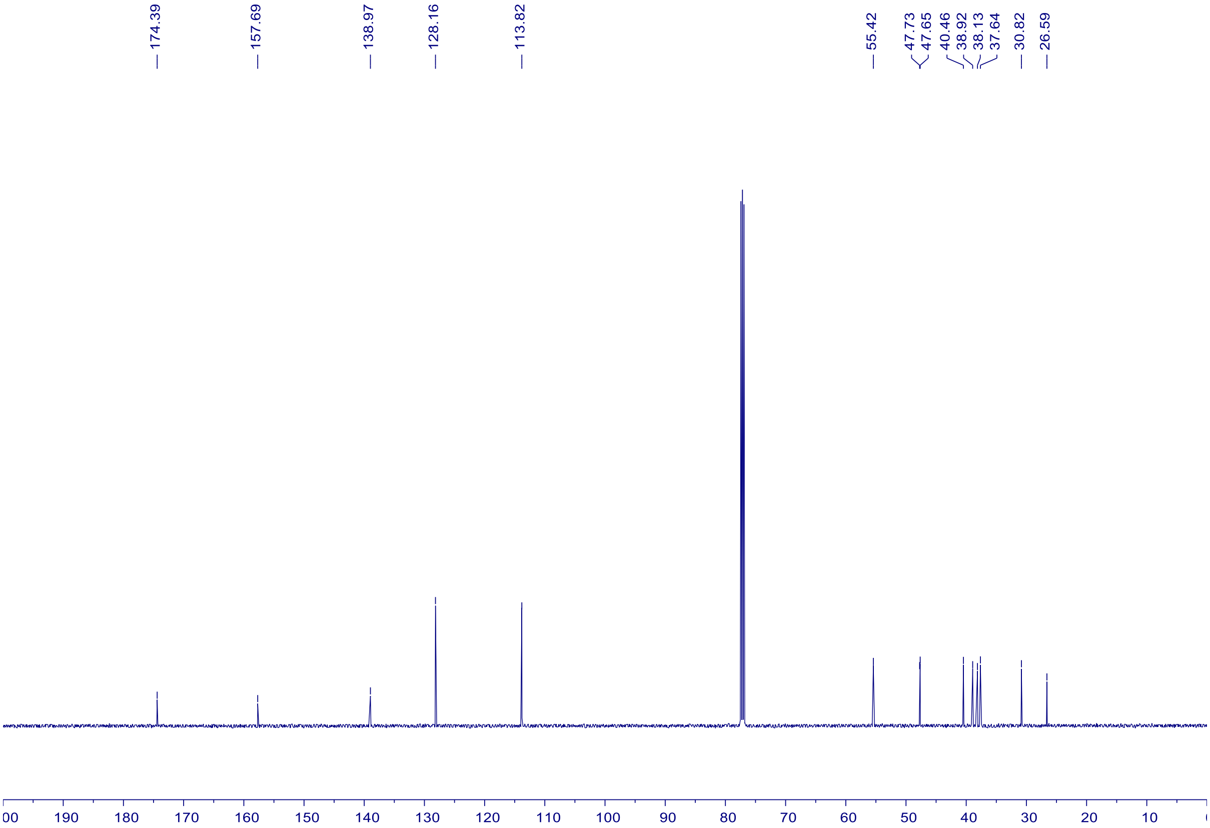

63 ^{1}H NMR (500 MHz, $CDCl_3$)

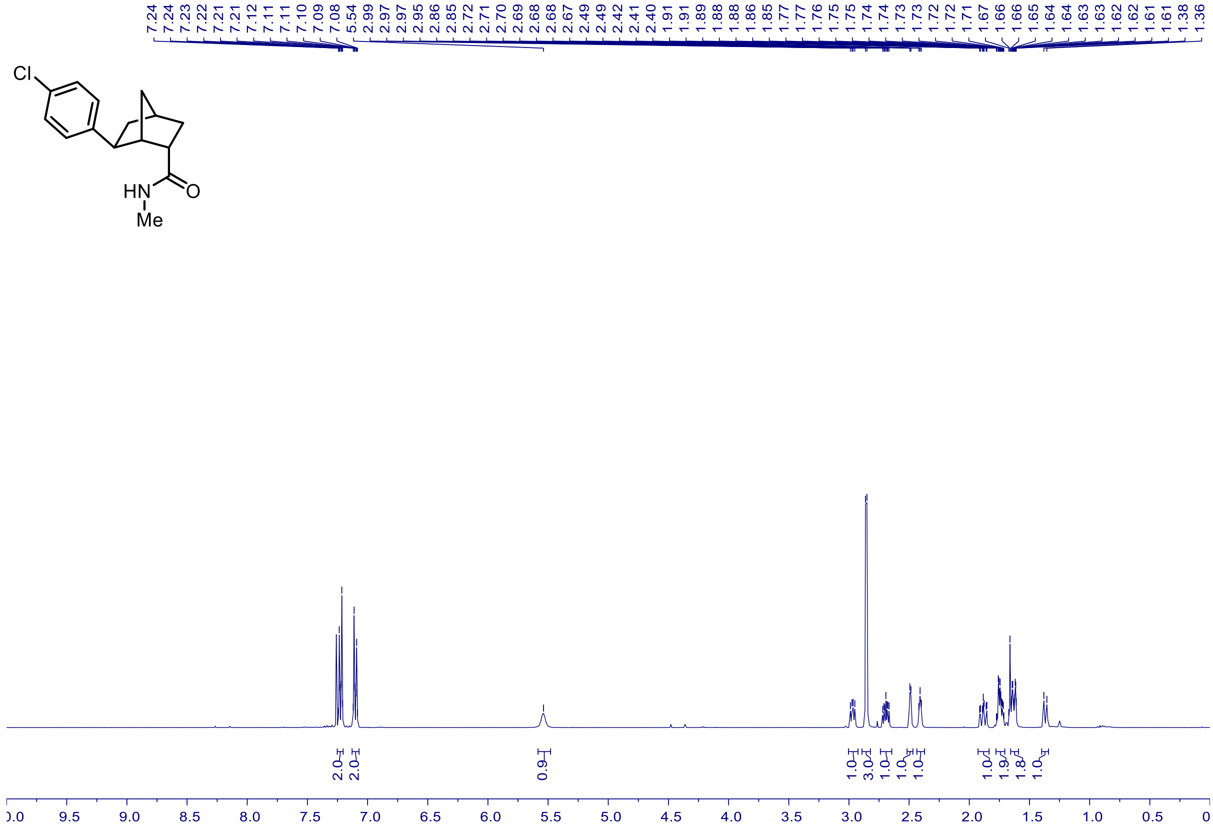

63 ^{13}C NMR (126 MHz, $CDCl_3$)

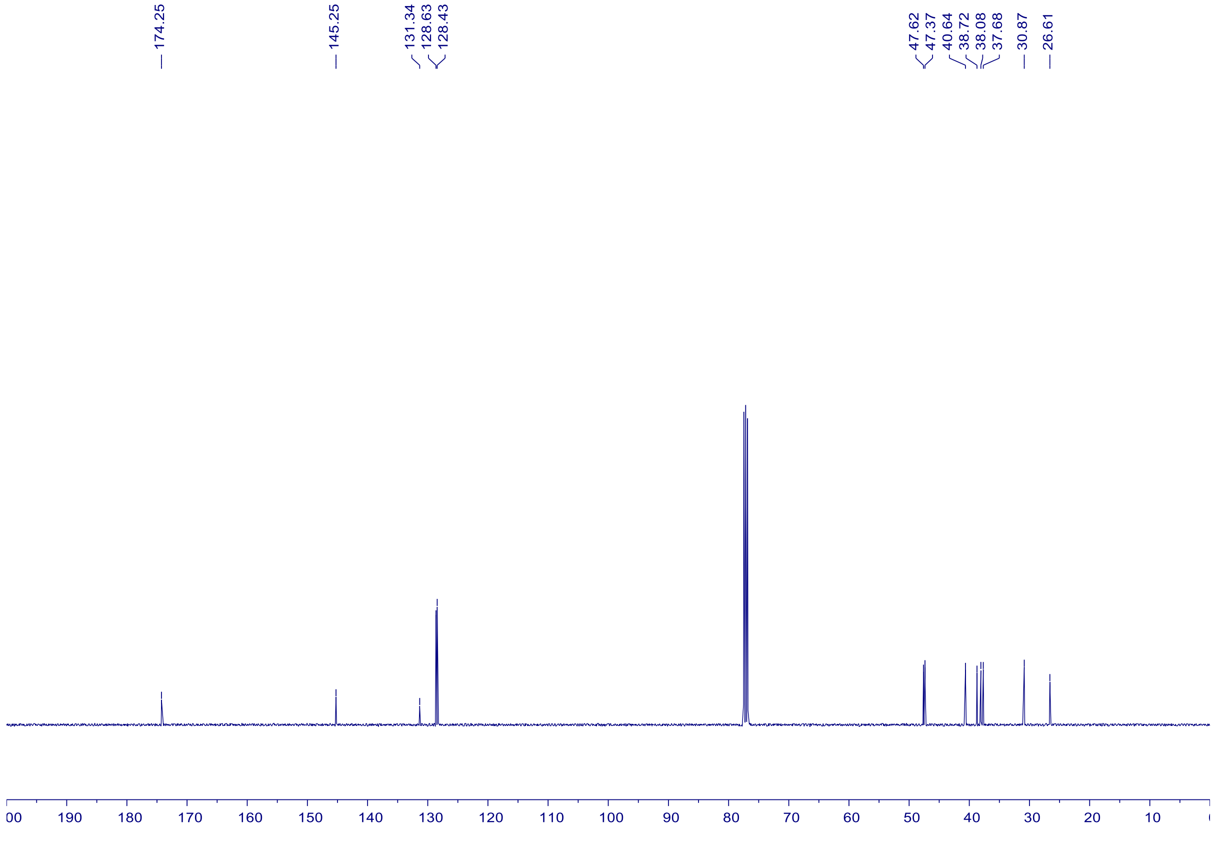

64 ^{1}H NMR (500 MHz, $CDCl_3$)

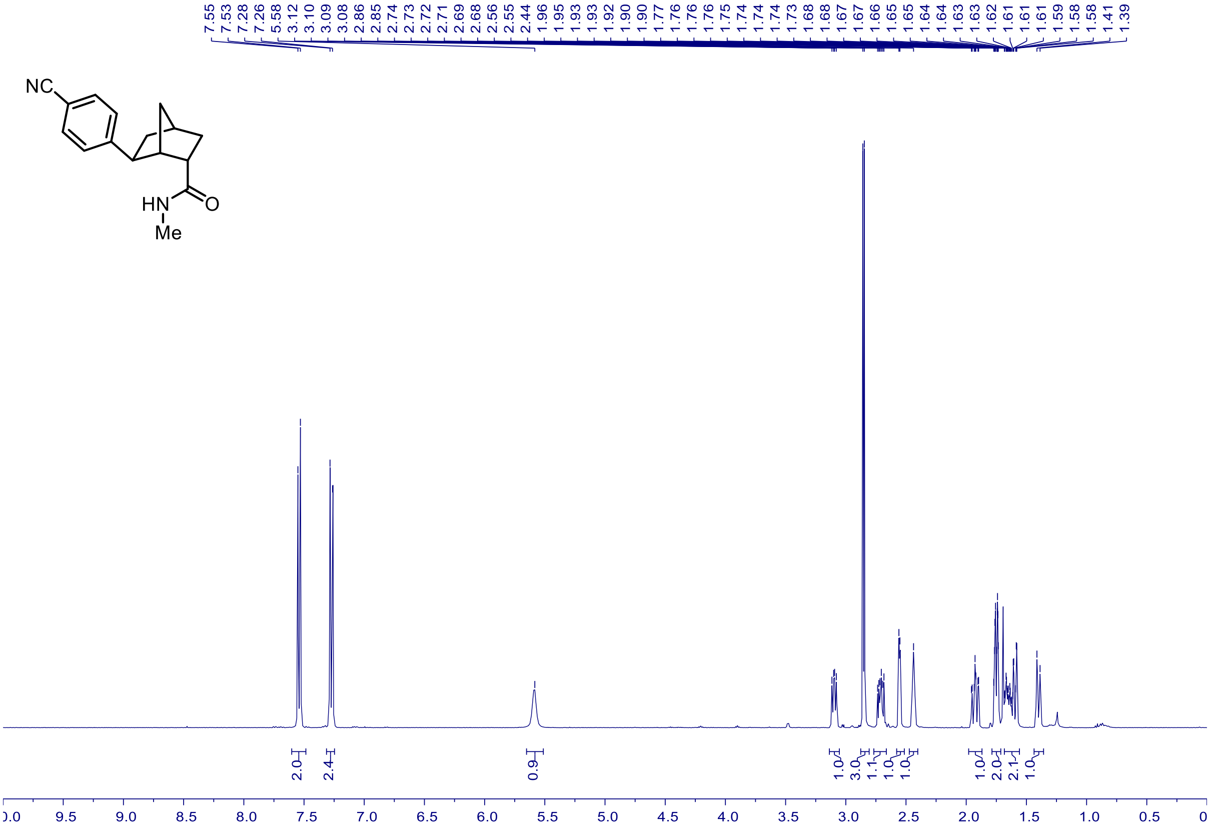

64 ^{13}C NMR (126 MHz, $CDCl_3$)

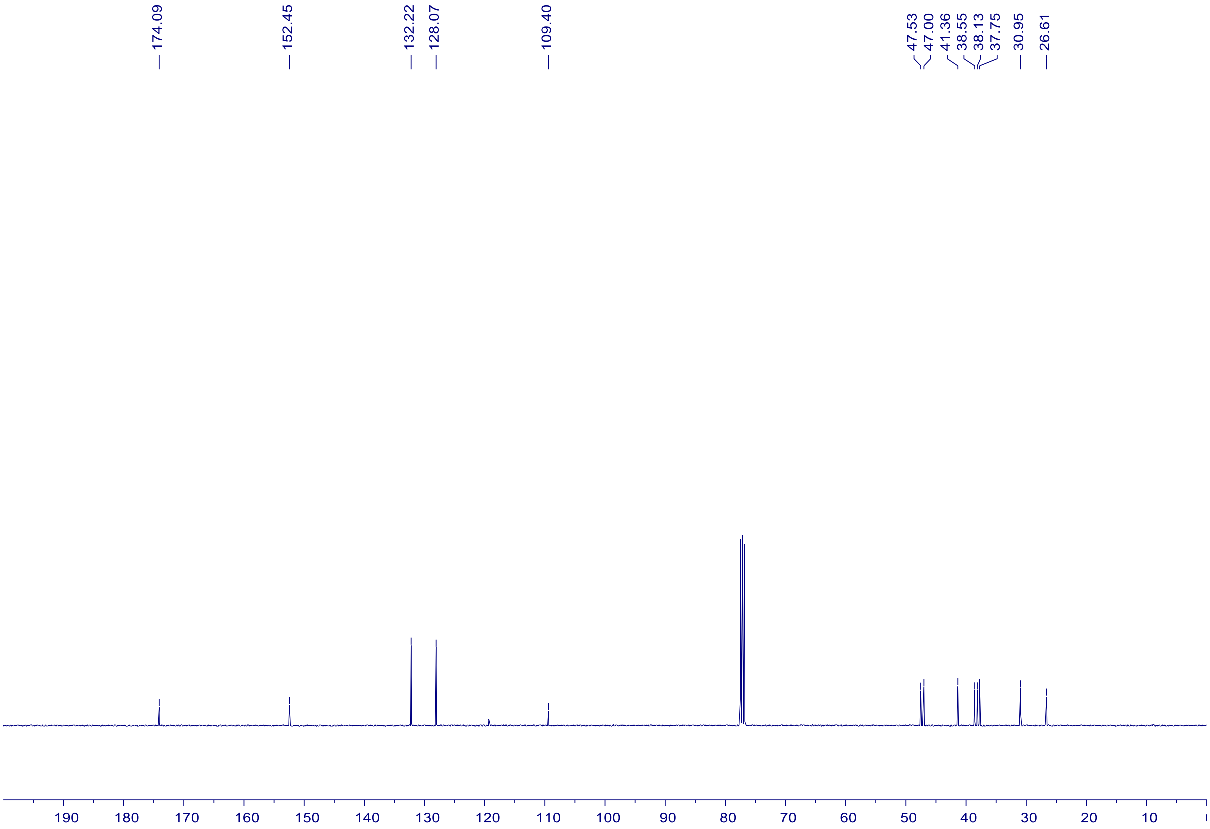

67 ^{1}H NMR (500 MHz, $CDCl_3$)

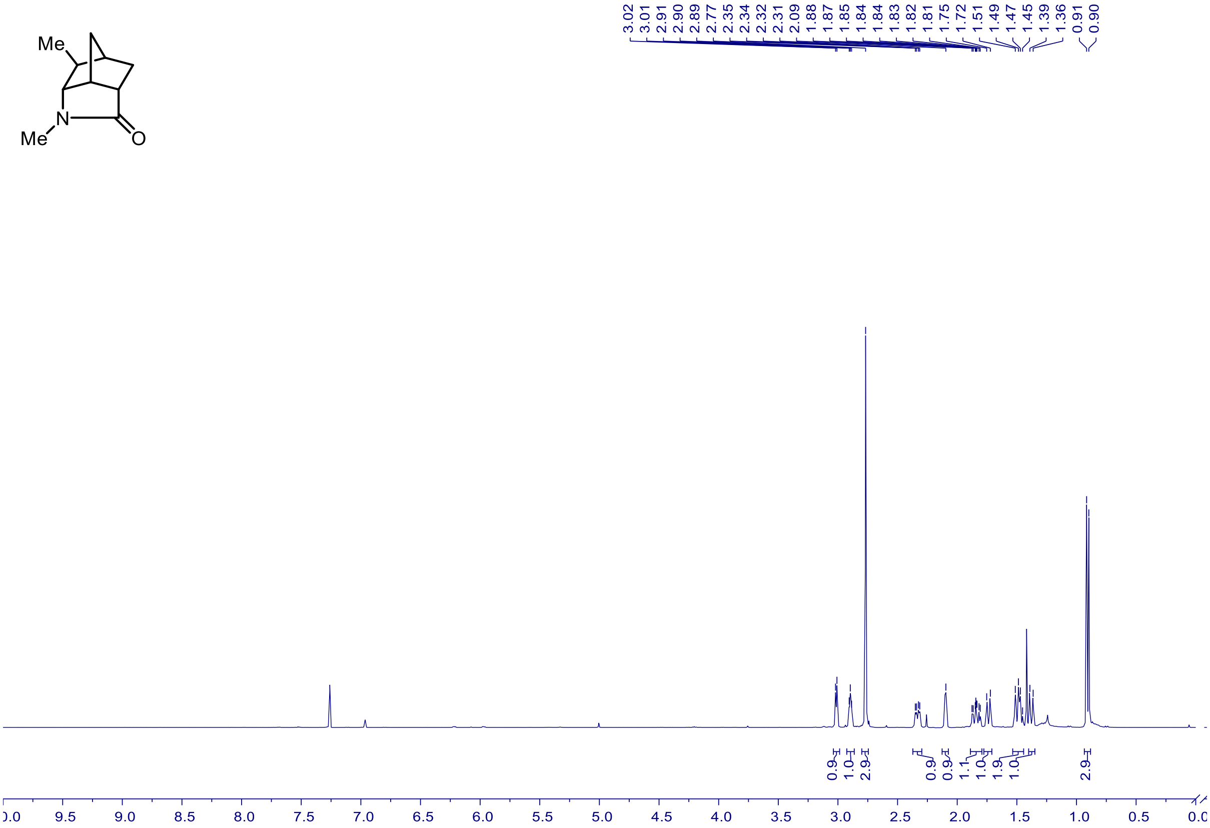

67 ^{13}C NMR (126 MHz, $CDCl_3$)

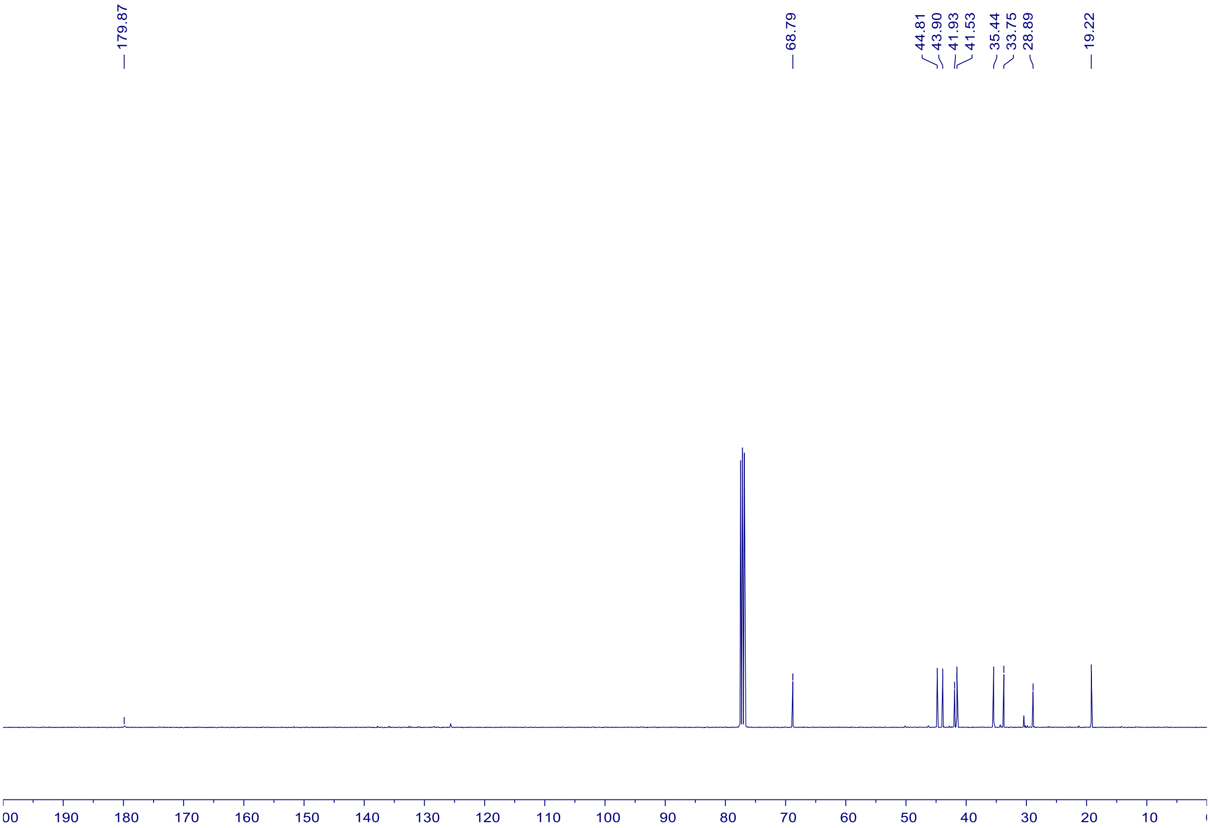

68 ^{1}H NMR (500 MHz, $CDCl_3$)

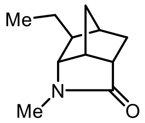

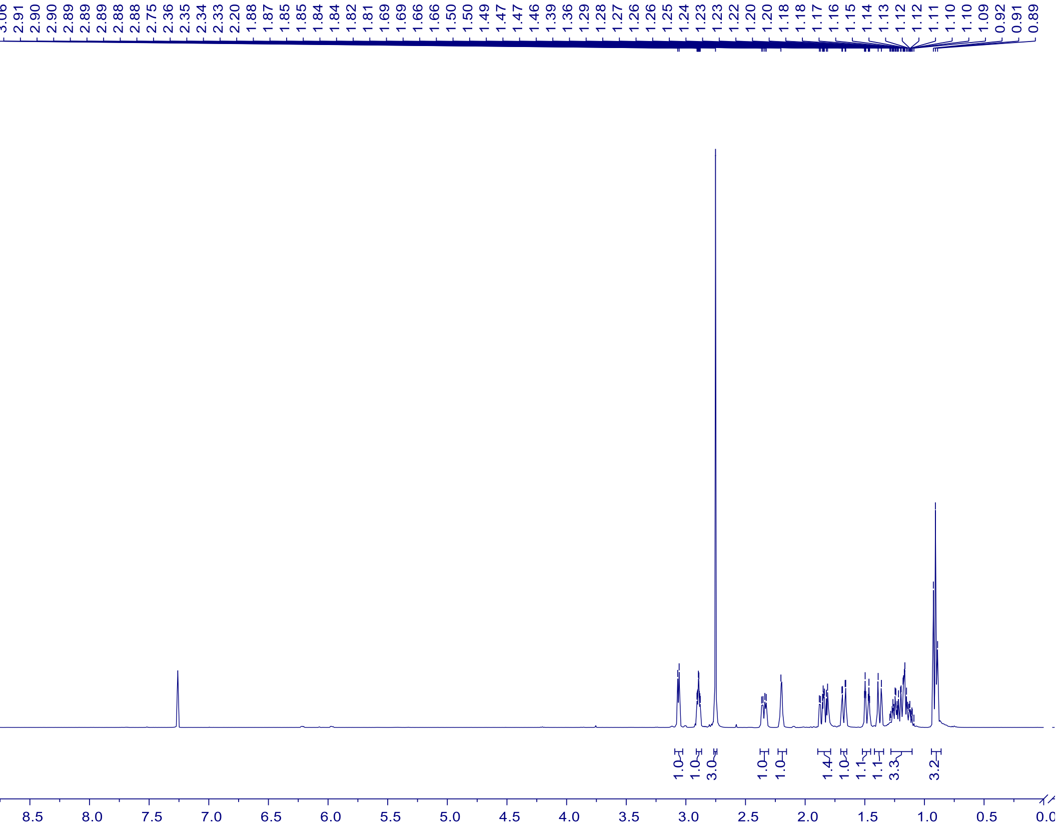

68 ^{13}C NMR (126 MHz, $CDCl_3$)

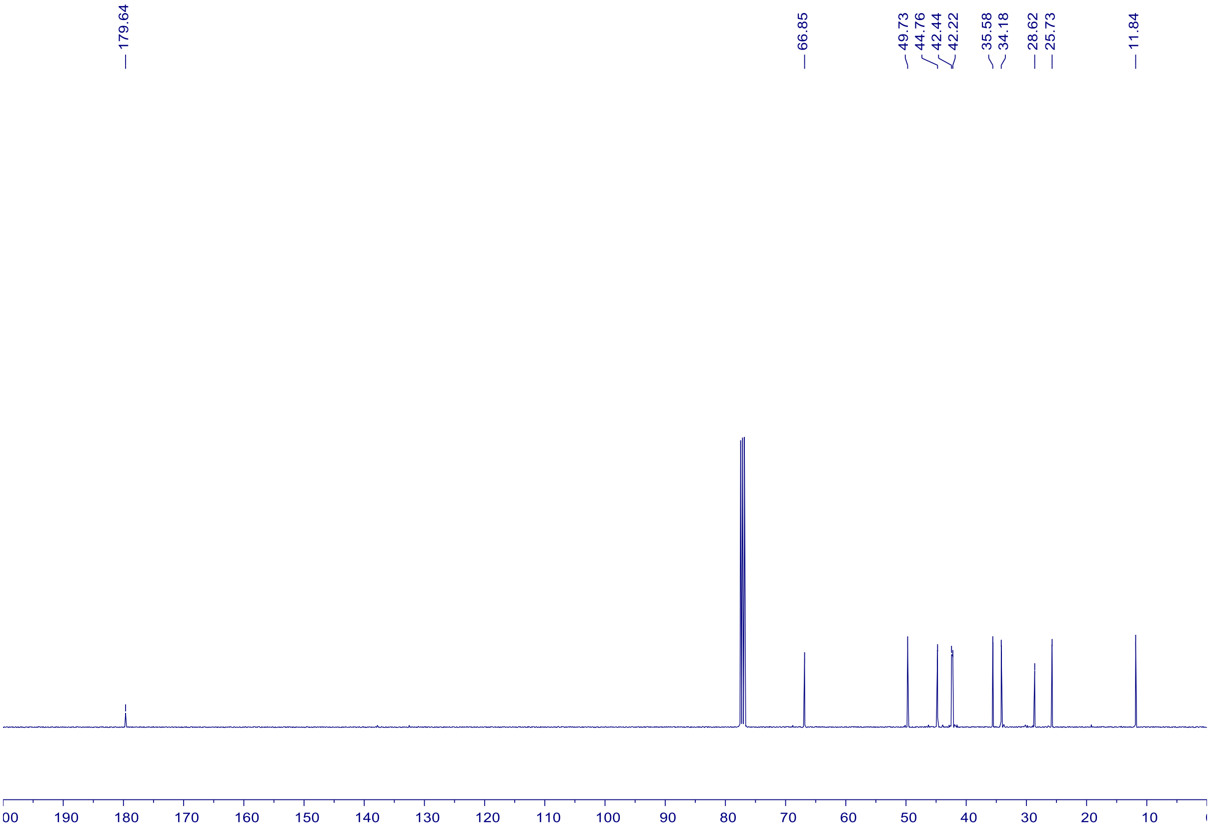

69 ^{1}H NMR (500 MHz, $CDCl_3$)

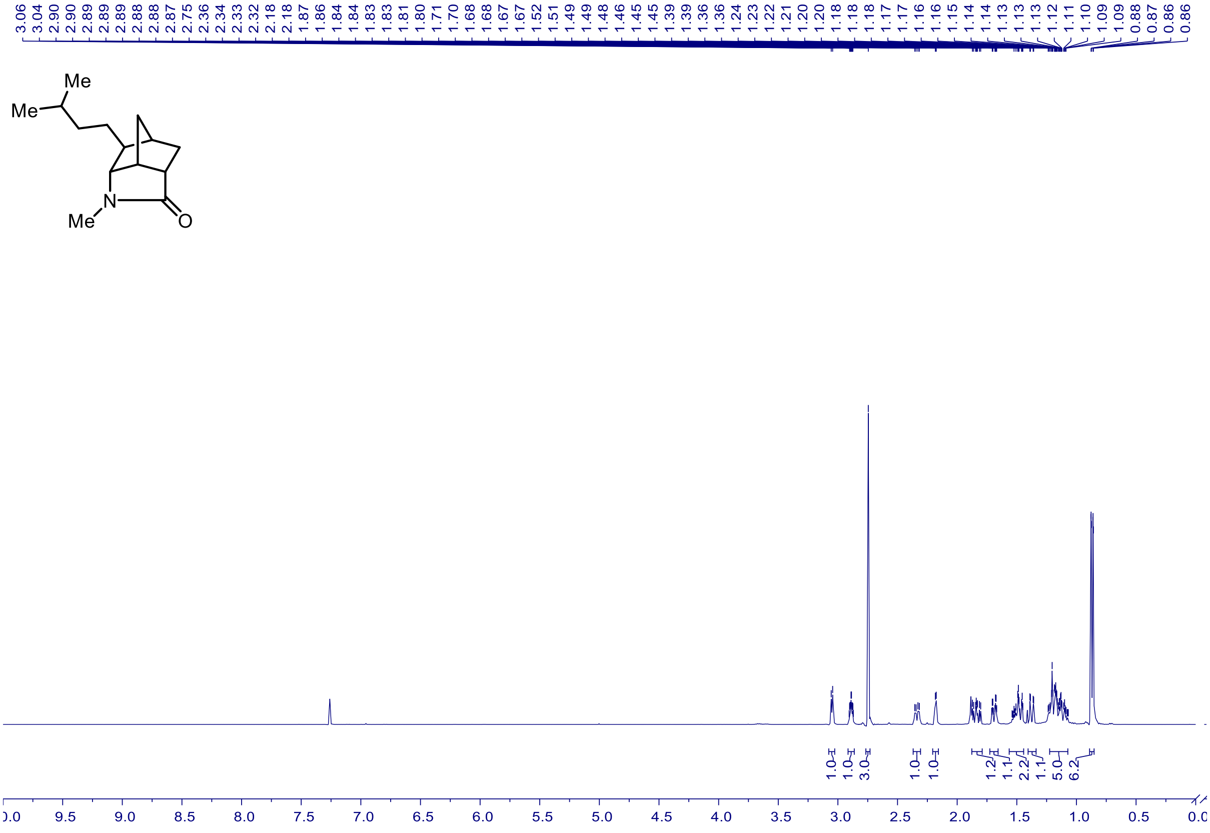

69 ^{13}C NMR (126 MHz, CDCl$_3$)

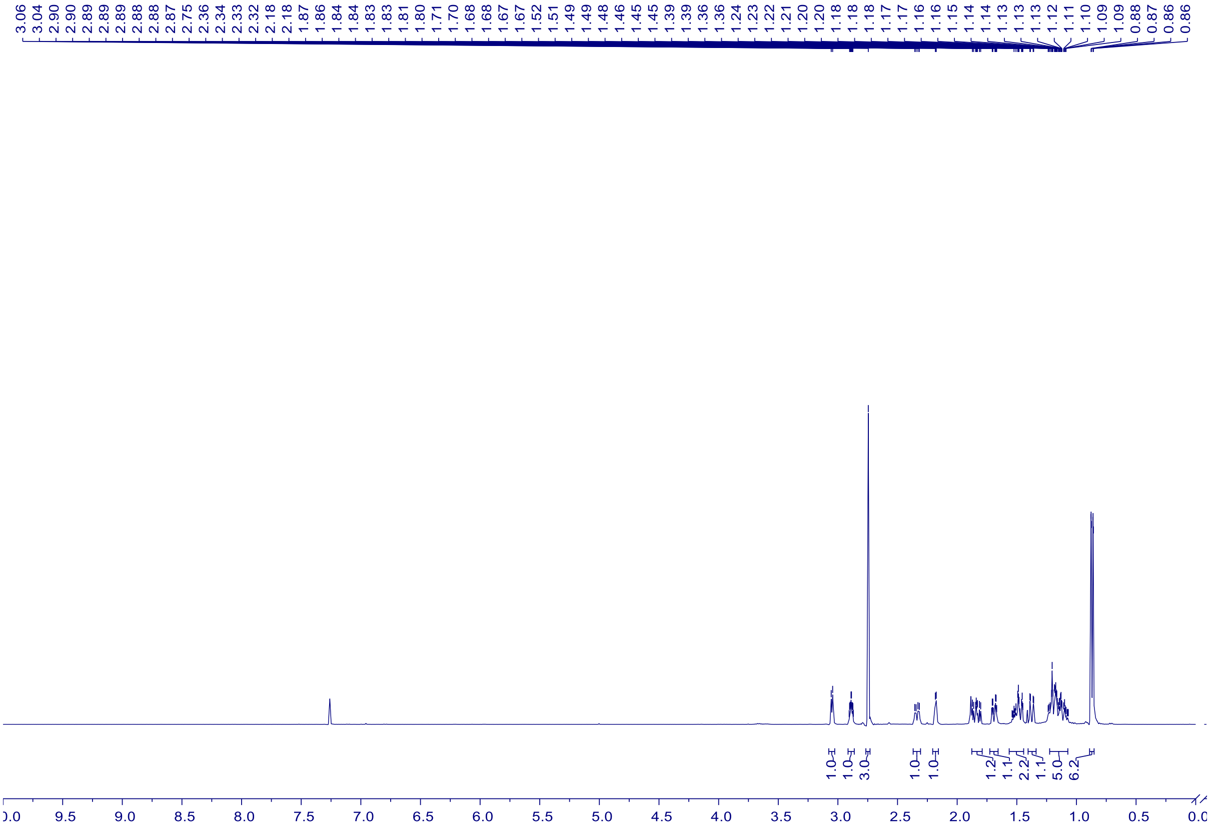

70 ^{1}H NMR (500 MHz, CDCl$_3$)

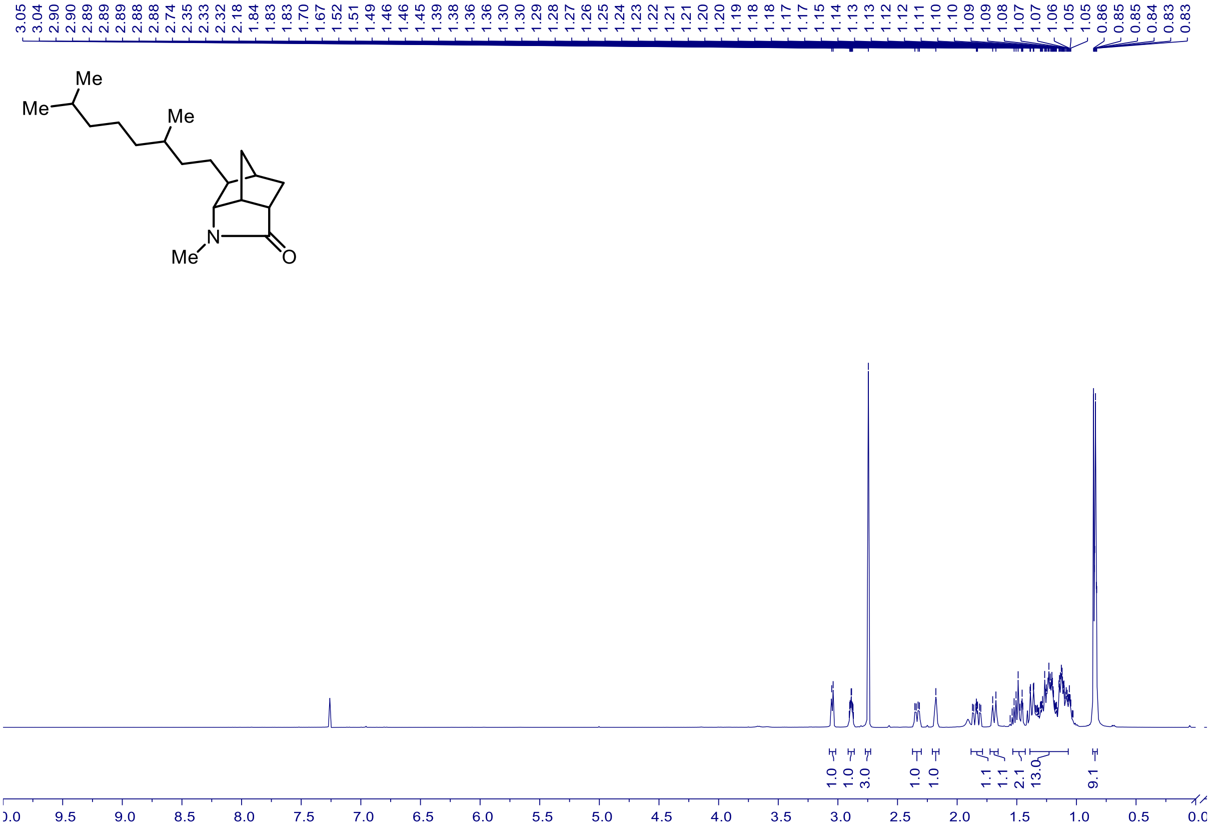

70 ^{13}C NMR (126 MHz, $CDCl_3$)

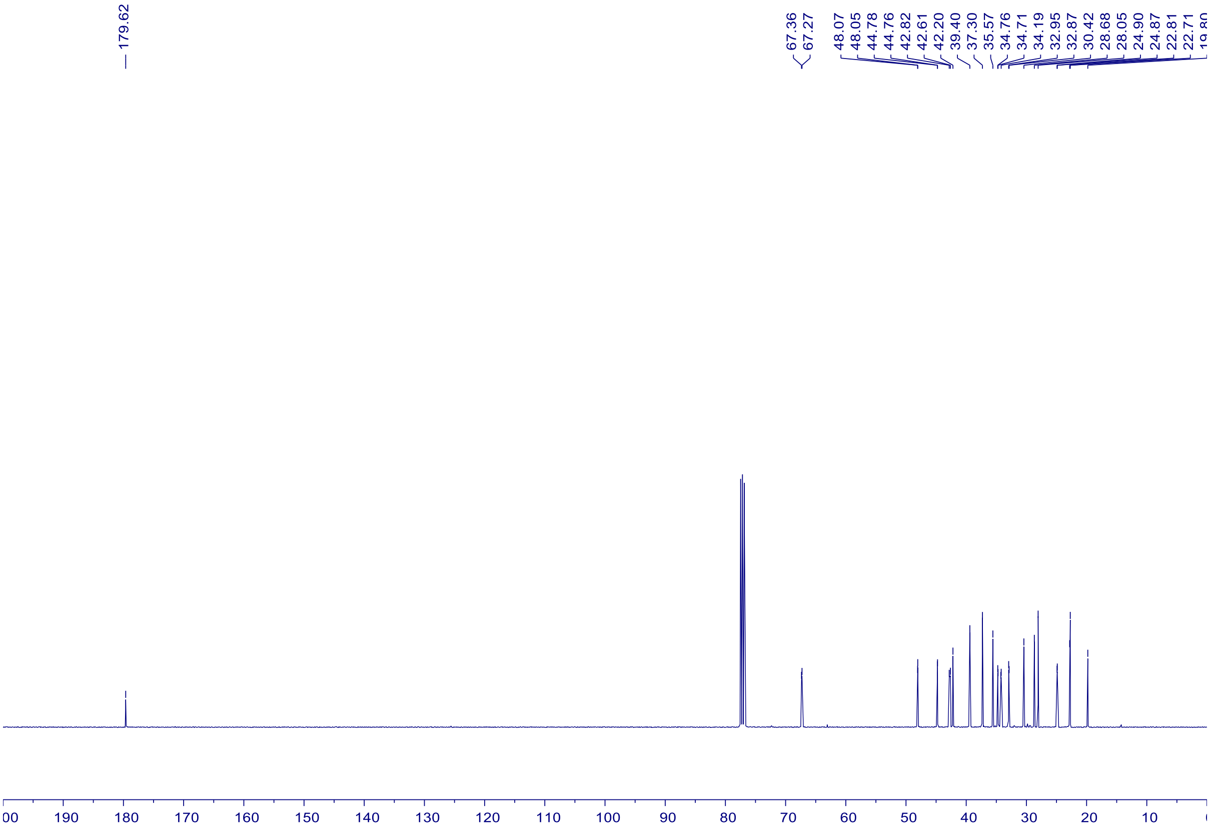

72 ^{1}H NMR (500 MHz, $CDCl_3$)

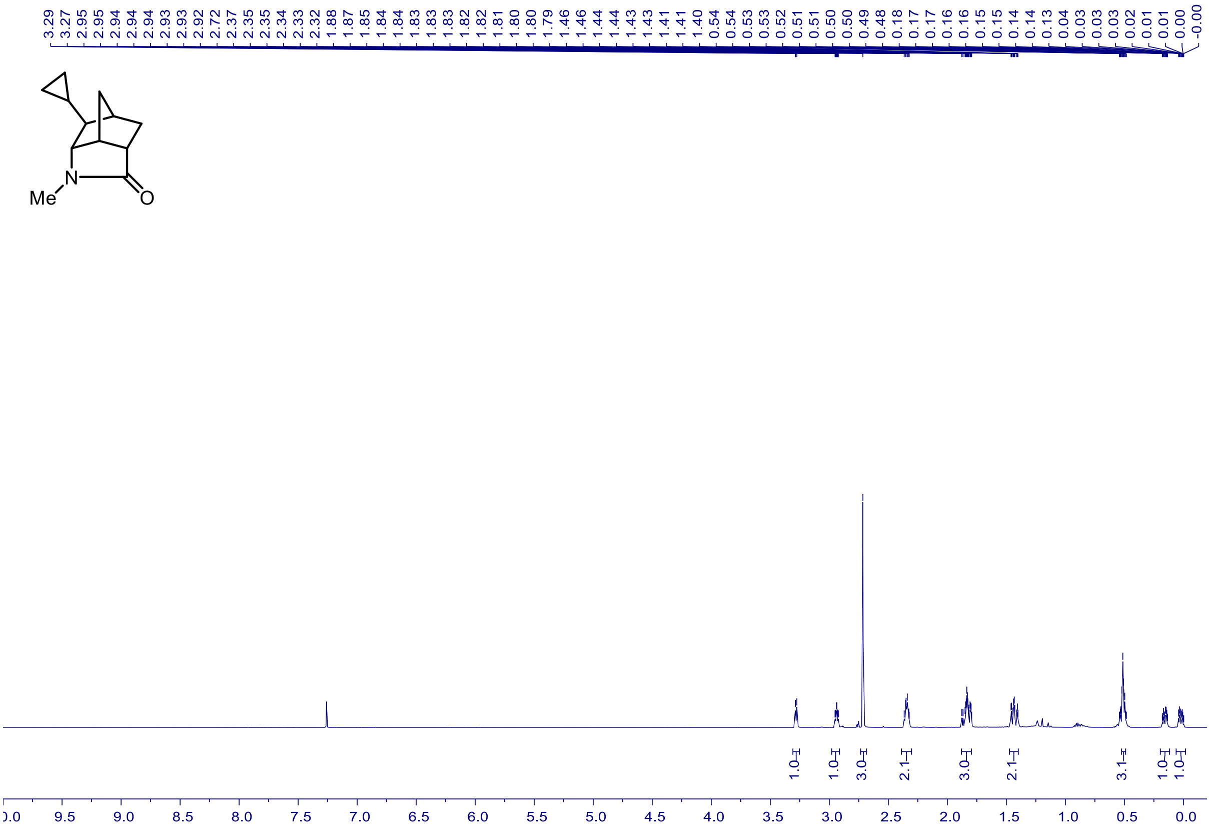

72 ^{13}C NMR (126 MHz, $CDCl_3$)

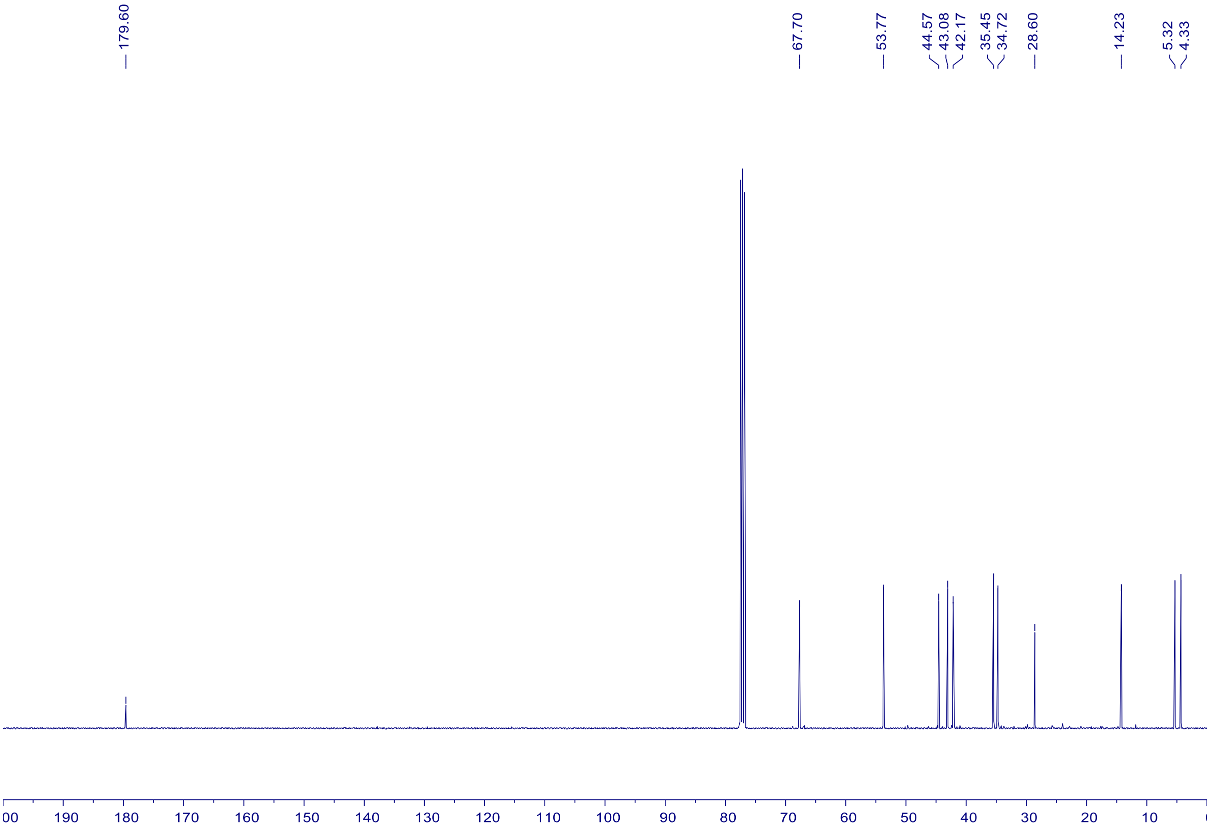

75 ^{1}H NMR (500 MHz, $CDCl_3$)

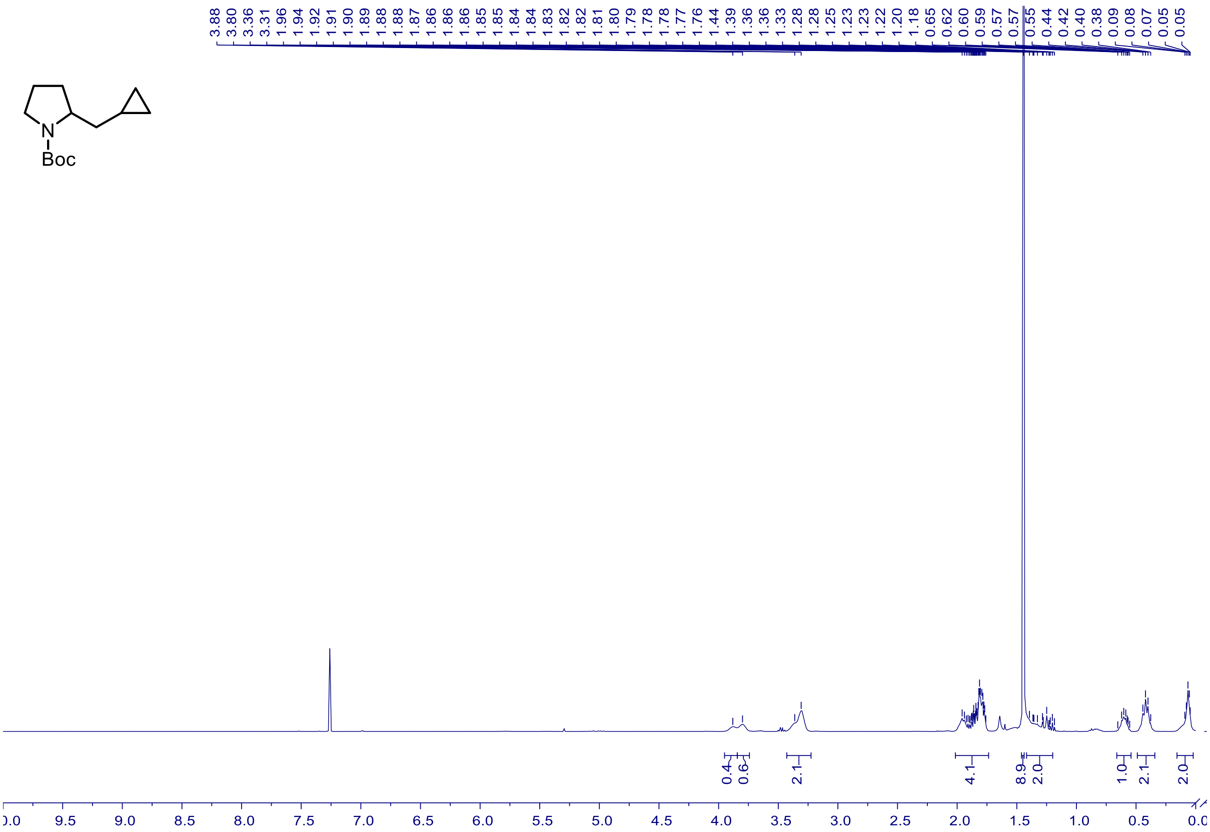

75 ^{13}C NMR (126 MHz, $CDCl_3$)

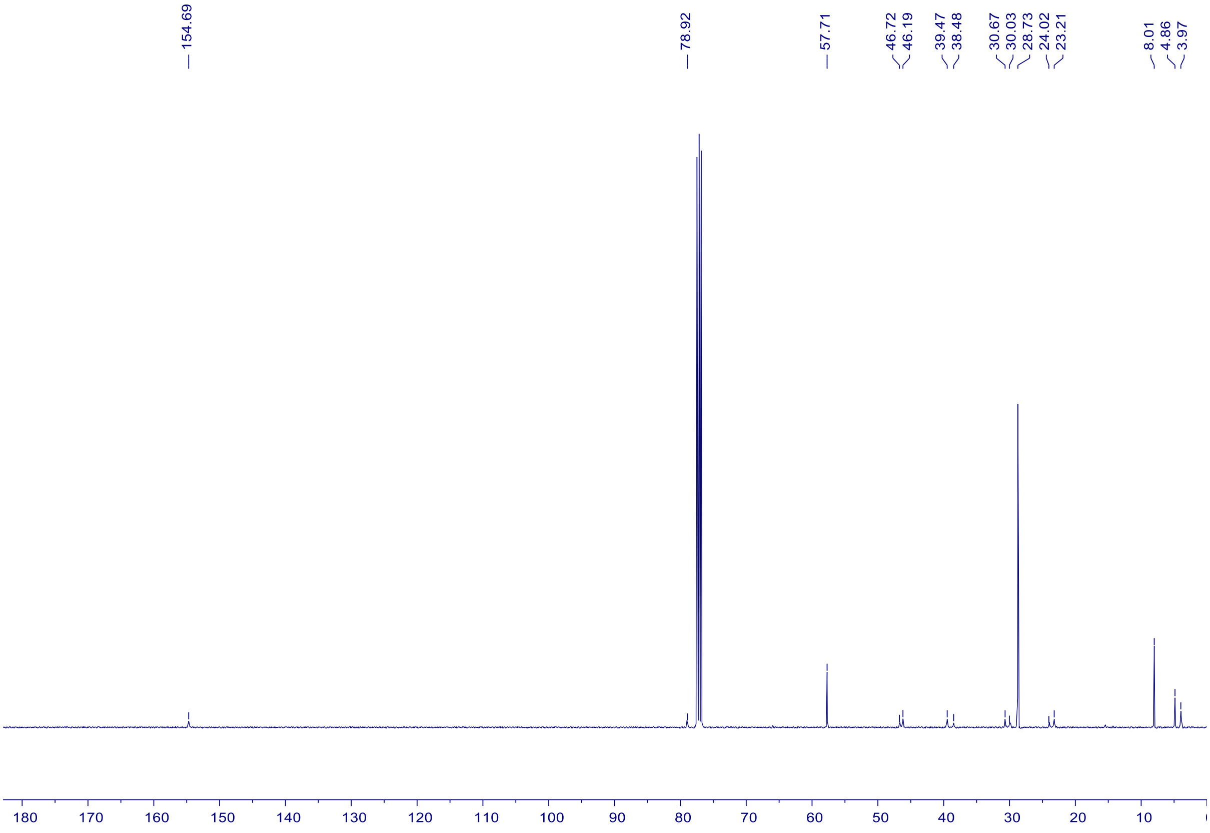

76 ^{1}H NMR (500 MHz, $CDCl_3$)

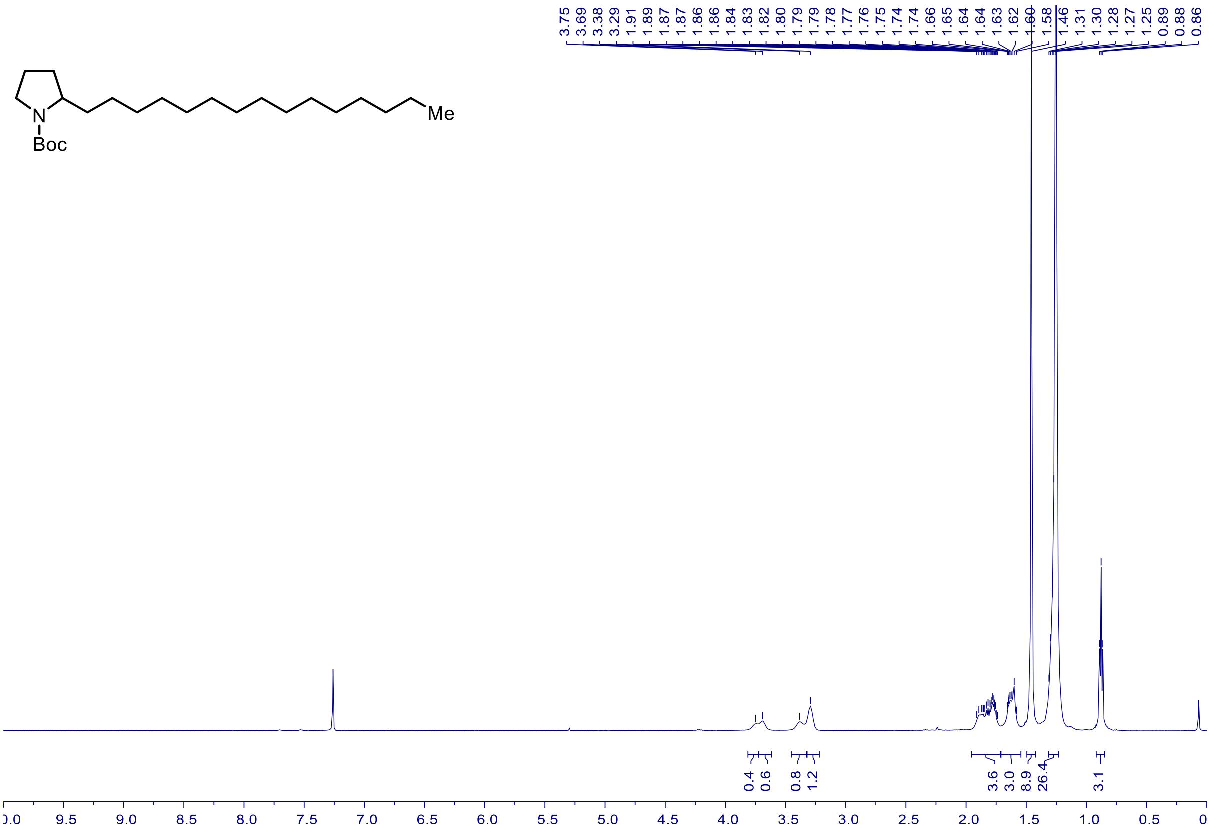

76 ^{13}C NMR (126 MHz, $CDCl_3$)

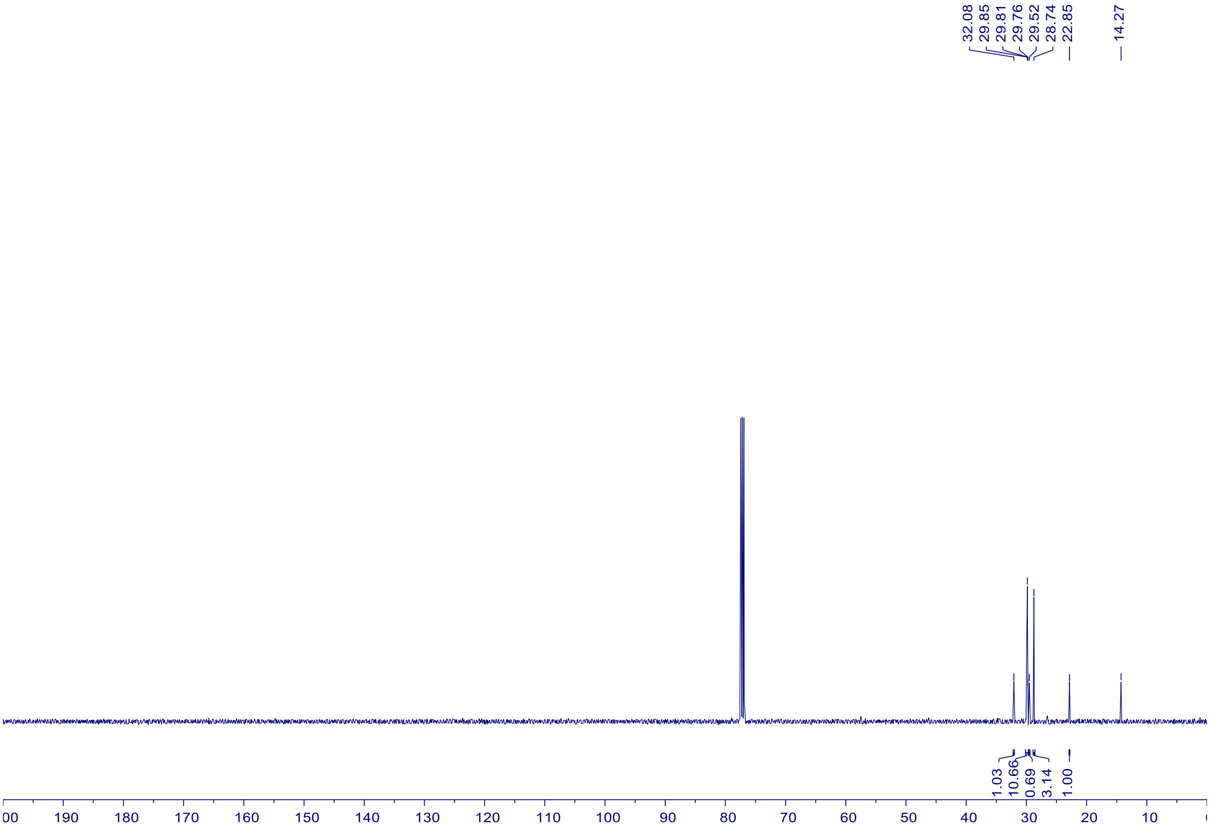

80 ^{1}H NMR (500 MHz, $CDCl_3$)

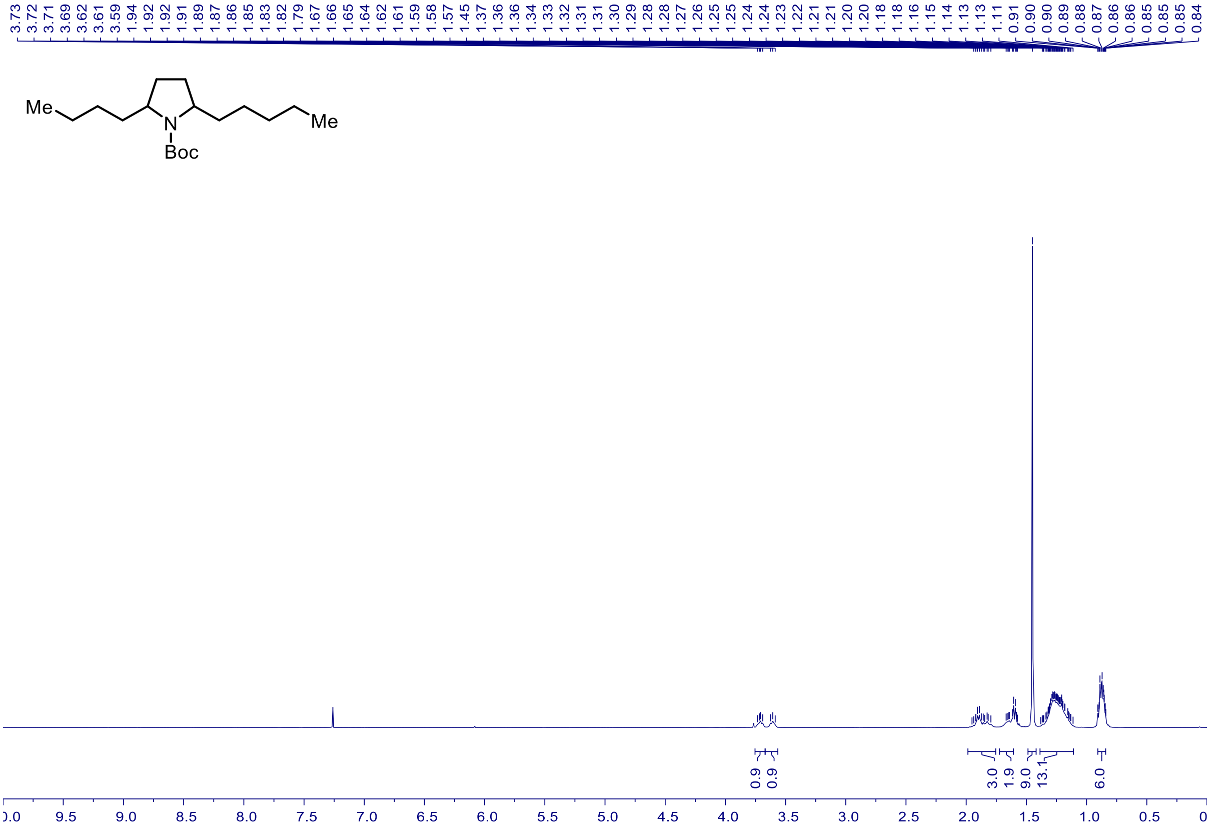

80 ^{13}C NMR (126 MHz, $CDCl_3$)

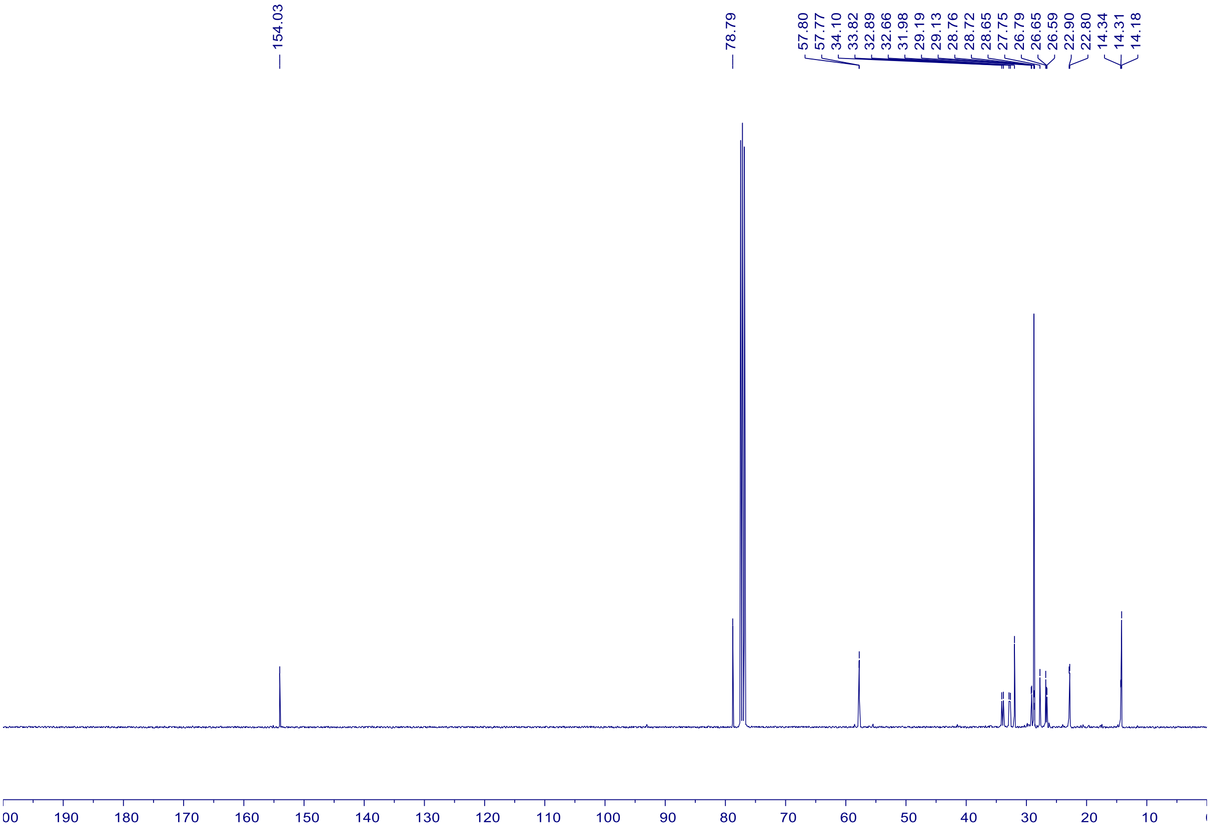

81 ^{1}H NMR (500 MHz, $CDCl_3$)

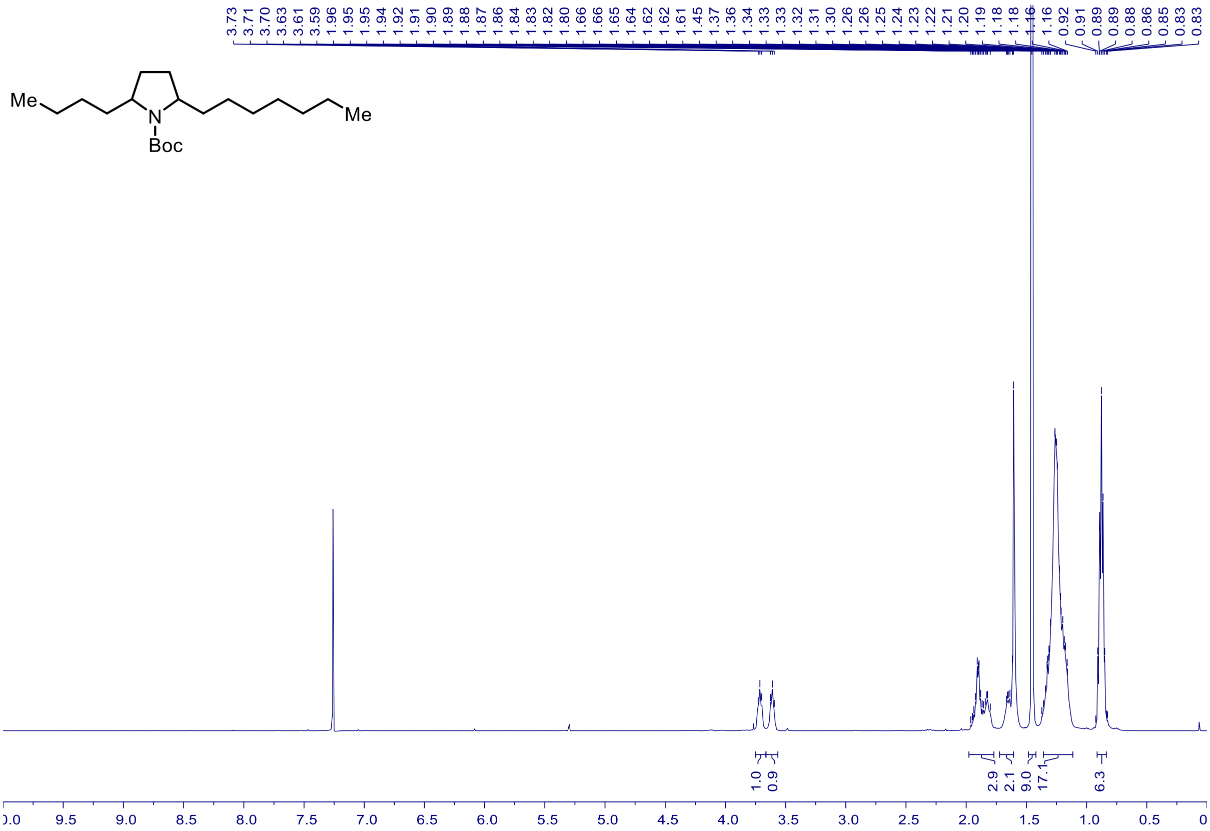

81 ^{13}C NMR (126 MHz, $CDCl_3$)

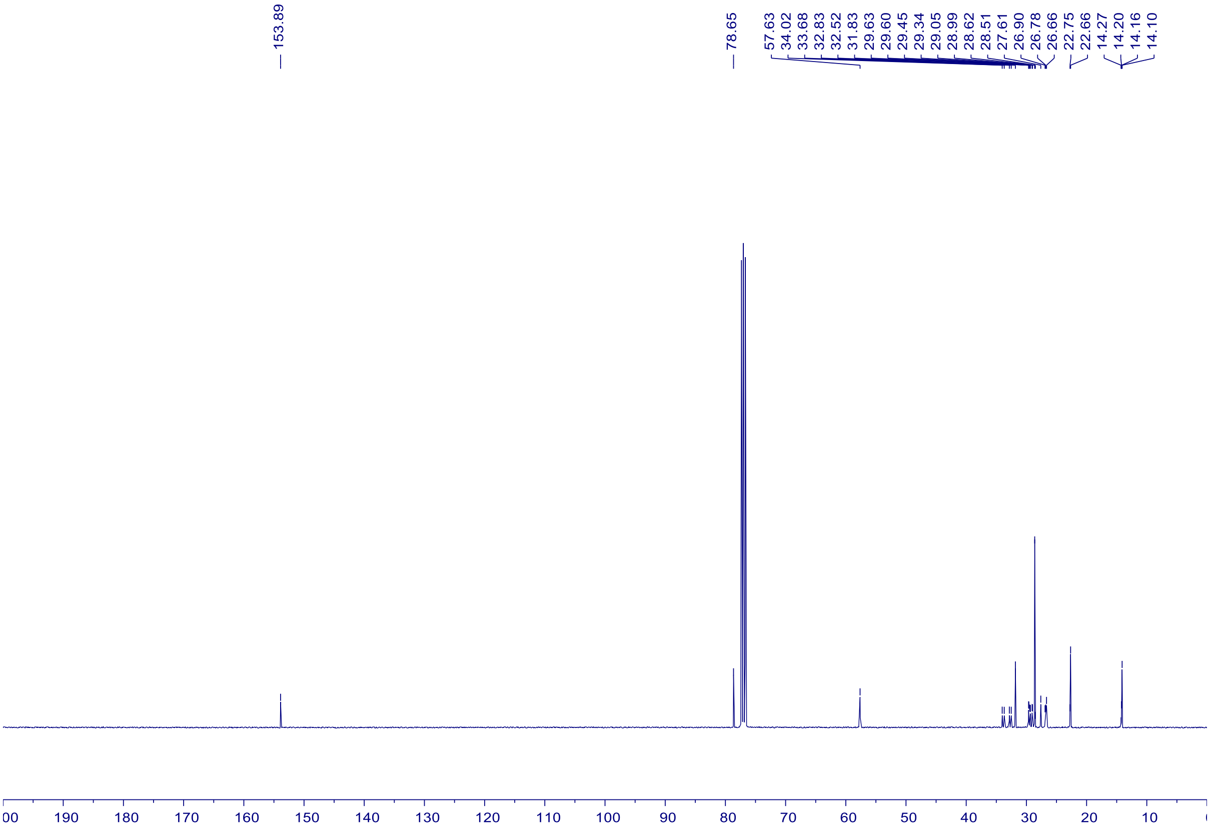

10 References

1 J. Davies, S. G. Booth, S. Essafi, R. A. W. Dryfe and D. Leonori, *Angew. Chem. Int. Ed*, **2015**, *54*, 14017–14021.

2 T. D. Svejstrup, A. Ruffoni, F. Juliá, V. M. Aubert and D. Leonori, *Angew. Chem. Int. Ed*, **2017**, *56*, 14948–14952.

3 A. Studer, C. G. Daniliuc and T. W. Greulich, *Org. Lett*, **2015**, *17*, 254–257.

4 P. Zhang, C. C. Le and D. W. C. MacMillan, *J. Am. Chem. Soc.*, **2016**, *138*, 8084–8087.

5 L. L. Anka-Lufford, K. M. M. Huihui, N. J. Gower, L. K. G. Ackerman and D. J. Weix, *Chem. - A Eur. J.*, **2016**, *22*, 11564–11567.

6 A. Banan, H. Valizadeh, A. Heydari and A. Moghimi, *Synlett*, **2017**, *28*, 2315–2319.

7 J. H. Horner, F. N. Martinez, O. M. Musa, M. Newcomb and H. E. Shahin, *J. Am. Chem. Soc.*, **1995**, *117*, 11124–11133.

8 J. Davies, T. D. Svejstrup, D. Fernandez Reina, N. S. Sheikh and D. Leonori, *J. Am. Chem. Soc.*, **2016**, *138*, 8092–8095.

9 E. J. Thomas and C. F. Vickers, *Tetrahedron Asymmetry*, **2009**, *20*, 970–979.

10 T. P. Blaisdell, T. C. Caya, L. Zhang, A. Sanz-Marco and J. P. Morken, *J. Am. Chem. Soc.*, **2014**, *136*, 9264–9267.

11 J. Cornella, J. T. Edwards, T. Qin, S. Kawamura, J. Wang, C. M. Pan, R. Gianatassio, M. Schmidt, M. D. Eastgate and P. S. Baran, *J. Am. Chem. Soc.*, **2016**, *138*, 2174–2177.

12 S. Vegiraju, B. C. Chang, P. Priyanka, D. Y. Huang, K. Y. Wu, L. H. Li, W. C. Chang, Y. Y. Lai, S. H. Hong, B. C. Yu, C. L. Wang, W. J. Chang, C. L. Liu, M. C. Chen and A. Facchetti, *Adv. Mater.*, **2017**, *29*, 1–8.

13 A. Krasovskiy and P. Knochel, *Synthesis (Stuttg).*, **2006**, 890–891.

14 E. Brenner, R. Scheider and Y. Fort, *Tetrahedron*, **1999**, *55*, 12829–12842.

15 M. Quadri, C. Stokes, A. Gulsevin, A. C. J. Felts, K. A. Abboud, R. L. Papke and N. A. Horenstein, *J. Med. Chem.*, **2017**, *60*, 7928–7934.

16 Z. Aouf, R. Mansouri, S. Lakrout, M. Berredjem and N.-E. Aouf, *J. Korean Chem. Soc.*, **2017**, *61*, 151–156.

17 C. G. McPherson, N. Caldwell, C. Jamieson, I. Simpson and A. J. B. Watson, *Chem. Commun.*, **2017**, *15*, 3507–3518.

18 Q. Xiao, L. Tian, R. Tan, Y. Xia, D. Qiu, Y. Zhang and J. Wang, *Org. Lett*, **2012**, *14*, 4230–4233.

19 P. Horrillo-Martínez, K. C. Hultzsch, A. Gil and V. Branchadell, *Eur. J. Org. Chem.*, **2007**, *2007*, 3311–3325.

20 Y. Yoshida, S. Otsuka, K. Nogi and H. Yorimitsu, *Org. Lett.*, **2018**, *20*, 1134–1137.

21 Y. Zhang, V. César, G. Storch, N. Lugan and G. Lavigne, *Angew. Chem. Int. Ed*, **2014**, *53*, 6482–6486.

22 J. C. Vantourout, R. P. Law, A. Isidro-Llobet, S. J. Atkinson and A. J. B. Watson, *J. Org. Chem.*, **2016**, *81*, 3942–3950.

23 Y. B. R. D. Rajesh, *J. Heterocycl. Chem.*, **2018**, *55*, 486–491.

24 Z. Dong, G. Lu, J. Wang, P. Liu and G. Dong, *J. Am. Chem. Soc.*, **2018**, *140*, 8551–

8562.

25 M. Klikar, V. Jelínková, Z. Růžičková, T. Mikysek, O. Pytela, M. Ludwig and F. Bureš, *Eur. J. Org. Chem.*, **2017**, *2017*, 2764–2779.

26 Z. Lu and R. J. Twieg, *Tetrahedron*, **2005**, *61*, 903–918.

27 P. Huang, Y. X. Wang, H. F. Yu and J. M. Lu, *Organometallics*, **2014**, *33*, 1587–1593.

28 J. C. Vantourout, H. N. Miras, A. Isidro-Llobet, S. Sproules and A. J. B. Watson, *J. Am. Chem. Soc.*, **2017**, *139*, 4769–4779.

29 H. Ma, D. Li and W. Yu, *Org. Lett.*, **2016**, *18*, 868–871.

30 C. P. Johnston, R. T. Smith, S. Allmendinger and D. W. C. MacMillan, *Nature*, **2016**, *536*, 322–325.

31 T. H. Jepsen, M. Larsen and M. B. Nielsen, *Tetrahedron*, **2010**, *66*, 6133–6137.

32 M. B. Bertrand and J. P. Wolfe, *Tetrahedron*, **2005**, *61*, 6447–6459.

33 C. Le, Y. Liang, R. W. Evans, X. Li and D. W. C. MacMillan, *Nature*, **2017**, *547*, 79–83.

34 S. Kamijo, K. Kamijo, K. Maruoka and T. Murafuji, *Org. Lett.*, **2016**, *18*, 6516–6519.

35 L. Yan, J. J. Hale, C. L. Lynch, R. Budhu, A. Gentry, S. G. Mills, R. Hajdu, C. A. Keohane, M. J. Rosenbach, J. A. Milligan, G. J. Shei, G. Chrebet, J. Bergstrom, D. Card, H. Rosen and S. M. Mandala, *Bioorganic Med. Chem. Lett.*, **2004**, *14*, 4861–4866.

36 I. Coldham and D. Leonori, *J. Org. Chem.*, **2010**, *75*, 4069–4077.

37 R. G. Parr and W. Yang, *Density-Functional Theory of Atoms and Molecules*; Oxford University Press, U.K., **1989**.

38 M. J. Frisch, G. W. Trucks, H. B. Schlegel, G. E. Scuseria, M. A. Robb, J. R. Cheeseman, G. Scalmani, V. Barone, B. Mennucci, G. A. Petersson, H. Nakatsuji, M. Caricato, X. Li, H. P. Hratchian, A. F. Izmaylov, J. Bloino, G. Zheng, J. L. Sonnenberg, M. Hada, M. Ehara, K. Toyota, R. Fukuda, J. Hasegawa, M. Ishida, T. Nakajima, Y. Honda, O. Kitao, H. Nakai, T. Vreven, J. A. Montgomery Jr., J. E. Peralta, F. Ogliaor, M. Bearpark, J. J. Heyd, E. Brothers, K. N. Kudin, V. N. Staroverov, T. Keith, R. Kobayashi, J. Normand, K. Raghavachari, A. Rendell, J. C. Burant, S. S. Iyengar, J. Tomasi, M. Cossi, N. Rega, J. M. Millam, M. Klene, J. E. Knox, J. B. Cross, V. Bakken, C. Adamo, J. Jaramillo, R. Gomperts, R. E. Stratmann, O. Yazyev, A. J. Austin, R. Cammi, C. Pomelli, J. W. Ochterski, R. L. Martin, K. Morokuma, V. G. Zakrzewski, G. A. Voth, P. Salvador, J. J. Dannenberg, S. Dapprich, A. D. Daniels, O. Farkas, J. B. Foresman, J. V. Ortiz, C. J. and D. J. Fox, *Gaussian 09*, revision E.01, Gaussian Inc., Wallingfort, CT, **2013**.

39 R. Denningtom, T. Keith and J. Millam, *GaussView*, version 5; Semichem Inc.; Shawnee Mission, KS, **2009**.

40 P. J. Stephens, F. J. Devlin, C. F. Chabalowski and M. J. Frisch, *J. Phys. Chem.*, **1994**, *98*, 11623–11627.

41 A. D. Becke, *J. Chem. Phys.*, **1993**, *98*, 5648–5652.

42 A. D. Becke, *J. Chem. Phys.*, **1993**, *98*, 1372–1377.

43 C. Lee, W. Yang and R. G. Parr, *Phys. Rev.*, **1988**, *37*, 785–789.

44 M. J. Cabrera-Afonso, Z. P. Lu, C. B. Kelly, S. B. Lang, R. Dykstra, O. Gutierrez and G. A. Molander, *Chem. Sci.*, **2018**, *9*, 3186–3191.

45 J. Liu, S. Niwayama, Y. You and K. N. Houk, *J. Org. Chem.*, **1998**, *63*, 1064–1073.
46. C. Shu, M.-Q. Liu, S.-S. Wang, L. Li, L.-W. Ye, *J. Org. Chem.*, **2013**, *78*, 7, 3292.
47. W. P. Teh, F. E. Michael, *Org. Lett.* **2017**, *19*, 1738.
48. F. A. Davis, M. Song, A. Augustine, *J. Org. Chem.*, **2006**, *71*, 2779.
49. Y.-F. Cheng, H.-J. Rong, C.-B. Yi, J.-J. Yao, J. Qu, *Org. Lett.*, **2015**, *17*, 4758.
